# Numerical investigation of ultrasound-induced acoustic streaming and shear stress for blood clot manipulation

**DOI:** 10.1038/s41598-026-44521-5

**Published:** 2026-04-20

**Authors:** Aisha Hisham, Mohammed A. Hassan, Ashraf A. Wahba

**Affiliations:** 1 Faculty of Computer Science and Engineering, Galala University, Suez, Egypt; 2 Biomedical Engineering Department, Faculty of Engineering, Capital University, Cairo, Egypt

**Keywords:** Acoustic streaming, COMSOL, Blood clot, Shear stress, Computational modeling, Diseases, Engineering, Health care

## Abstract

Venous thromboembolism affects over 10 million people each year, and research indicates that the mortality rate from hemorrhagic stroke is reaching 21%, and the disability rate is 41%. These statistics underscore the critical importance of early diagnosis and prompt therapeutic intervention to improve patient outcomes and mitigate the overall burden of the disease. This work aims to use computational simulation to model how acoustic waves affect blood clot disintegration inside blood vessels. Analysis was conducted to determine the effects of acoustic pressure, frequency, and the position of the transducer on the generation of acoustic streaming velocity near the thrombus surface. The suggested numerical model successfully generated several shear stress values, the highest of which was 10.894 Pa above the clot surface, due to acoustic streaming. This value is sufficient to cause thrombus fragmentation, as it exceeds the minimal threshold needed for clot disintegration, which is 4.1 Pa. Additionally, ultrasound was applied to thicker vessels, and the initial shear stress was observed to decrease to 2.6915 Pa. To increase this stress, the ultrasound parameters were adjusted, successfully raising the shear stress to 2.9763 Pa. This demonstrates that shear stress may have an indirect effect on clots. Realistic models, changeable properties, and experimental validation are necessary for future research.

**Supplementary Information:**

The online version contains supplementary material available at 10.1038/s41598-026-44521-5.

## Introduction

Thrombus formation is a critical physiological response to endothelial cell injury, hypercoagulability, abnormal blood flow dynamics, and other contributing conditions. However, the abnormal formation of thrombi can obstruct blood flow, leading to severe medical conditions such as stroke, myocardial infarction, and pulmonary embolism. Early diagnosis and treatment with anticoagulants reduce mortality rates and increase the chances of treatment^1^. But current thrombosis treatment methods, such as anticoagulant drugs or thrombolytic therapy, have limitations, including restrictions on giving the drug, and often carry risks, including bleeding complications and incomplete thrombus dissolution. To avoid these limitations, non-invasive techniques such as ultrasound-based thrombus treatment are gaining increasing attention for their potential to enhance thrombus dissolution safely and efficiently^2^.

Venous thrombotic disease (VTD) is a significant global health concern, impacting millions of individuals annually. It encompasses conditions such as deep vein thrombosis, characterized by the formation of thrombi within the venous system. VTD is ranked as the third most prevalent vascular disorder worldwide, following myocardial infarction and stroke, with approximately ten million new cases reported each year^3^.

Research indicates that the mortality rate from hemorrhagic stroke is reaching 21%, and the disability rate, as measured by the Barthel index, is reaching 41%. These statistics underscore the critical importance of early diagnosis and prompt therapeutic intervention to improve patient outcomes and mitigate the overall burden of the disease^3^.

Ultrasound’s mechanical effects on biological tissues have been extensively studied as a therapeutic technique^4^. Through processes like acoustic streaming, cavitation, and direct mechanical vibration, the use of ultrasonic waves in thrombolysis has demonstrated promise in improving the disintegration of clots^5^.

Previous studies have explored various ultrasound-based techniques to enhance thrombus dissolution, but they depend on some clot-dissolving medications, such as recombinant tissue plasminogen activator (rt-PA)^6^. Among these methods, cavitation therapy has garnered attention due to its ability to generate microbubbles that produce localized mechanical stress on the clot surface^7,8^. Another approach involves microbubble (MB) or nanodroplet (ND) mediated ultrasound thrombolysis^8^. This method leverages the oscillation and collapse of MBs or NDs under ultrasonic waves to create mechanical forces that disrupt the thrombus structure, improving drug penetration and enhancing thrombolysis^9^. These studies have mostly concentrated on techniques that combine ultrasound and thrombolytic drugs, which may have some complications, such as bleeding^6^. Dual-frequency ultrasound thrombolysis employs two frequencies (low for drug-loaded droplet penetration, high for droplet cavitation) to cooperatively improve disintegration, while catheter-mediated ultrasound thrombolysis delivers energy directly to the clot to enable targeted drug delivery^10–13^ Notwithstanding their effectiveness, these methods have drawbacks in terms of drug administration and higher patient costs. This makes it evident that new, non-invasive, and drug-free ultrasound technologies that can achieve thrombus fragmentation are needed.

This study focuses on a particularly interesting mechanism: acoustic streaming^3^. This method creates shear stress on the thrombus surface by creating vortex movements in the fluid surrounding the thrombus. Its structural disintegration and eventual dissolution are facilitated by this mechanical stress, providing a new and focused method to improve thrombolytic efficacy.

Our goal is to better understand and enhance this mechanism, emphasizing its potential as a successful non-invasive thrombus therapy technique. The continuous flow of fluid caused by ultrasonic vibrations propagating through a material is referred to as “acoustic streaming.” Shear pressures produced by this event may aid in the breakdown of fibrin networks inside the clot, promoting clot fragmentation.

This research has shown that the amount of shear stress produced by acoustic streaming can overcome the blood clots’ mechanical strength, encouraging their disintegration and breakup^4^. To investigate this phenomenon, COMSOL Multiphysics^®^ (version 6.2) was used to construct a blood clot model and analyze the results after applying ultrasonic waves to the clot. The modeling of intricate physical phenomena, which include acoustic streaming and its relationship to fluid dynamics, is made possible by the potent simulation program COMSOL. Utilizing this software’s capabilities, we developed a numerical model to predict the fluid flow around a blood clot exposed to ultrasonic waves and simulate the acoustic pressure field^3,4^.

The study’s goal is to examine how different ultrasonic factors, including frequency, pressure amplitude, and transducer position, affect the shear stress produced to break down the clot. Our method combines computational techniques to solve the Navier-Stokes equations for fluid dynamics and the second-order acoustic field equations. With a focus on determining the most suitable conditions for clot disintegration, the simulated results provide light on the association between ultrasound parameters and the mechanical forces of streaming acting on the clot.

Building on the foundational work of previous studies, this enabled us to select a research topic and develop an acoustic streaming model^3^. Furthermore, this study integrates a clot geometry into the computational domain to allow us to experiment with various acoustic parameters, such as frequencies and acoustic pressures, to advance the understanding of acoustic thrombus interactions^1,14,15^. The findings of this study have implications for the development of optimal ultrasound-mediated thrombolysis therapy and non-invasive therapy methods.

## Numerical simulation model

**Fig. 1:**
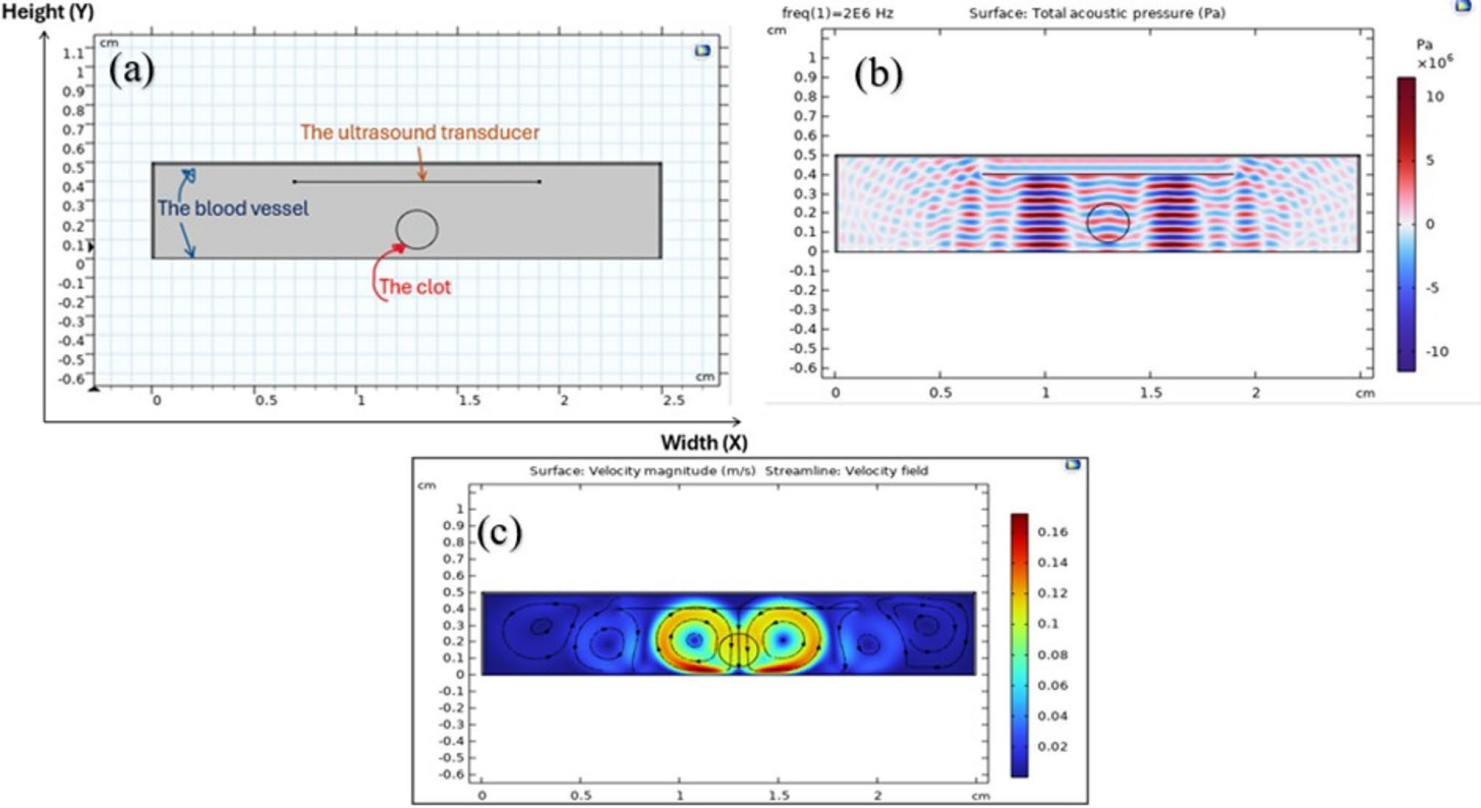
**a** Simulation model of the blood clot in the blood vessel using COMSOL Multiphysics as a rectangular domain of 0.5 cm in height(Y) and 2.5 cm in width(X). **b** Sound waves in the form continuous acoustic wave, the acoustic field is solved with Pressure Acoustics, Frequency domain. **c** The fluid flow is modeled with the Laminar Flow interface and acoustic field (Multiphysics coupling Acoustic Streaming Domain Coupling).

In the current investigation, a numerical simulation was performed using the COMSOL Multiphysics software (version 6.2) to describe the interaction of ultrasound waves with a clot. A two-dimensional (2D) model was employed to represent the geometry of the vessel and thrombus, enabling an extensive exploration of acoustic interactions and flow dynamics within the system. The blood vessel wall was modeled as a rigid and non-deforming domain and has been demonstrated as a rectangular domain of 0.5 cm in height(Y) and 2.5 cm in width(X). The thrombus was modeled as a viscous fluid and has been demonstrated as an ellipse within the vessel, with semi-major and semi-minor axes of 0.1 cm, as shown in Fig. 1(a). The ultrasound transducer is modeled as a straight-line source with a width of 1.2 cm, which was excited with a continuous acoustic signal, as the model was solved in the frequency domain^16^. This approach allowed us to analyze the steady-state acoustic and fluid dynamics. Ultrasound waves at a frequency of 2 MHz and an acoustic pressure of 2 MPa were applied. To accurately simulate the acoustic-thrombus interaction, the transducer was positioned within the vessel in our numerical model. This allows for a focused investigation into the direct effects of acoustic waves and fluid dynamics on the clot itself^3^. This methodology provides a controlled environment to isolate the primary physical phenomena, which is a crucial foundational step before advancing to more clinically representative models in future work. A Perfectly Matched Layer (PML) was applied to the sides and upper surface of the blood vessel to prevent wave reflection. This approach was a preliminary step to simplify computations, allowing us to directly study the interaction between acoustic waves and the clot. We acknowledge that the transducer is external in reality, and the use of PML on the upper surface helped to simulate more realistic wave propagation conditions^3^.

### Material properties and initial boundary conditions

Standard characteristics were used to characterize the material properties of the fluid, which was modeled as water inside the vessel, as in Table 1^3,4,14^.

**Table 1:** Material Properties of Water and Vessel.

Material Properties of Water	The value
Density (₀)	998 [kg/m^3]
Sound speed (c₀)	1495 [m/s]
Bulk viscosity (µB)	0.00247[Pa*s]
dynamic viscosity(µ)	0.000893[Pa*s]
Thermal expansion coefficient	0.000297[1/K]
Specific heat capacity	4183[J/(kg* K)]
Thermal conductivity	0.603[W/(m* K)]
Specific heat ratio	1
Compressibility β0	4.49 × 10^ (− 10) [Pa^ (− 1)]

Material Properties of the vessel	The value
Density (${{\uprho}}_{v}$)	1053[kg/m^3]
Sound speed (${c}_{v}$)	1650[m/s]
The dynamic viscosity of the thrombus	0.4 Pa·s

With an initial velocity of zero across the domain, beginning conditions presupposed a stationary state^3^. The vessel walls were subjected to boundary conditions, assuming them to be rigid and non-deforming. Because the ultrasound transducer was set up to produce a continuous acoustic wave consistently, as shown in Fig. 1(b), the acoustic pressure remained constant during the simulation.

### Governing equations used in the model

The two main physics interfaces offered by COMSOL Multiphysics, the Laminar Flow interface and the Pressure Acoustics, Frequency Domain interface, served as the foundation for numerical simulation^17–19^.

The dynamic behavior of the fluid and the propagation of the ultrasound waves were simulated using these interfaces^20^. Every domain in the simulation, including the water and clot, was subjected to the Pressure Acoustics Frequency Domain interface. The Helmholtz equation describes how acoustic waves propagate in these complex media. The equation is expressed as^21,22^:

1 $$\nabla\left(\frac{1}{\rho}\nabla P-\frac{{F}^{\mathrm{*}}}{\rho}\right)+\frac{{{K}_{eq}}^{2}}{\rho}P=0$$

2 $${{K}_{eq}}^{2}={\left(\frac{\omega}{c}\right)}^{2}-{{K}_{Z}}^{2}$$

where ρ is the medium’s density, P is the acoustic pressure, ${F}^{*}$ it is an acoustic driving force, used to describe a nonlinear wave equation for the behavior of the acoustic field. It also includes background volumetric force^21^. Equation (2) provides the modified wave number ${{K}_{eq}}^{2}$it represents the complex acoustic pressure field. It is adjusted to incorporate additional physical effects that impact wave propagation within a fluid. This allows the model to account for various nonlinear effects and other complex phenomena, making the simulation more accurate and realistic.

ω is the ultrasound wave’s angular frequency, which is defined as ω = 2π f, where f is the frequency, c is the speed of sound in the medium, and it is possible to specify an out-of-plane wave number ${K}_{Z}$^23^. By disregarding the influence of weaker, accompanying sound waves and presuming that the dominant sound wave prevails, this approach simplifies the issue. The oscillatory nature of the acoustic waves is captured by this equation, which is a second-order partial differential equation.

The Laminar Flow interface, which solves the continuity equation for mass conservation and the Navier-Stokes equations for momentum conservation, was used to simulate the fluid flow of water and a clot. It solves in a stationary state^17^. The formula for the Navier-Stokes equations is:

3 $$\rho\left(\frac{\partial u}{\partial t}+u.\nabla u\right)=-\nabla P+\mu{\nabla}^{2}u+{f}_{aco}$$

where ρ is the density,$u$ is the velocity vector of the fluid, $\mu$ is the dynamic viscosity, ${f}_{aco}$ is the acoustic body force^17^. Initially, the Pressure Acoustics interface was used to solve the acoustic field in the frequency domain. The fluid flow simulation was then modeled using the Laminar Flow interface via a Multiphysics Acoustic Streaming Domain Coupling, with the computed acoustic field acting as an input^24^ Fig. 1(b) and (c) show that.

A coupling equation governed the interaction between the acoustic field and fluid flow. The acoustic body force in second order, where Γ is the dissipation coefficient^25^. Is given as:

4 $${f}_{aco}=\frac{\varGamma\omega}{{c}^{2}} \left\langle {v1p1} \right\rangle$$

Where ω is the angular frequency, c ​is the speed of sound, $p1$ is the first-order acoustic pressure, $v1$ is the first-order acoustic particle velocity.

## Results

### Acoustic streaming and shear stress analysis

**Fig. 2:**
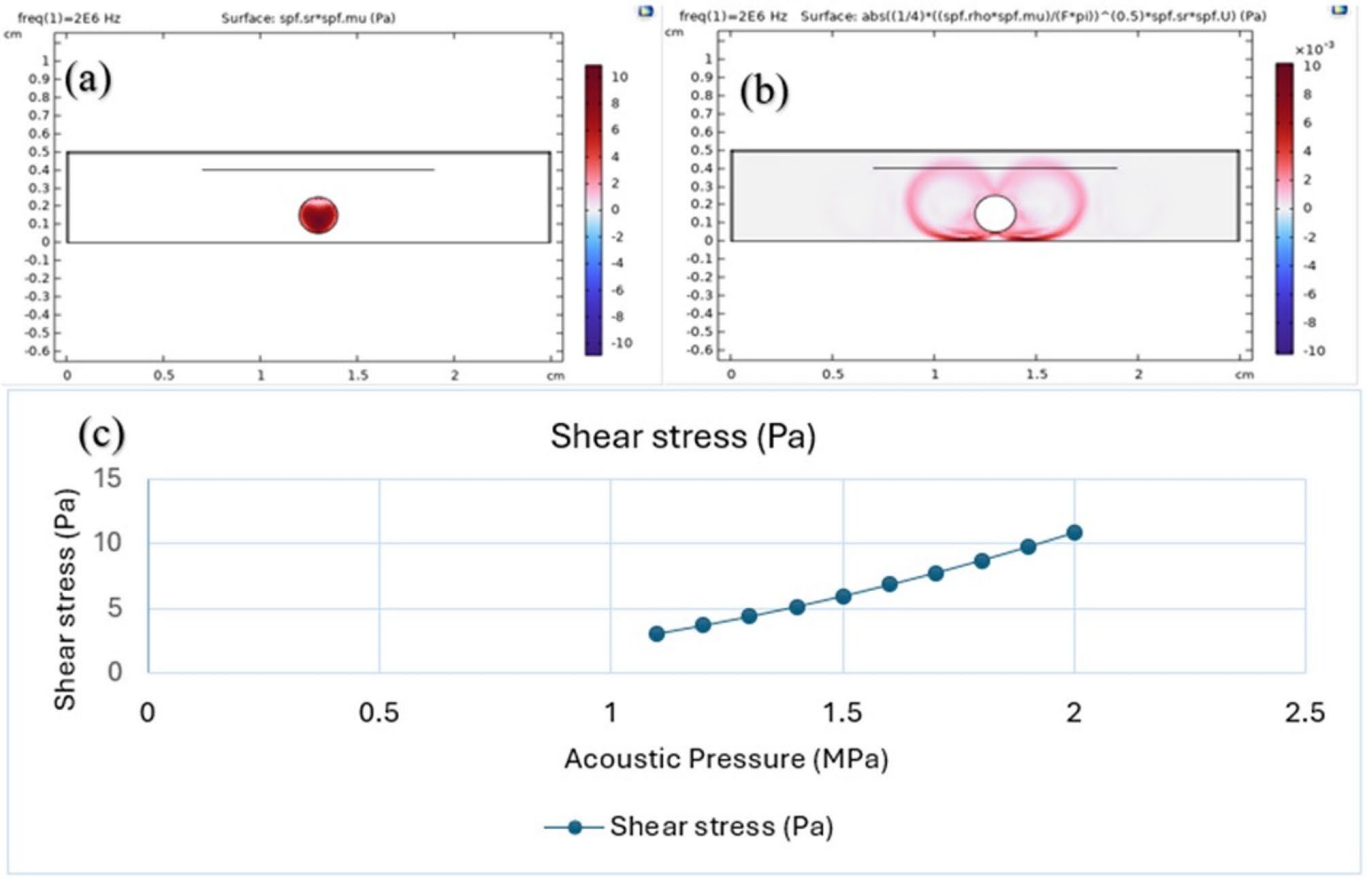
**a** Shear stress within the clot resulting from acoustic pressure. **b** Shear stress in the surrounding fluid resulting from fluid flow. **c** The results in the graph show that as the acoustic pressure increases from 1.1 MPa to 2 MPa, the shear stress increases.

The effect of sound waves on blood clots was examined numerically using COMSOL Multiphysics. The computational model used the Laminar Flow physics module and the Pressure Acoustics, Frequency Domain interface to simulate how ultrasound waves interact with a clot. The simulation illustrated the generation of acoustic streaming and the ensuing shear stress on the clot surface. The acoustic field interacts with the fluid, generating streaming flows. This acoustic streaming forms vortices near the surface of the clot. As these vortices flow, they induce shear stress on the surface of the clot. A mathematical formula can be employed to represent shear stress^26^:

5 $$\left(\boldsymbol{\tau}=\boldsymbol{\mu}\frac{\boldsymbol{d}\boldsymbol{v}}{\boldsymbol{d}\boldsymbol{z}}\right)$$

where is the dynamic viscosity of the fluid (Pa·s), / is the shear rate of fluid velocity (in s − 1). By raising the acoustic pressure from 1.1 MPa to 2 MPa and measuring the shear stress at each value, it was found that the shear stress increases with increasing acoustic pressure, as shown in Fig. 2 (c). This effect resulted in the highest shear stress value of up to 10.894 Pa, which may lead to the rupture of the fibrin network and facilitate thrombolysis^26^.

### The impact of acoustic streaming induced by ultrasound waves on the clot was investigated by varying several parameters

The parameters affecting the acoustic streaming included the clot’s position relative to the transducer, the frequency of the ultrasound waves, and the acoustic pressure.

#### Effect of the clot’s position

**Fig. 3:**
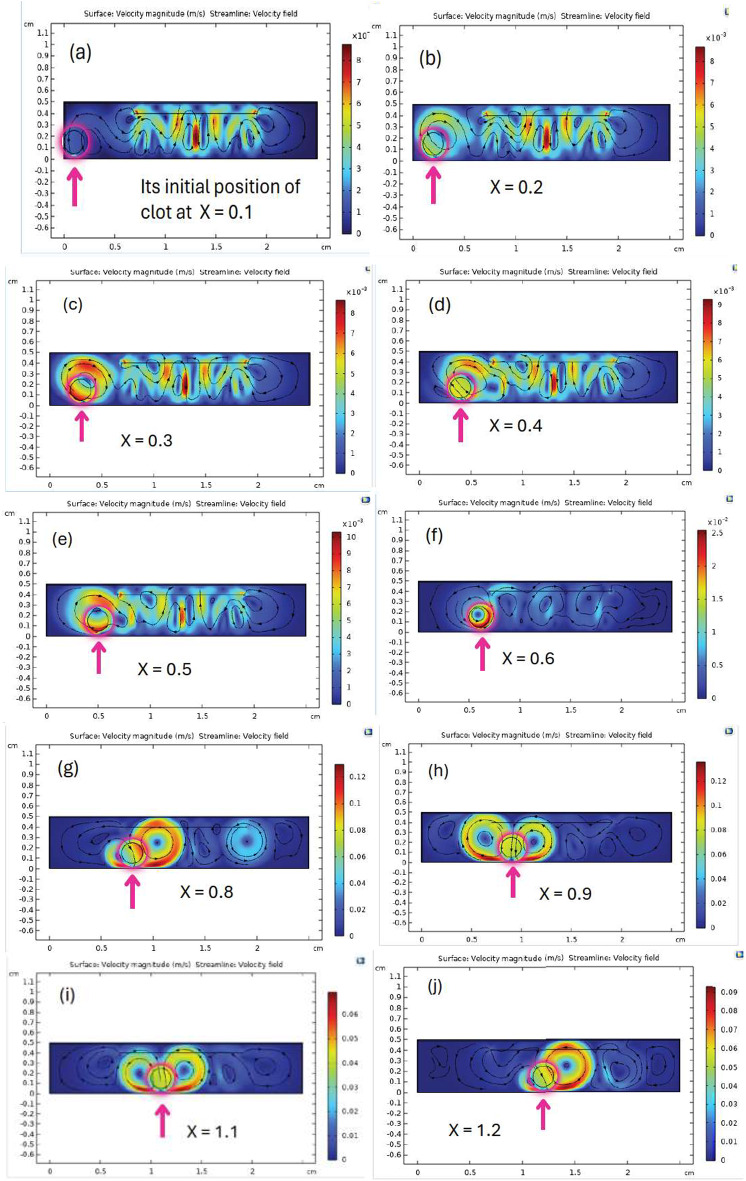

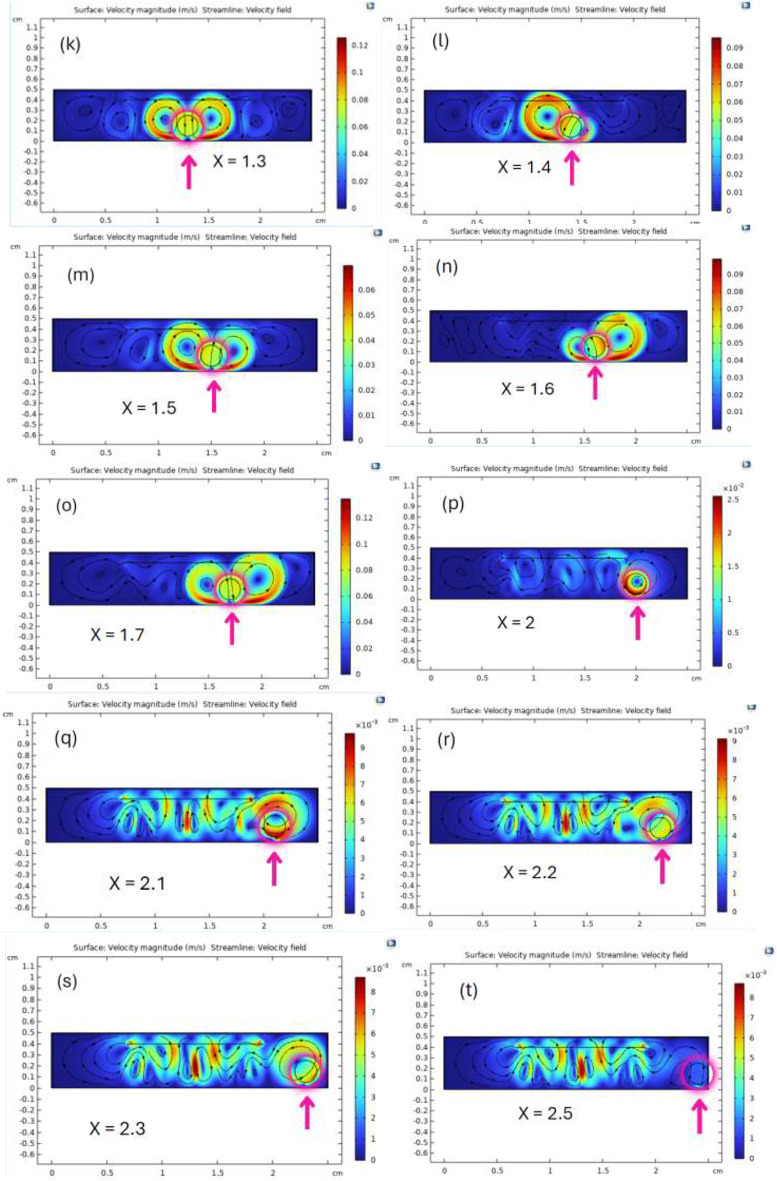
The spatial distribution and magnitude of the acoustic streaming velocity on the clot surface are shown for different transducer-to-clot distances. The figures from (a) to (t) illustrate the effect of varying the transducer’s position relative to the clot from 0.1 cm to 2.5 cm on the resulting flow patterns and velocities.

When applying an acoustic pressure of 1.5 MPa and a frequency of 2 MHz, varying the clot’s position along the x-axis from x = 0.1 cm to x = 2.5 cm produced significant changes in streaming velocity, as shown in Fig. 3.

The velocity magnitude field and streamlines of the velocity field produced by ultrasound-induced acoustic streaming within the computational model are illustrated in Fig. 3.

The velocity magnitude can be observed by the color scale in the figure, where blue parts denote lower velocities close to zero and red regions represent the highest velocities, which can reach 0.12 m/s. Maximum velocity is observed around the thrombus and its immediate surroundings in Fig. 3(g), (k), (o), particularly at the center of the simulation domain. The streamlines, shown as black lines, depict the flow direction and pattern of acoustic streaming, revealing symmetrical vortices forming around the thrombus. Two prominent pairs of vortices appear on either side in Fig. 3(k), reflecting the localized disturbance caused by the thrombus. The thrombus is positioned near the center of the domain at approximately 1.3 cm, where its effect on the flow field is most pronounced, leading to higher velocity fields in the surrounding medium.

Near the lateral boundaries of the domain in Fig. 3(a) to (f), (p) to (t), the velocity magnitude decreases, and the streamlines appear less affected, indicating a minimal influence of the ultrasound in these regions.

This visualization emphasizes the significant role of the ultrasound transducer position in shaping the velocity magnitude and flow structure on the thrombus, underlining the importance of precise positioning for optimizing the effects of acoustic streaming.

**Fig. 4:**
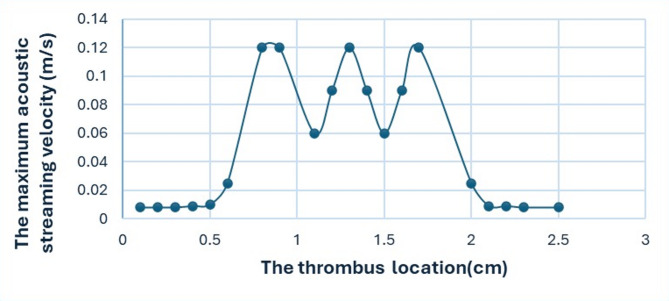
The spatial variance characteristic of a standing wave pattern is demonstrated by the maximum acoustic streaming velocity (m/s) over the clot surface at different positions for the clot extracted from the color scale in Fig. 3.

The acoustic streaming intensity is not linearly correlated with the thrombus position, as shown in Fig. 4, indicating a more complex interaction between the ultrasonic waves and the clot. The peaks and troughs observed in the data suggest the presence of standing wave patterns within the medium, which can significantly influence the fluid flow.

The distinct red and blue regions in Fig. 1(b), representing positive and negative acoustic pressures, reveal the wave pattern generated by the transducers. The grey lines, corresponding to pressure nodes, provide insight into standing wave formation and interference patterns within the medium.

**Fig. 5:**
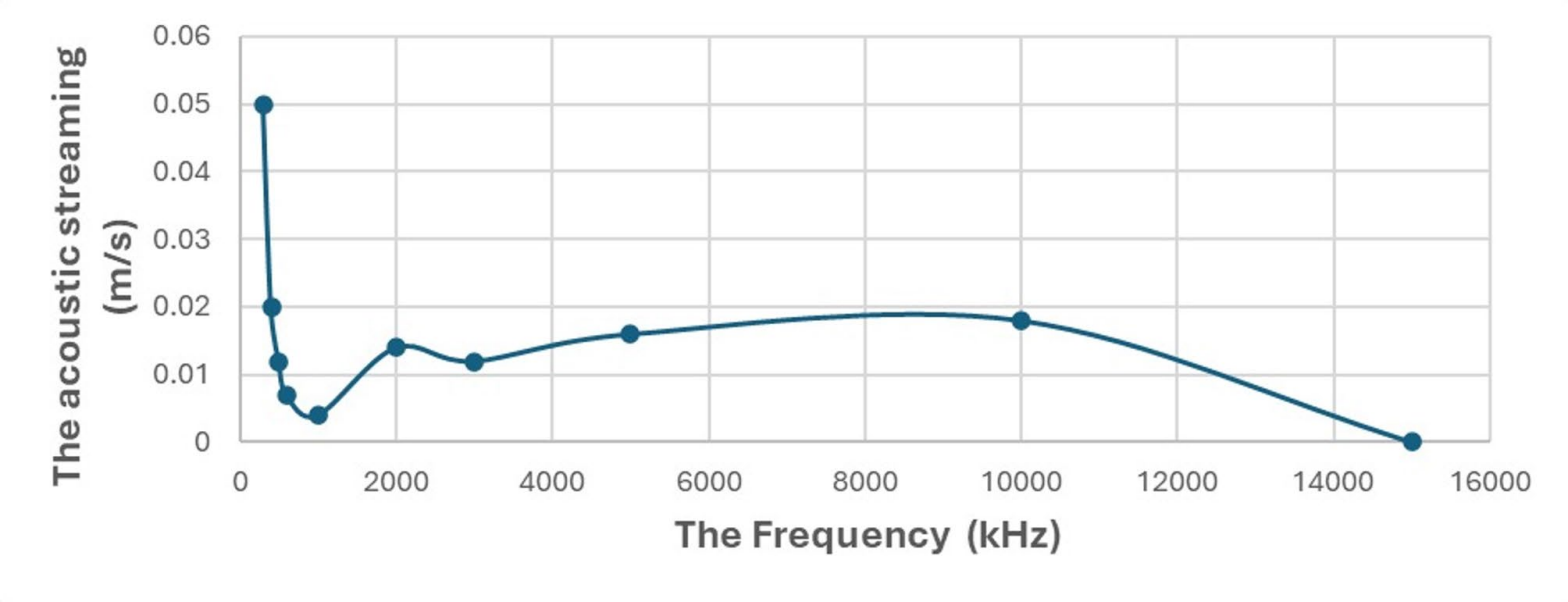
Acoustic streaming velocity (m/s) as a function of frequency (kHz), demonstrating a decrease in streaming velocity with increasing frequency from 300 kHz to 1 MHz, and irregular behavior from 1 MHz to 15 MHz at a constant acoustic pressure of 320 kPa and thrombus location X = 1.3 cm.

#### Effect of frequency variation

**Fig. 6:**
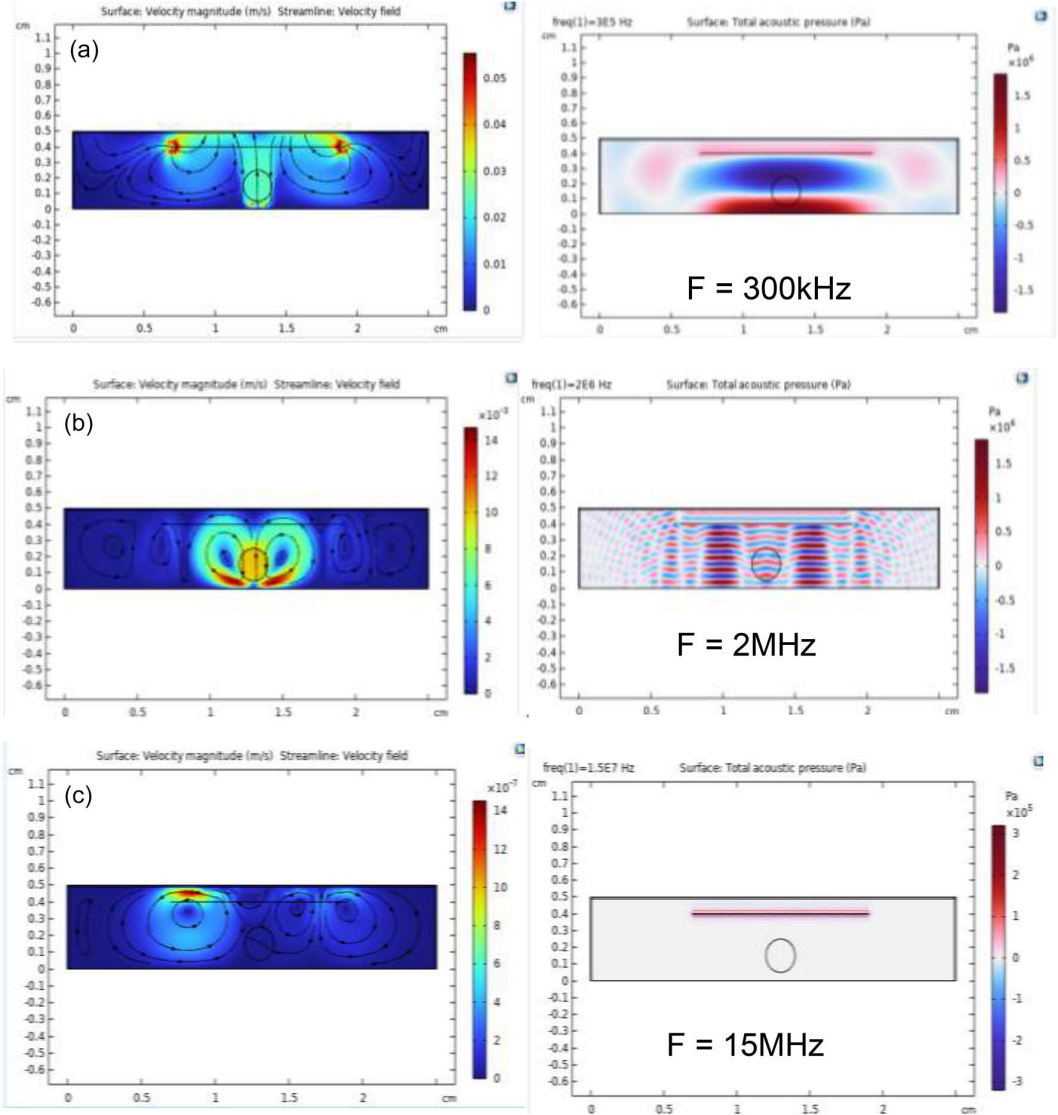
This figure illustrates the effect of different frequencies on acoustic streaming velocity and the acoustic pressure field: **a** at 300 kHz, **b** at 2 MHz, and **c** at 15 MHz.

By fixing the Acoustic Pressure at 320 kPa and the thrombus location X = 1.3 cm and altering the Frequency, the following results were observed in the frequency range from 300 kHz to 15 MHz: It is observed that with increasing frequency from 300 kHz to 1 MHz, the acoustic streaming decreases, but with increasing frequency from 1 MHz to up 15 MHz values, irregular behavior occurs. as shown in Figs. 5 and 6.

The results showed that the acoustic streaming velocity is significantly affected by the applied frequency. While low frequencies, such as 300 kHz, shown in Fig. 6(a), may generate high streaming velocities, their use in therapeutic applications may cause an increased risk of uncontrolled acoustic cavitation and poor energy focus^15^. Conversely, frequencies above 2 MHz, such as the 15 MHz shown in Fig. 6(c), exhibit high attenuation, leading to unwanted tissue heating and a reduced effect on the thrombus. Therefore, our results suggest that the 2 MHz frequency shown in Fig. 6(b) represents an ideal balance, providing sufficient and effective acoustic streaming to promote thrombolysis while mitigating the risks of excessive cavitation and unwanted heating, thus supporting its use in therapeutic applications^27^.

#### Impact of acoustic pressure variation

**Fig. 7:**
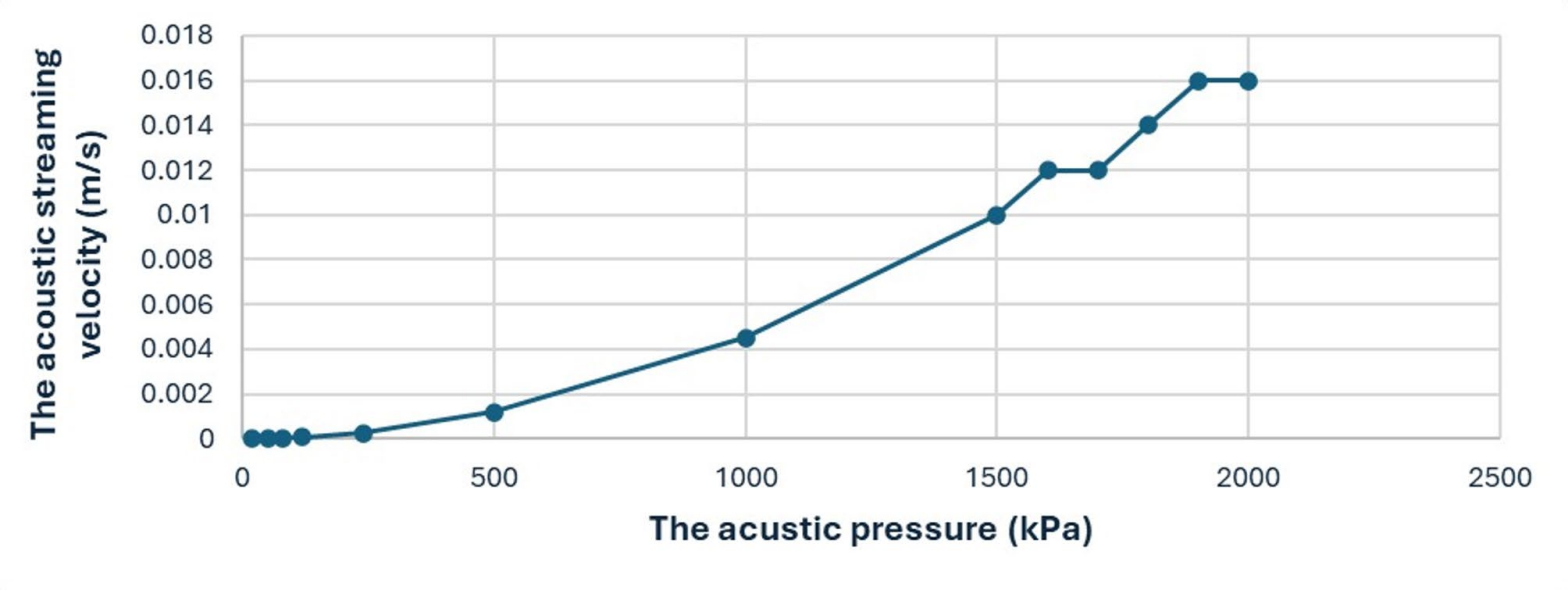
The acoustic streaming velocity (m/s) increases with increasing acoustic pressure (kPa) at a fixed clot location (X = 1.3 cm) and frequency (300 kHz). The acoustic streaming velocity exhibits stabilization at acoustic pressure values of 1.7 MPa and 2 MPa.

To investigate the effect of acoustic pressure, the thrombus location was held constant at X = 1.3 cm, and the frequency at 300 kHz. Under these controlled conditions, the results show that the acoustic streaming velocity increases monotonically with rising acoustic pressure, across the tested range of 20 kPa to 2 MPa. However, the acoustic streaming velocity shows a state of stabilization at acoustic pressure values of 1.7 MPa and 2 MPa, which suggests that the fluid’s resisting viscous forces have reached a state of balance with the increasing acoustic pressure^28^, as shown in Fig. 7.

### Model of applying ultrasound on the thicker vessels

**Fig. 8:**
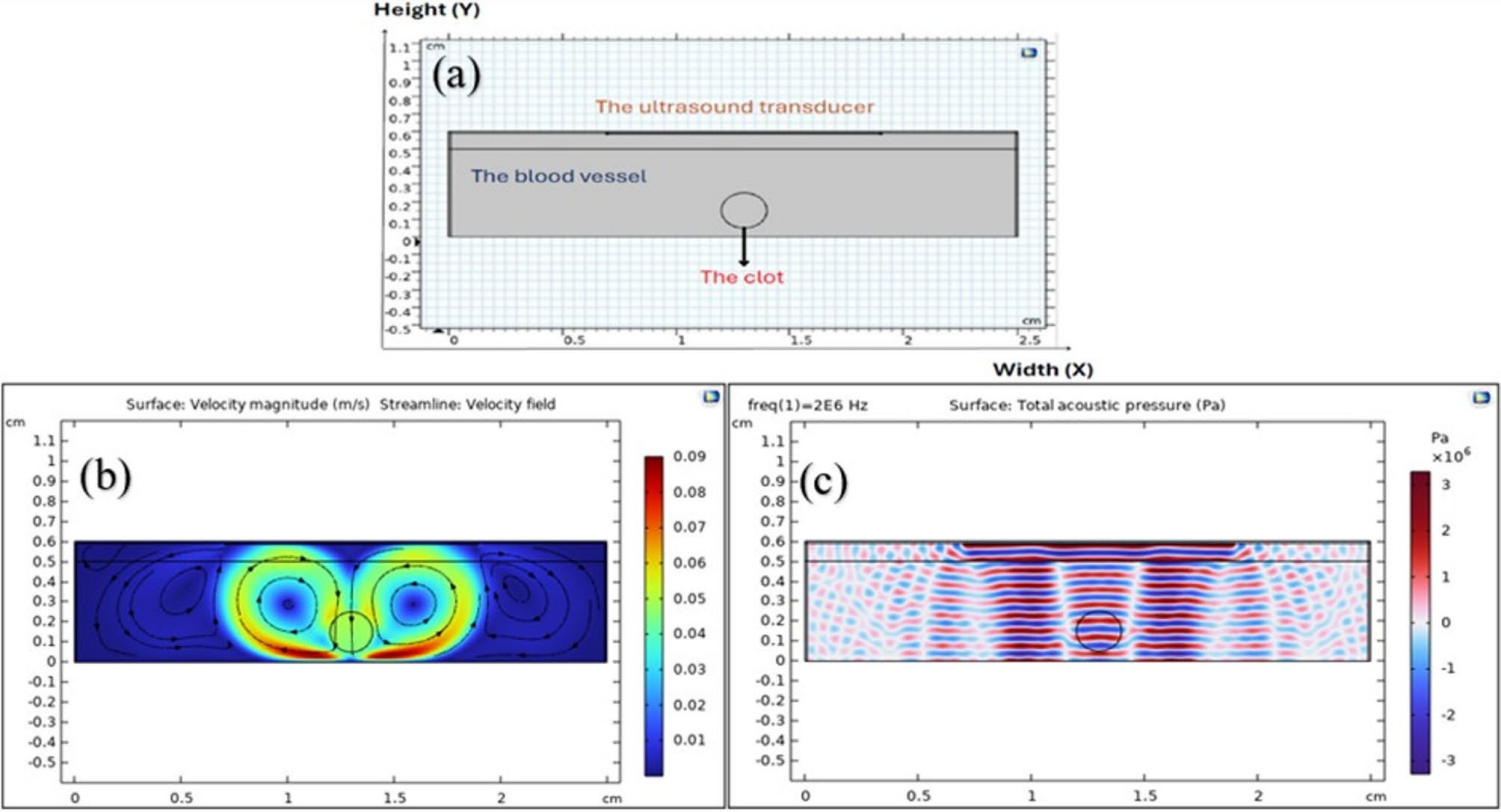
**a** The blood vessel’s thicker layer is added to the geometry model. **b** & **c** The figure illustrates the acoustic pressure and streaming velocity magnitude, after the addition of a vessel wall layer.

The model was modified to include a thicker layer for the blood vessel wall, as shown in Fig. 8. This addition aims to simulate conditions like atherosclerosis, thereby enhancing the physiological realism of the model. Through these changes, we were able to study the effect of the extra layer and the transducer’s distance from the clot on acoustic wave propagation, and the potential alteration of the shear stress applied to the clot.

This modification was implemented by modeling as an additional rectangular wall subdomain (width = 2.5 cm, height = 0.1 cm) with the same acoustic properties as the original vessel wall, representing increased wall thickness rather than a material change. In parallel, a stiffened lumen–wall interface was modeled by imposing an interior impedance boundary condition at Y = 0.5 cm, representing an increased effective acoustic impedance at that boundary and accounting for partial wave reflection and attenuation.

Initially, the shear stress at the clot surface was calculated at 2 MHz, 2 MPa after the addition of a thicker vessel above the clot in the vessel wall, resulting in a decrease in value to 2.6915 Pa. Subsequently, by studying the parameters of the sound waves that affected the acoustic streaming, applying the same frequency 2 MHz and increasing the acoustic pressure to 2.1 MPa, the shear stress at the thrombus surface was observed to increase to 2.9763 Pa, and the shear stress value increases with the increase in applied acoustic pressure.

## Discussion

In this research, we initially seek to clarify the relationship between the position of the clot and the intensity of the acoustic streaming to determine the optimal position for the ultrasound transducer to be able to generate acoustic vortices and shear stresses above the surface of the clot to facilitate its fragmentation.

Figure 4 shows a pattern of acoustic oscillations that affects the velocity of acoustic streaming as the position of the clot changes, with the oscillations decreasing and increasing proportionally^29^. Referring to Fig. 1(b), this represents the distribution of acoustic pressure on the clot within the blood vessel. When sound waves propagate and strike the boundaries of the clot, a portion of these waves is reflected. The original waves emitted by the transducer interfere with the reflected sound waves, creating the oscillation pattern shown in Fig. 4^30^. This is the standing wave theory, which is formed when two waves of the same frequency and amplitude, propagating in opposite directions, interfere. This explains the regular wave pattern, which is the regular spacing between areas of high pressure (red) and areas of low pressure (blue), which is a characteristic of standing waves. The presence of standing waves can affect a clot in several ways. The changes in acoustic pressure at the clot surface may generate shear stress forces capable of fragmenting the clot, or they may generate acoustic streaming and vortices in the fluid surrounding the clot that affect the clot’s stability and disintegration.

Determining the optimal frequency for the ultrasound transducer is the most crucial step in research to understand the effect of acoustic streaming and shear stress in the therapeutic applications of ultrasound. When frequencies decrease, characterized by their long wavelength, this enables them to penetrate tissues more deeply, thereby enhancing access to the clot and inducing effective acoustic streaming and shear stress over the clot surface, as shown in Fig. 6(a).

Conversely, when acoustic frequencies get too high, characterized by their short wavelength, these ultrasound waves are absorbed by the medium, leading to poor propagation. The attenuation coefficient rises with increasing frequency, which limits penetration depth. Consequently, the acoustic energy is predominantly converted into heat, resulting in minimal mechanical wave propagation or fluid movement, as illustrated in Fig. 6(c)^31,32^. Therefore, the results indicate that when the frequency of sound waves is reduced to 300 kHz, the penetration increases significantly, which in turn causes an increase in the velocity of the acoustic streaming, leading to the occurrence of shear stress above the surface of the thrombus. Typically, acoustic waves at lower frequencies exhibit greater penetration with less absorption. However, our findings reveal an intriguing exception at approximately 2 MHz, where an unanticipated increase in acoustic streaming velocity and penetration occurs^27^. This anomaly points towards the onset of intricate physical phenomena, such as resonance, creating an ideal frequency point that modifies the conventional relationship between frequency and streaming. This idea is of importance in therapeutic applications to maximize shear stress and acoustic streaming above the surface of the thrombus to break it up.

An ultrasound transducer operating at a frequency of 2 MHz and an acoustic pressure of 2 MPa was used in the current numerical configuration. As indicated by Fig. 1, according to simulations, in some of the experiments mentioned previously, shear stress was higher than the critical threshold of 4.1 Pa, which is enough to start thrombus disintegration^1^. Under the best possible conditions in this simulation, a maximum shear stress of 10.894 Pa was measured. Streaming flows are created at this point because of the interaction between the acoustic field and the surrounding fluid, and they are essential to the thrombolysis process. Localized areas of recirculating flow are produced by the vortices that form around the thrombus surface because of these acoustic streaming flows.

These vortices create shear stresses as they form and spread, which apply mechanical pressures to the thrombus surface that have the potential to compromise its structural stability. This mechanical engagement weakens the thrombus, making it easier for it to break down by gradually rupturing the fibrin network.

In the thick blood vessels in Fig. 8, the resulting shear stress above the clot surface is significantly reduced, making it insufficient to destroy the blood clot. However, it has been shown that increasing the acoustic pressure applied to the blood vessel leads to an increase in shear stress values, but this does not guarantee tissue safety. Figure 8 illustrates the combined effect of increased propagation path distance and the altered acoustic interaction at a stiffened wall interface on acoustic wave propagation and the resulting shear stress applied to the clot. Thus, the increased transducer–clot distance can attenuate the acoustic field reaching the clot, while the stiffened interface can further modify wave transmission and reflection; both effects are therefore expected to jointly contribute to the observed changes.

Since the lumen and clot geometry, excitation parameters, and acoustic source configuration were kept similar to Fig. 6(b), the observed differences can be attributed primarily to the increased propagation distance and impedance effects introduced by the thicker wall. As a result, the streaming vortices remain qualitatively similar, while their magnitude is affected. This research suggests that the internal layers of the body, such as the tissues and muscles surrounding the vessel, have a negative impact on the efficiency of the disintegration process. Understanding this issue requires more in-depth research to uncover its mechanisms and provide appropriate solutions to the challenge.

### Limitations & recommendations

This model has a few limitations, despite providing insightful information on how acoustic waves affect thrombus fragmentation in blood vessels. First, the blood vessel and thrombus geometry were simplified, which might have diverged from the complex nature of biological structures.

Second, it was expected that the thrombus and blood would have constant material features; nevertheless, individual differences and pathological alterations may cause these properties to change in vivo. Thirdly, the study only examined a small range of acoustic pressures and frequencies, which might not accurately reflect how the thrombus reacts to acoustic waves. Fourth, the results could be affected by the computer model’s correctness, especially in areas with intricate flow patterns. Fifth, there was no experimental validation conducted in either clinical or laboratory trials. Sixth, modeling the blood clot as a fluid with a distinct viscosity and assuming rigid, non-deforming vessel walls represent simplifications that limit the physiological realism of the model. Treating the clot as a fluid ignores its complex mechanical properties, such as its viscoelastic or porous nature, thereby preventing the modeling of its deformation or fracture and disregarding internal fluid-structure interactions within it. Similarly, assuming rigid vessel walls disregards the natural elasticity of blood vessel walls, precluding the modeling of fluid-structure interaction (FSI) and the impact of wall deformation on flow dynamics and clot behavior, which ultimately reduces the overall physiological realism of the model. Seventh, we modeled the blood velocity as zero. Eighth, the assumption of laminar flow in the presence of a clot ignores the complex dynamics of blood flow, which may transition from laminar to turbulent and potentially impede the acoustic streaming affecting the clot. Ninth, the transducer was placed inside the blood vessel to study the direct effect of sound waves on the clot, which does not represent a clinical application.

For future work, we plan to use more accurate models for the thrombus and the blood vessel wall, considering differences in material properties, thermal effects, and moving from simplified models (a fluid clot and rigid vessel walls) to more complex, viscoelastic models for the blood clot and elastic models for the vessel walls. We will also develop a more realistic model to simulate the effects of sound waves reflected from the vessel wall, along with other factors, and their subsequent impact on acoustic streaming around the clot. We also plan to expand the frequency and acoustic pressure ranges and develop a 3D simulation model to be more accurate and realistic. We will model non-zero blood velocity to observe its effect on acoustic microstreaming around the clot, and addressing turbulent flow will be a crucial aspect of our future work to enhance the physiological realism and predictive capability of our models. The model will also be modified to include the placement of the transducer outside the body and applying (PML) to the outer borders.

## Supplementary Information

Below is the link to the electronic supplementary material.

Supplementary Material 1

Supplementary Material 2

Supplementary Material 3

Supplementary Material 4

## Data Availability

The datasets used and analyzed during the current study are available from the corresponding author upon reasonable request.

## Code availability

The COMSOL Multiphysics simulation files and mathematical algorithms used in this study are publicly available without restrictions in the GitHub repository at: [https://github.com/aishahishamsaid/acoustic-streaming-COMSOL-modeling]. To ensure the version of the code described in this publication is maintained and citable, it has been deposited in a DOI version control repository accessible via: [10.5281/zenodo.16932468].
