# Supplementary material for: Numerical investigation of ultrasound-induced acoustic streaming and shear stress for blood clot manipulation
Source: Sci Rep. 2026 Apr 20;16:12891. doi: 10.1038/s41598-026-44521-5 (PMC13096658; doi:10.1038/s41598-026-44521-5)
Supplement: Supplementary file 1 — Supplementary Material 1 [file 41598_2026_44521_MOESM1_ESM.pdf]

1. **Effect of Frequency Variation:**

- By fixing the Acoustic Pressure at 320kPa and the thrombus location X=1.3 and altering the Frequency, the following results were observed :

- A decrease in streaming velocity with increasing frequency from 300kHz to 1MHz, and irregular behavior from 1MHz to 15MHz. Considering that at a value of 2 MHz, there is an increase in streaming, which is a suitable value in therapeutic applications.

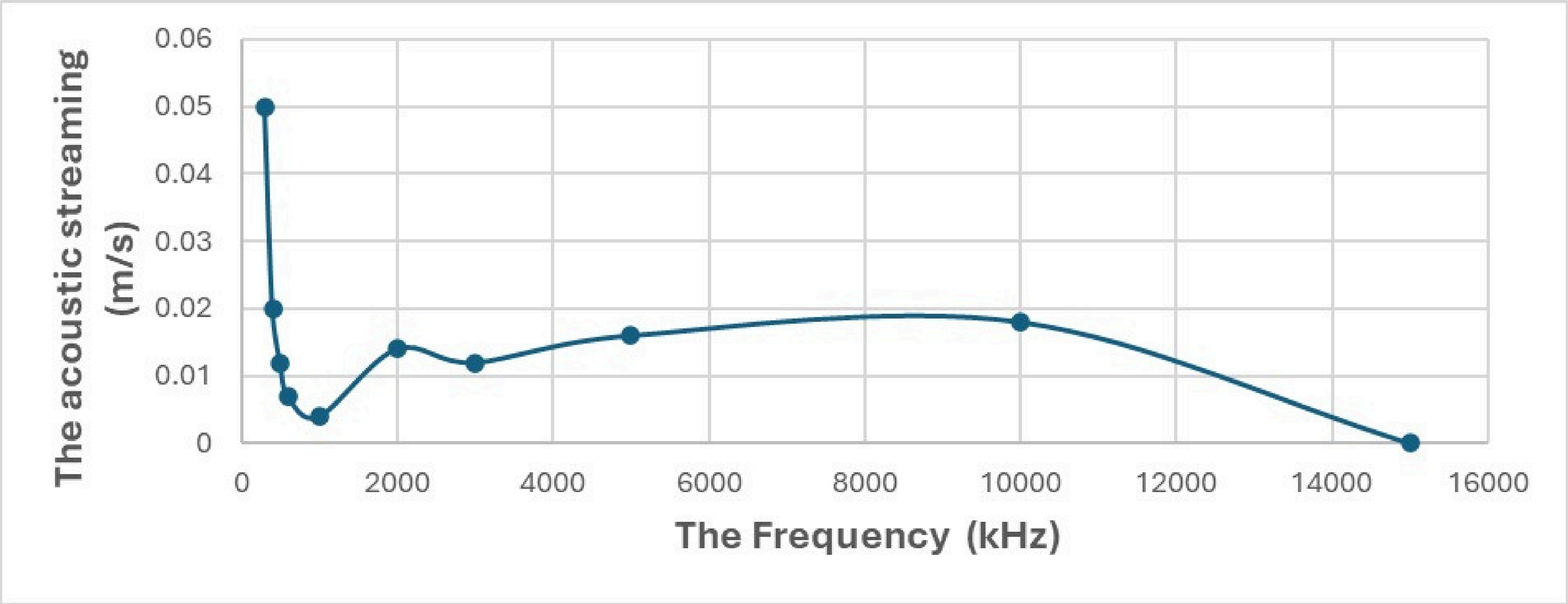

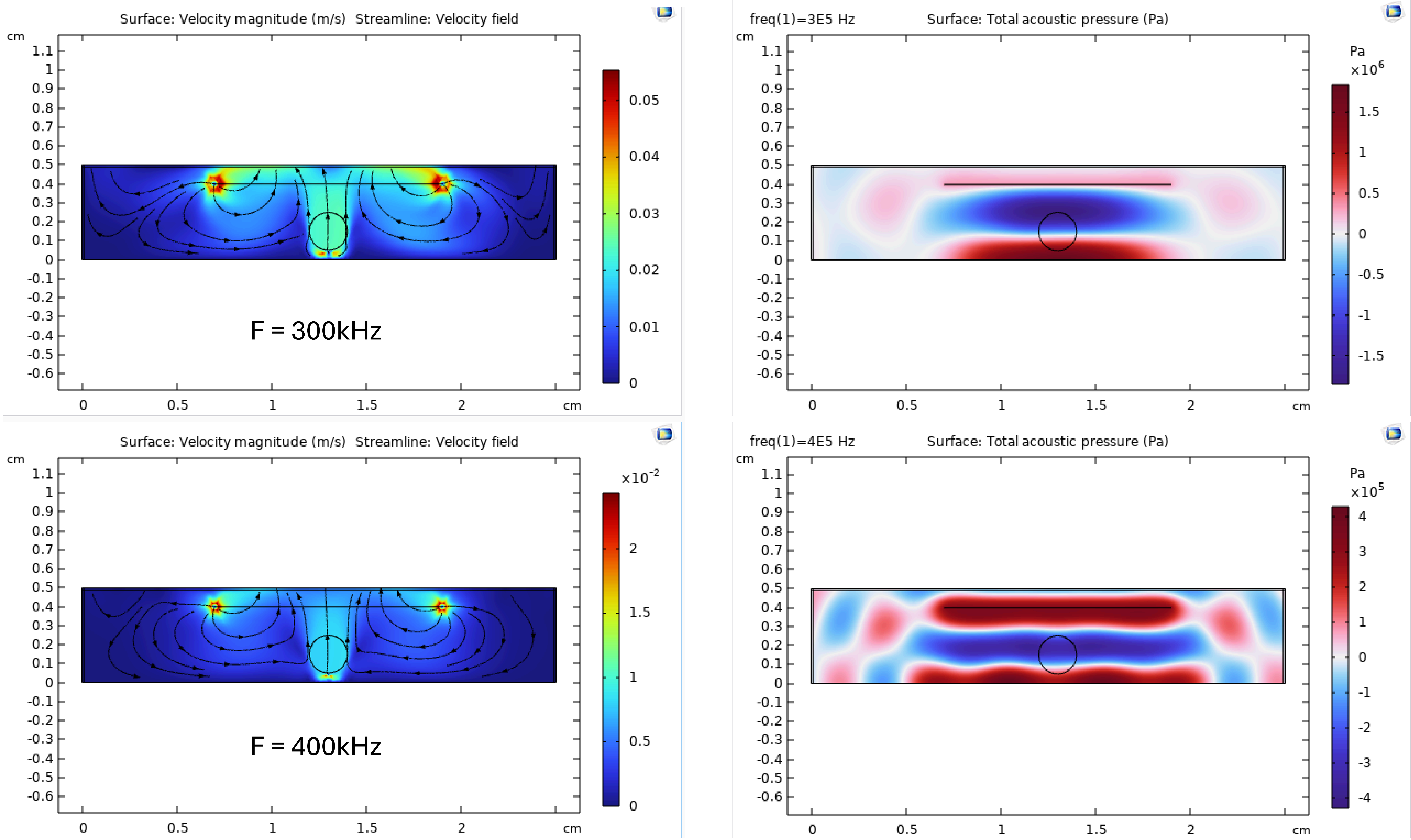

Surface: Velocity magnitude (m/s) Streamline: Velocity field
cm
F = 300kHz
freq(1)=3E5 Hz
Surface: Total acoustic pressure (Pa)
Pa
×10^6
Surface: Velocity magnitude (m/s) Streamline: Velocity field
×10^-2
F = 400kHz
freq(1)=4E5 Hz
Surface: Total acoustic pressure (Pa)
Pa
×10^5

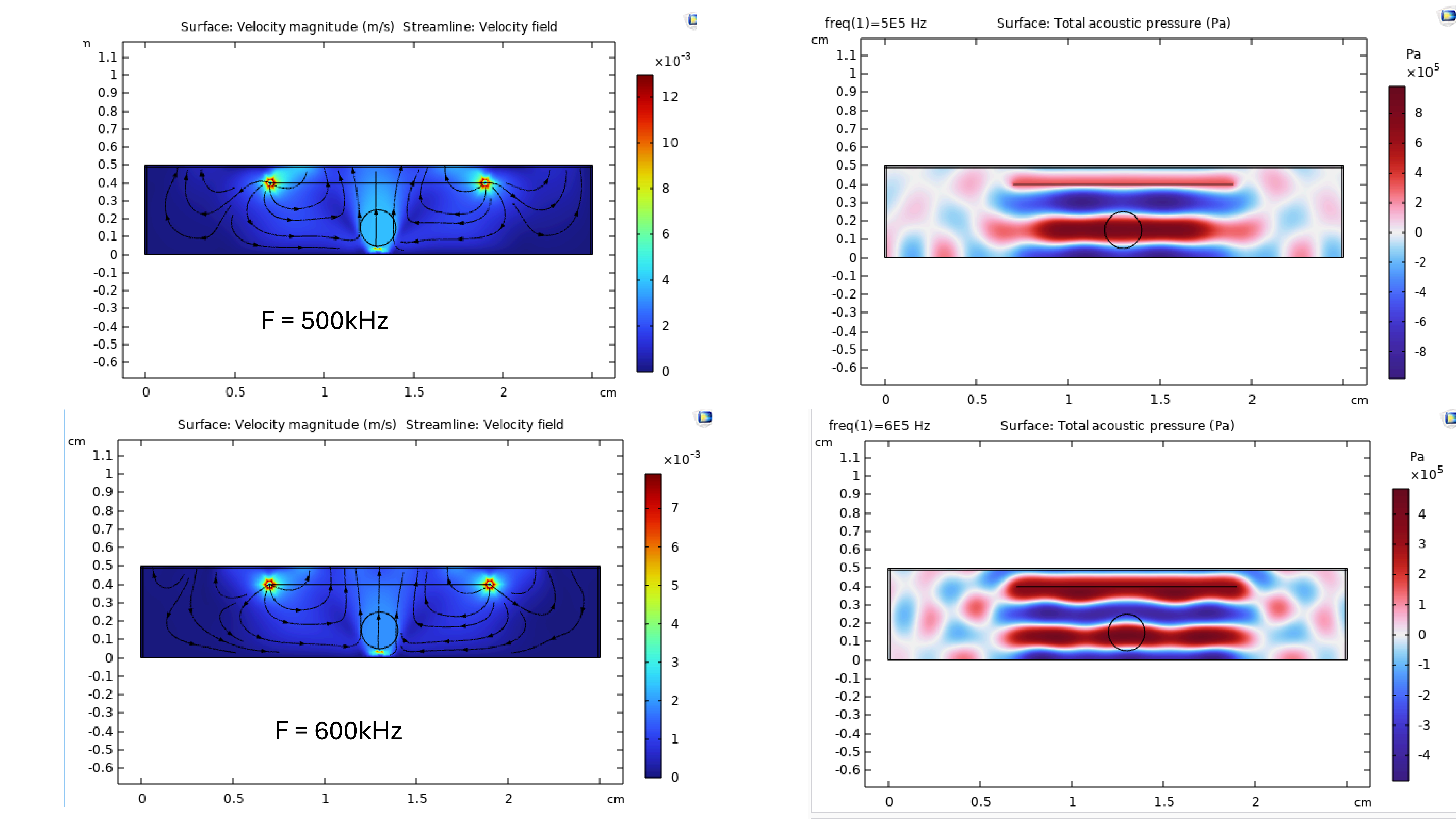
Surface: Velocity magnitude (m/s) Streamline: Velocity field
×10^-3
F = 500kHz
cm
freq(1)=5E5 Hz
Surface: Total acoustic pressure (Pa)
Pa
×10^5
cm
Surface: Velocity magnitude (m/s) Streamline: Velocity field
×10^-3
F = 600kHz
cm
freq(1)=6E5 Hz
Surface: Total acoustic pressure (Pa)
Pa
×10^5
cm

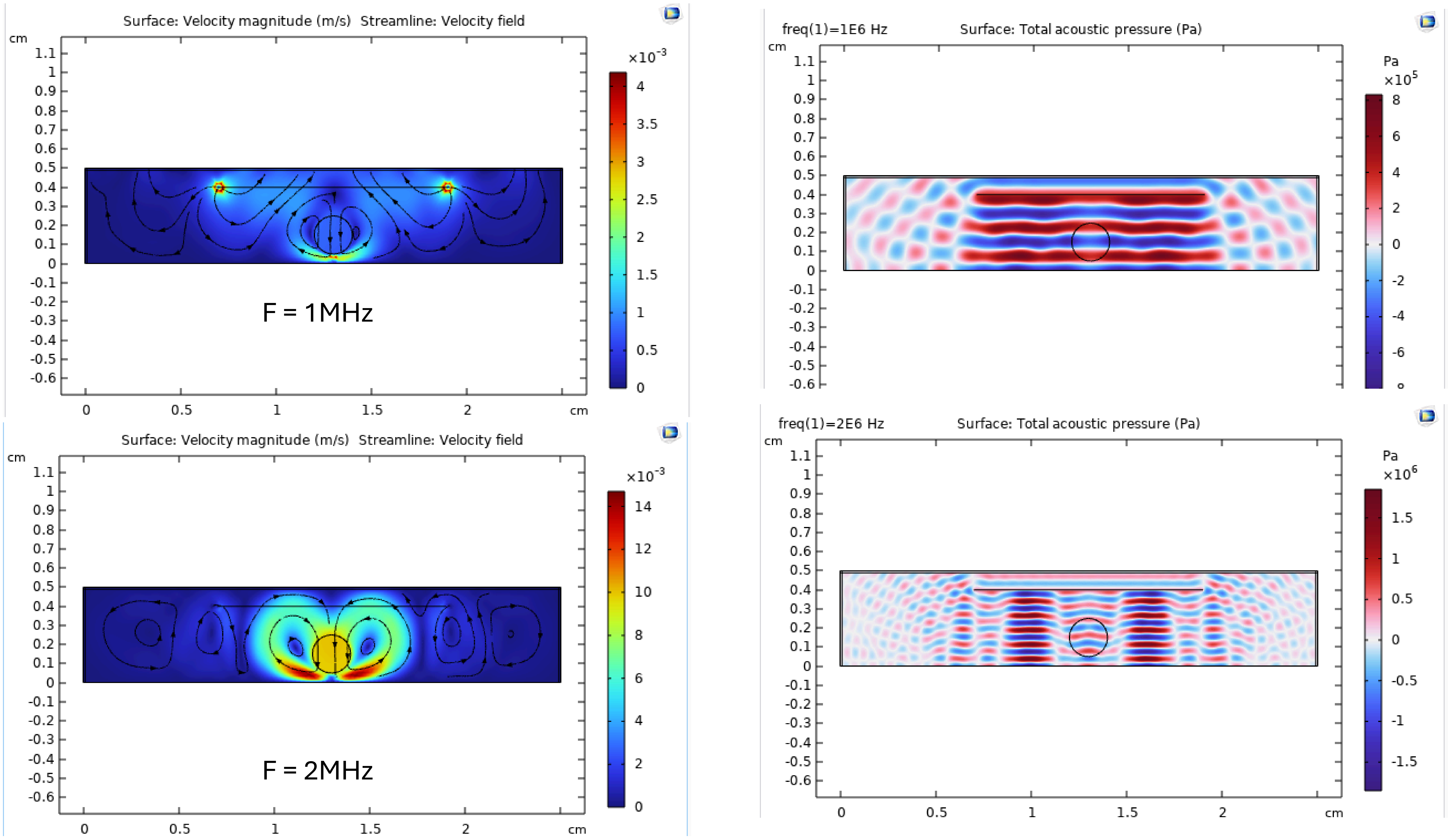

Surface: Velocity magnitude (m/s) Streamline: Velocity field
F = 1MHz
freq(1)=1E6 Hz
Surface: Total acoustic pressure (Pa)
Surface: Velocity magnitude (m/s) Streamline: Velocity field
F = 2MHz
freq(1)=2E6 Hz
Surface: Total acoustic pressure (Pa)

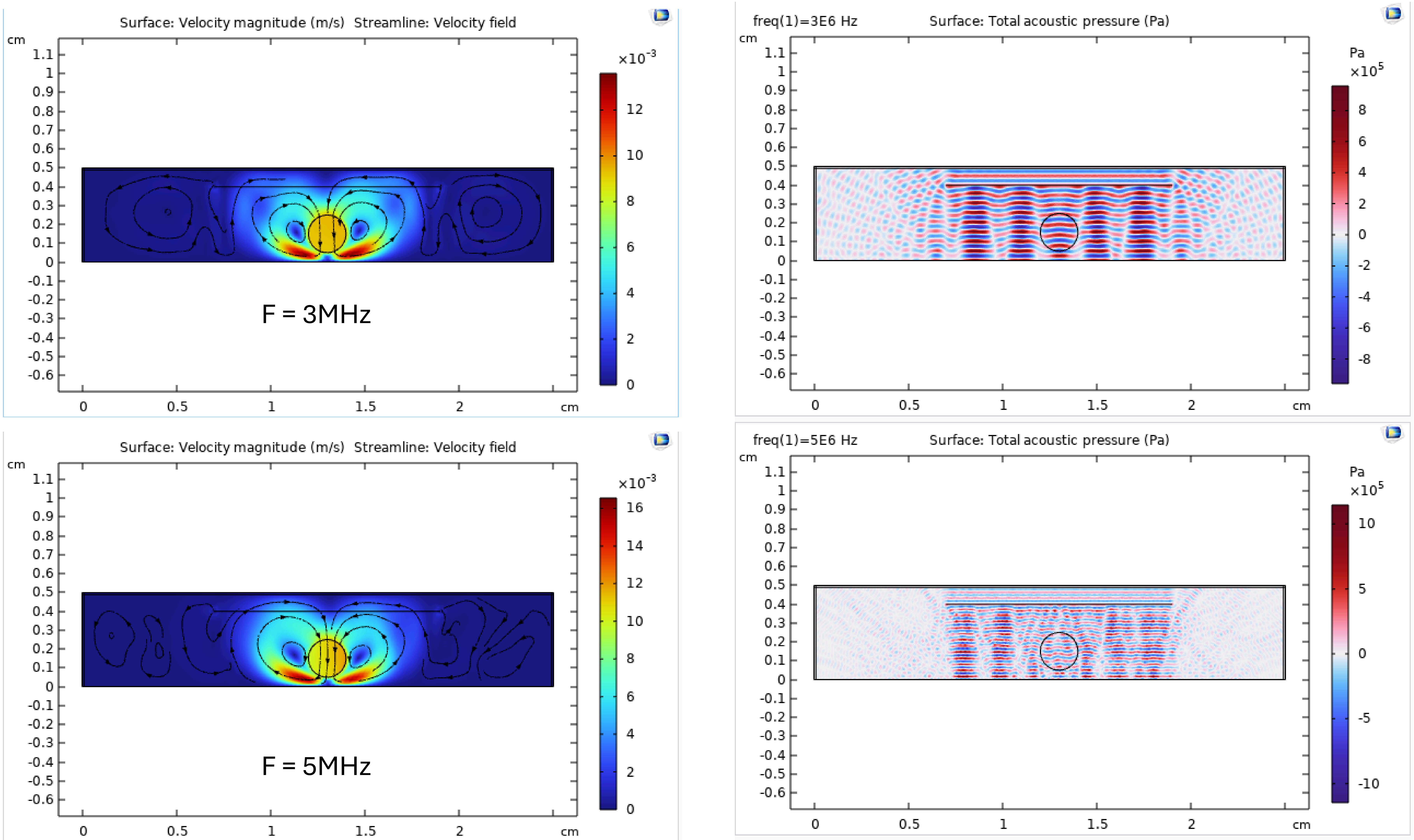
Surface: Velocity magnitude (m/s) Streamline: Velocity field
cm
×10^-3
F = 3MHz
freq(1)=3E6 Hz
Surface: Total acoustic pressure (Pa)
Pa
×10^5
Surface: Velocity magnitude (m/s) Streamline: Velocity field
F = 5MHz
freq(1)=5E6 Hz
Surface: Total acoustic pressure (Pa)

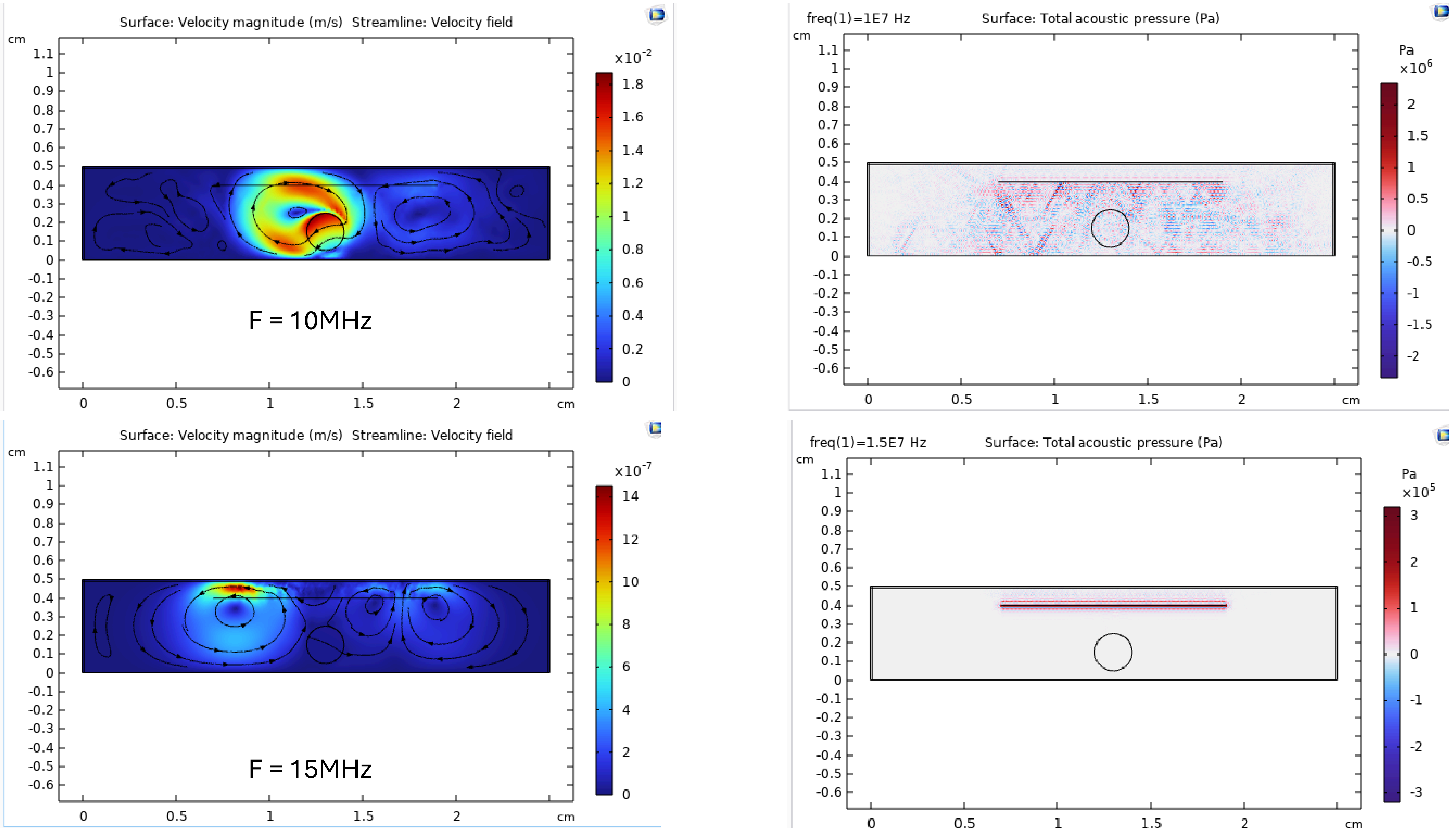
Surface: Velocity magnitude (m/s) Streamline: Velocity field
cm
×10^-2
F = 10MHz
freq(1)=1E7 Hz
Surface: Total acoustic pressure (Pa)
Pa
×10^6
Surface: Velocity magnitude (m/s) Streamline: Velocity field
×10^-7
F = 15MHz
freq(1)=1.5E7 Hz
Surface: Total acoustic pressure (Pa)
Pa
×10^5

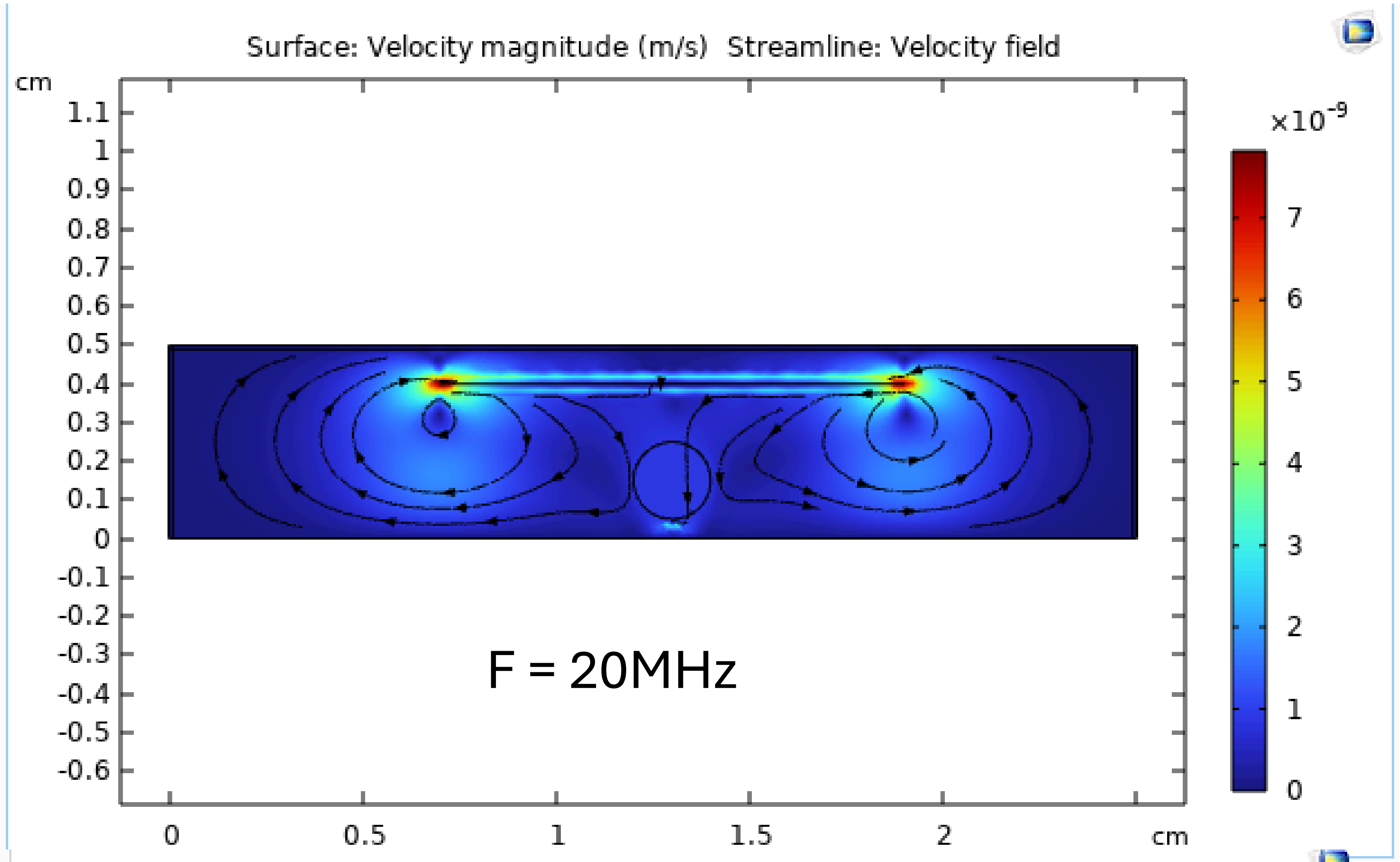
Surface: Velocity magnitude (m/s) Streamline: Velocity field
cm
×10^-9
F = 20MHz

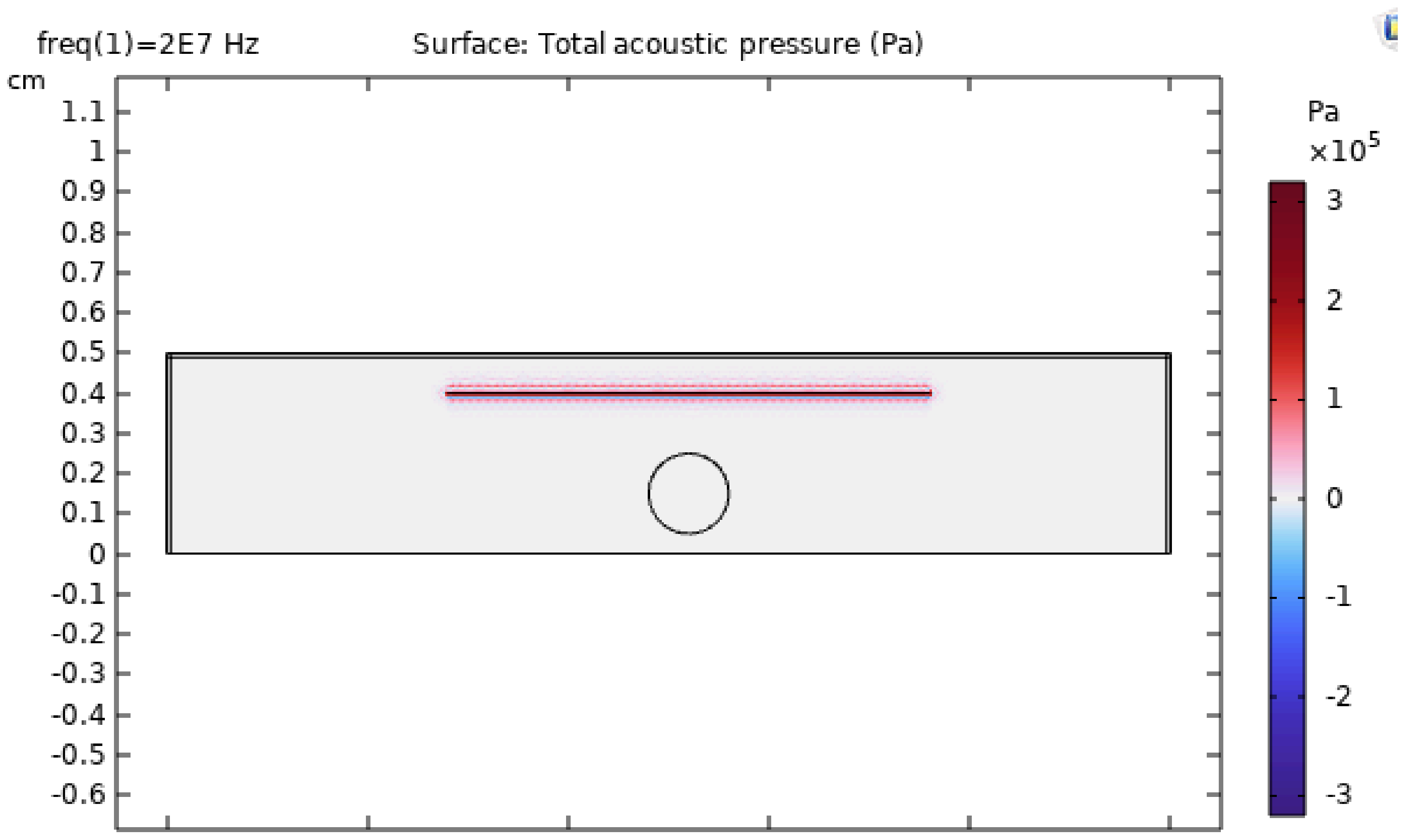
freq(1)=2E7 Hz
Surface: Total acoustic pressure (Pa)
cm
Pa
×10^5

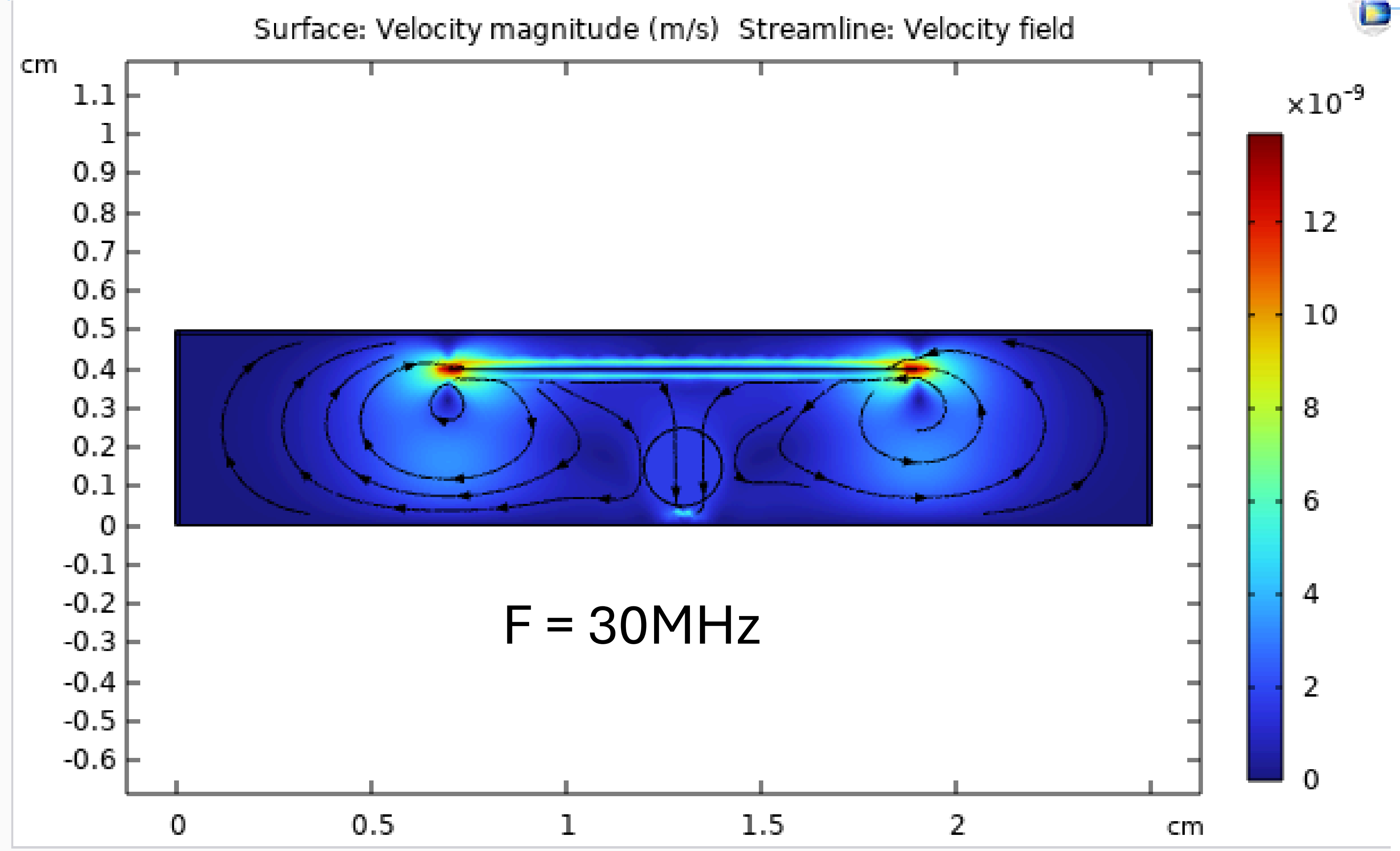
Surface: Velocity magnitude (m/s) Streamline: Velocity field
cm
×10^-9
F = 30MHz

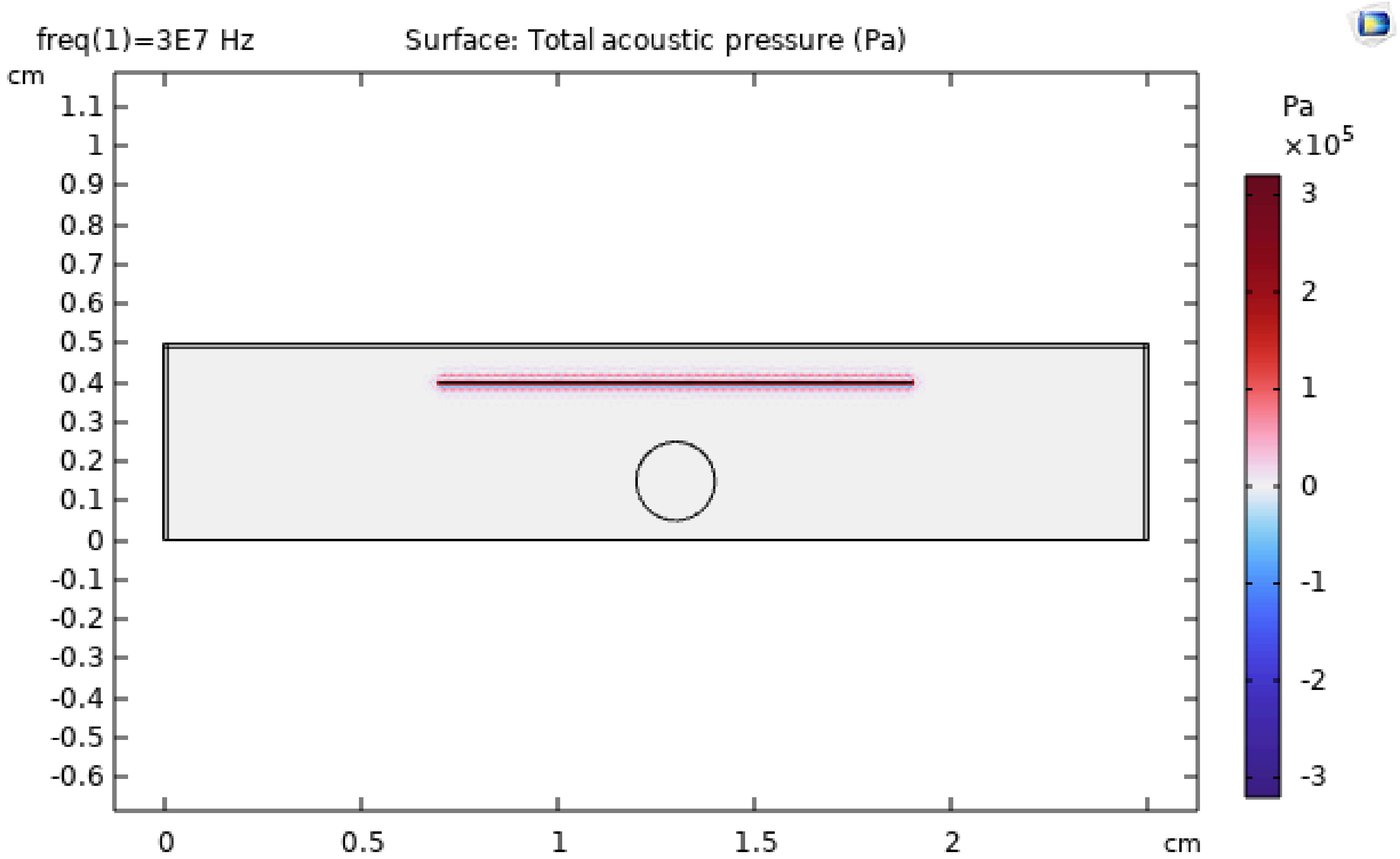
freq(1)=3E7 Hz
Surface: Total acoustic pressure (Pa)
cm
Pa
×10^5
