# Supplementary material for: Numerical investigation of ultrasound-induced acoustic streaming and shear stress for blood clot manipulation
Source: Sci Rep. 2026 Apr 20;16:12891. doi: 10.1038/s41598-026-44521-5 (PMC13096658; doi:10.1038/s41598-026-44521-5)
Supplement: Supplementary file 3 — Supplementary Material 3 [file 41598_2026_44521_MOESM3_ESM.pptx]

## Slide 1
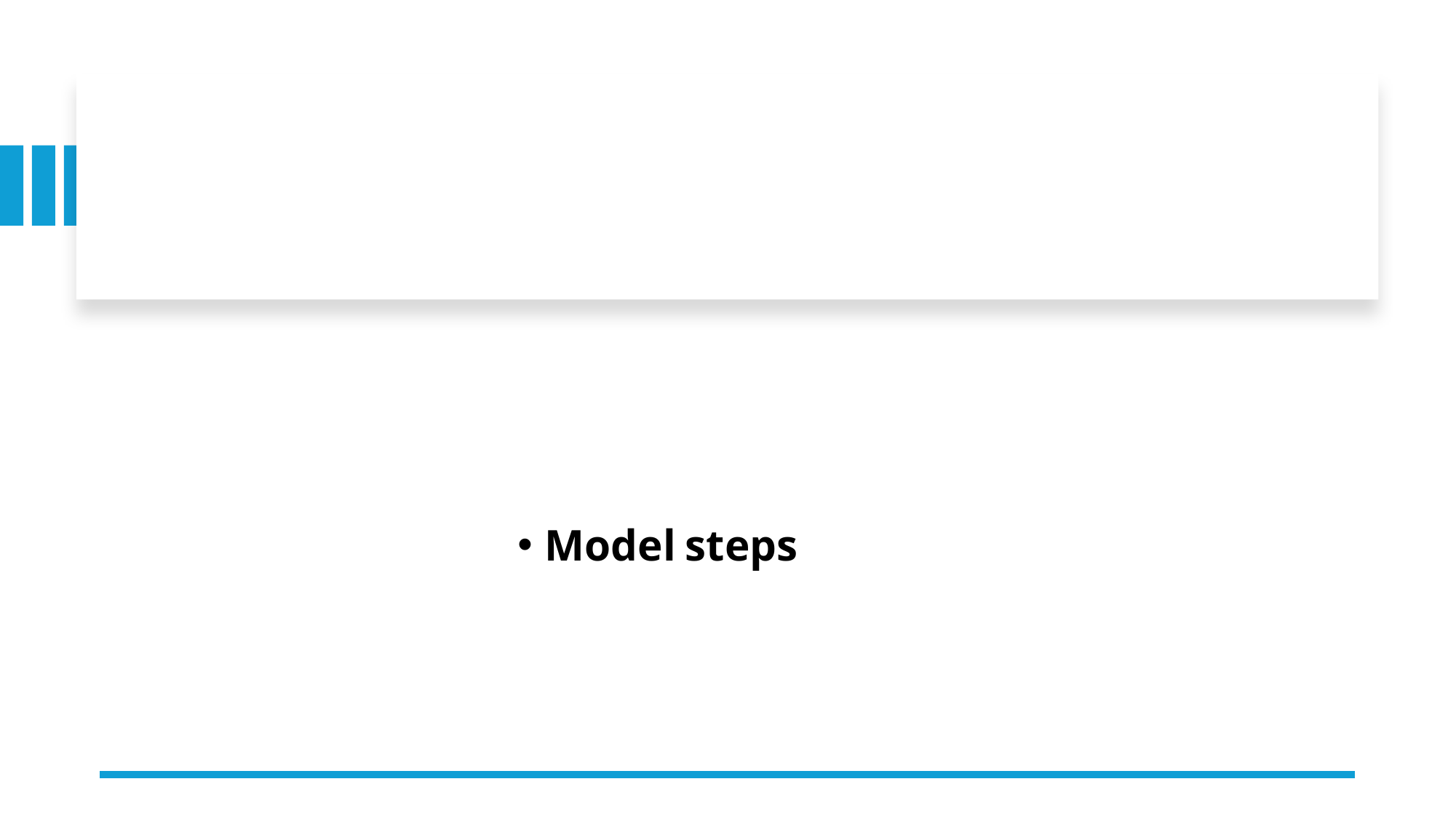

Model steps

## Slide 2
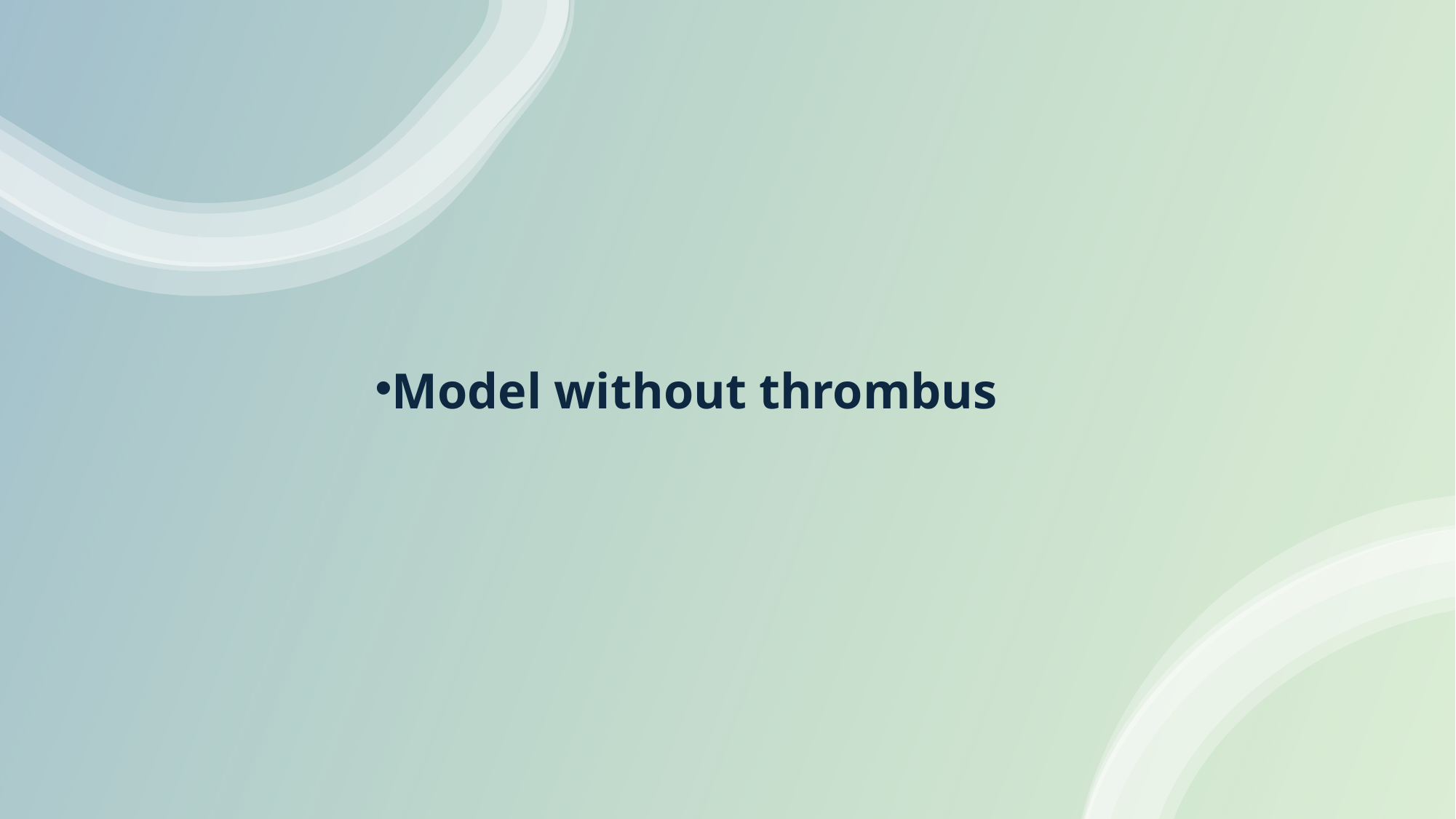

Model without thrombus

## Slide 3
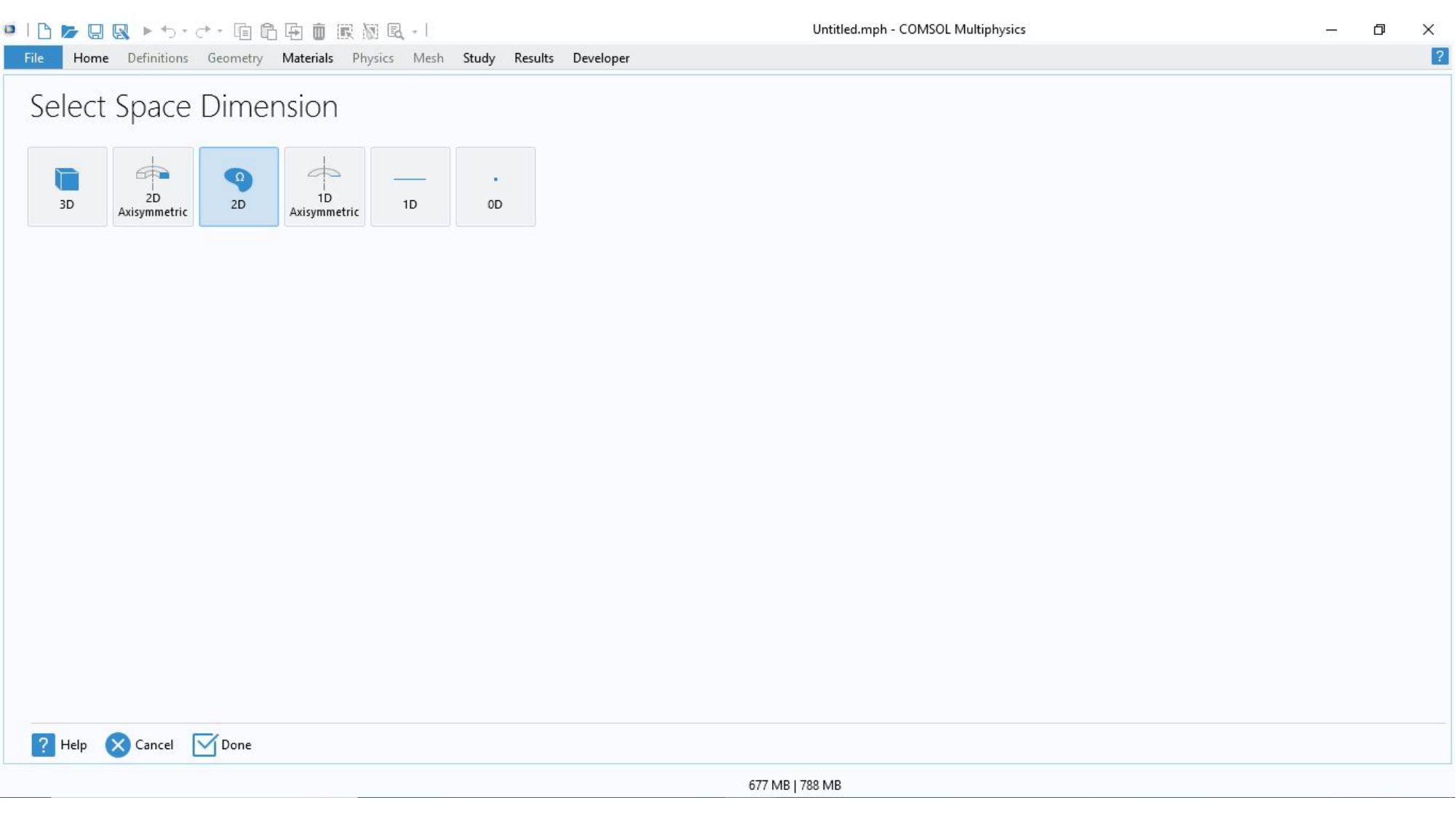

## Slide 4
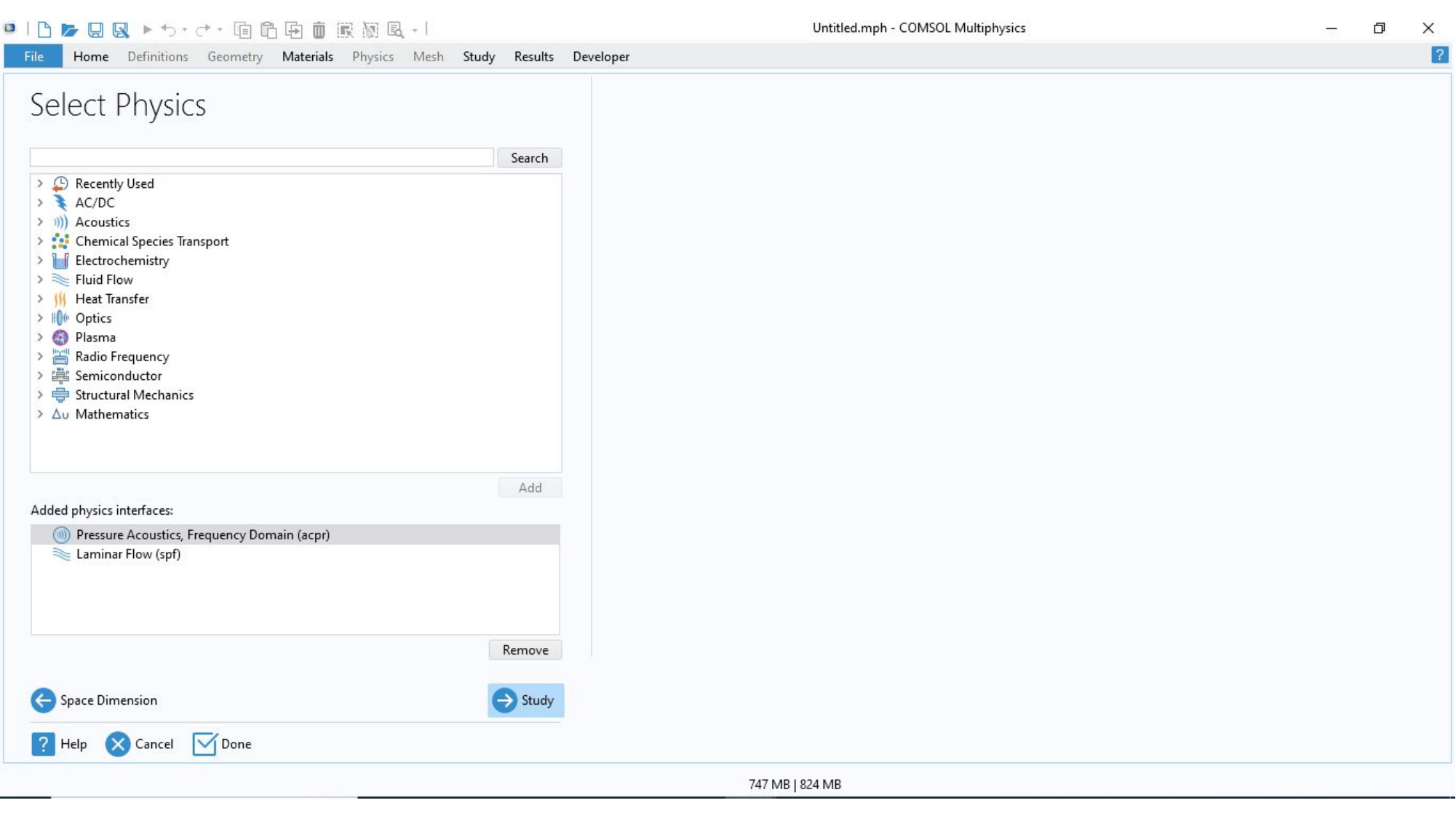

## Slide 5
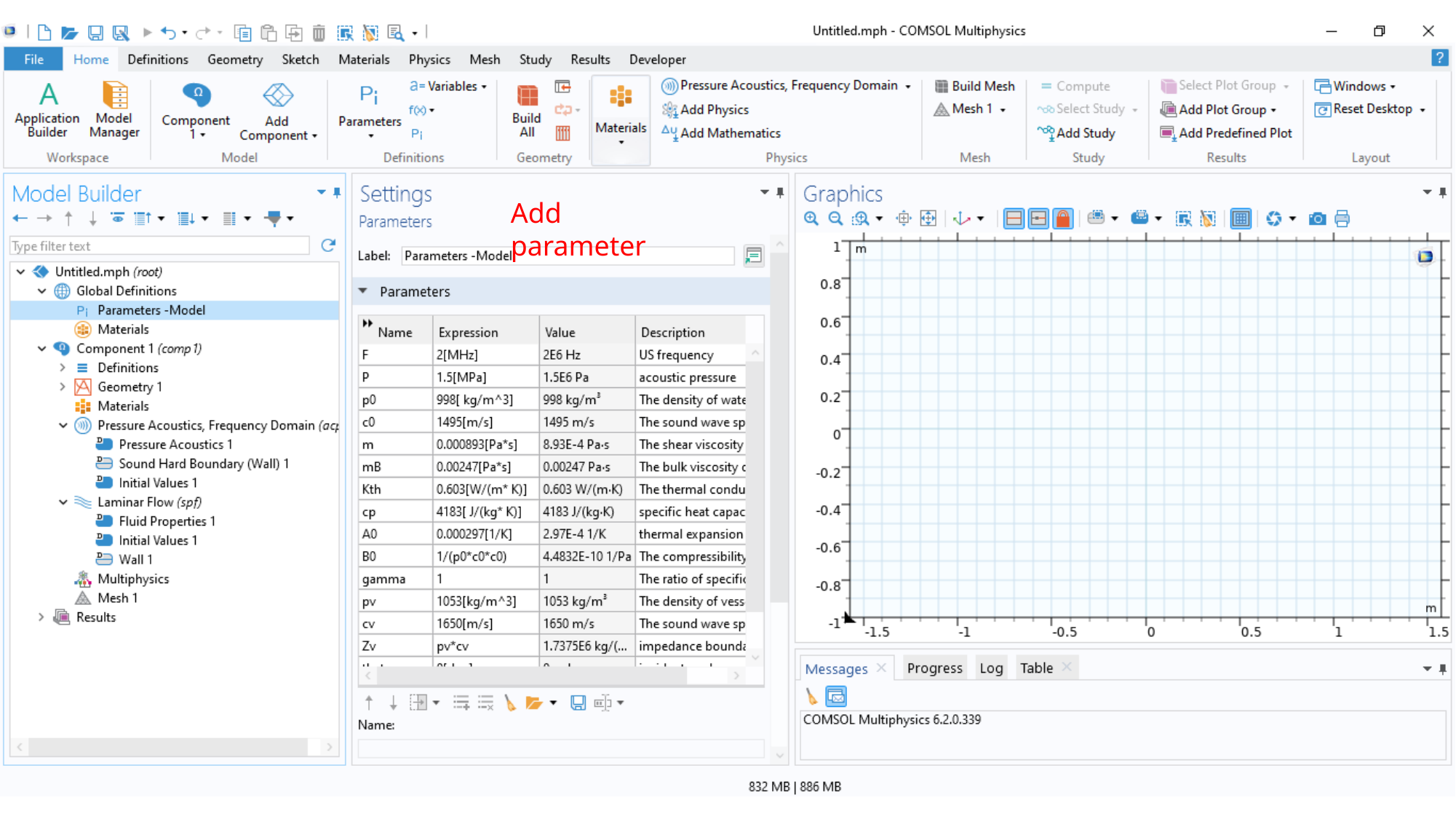

Add parameter

## Slide 6
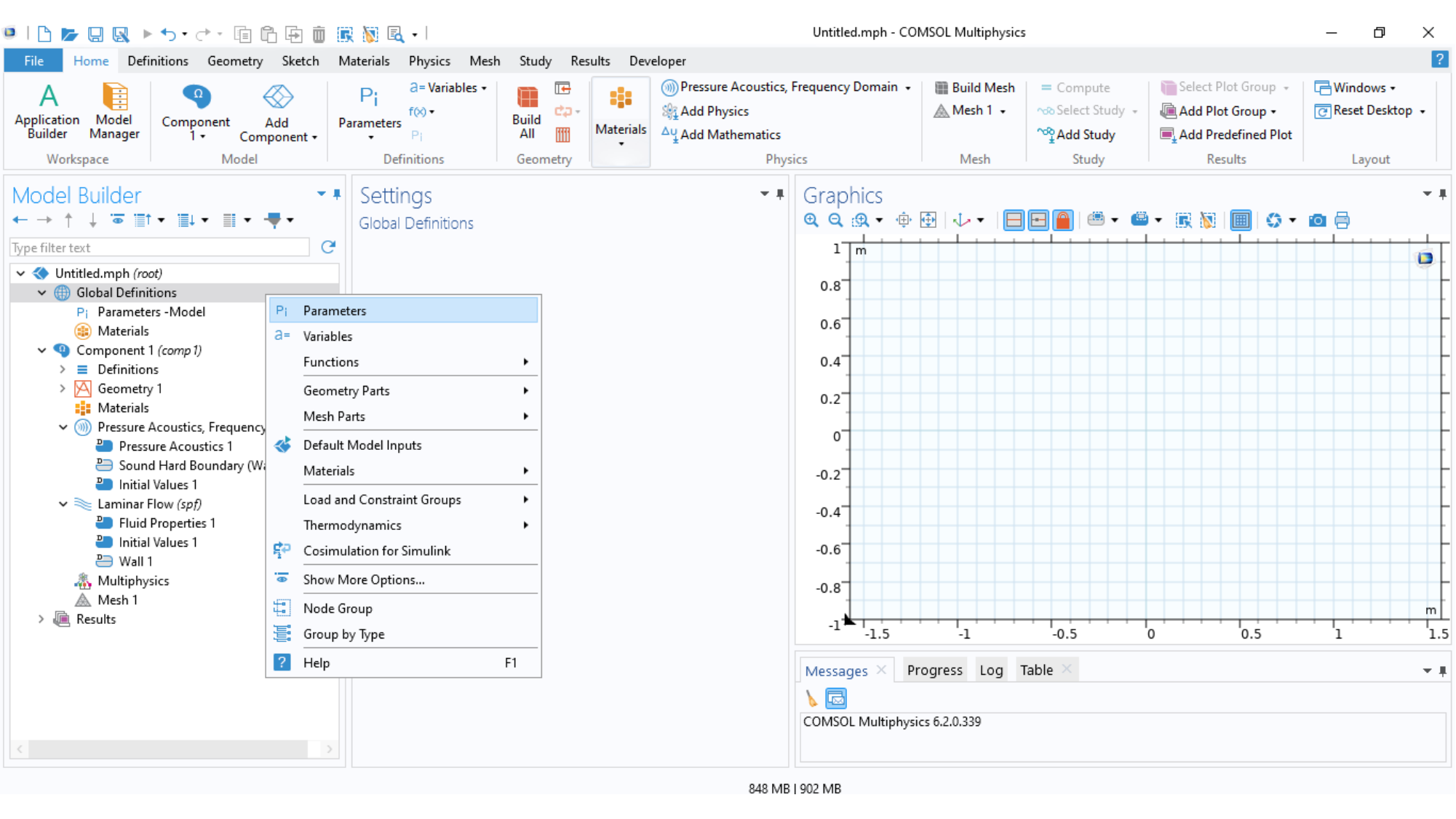

## Slide 7
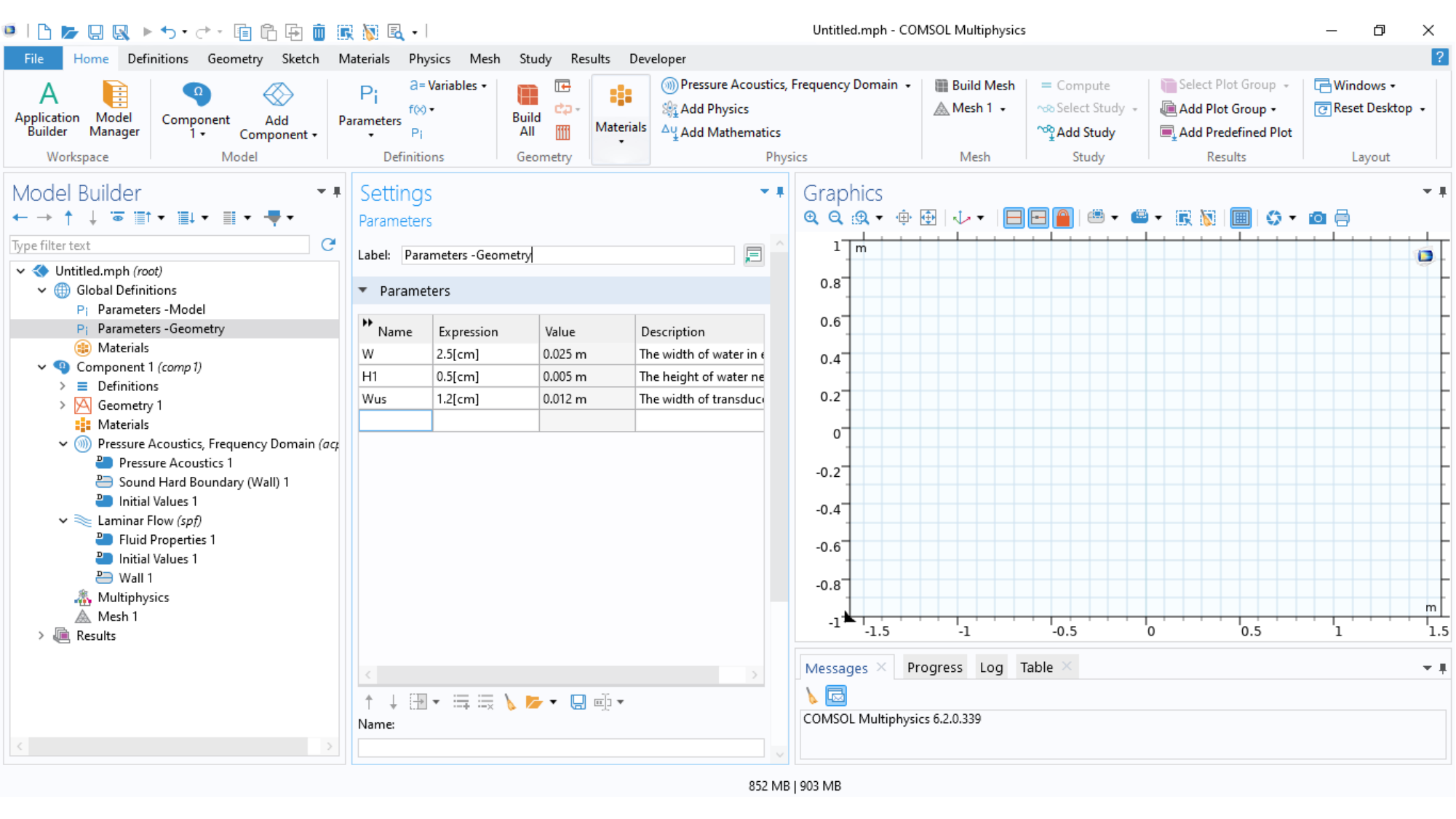

## Slide 8
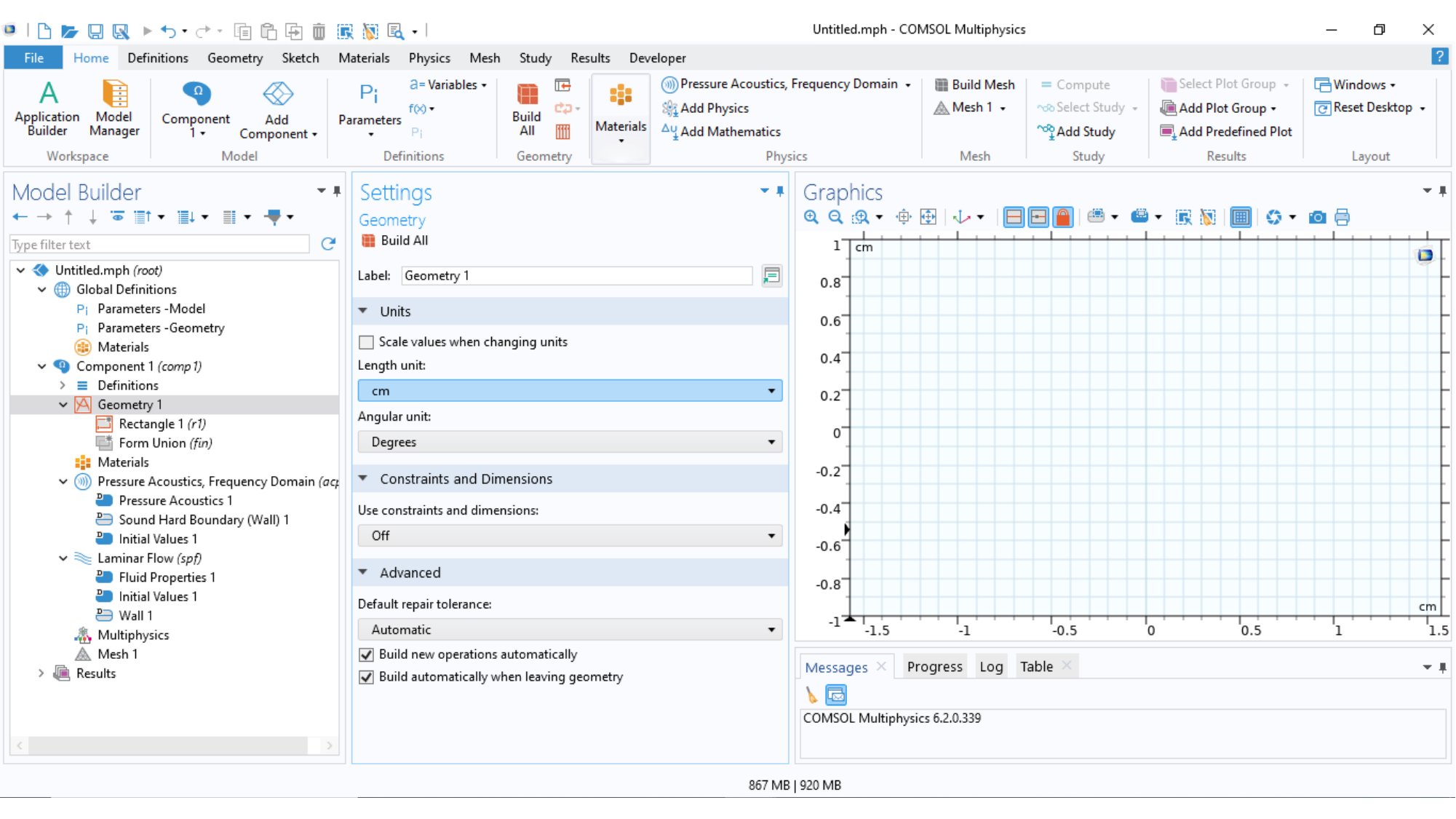

## Slide 9
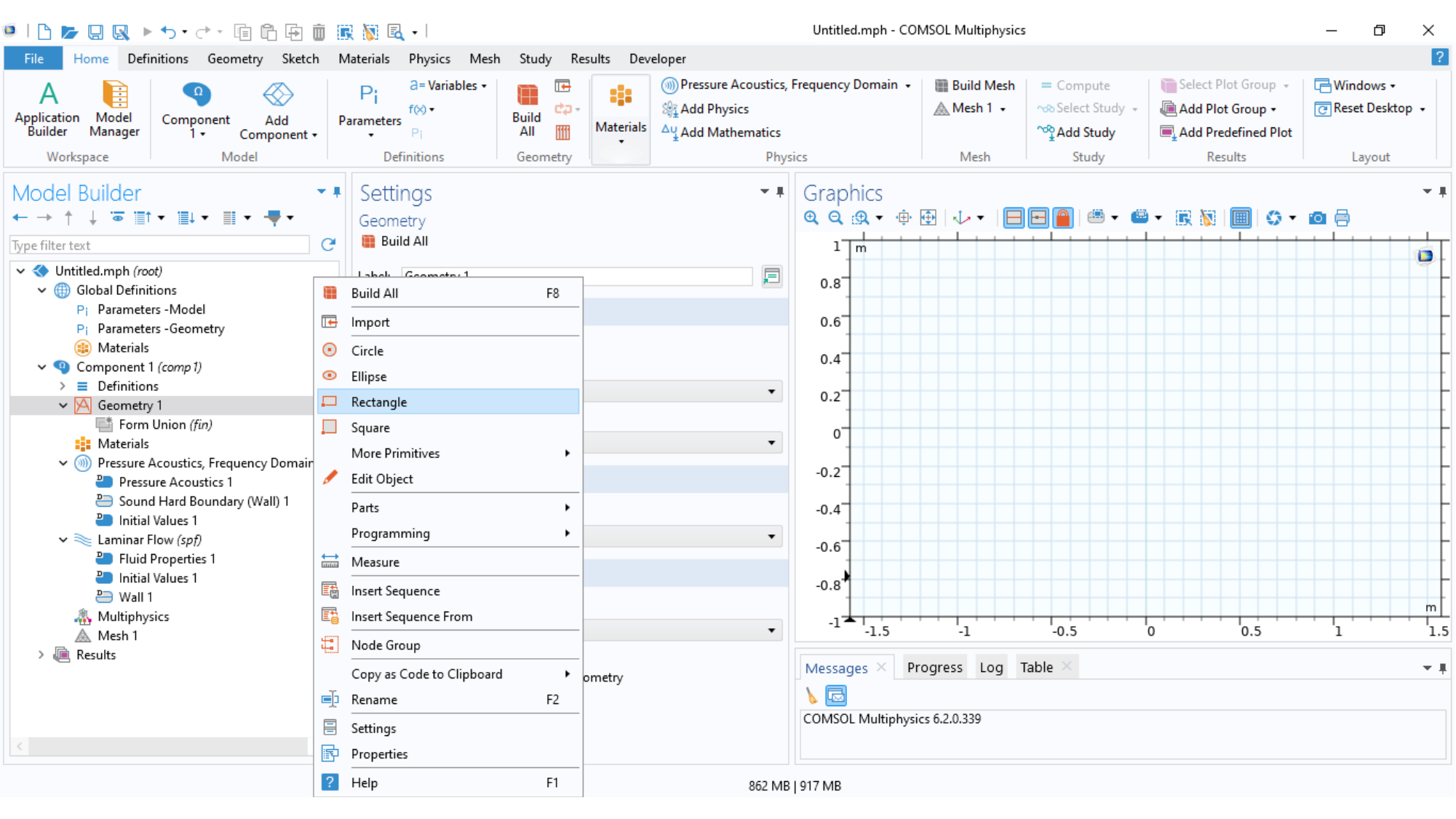

## Slide 10
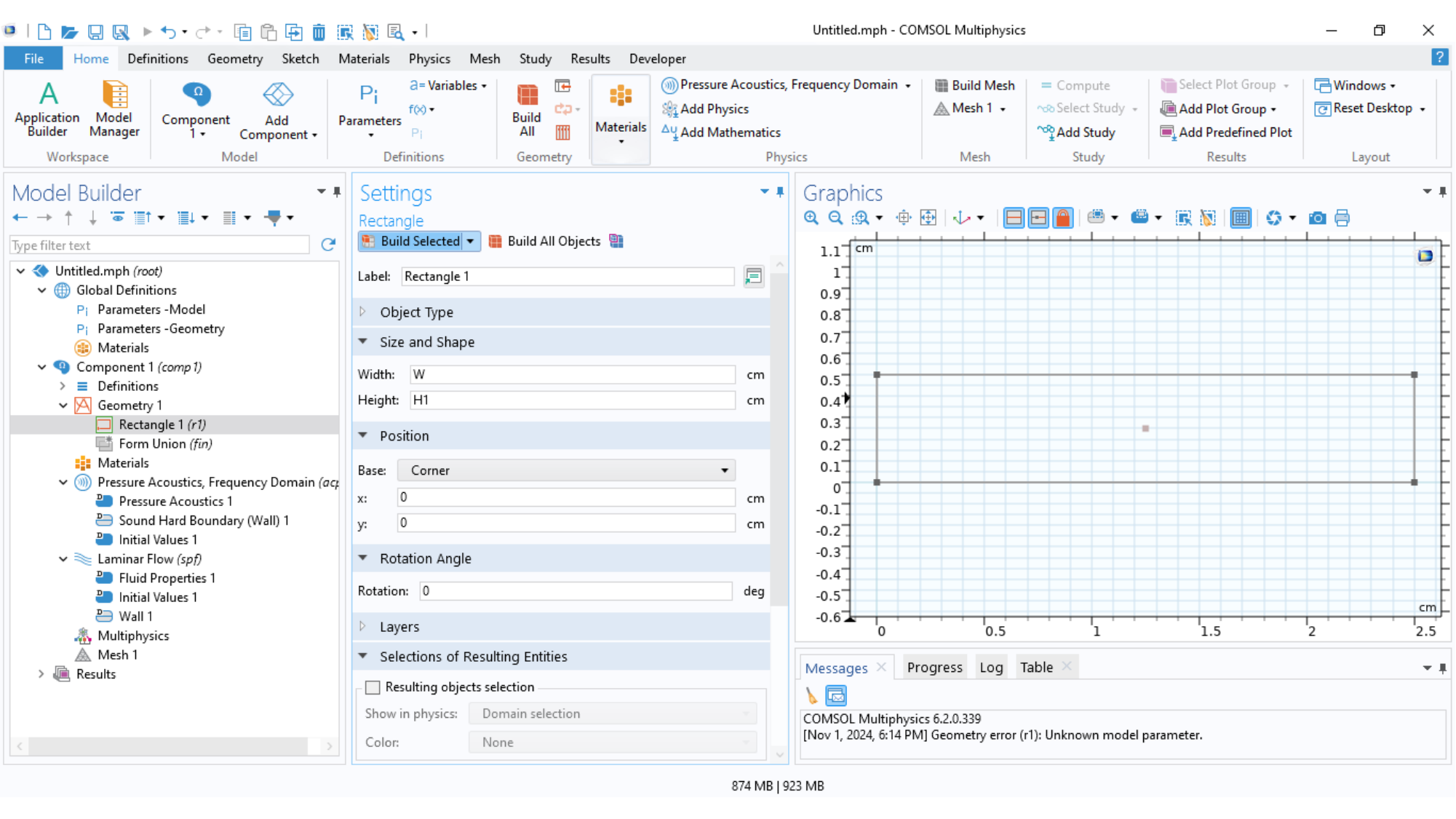

## Slide 11
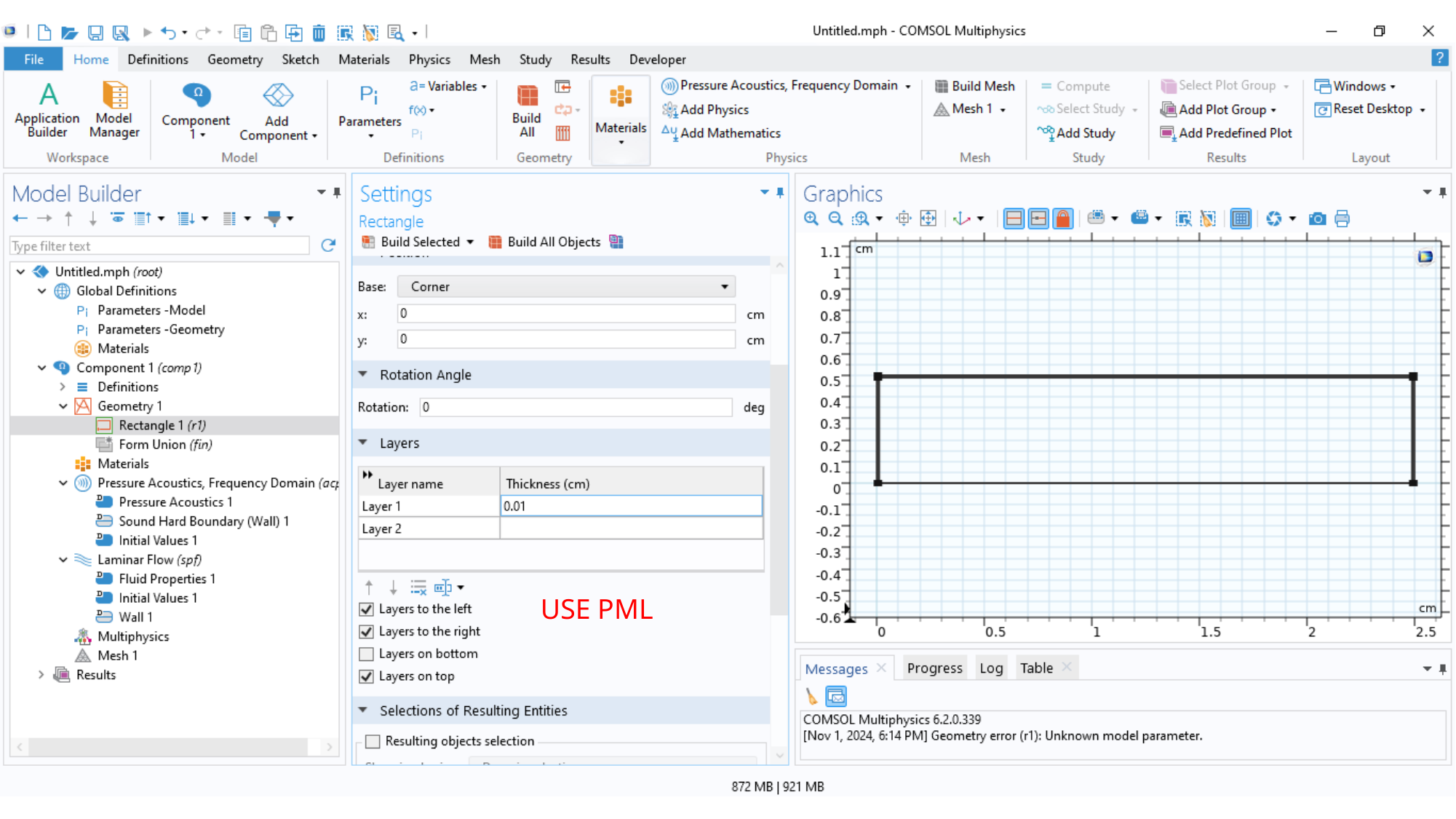

USE PML

## Slide 12
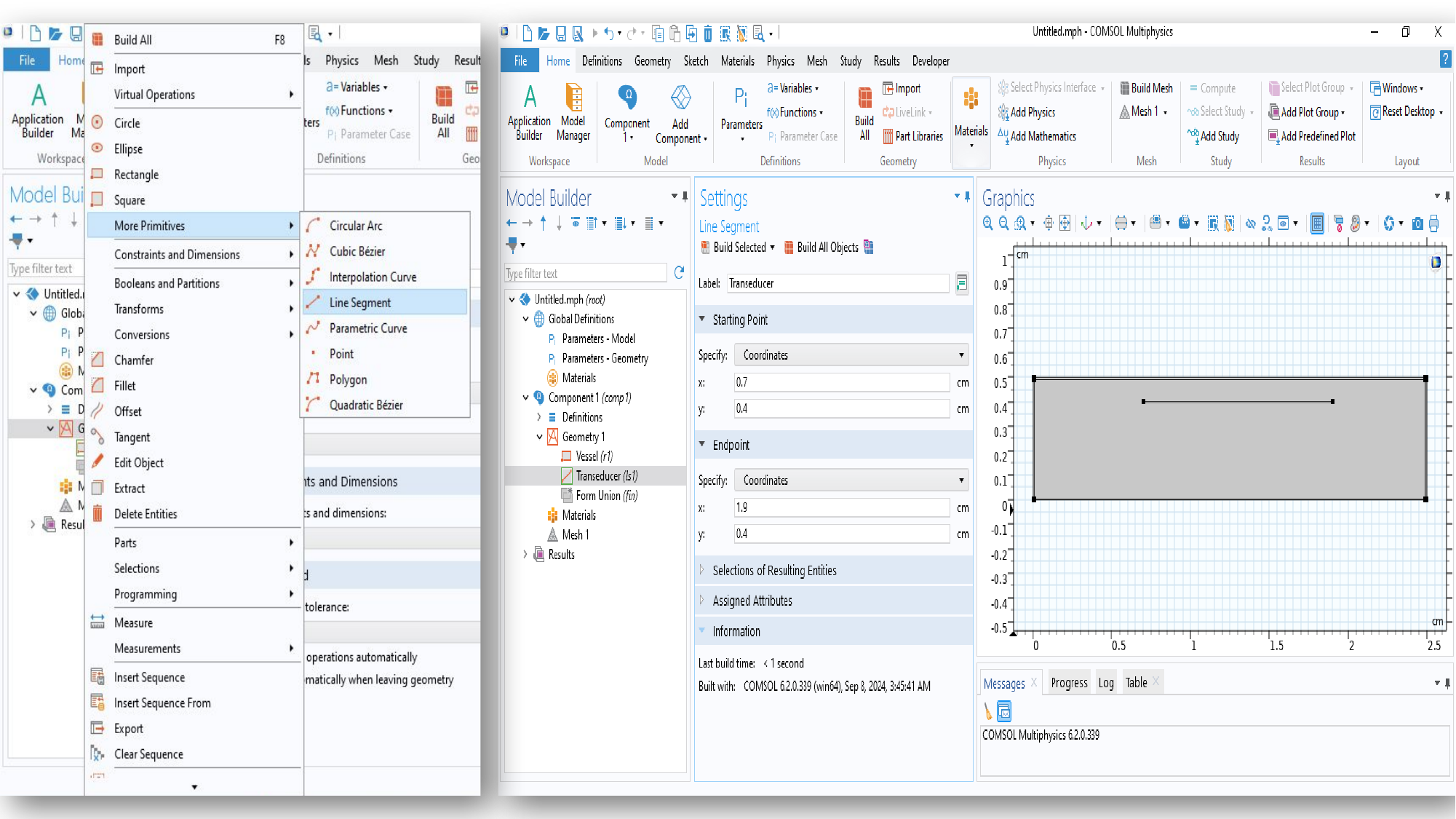

## Slide 13
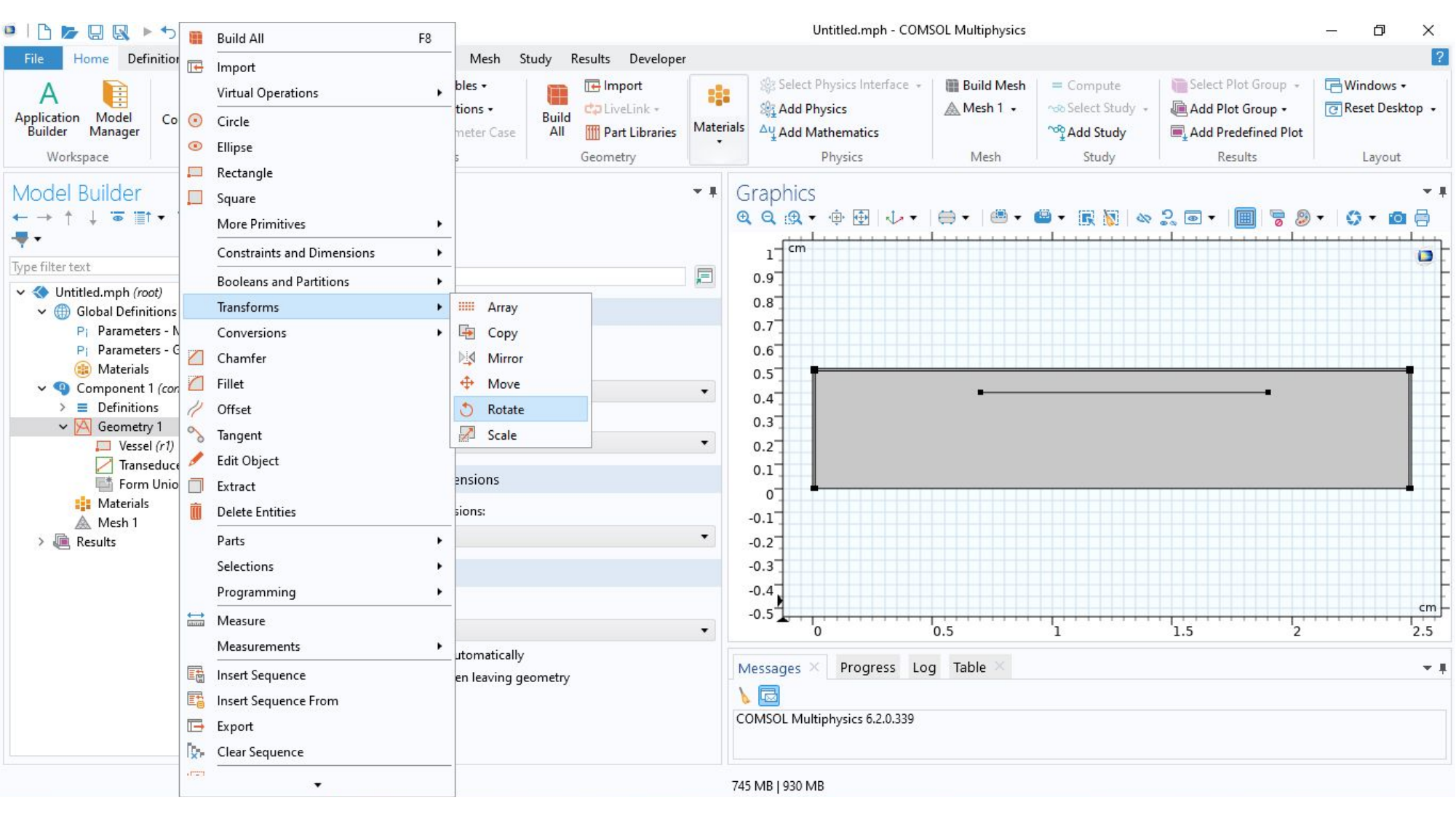

## Slide 14
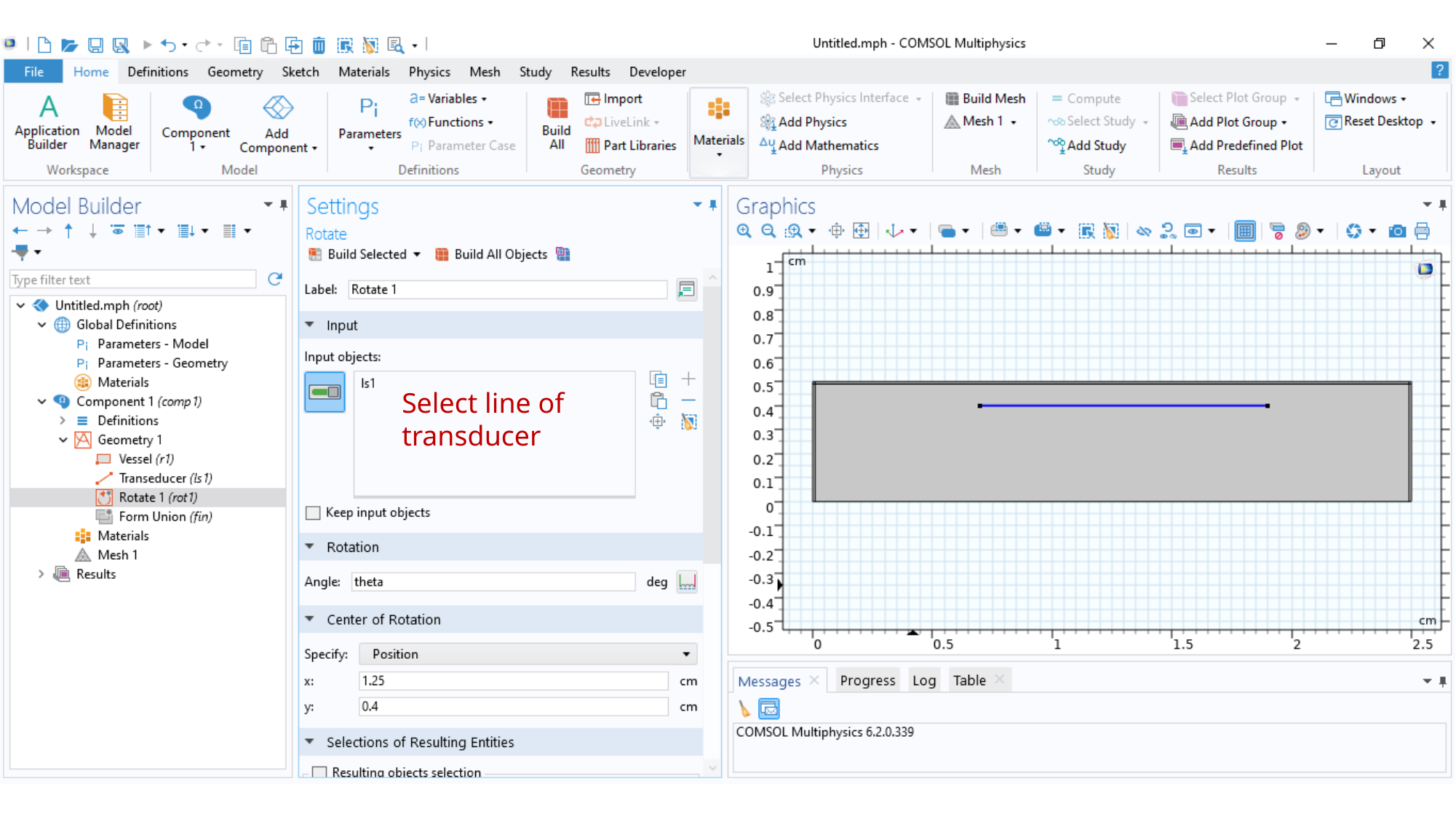

Select line of transducer

## Slide 15
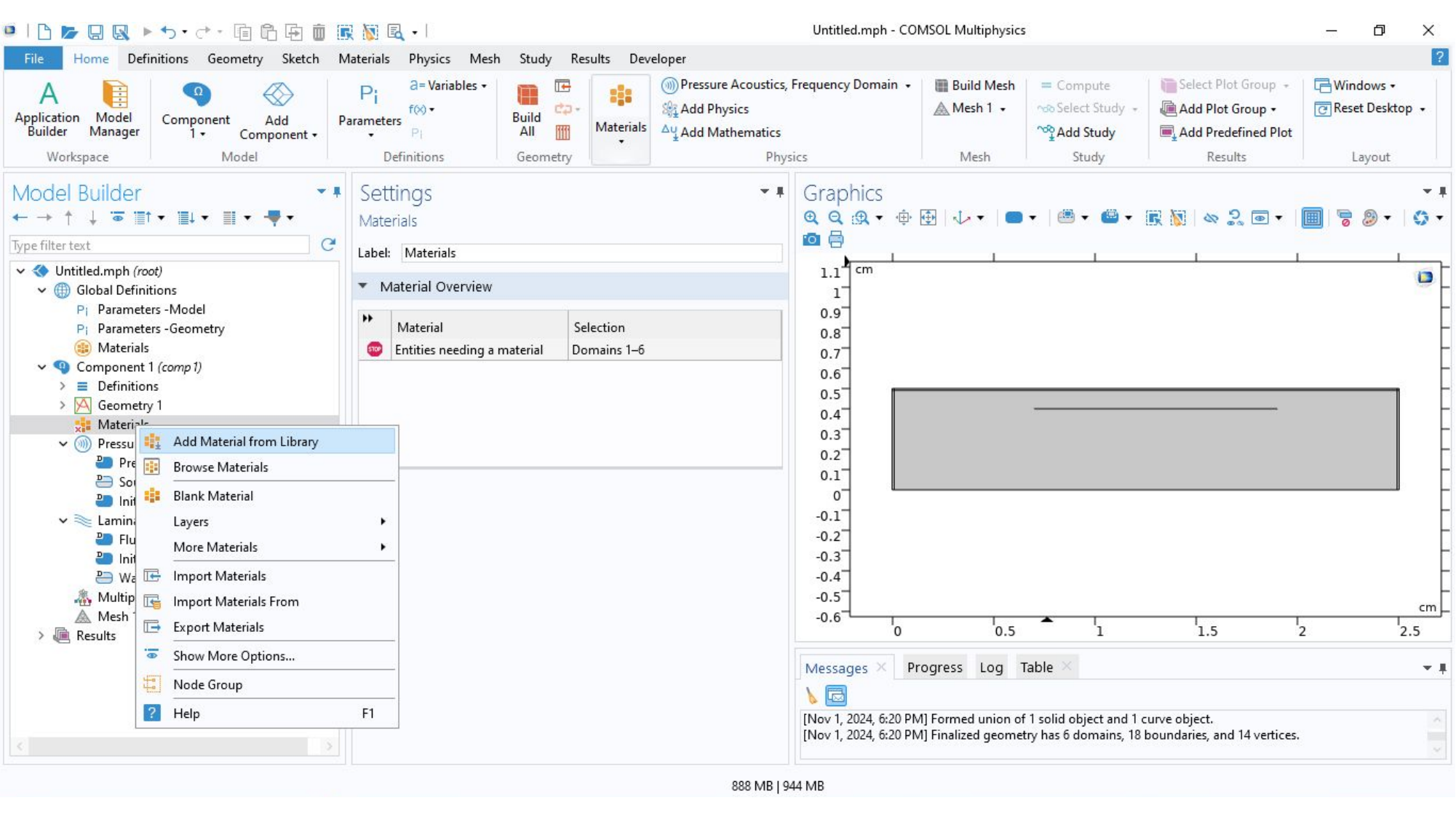

## Slide 16
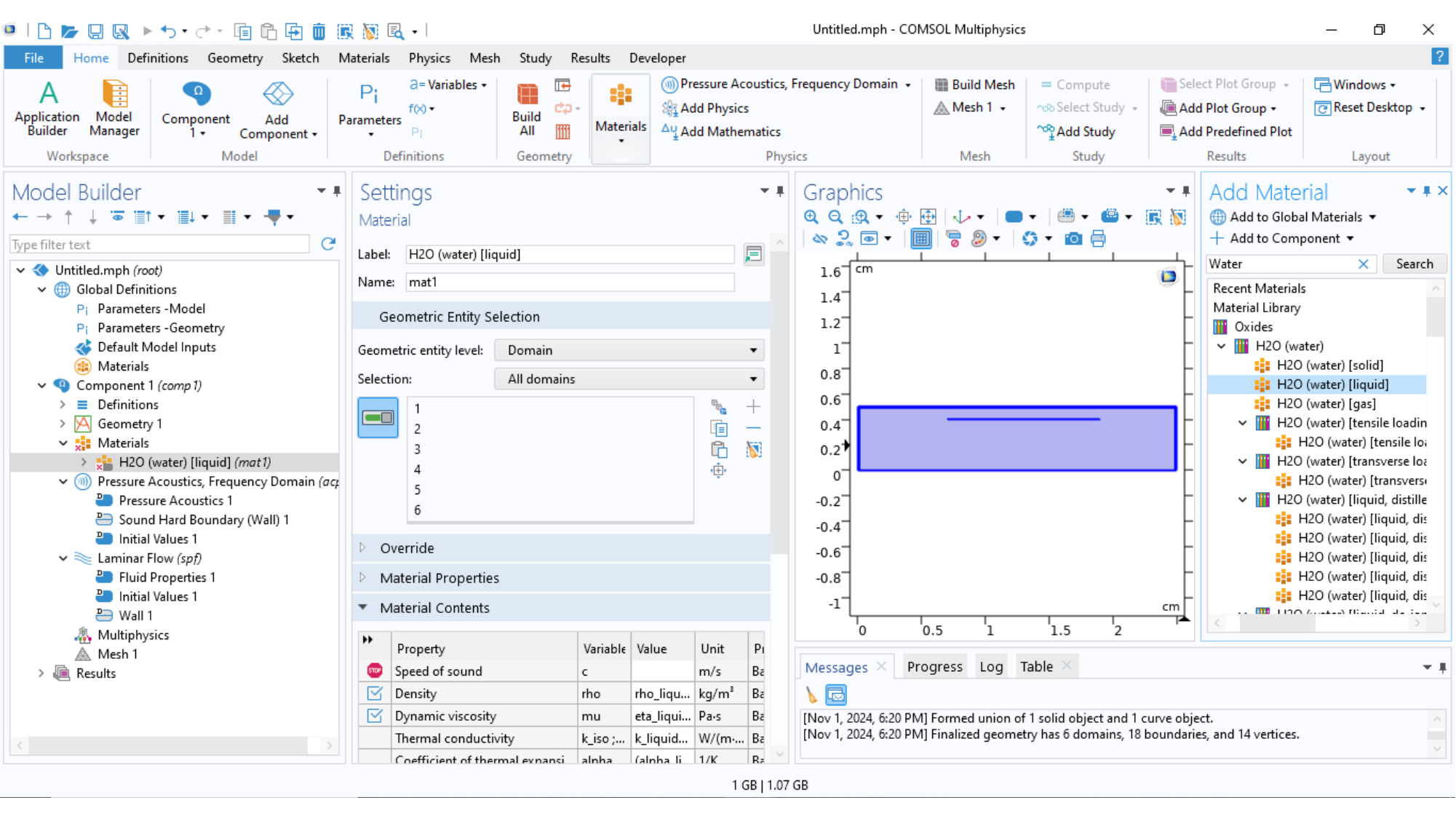

## Slide 17
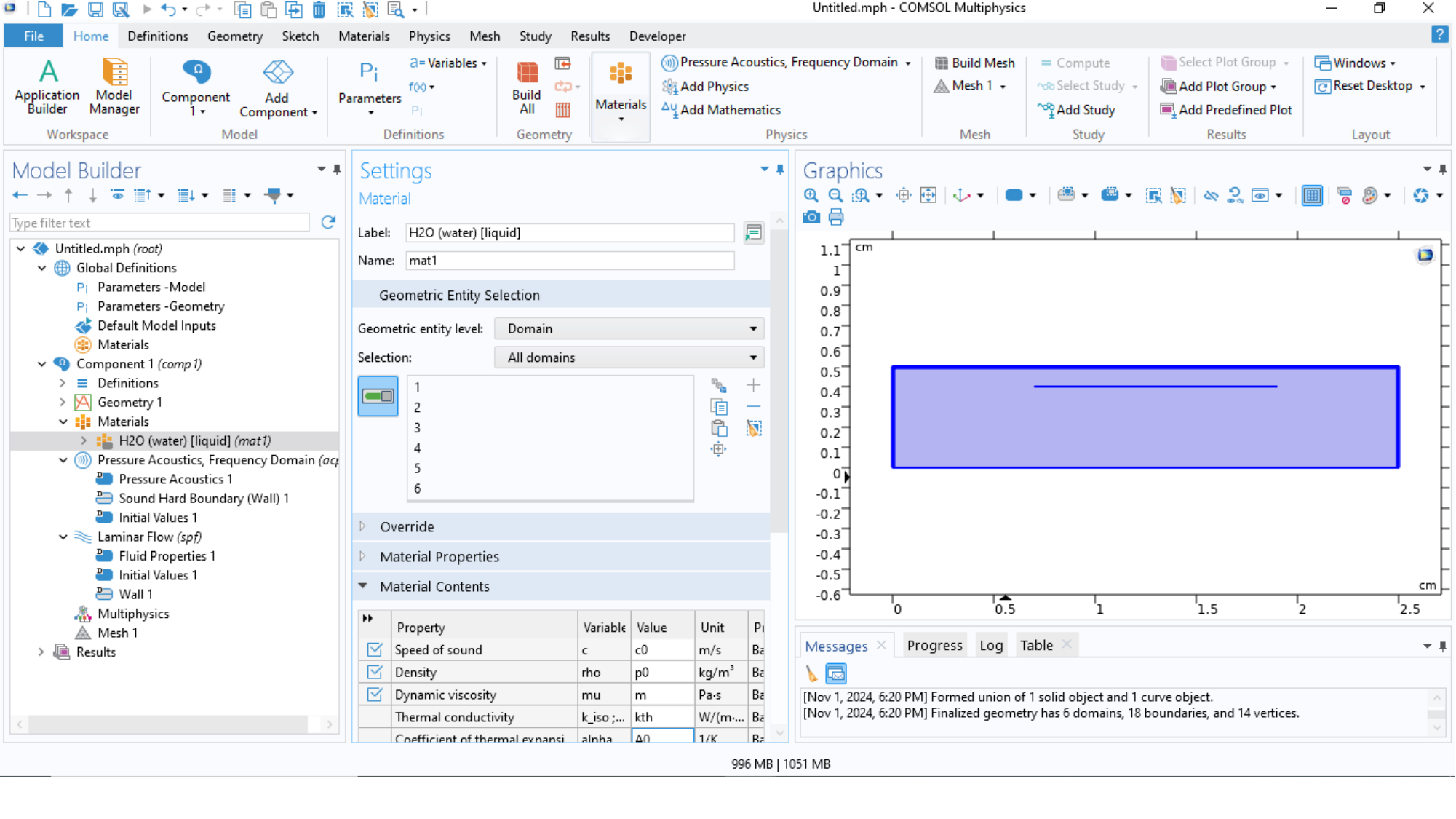

## Slide 18
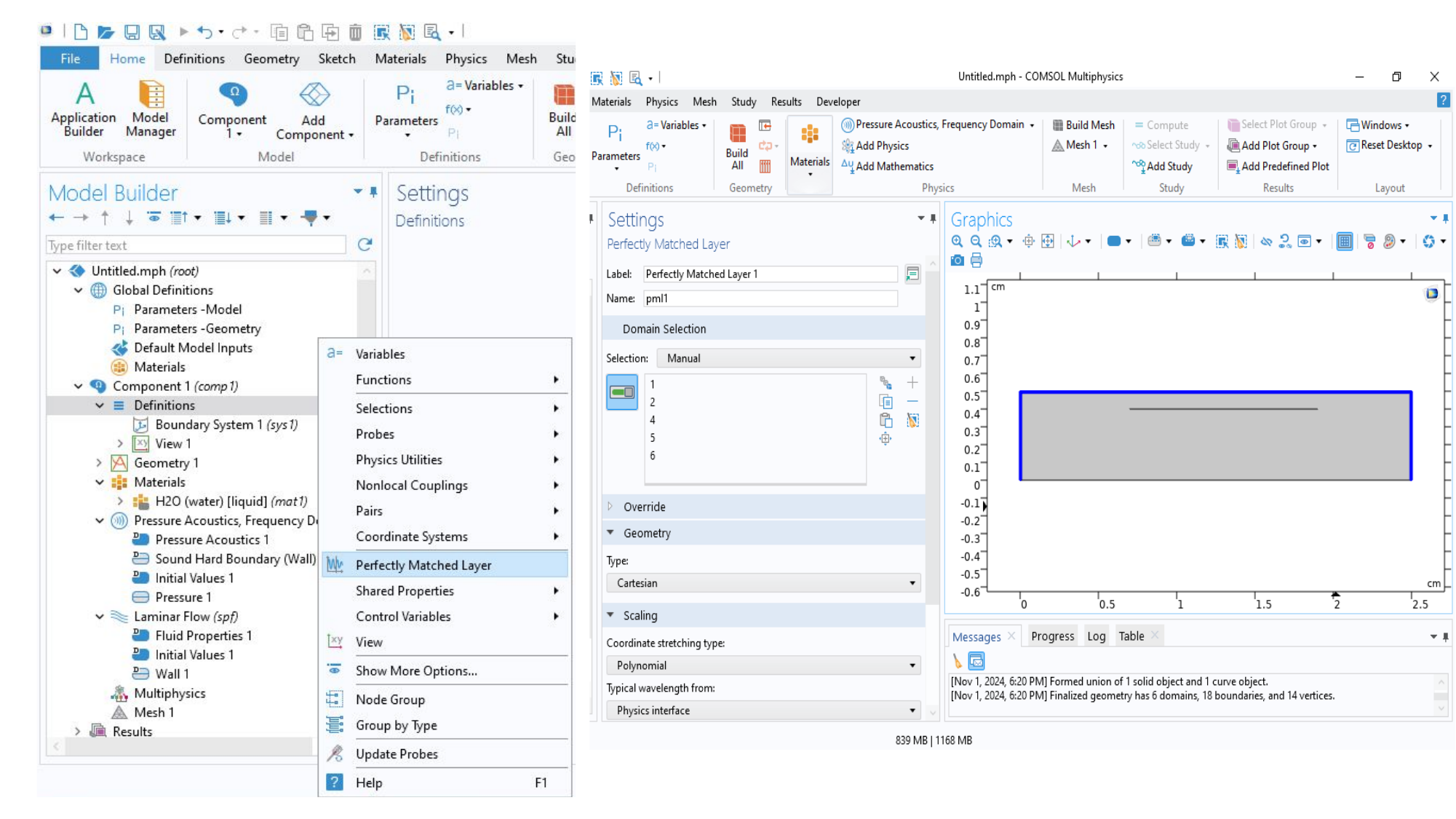

## Slide 19
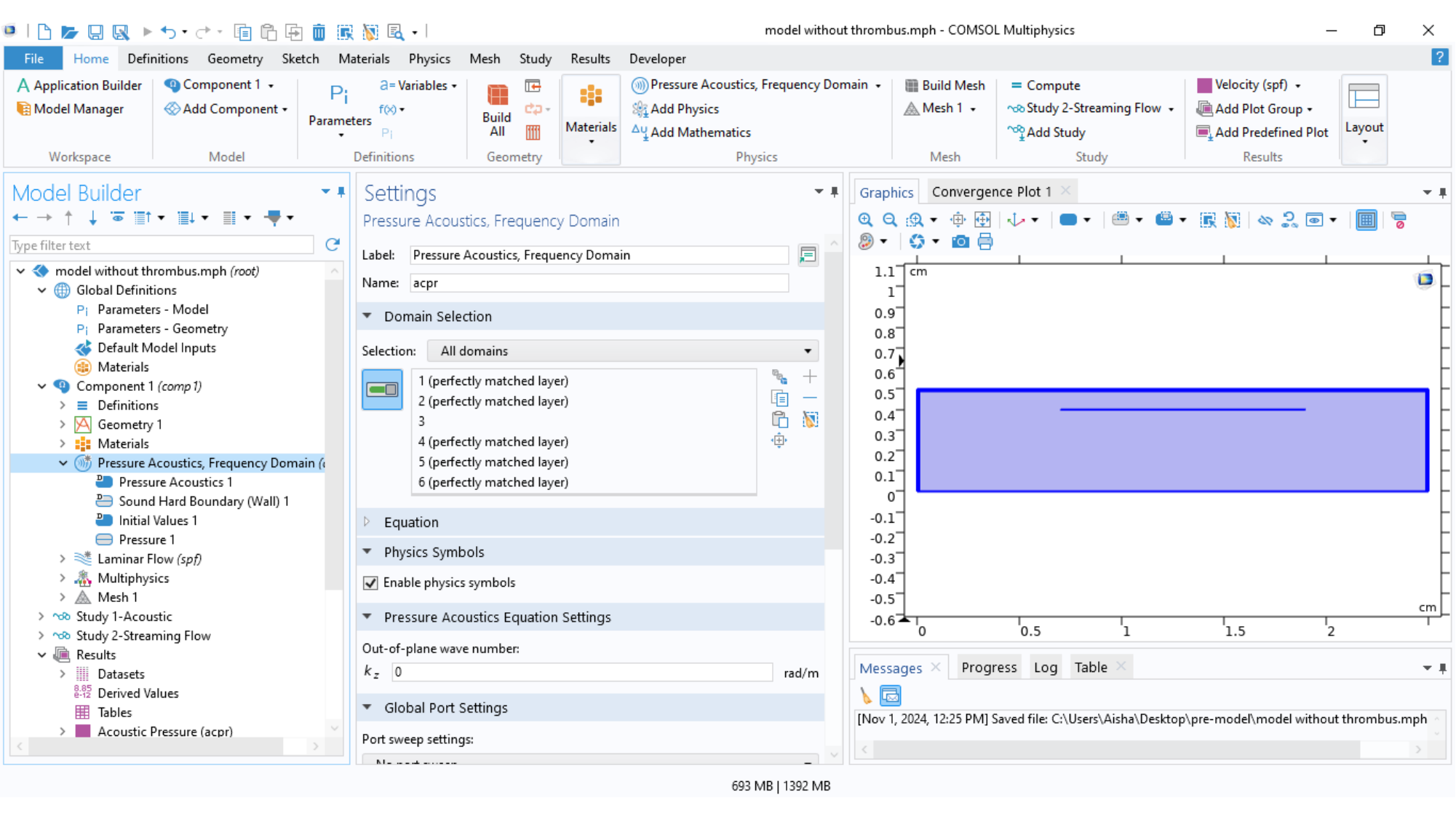

## Slide 20
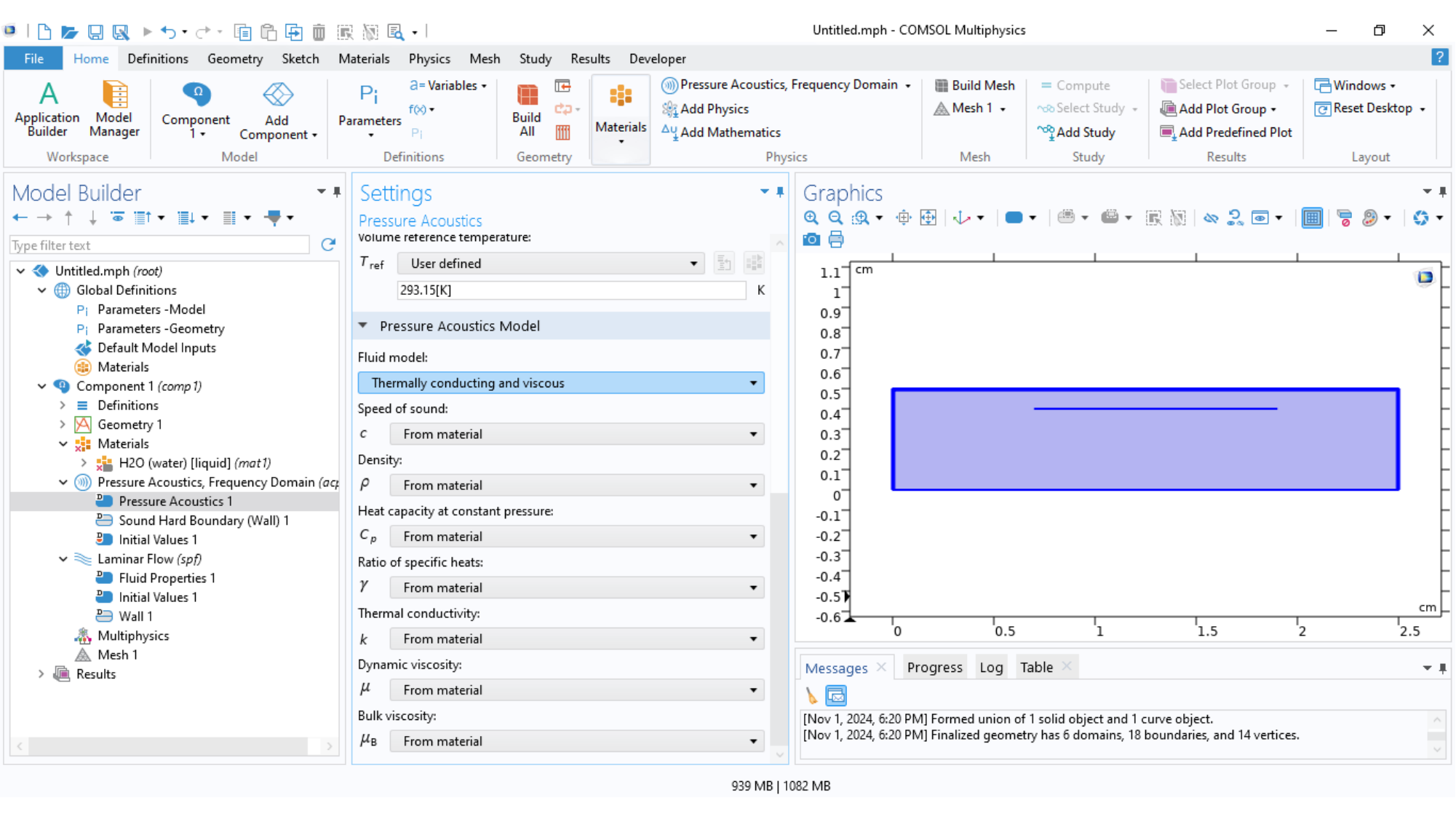

## Slide 21
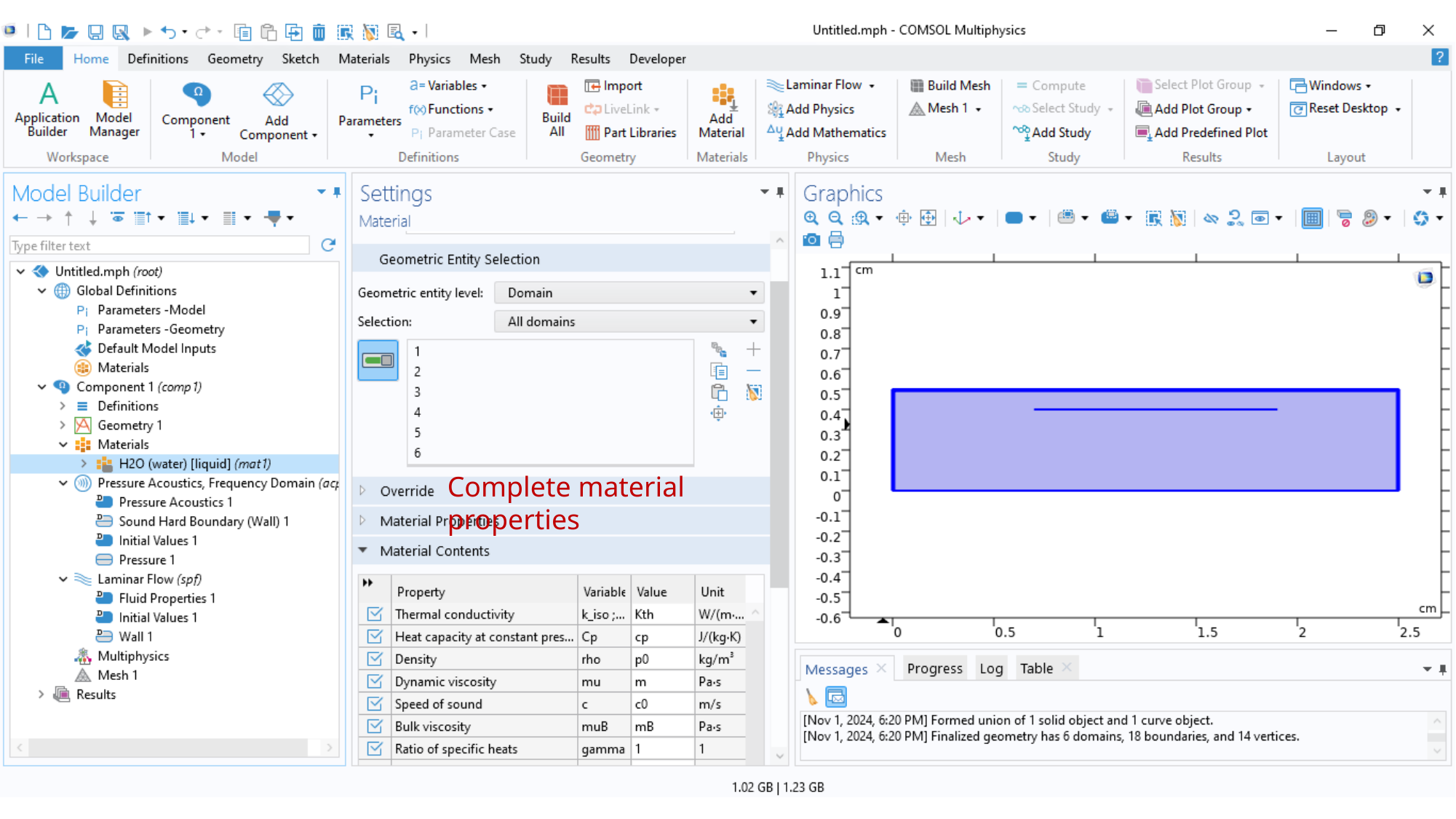

Complete material properties

## Slide 22
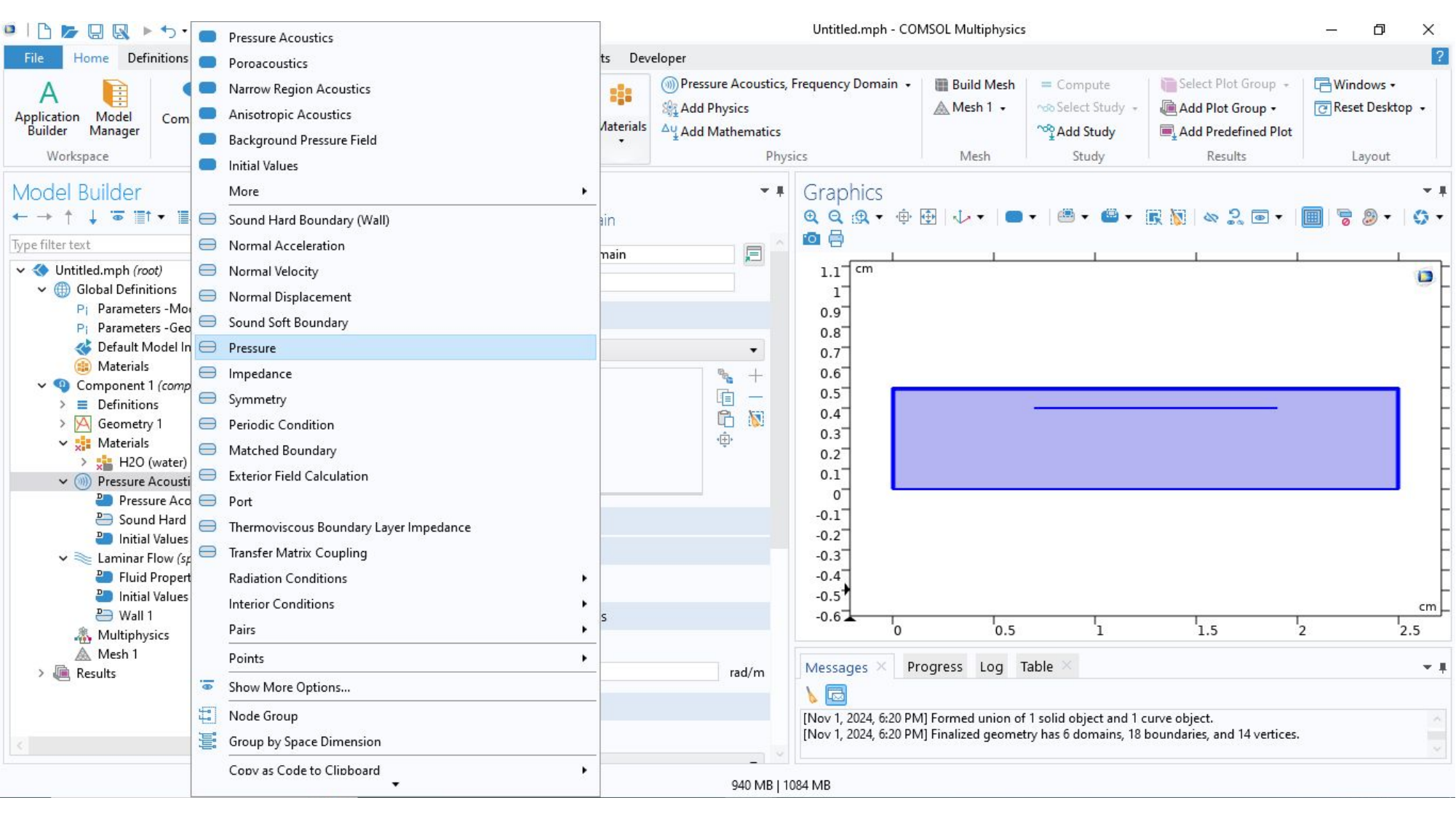

## Slide 23
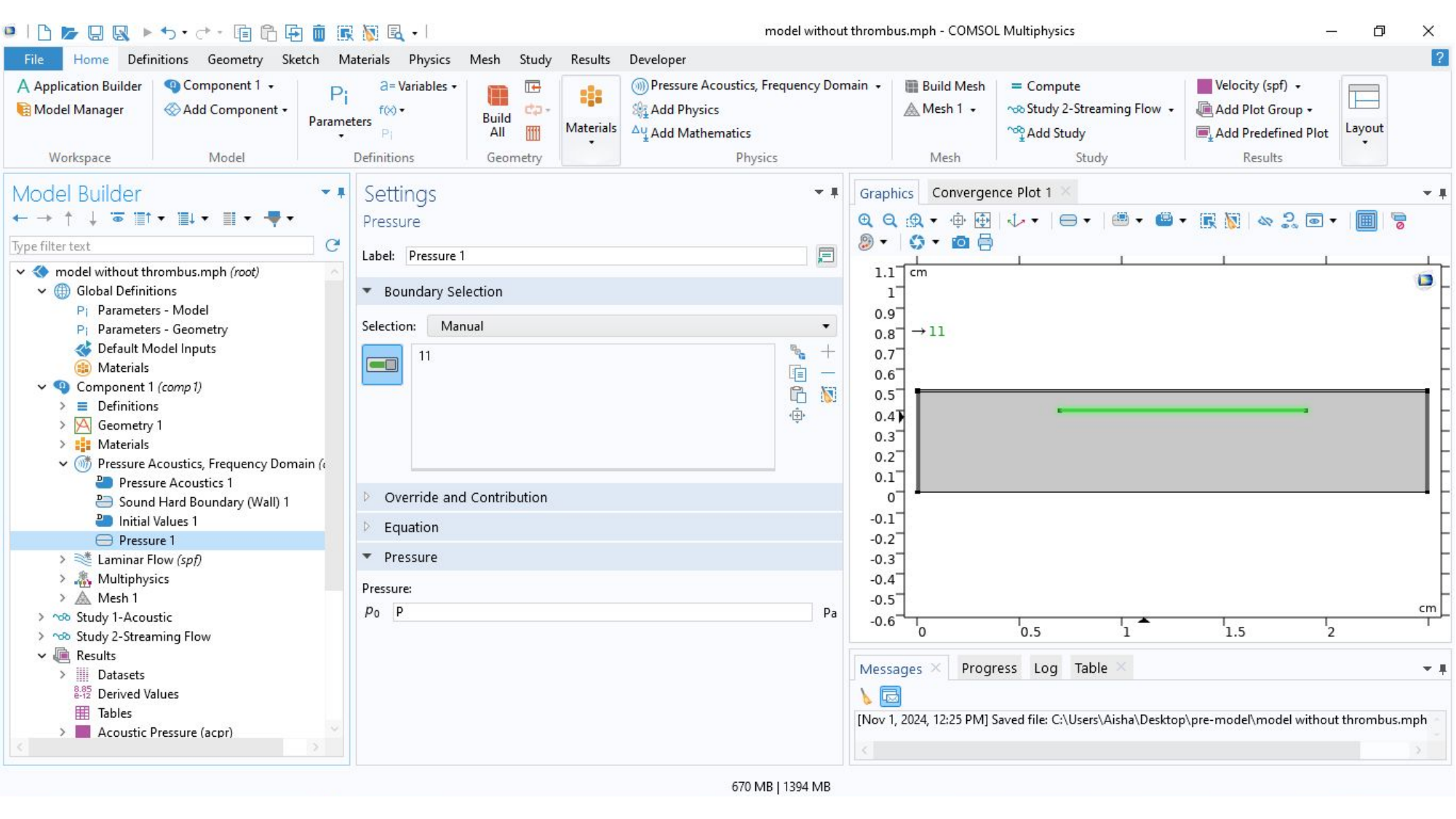

## Slide 24
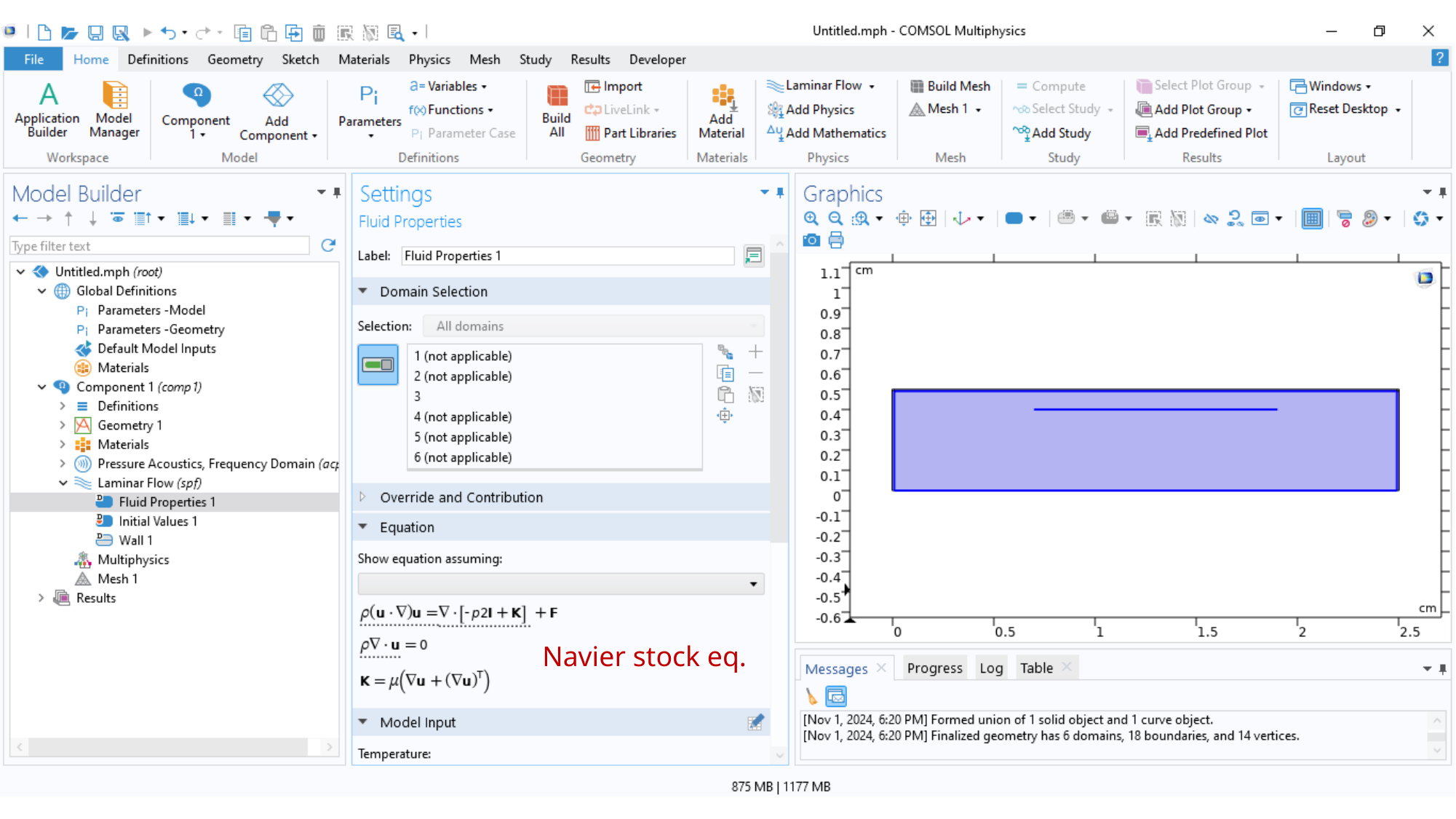

Navier stock eq.

## Slide 25
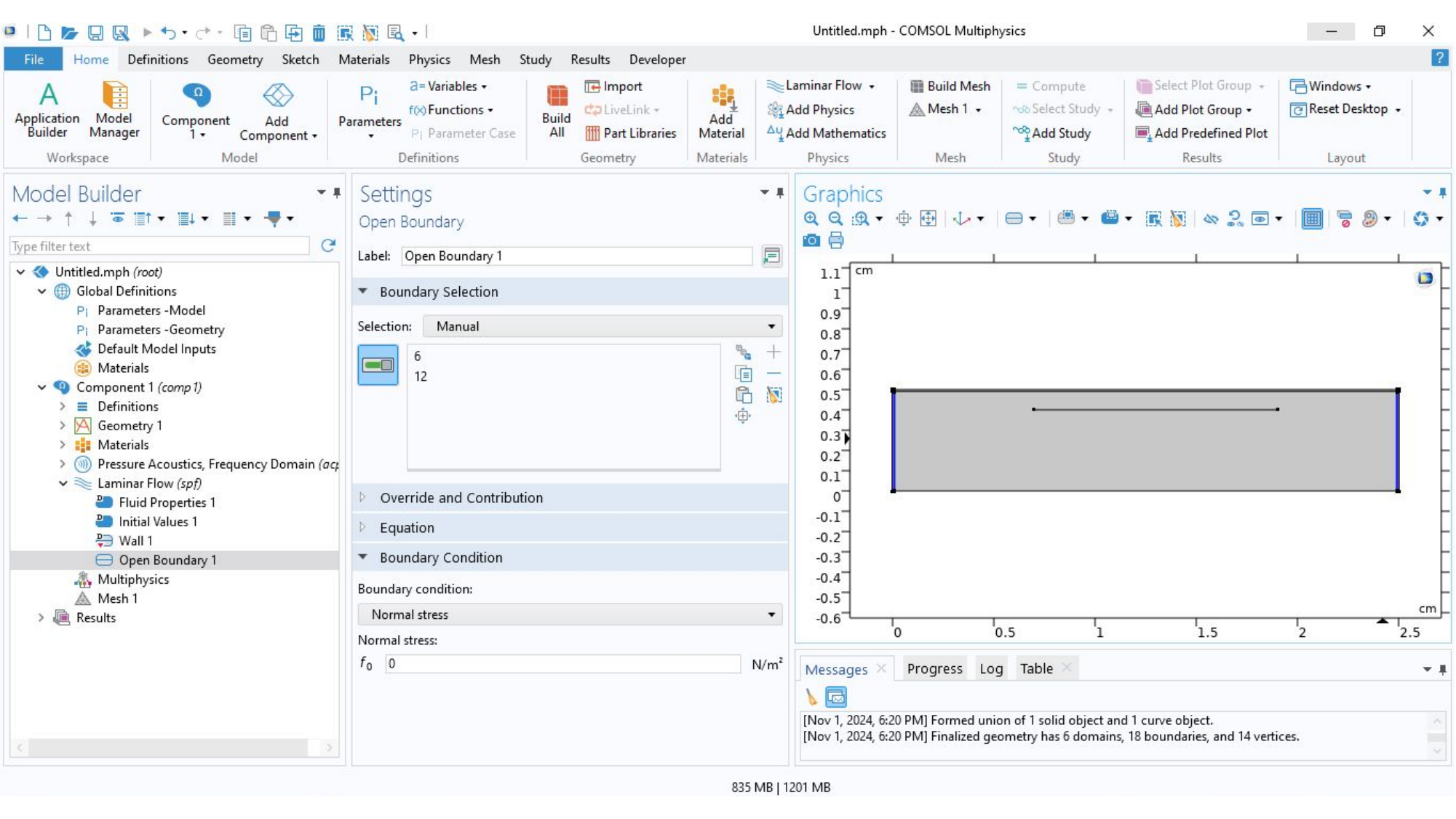

## Slide 26
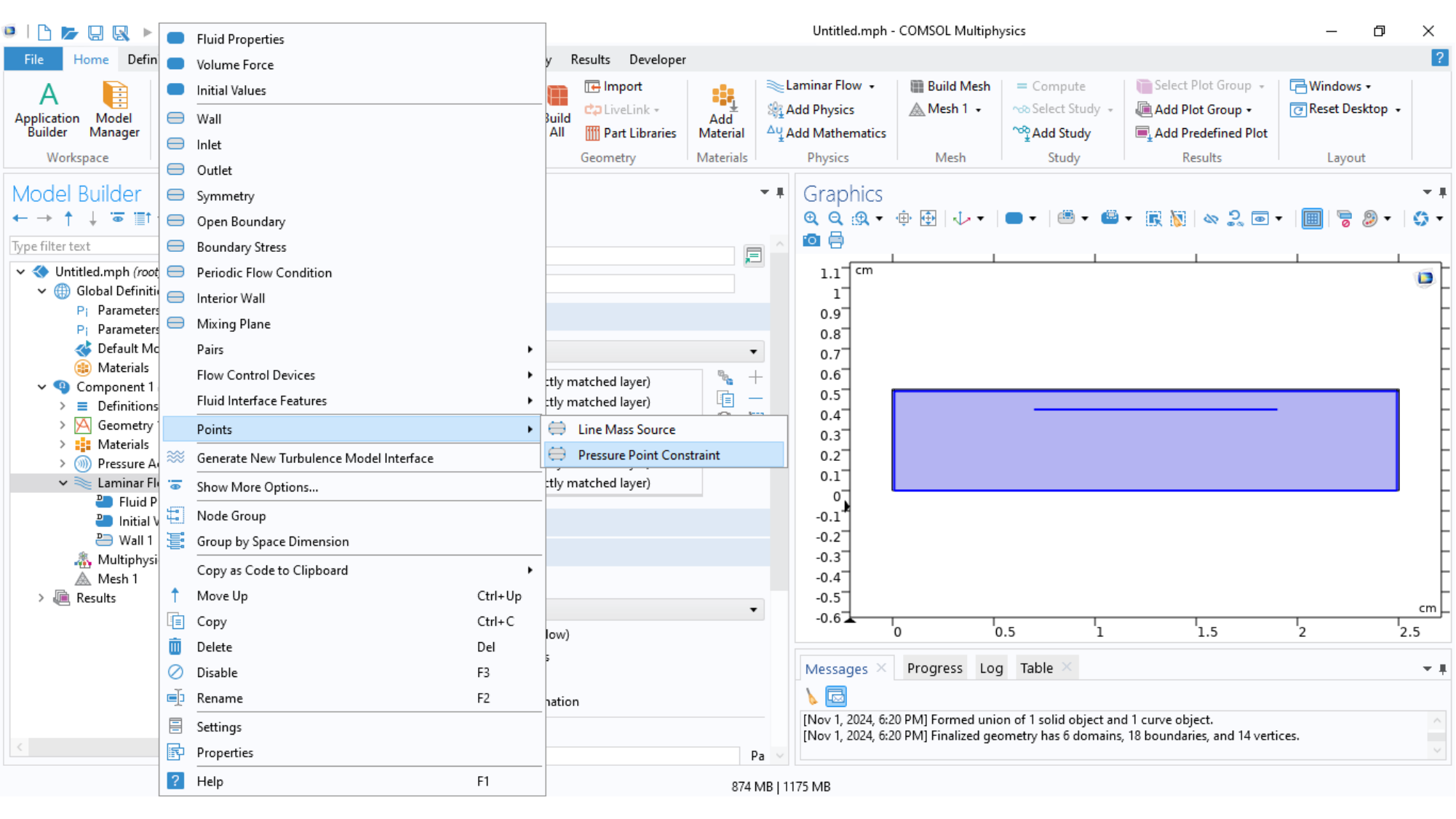

## Slide 27
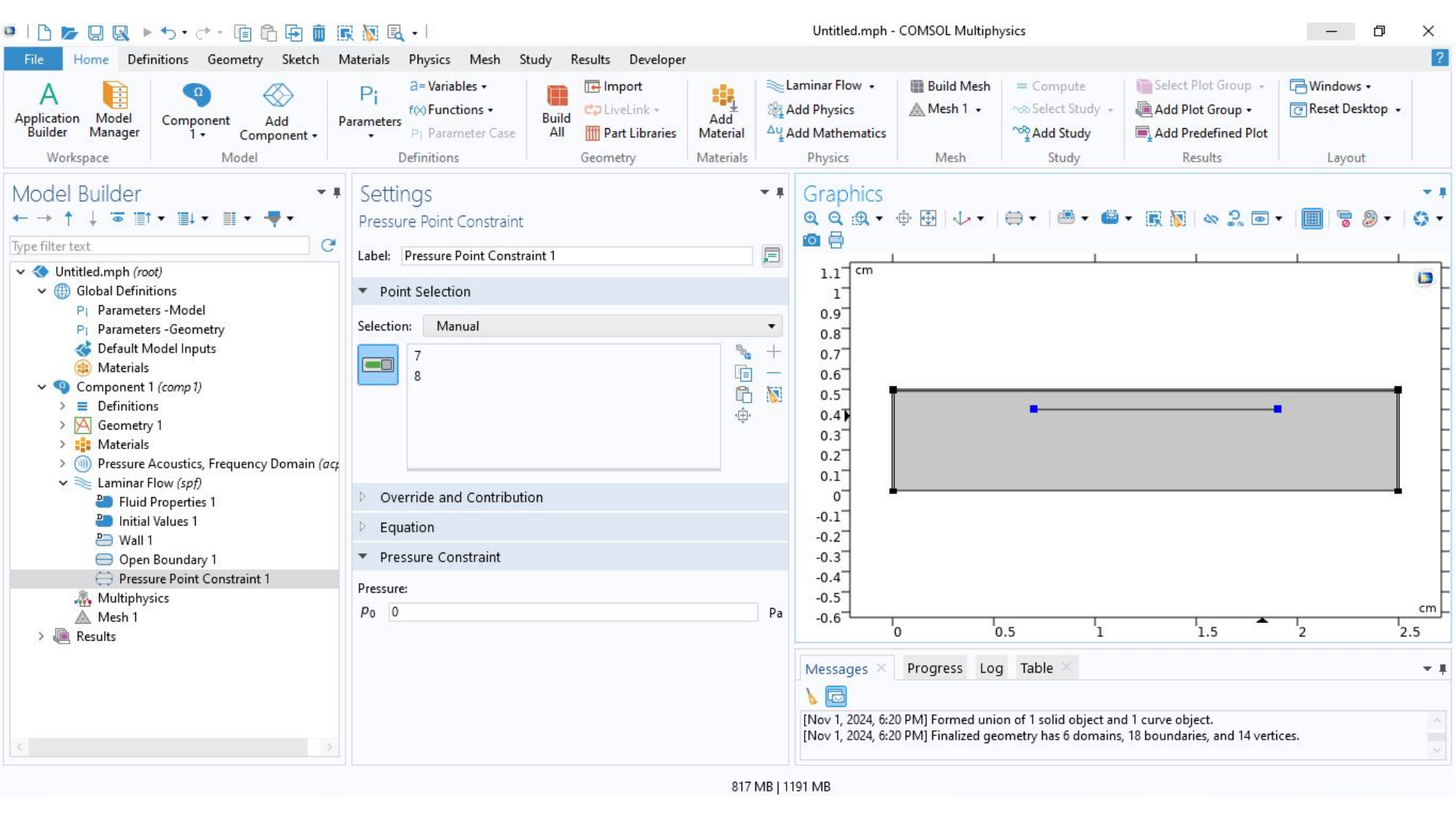

## Slide 28
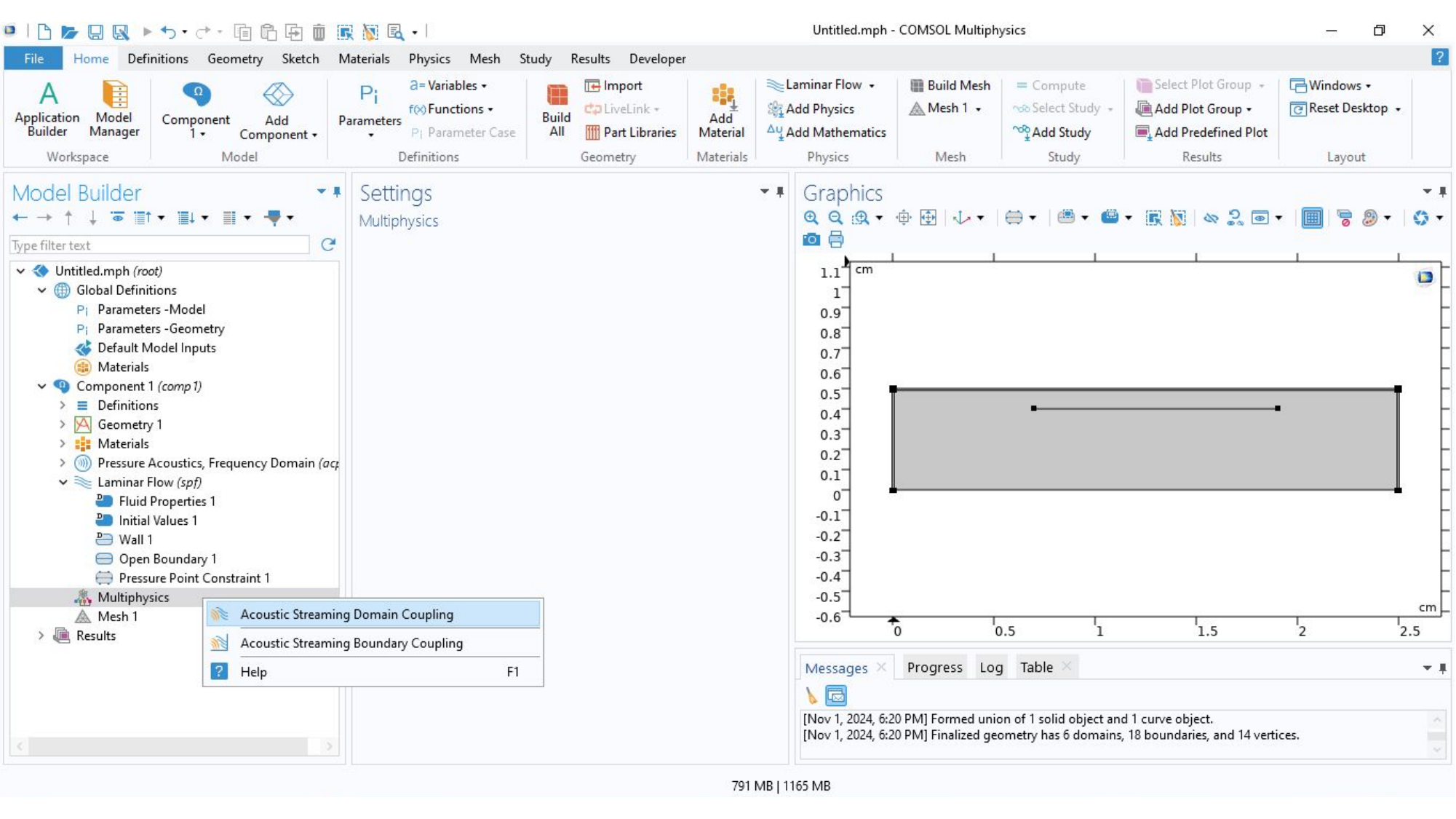

## Slide 29
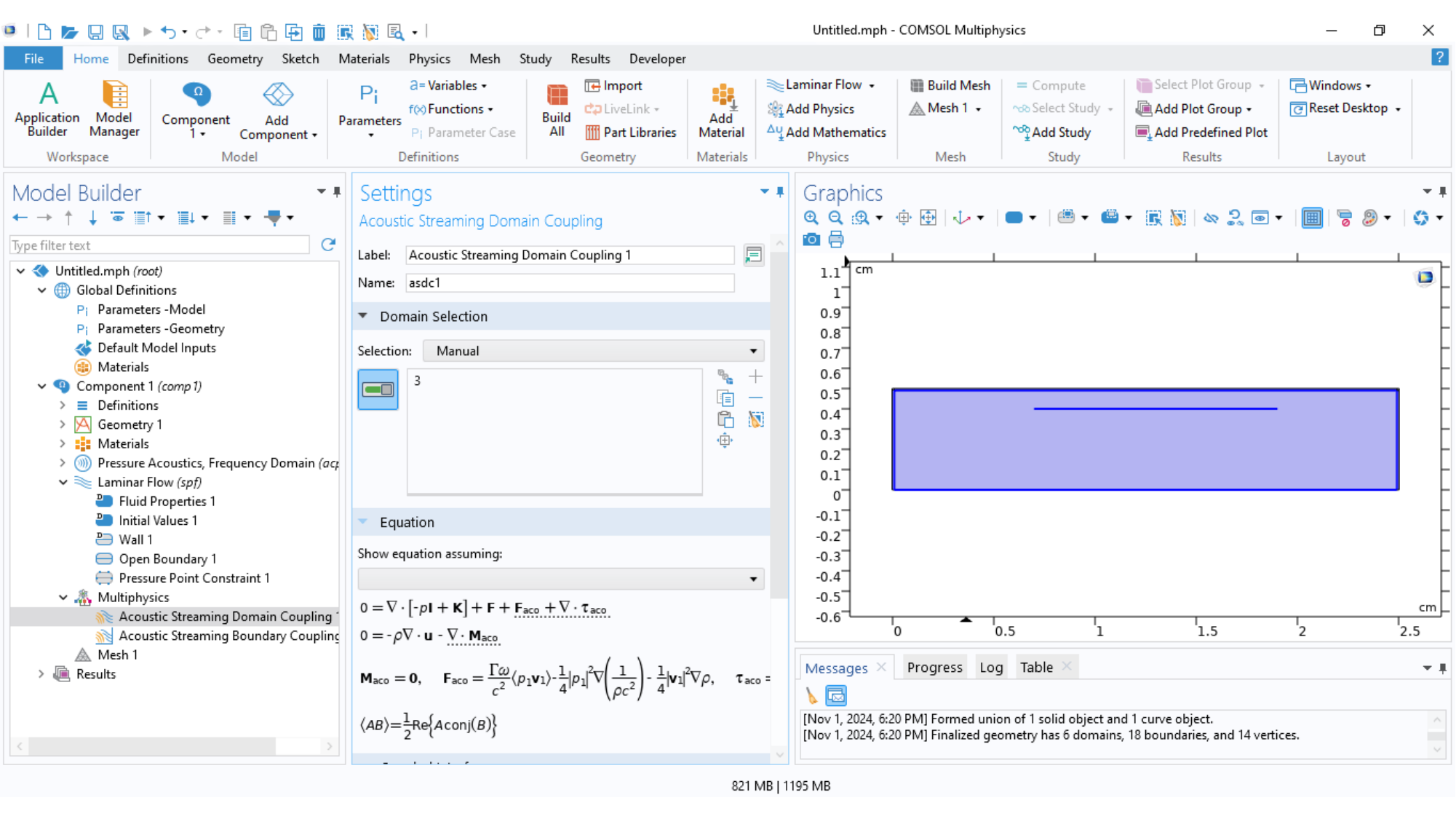

## Slide 30
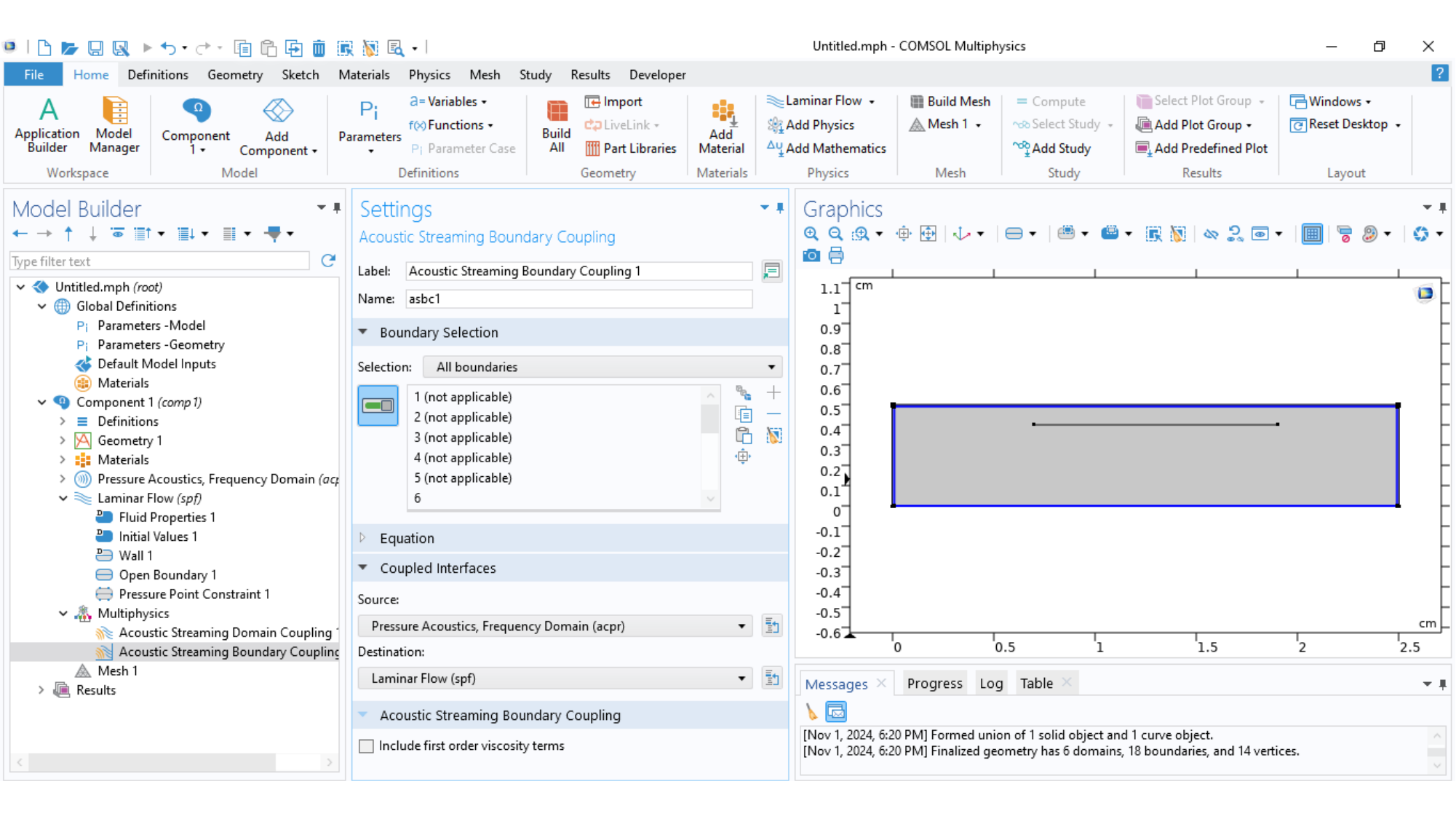

## Slide 31
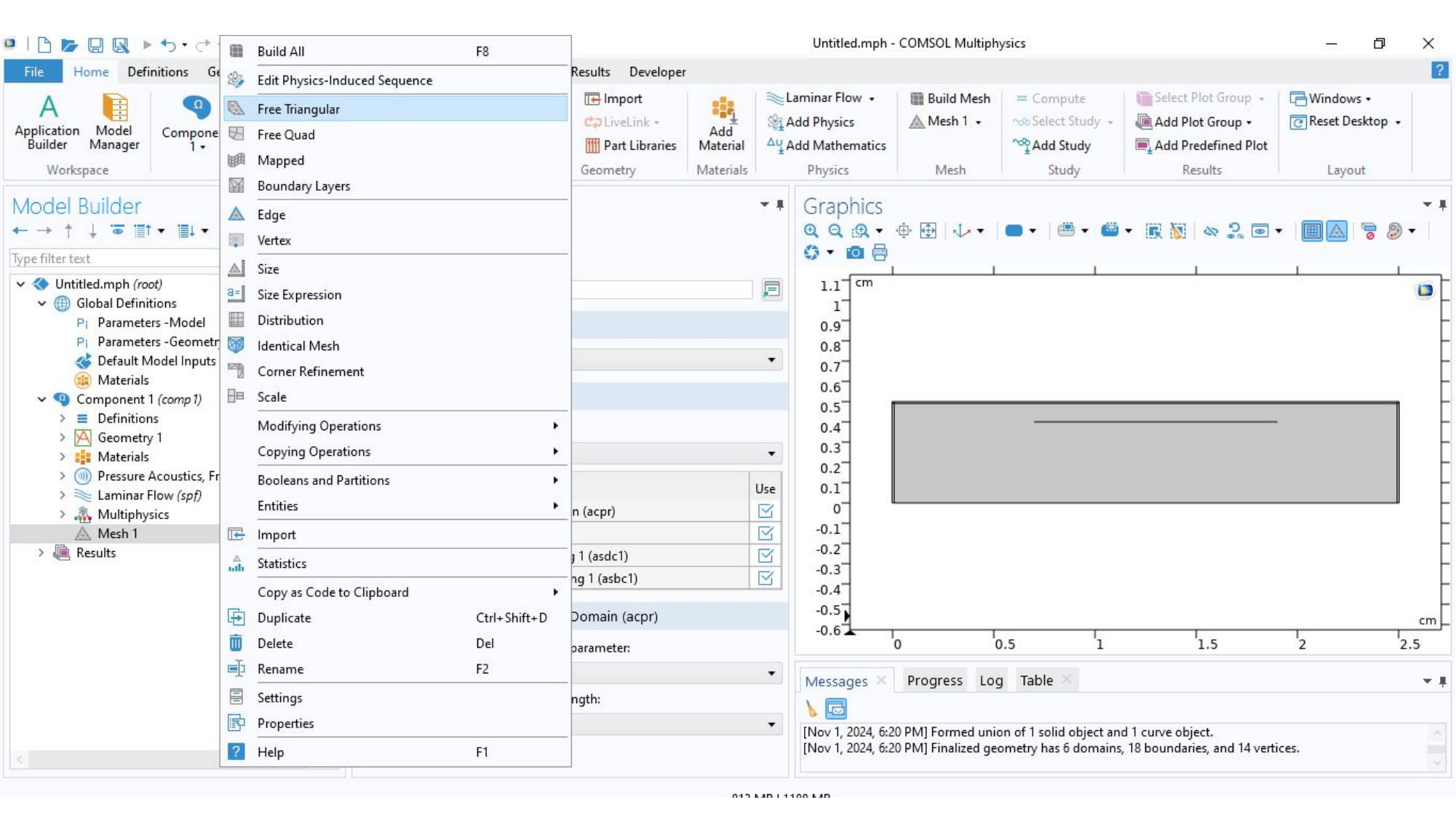

## Slide 32
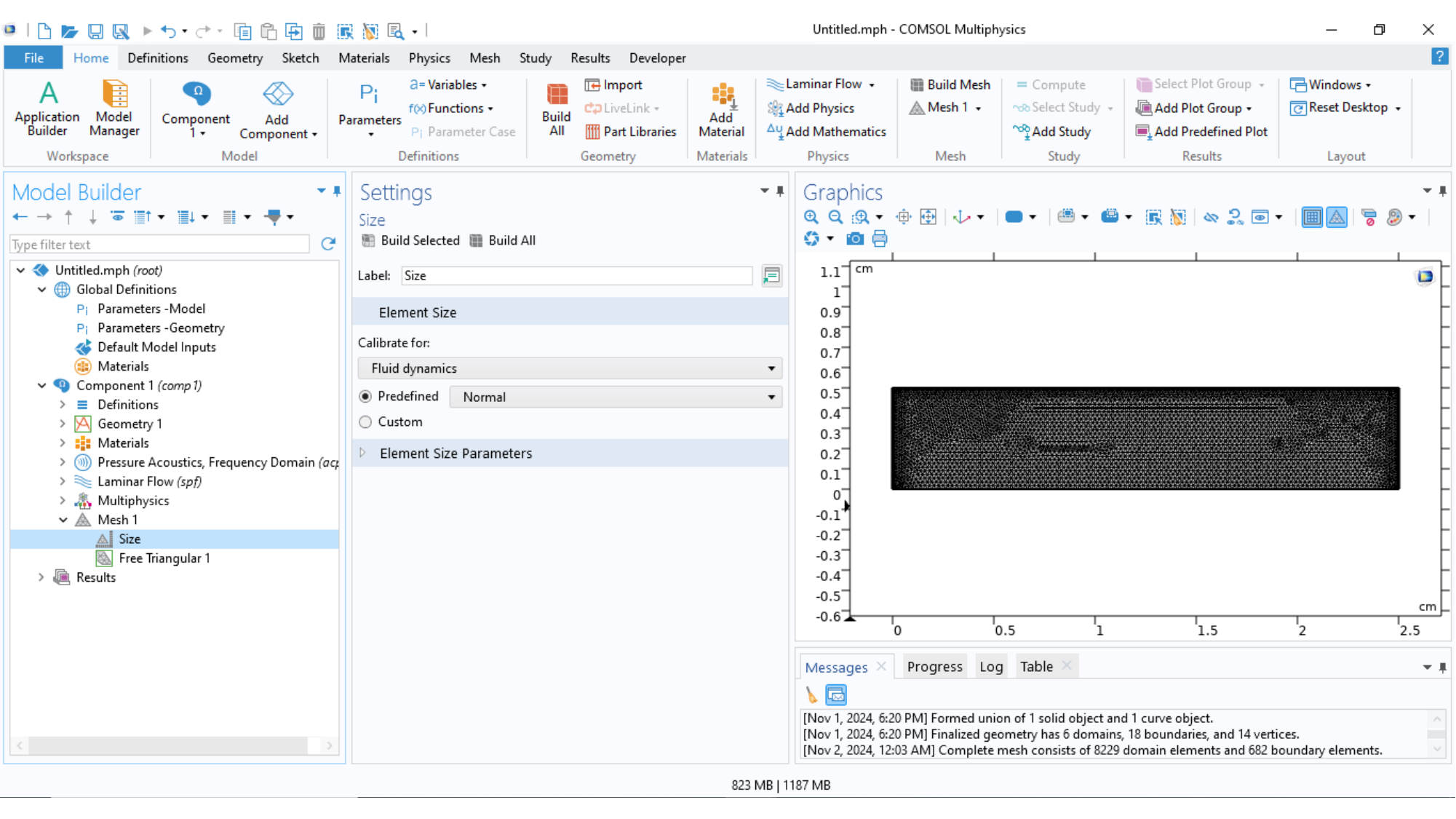

## Slide 33
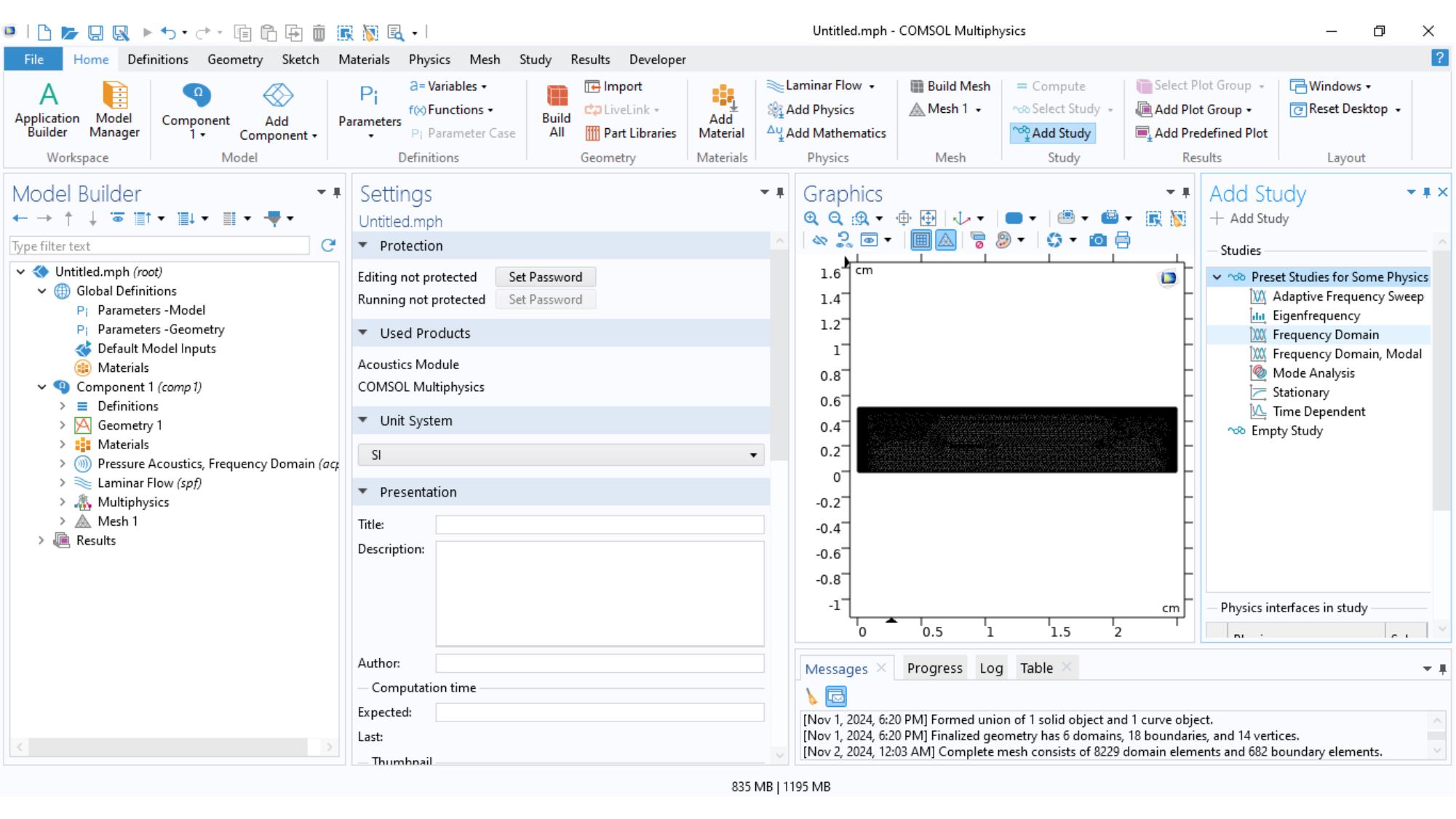

## Slide 34
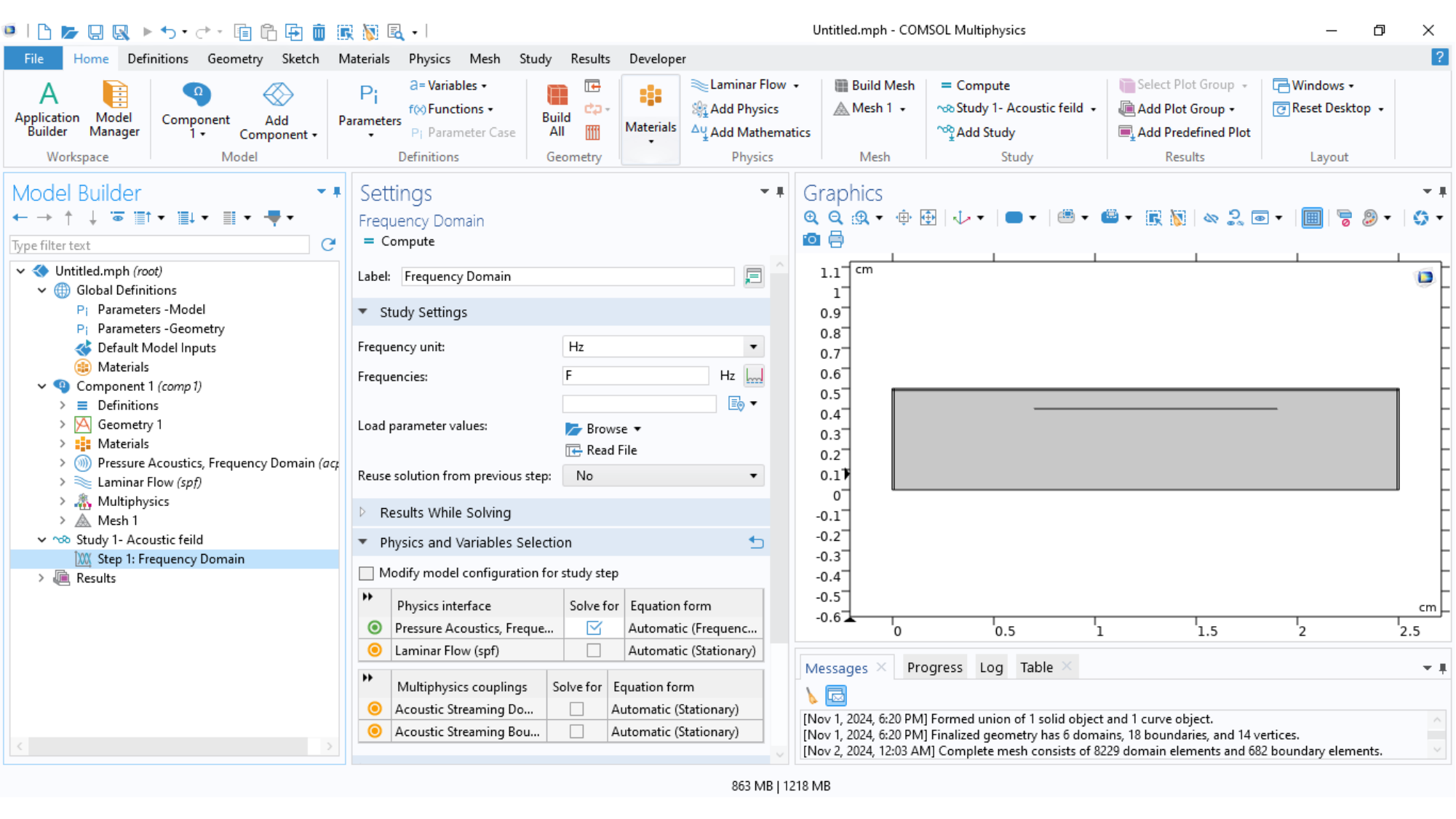

## Slide 35
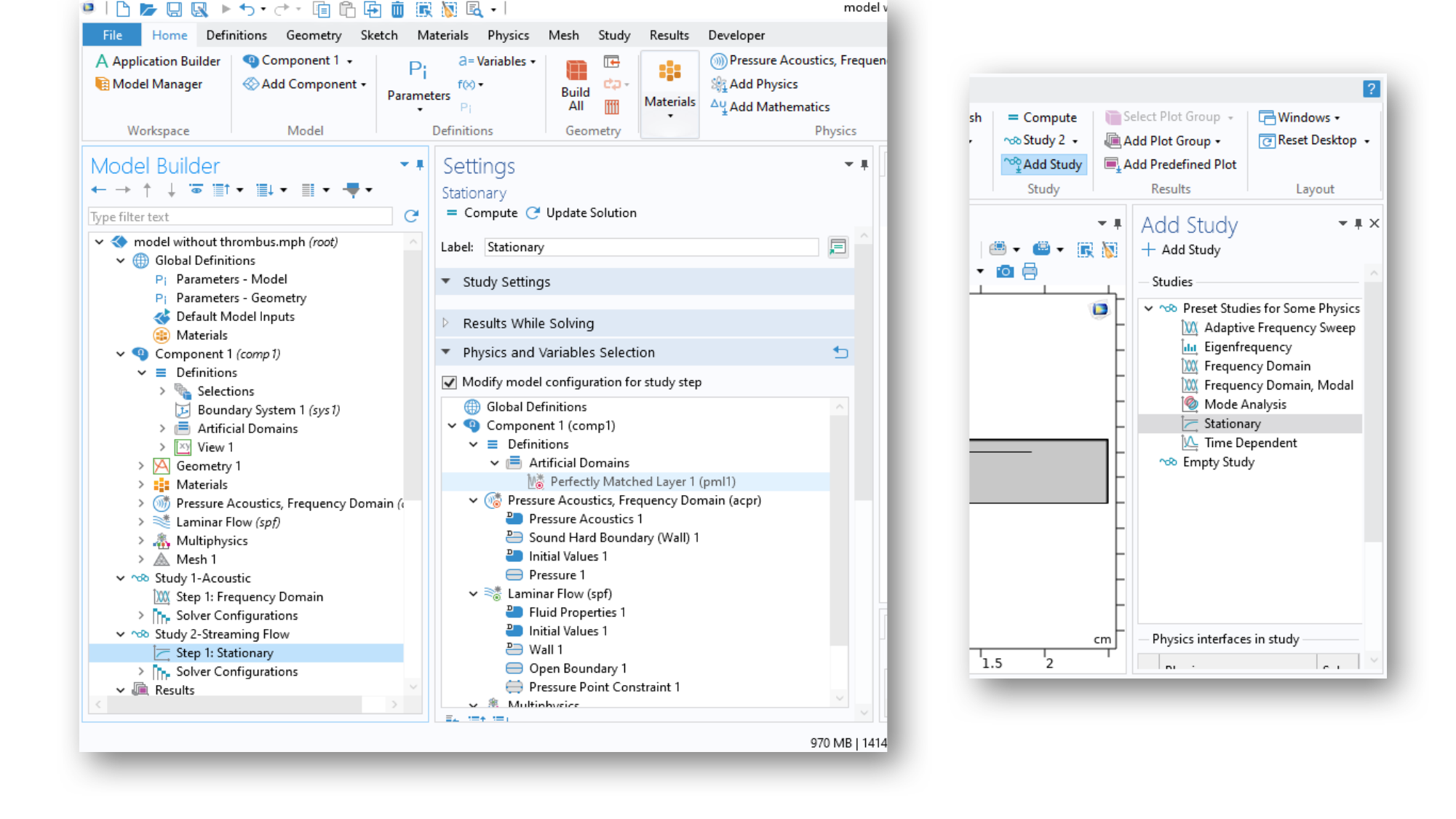

## Slide 36
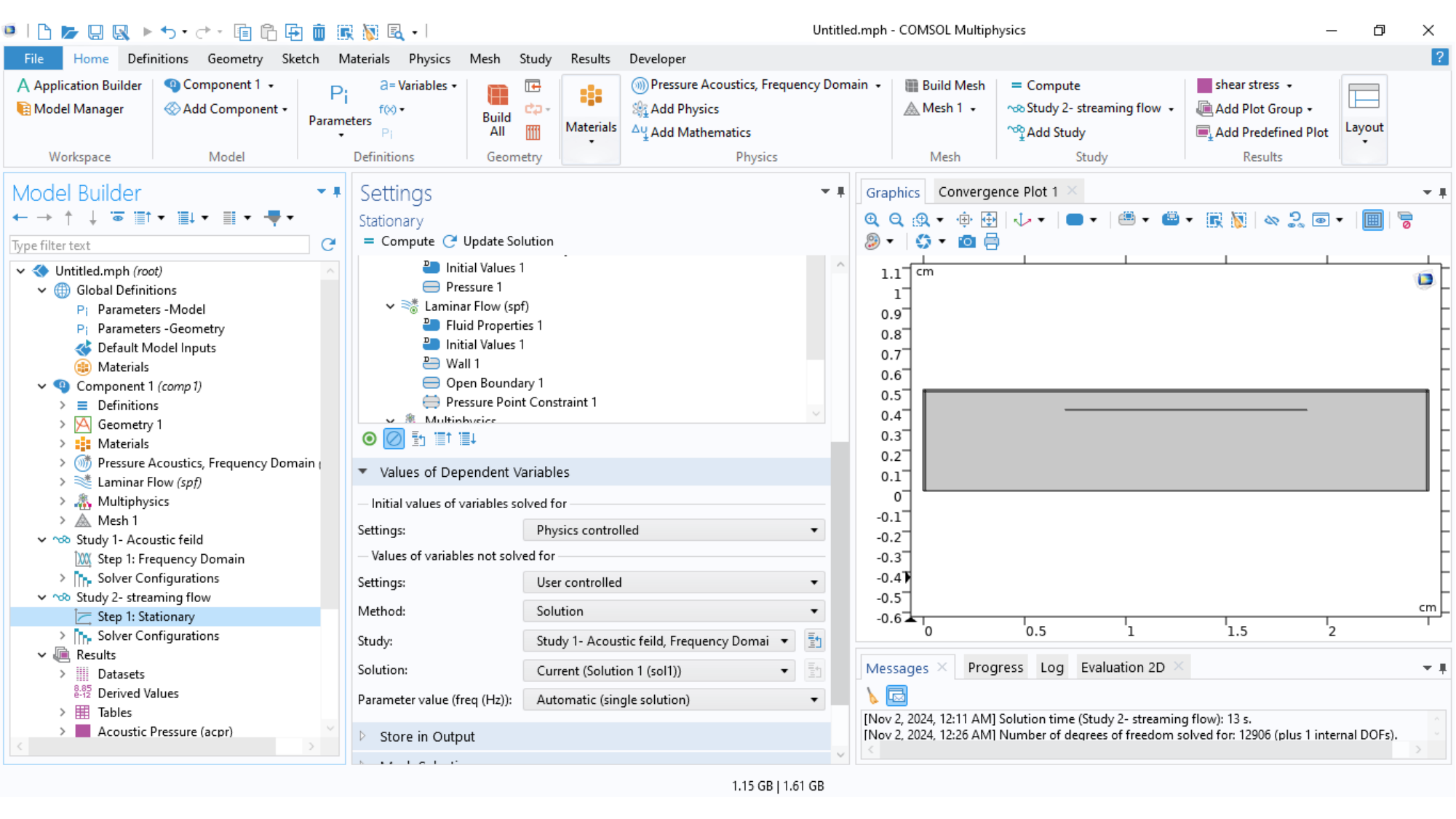

## Slide 37
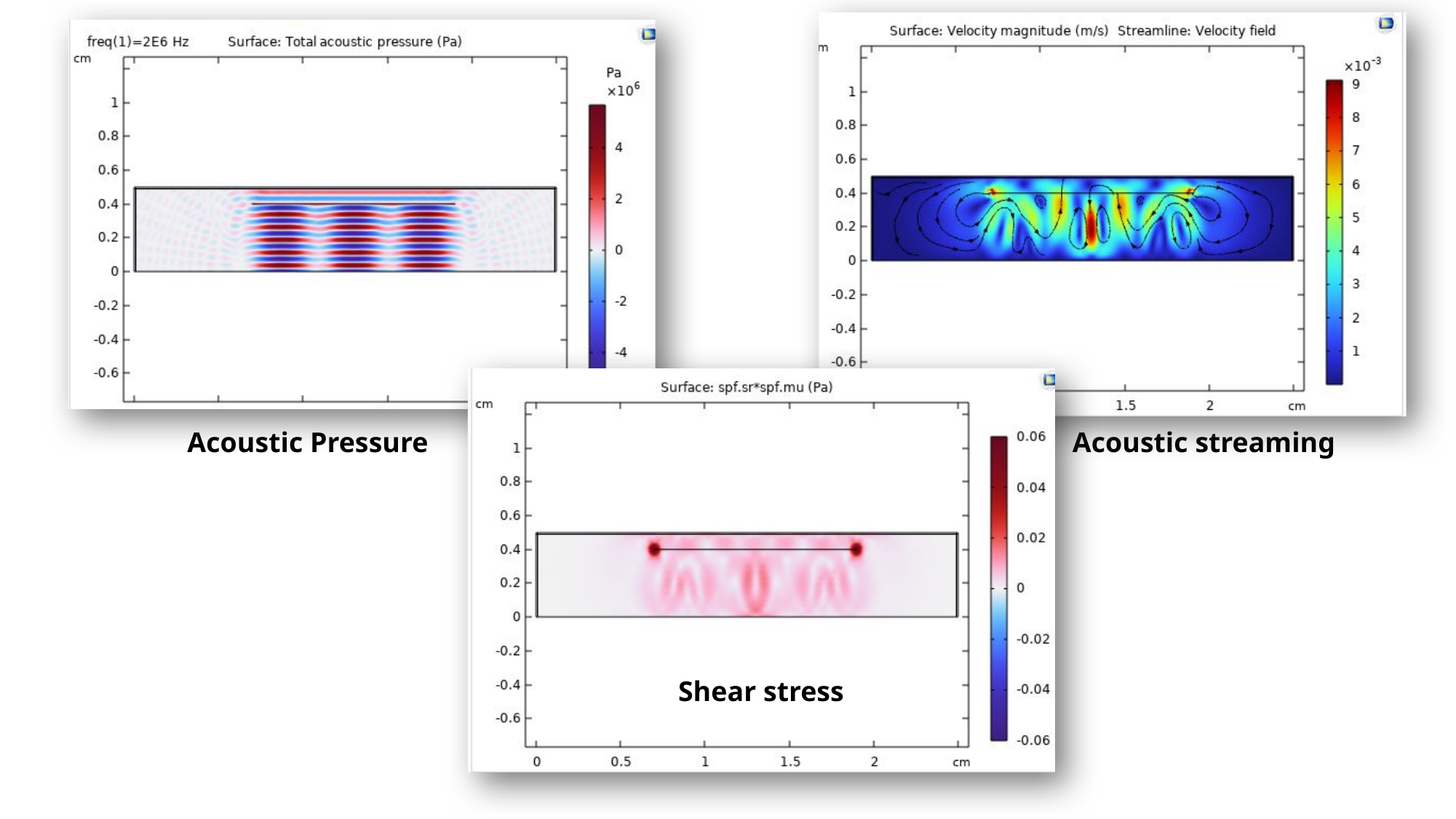

Acoustic Pressure
Acoustic streaming
Shear stress

## Slide 38
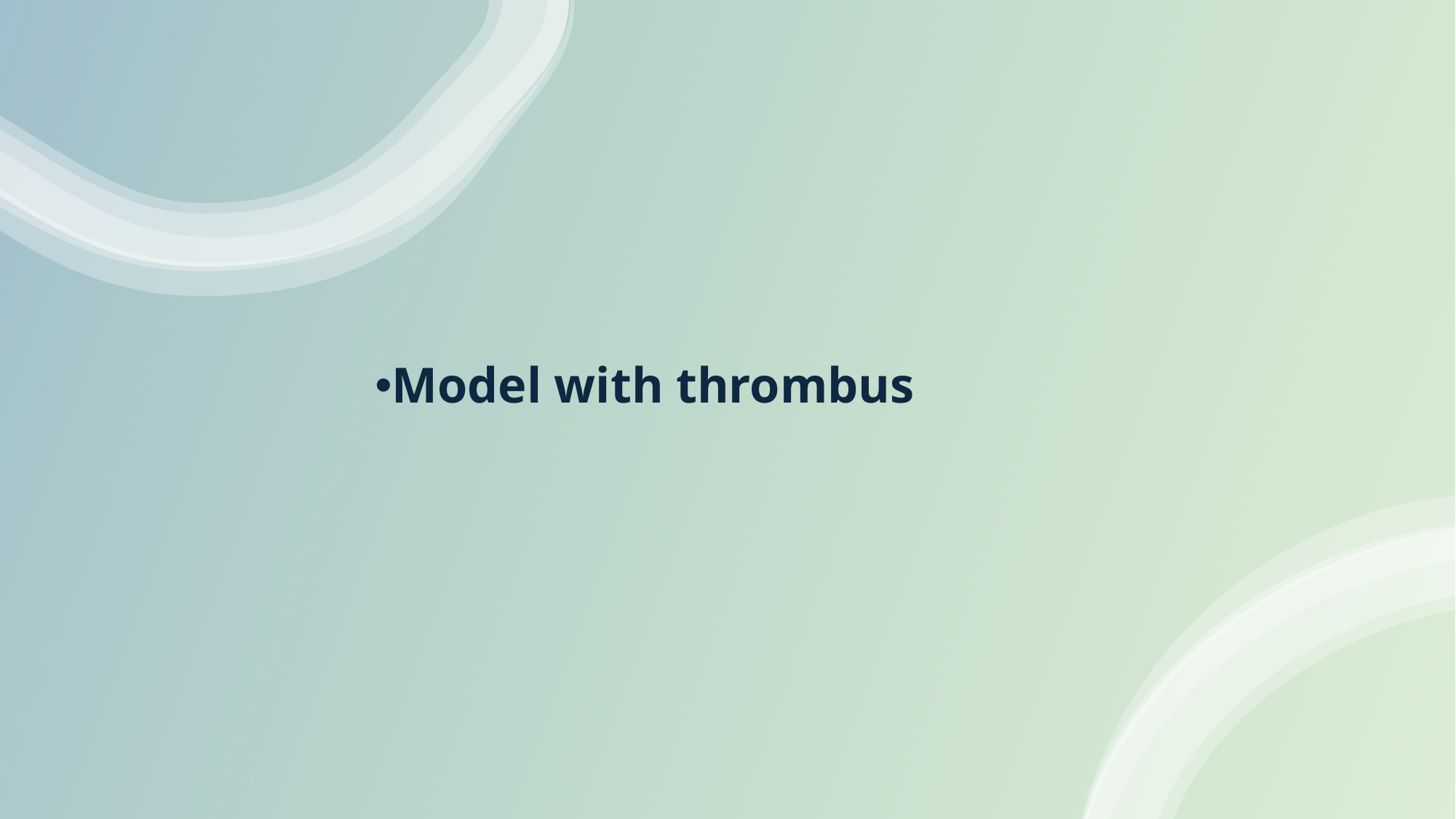

Model with thrombus

## Slide 39
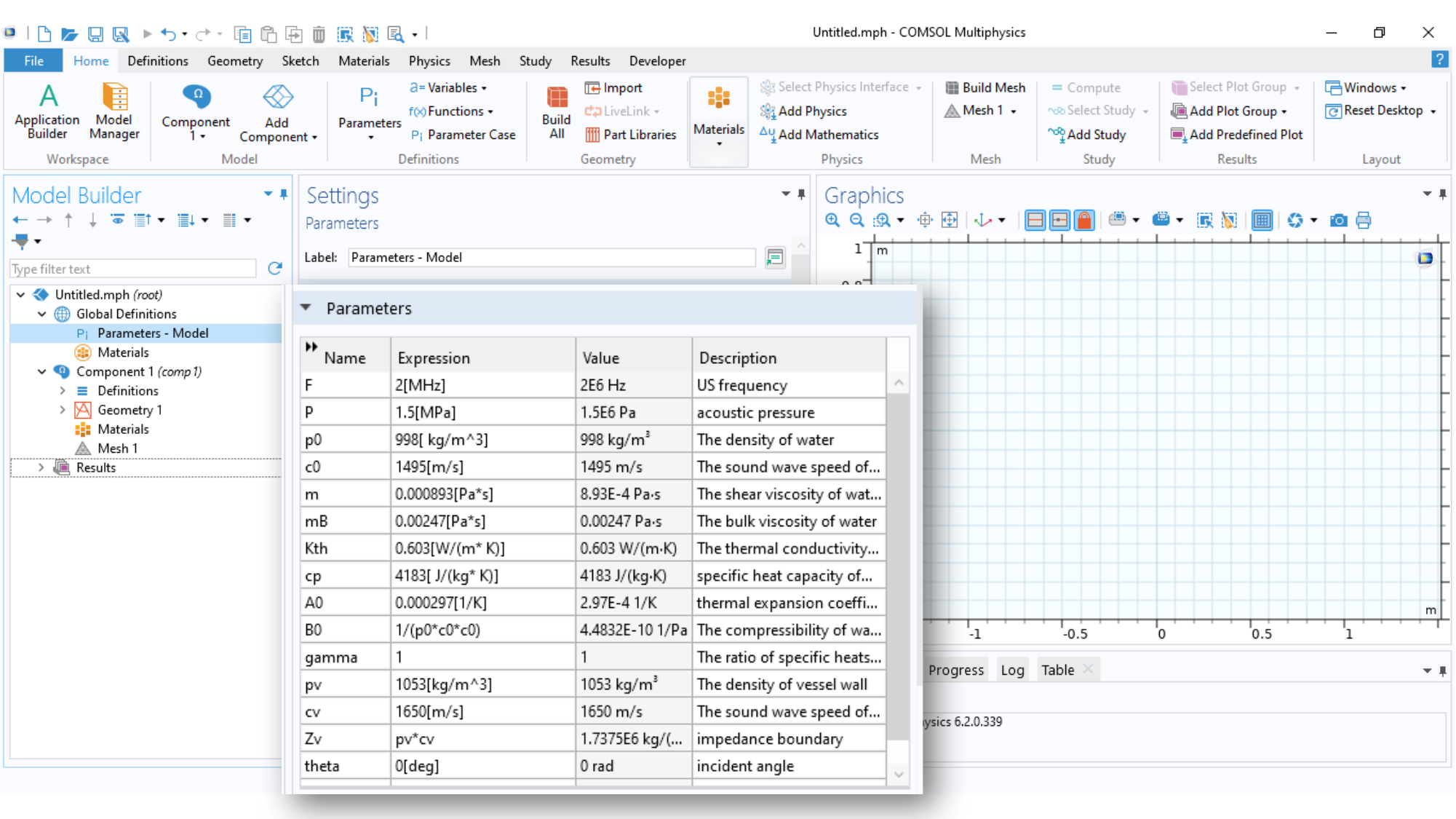

## Slide 40
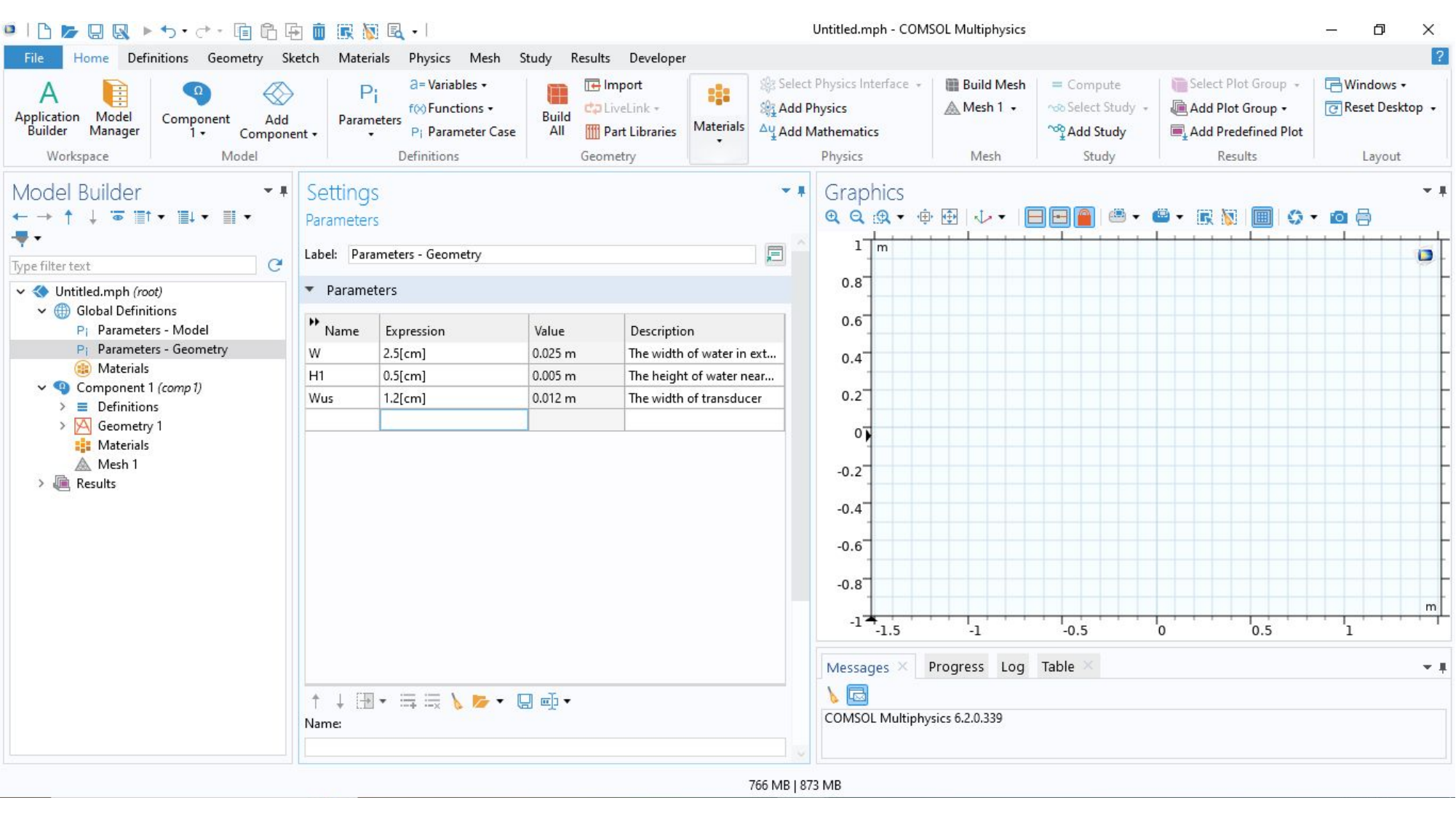

## Slide 41
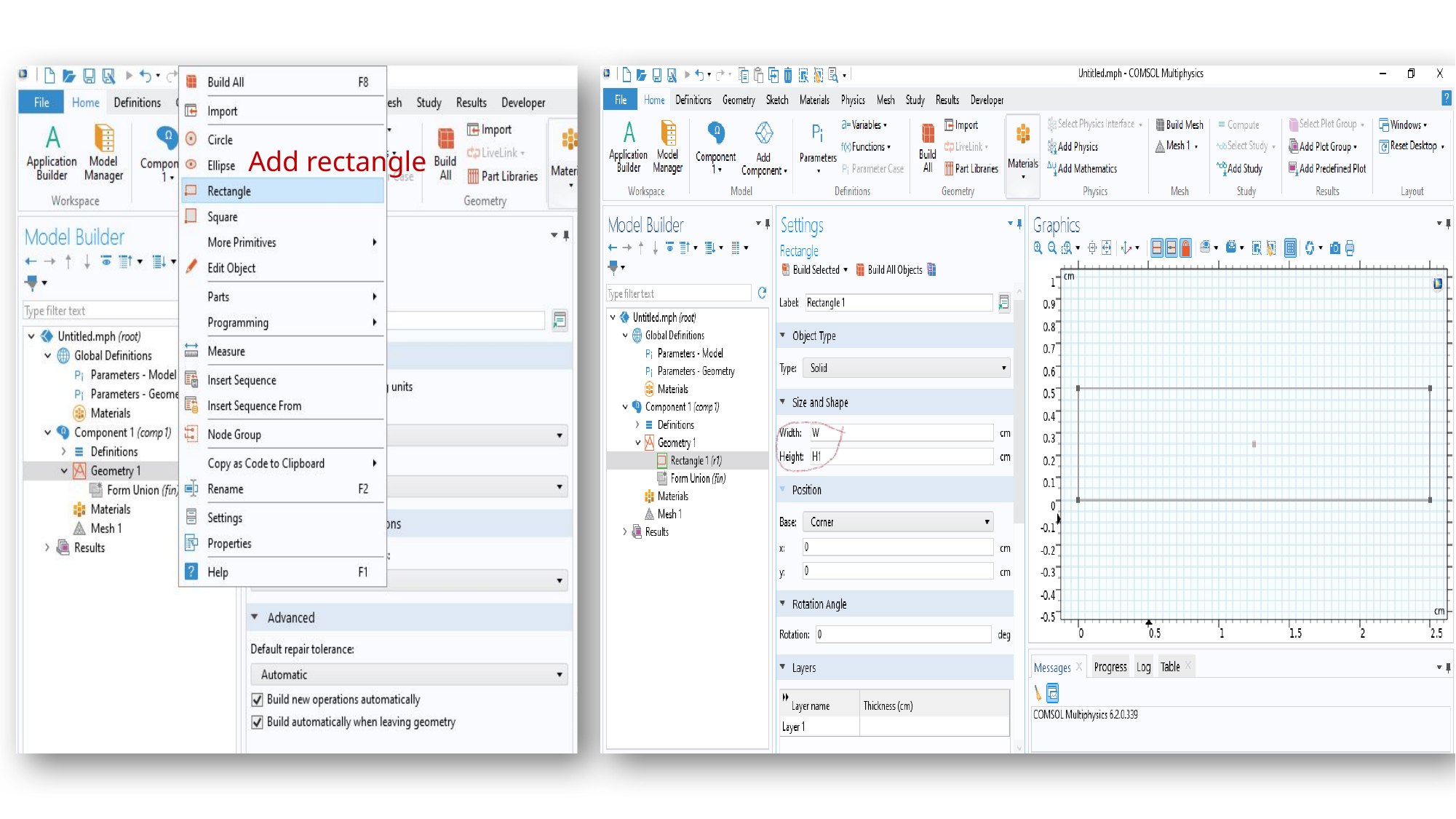

Add rectangle

## Slide 42
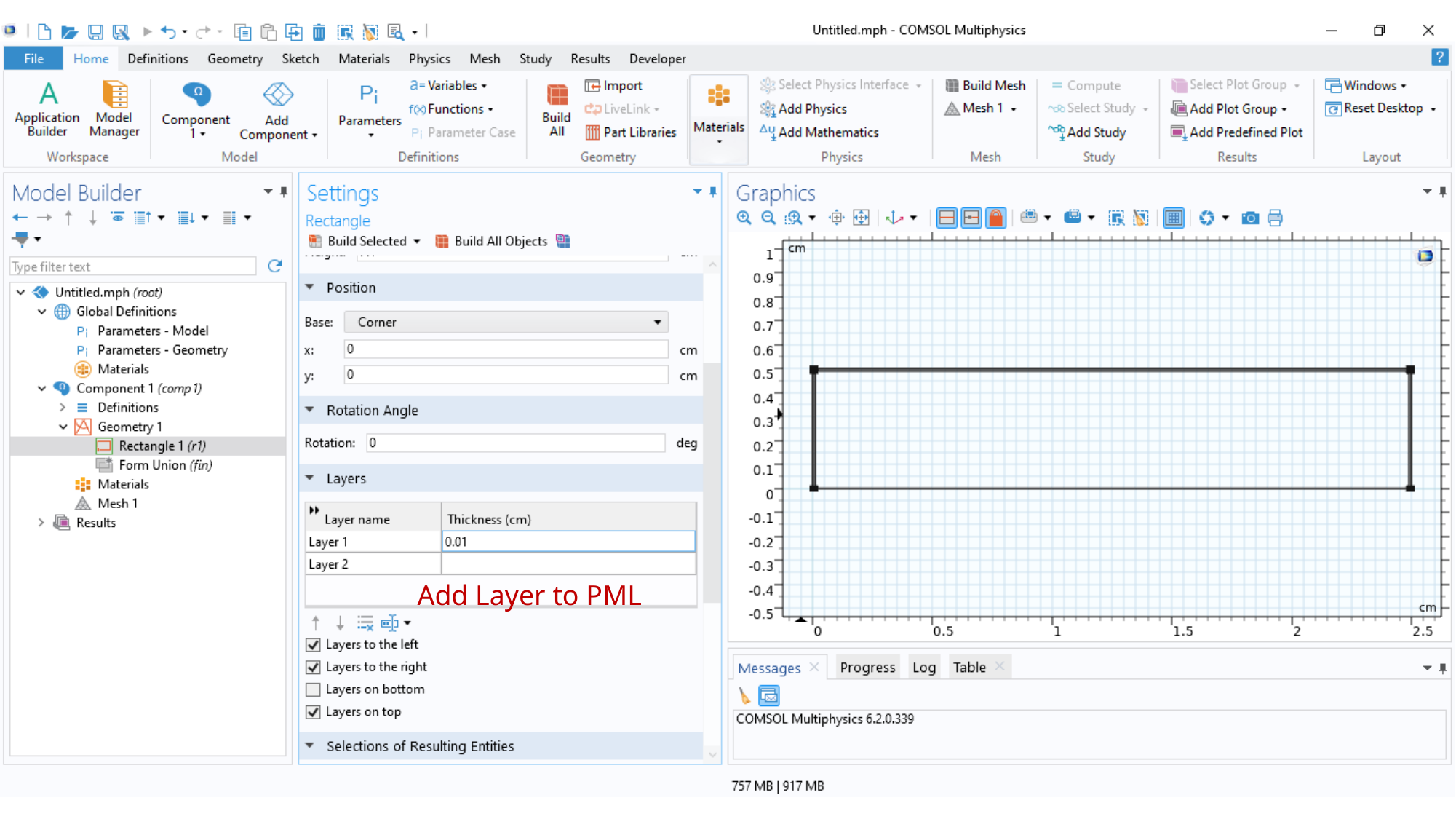

Add Layer to PML

## Slide 43
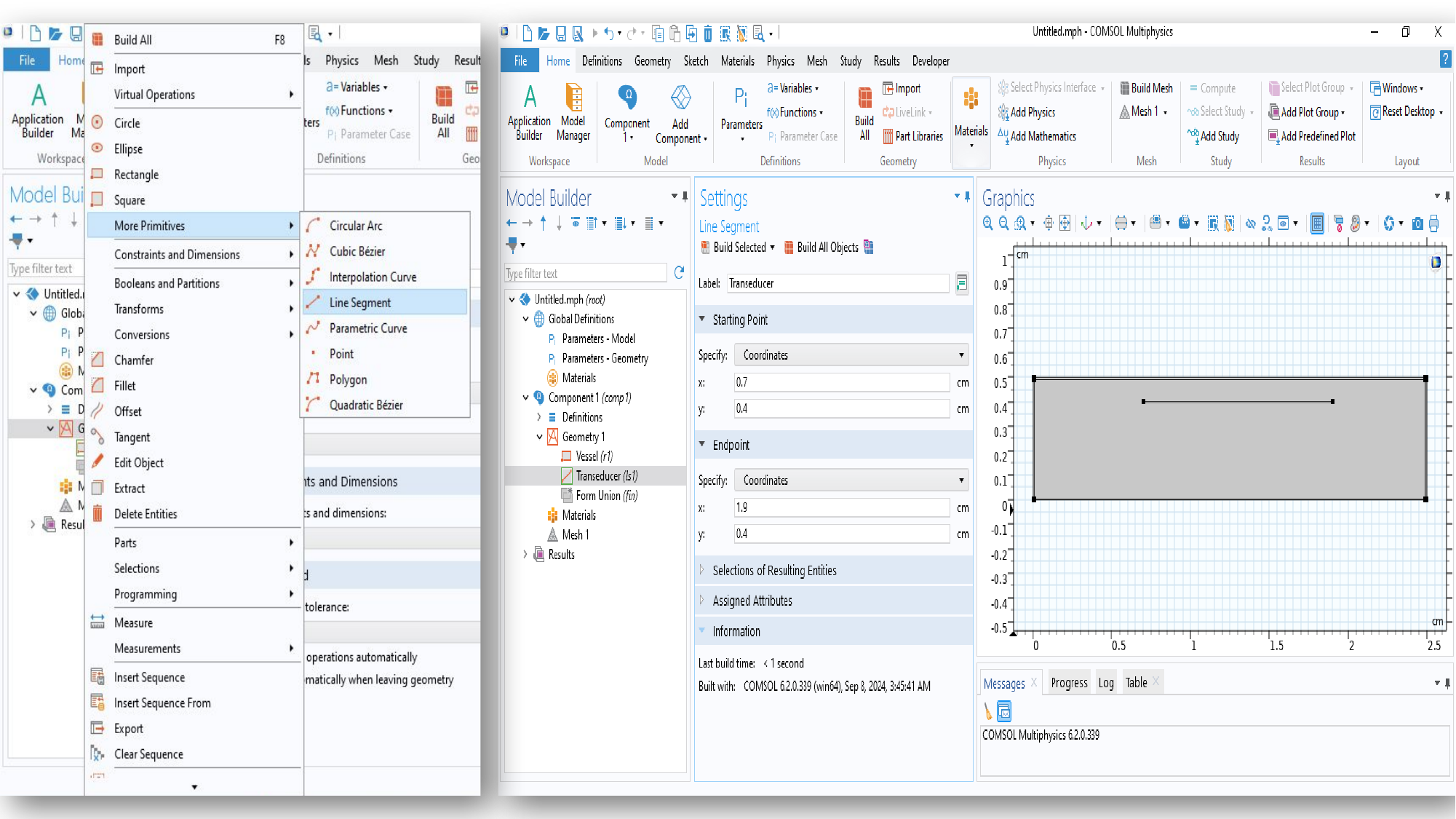

## Slide 44
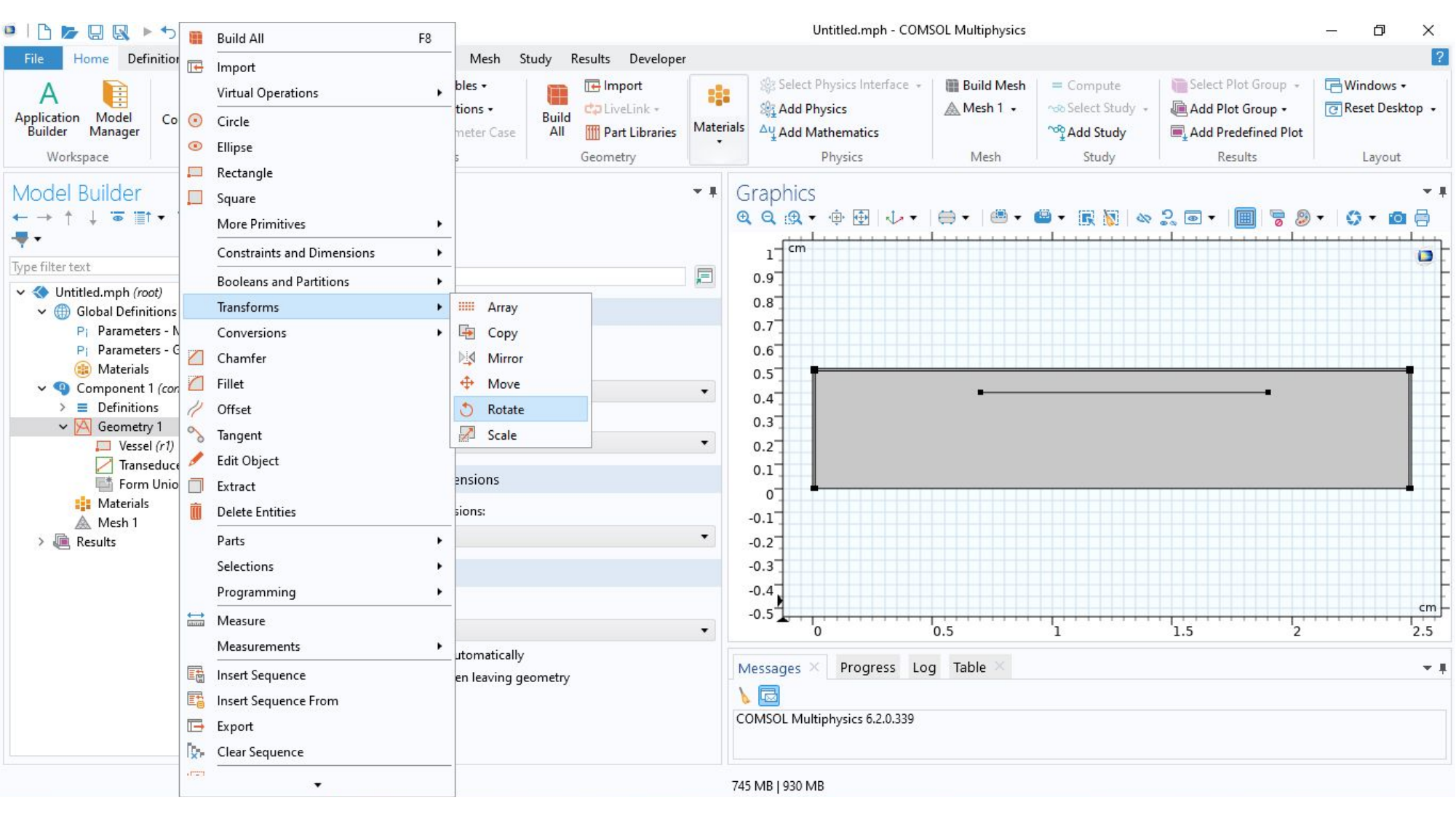

## Slide 45
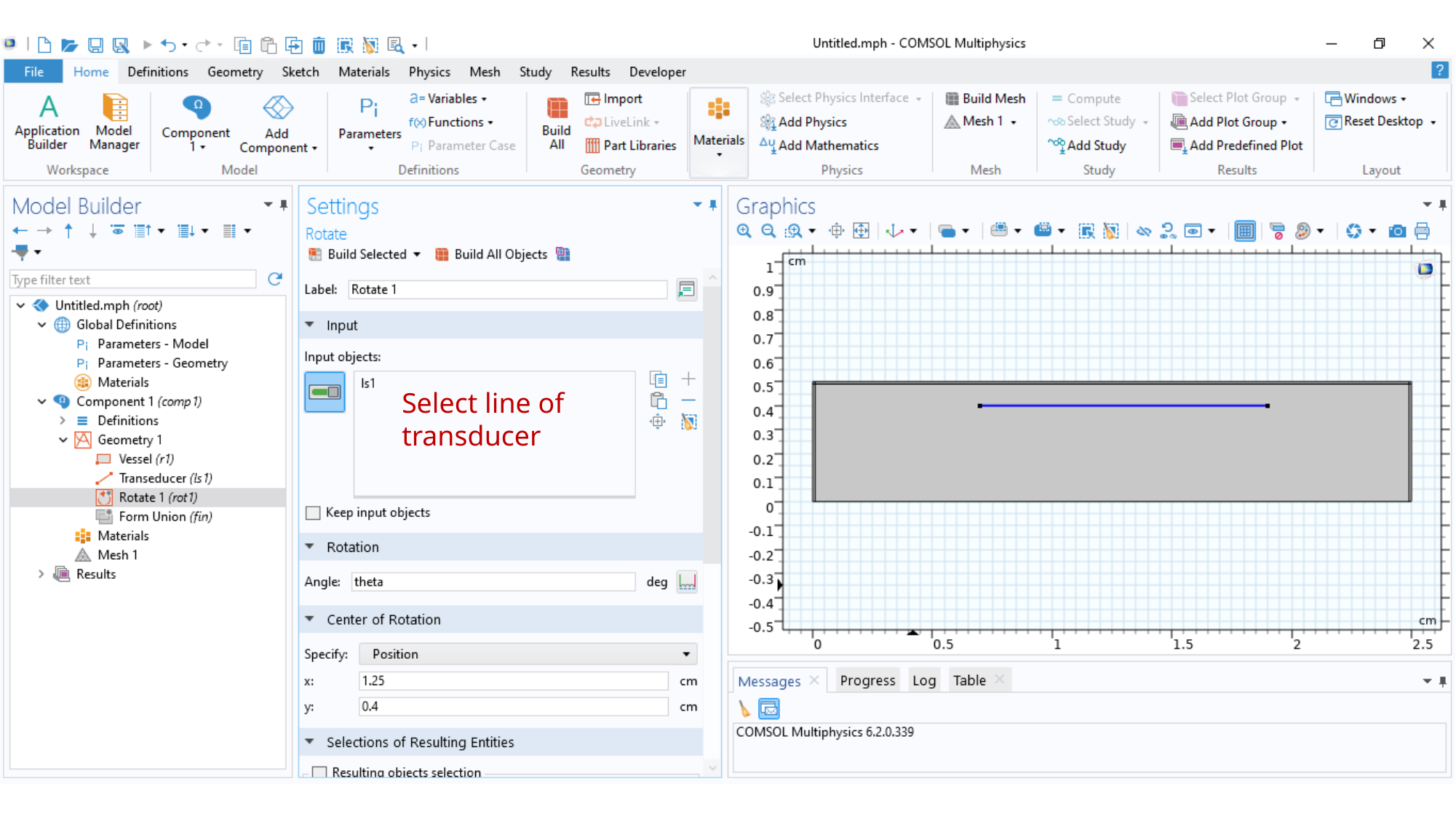

Select line of transducer

## Slide 46
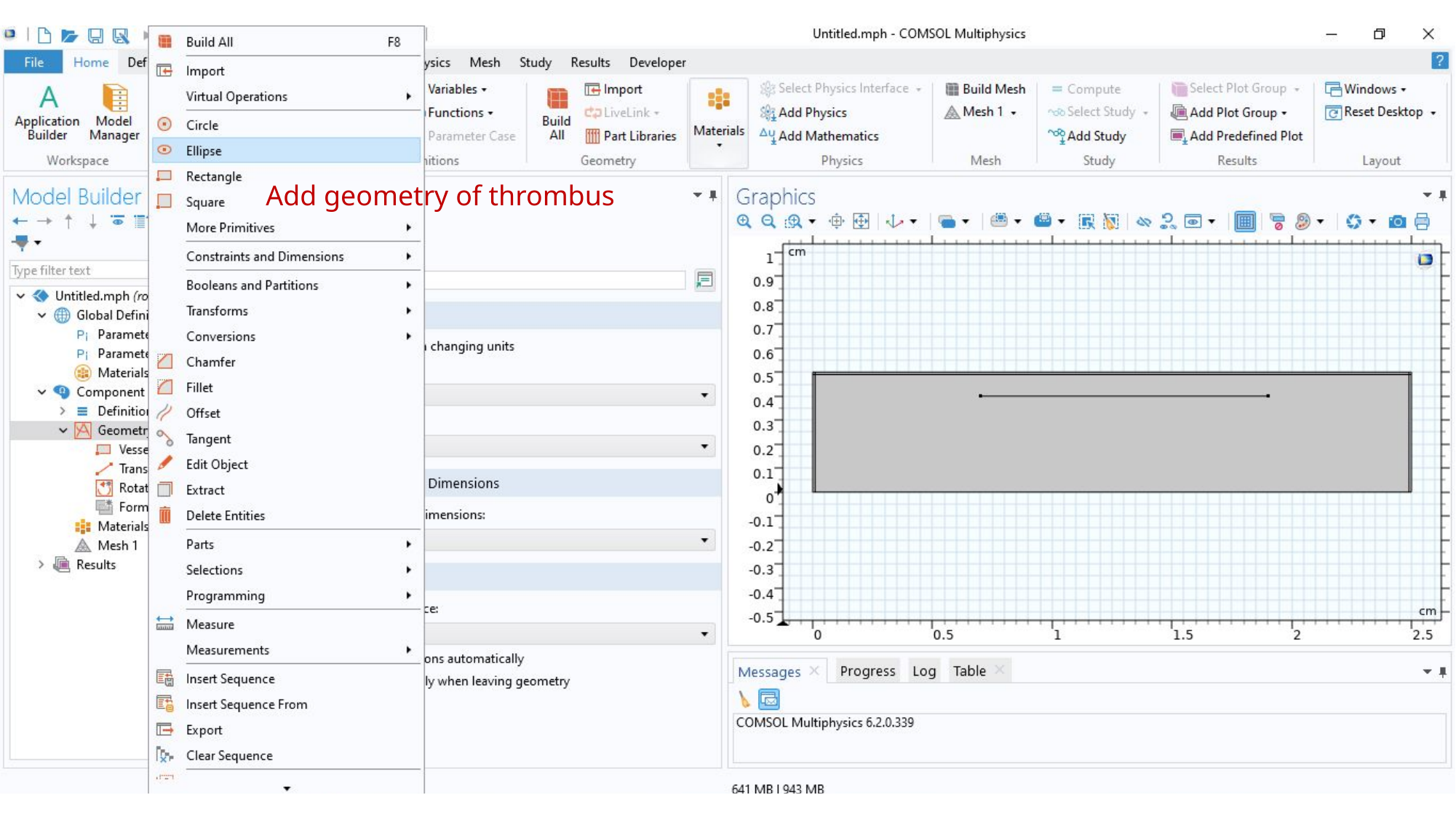

Add geometry of thrombus

## Slide 47
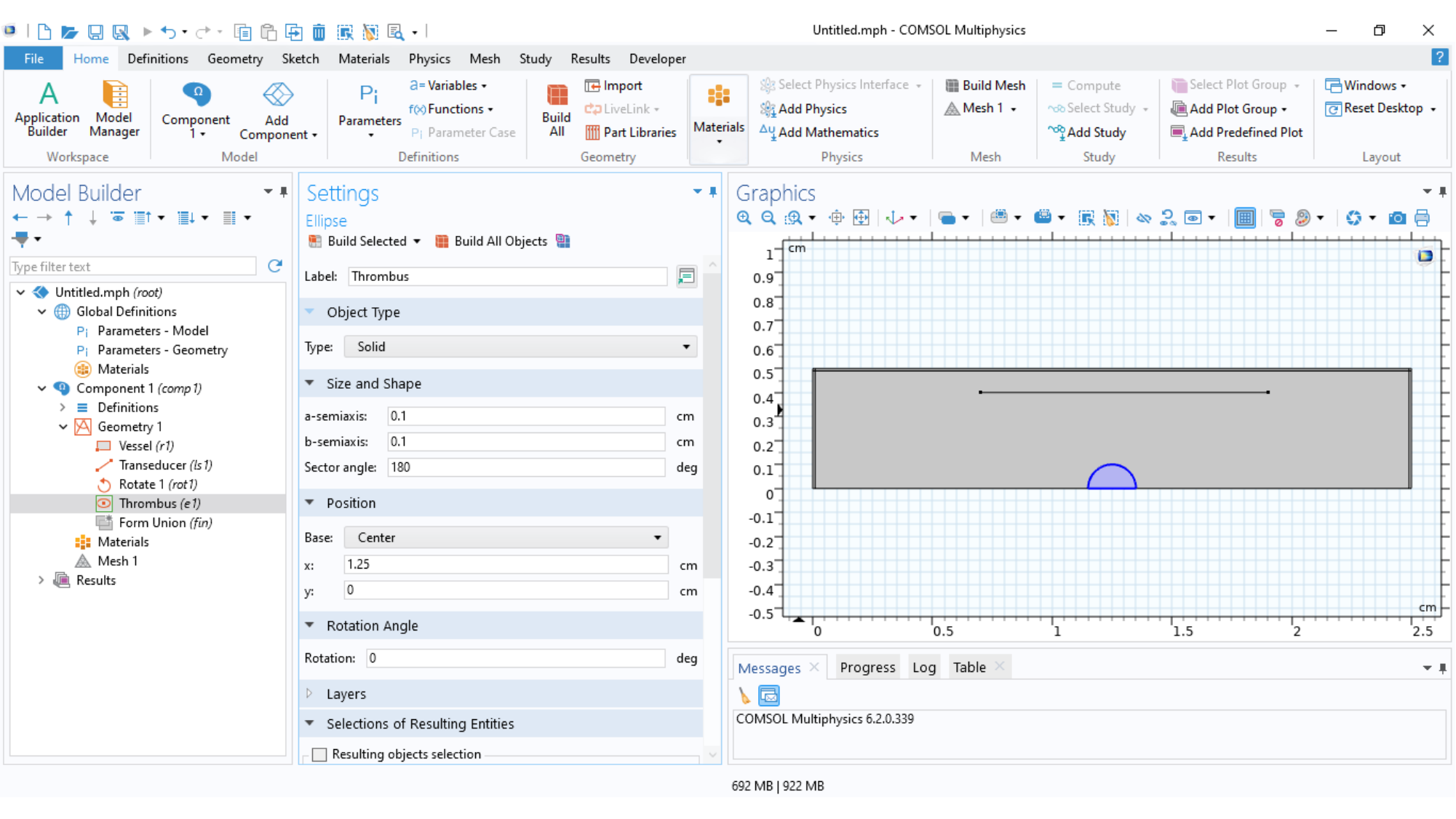

## Slide 48
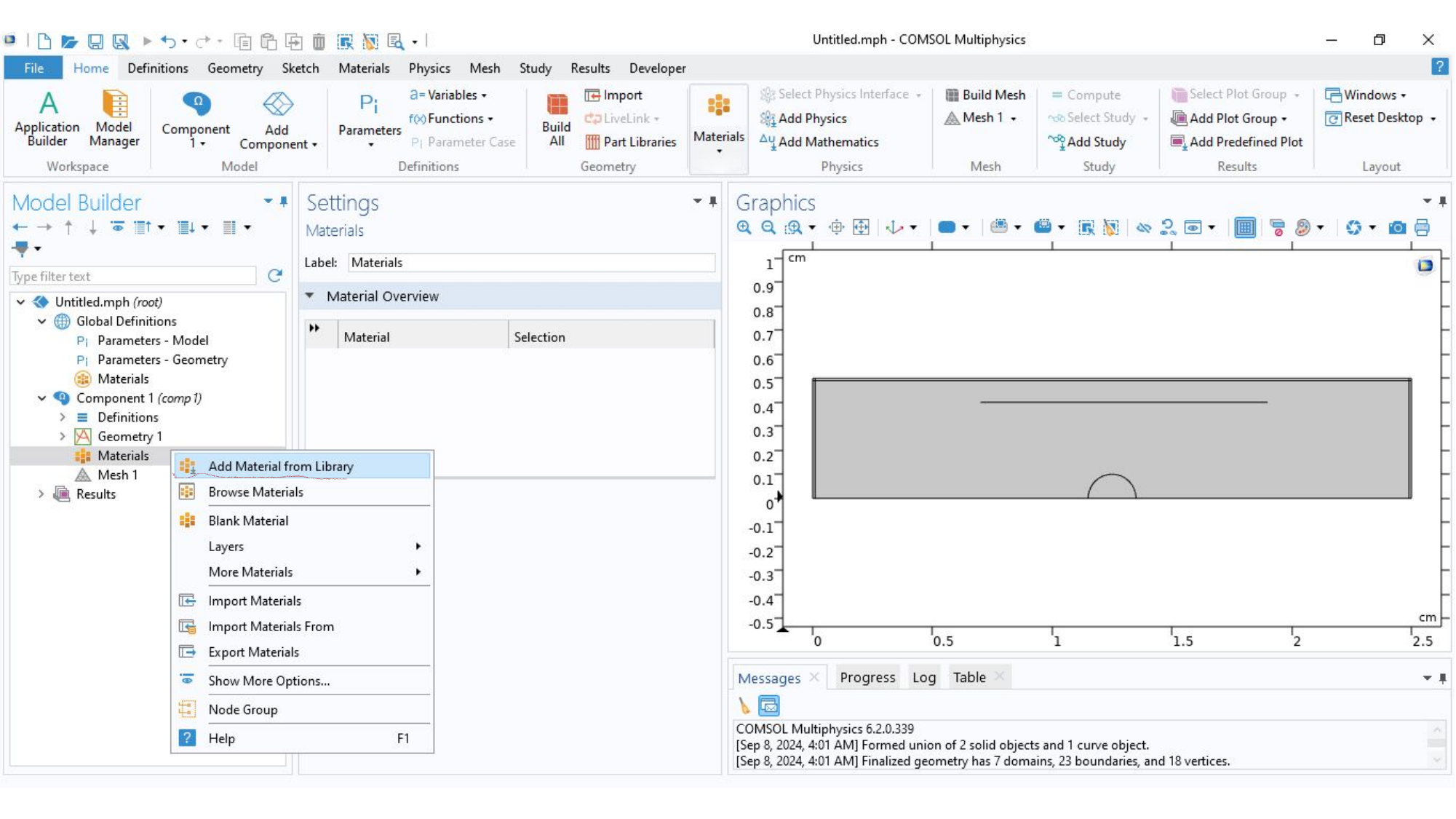

## Slide 49
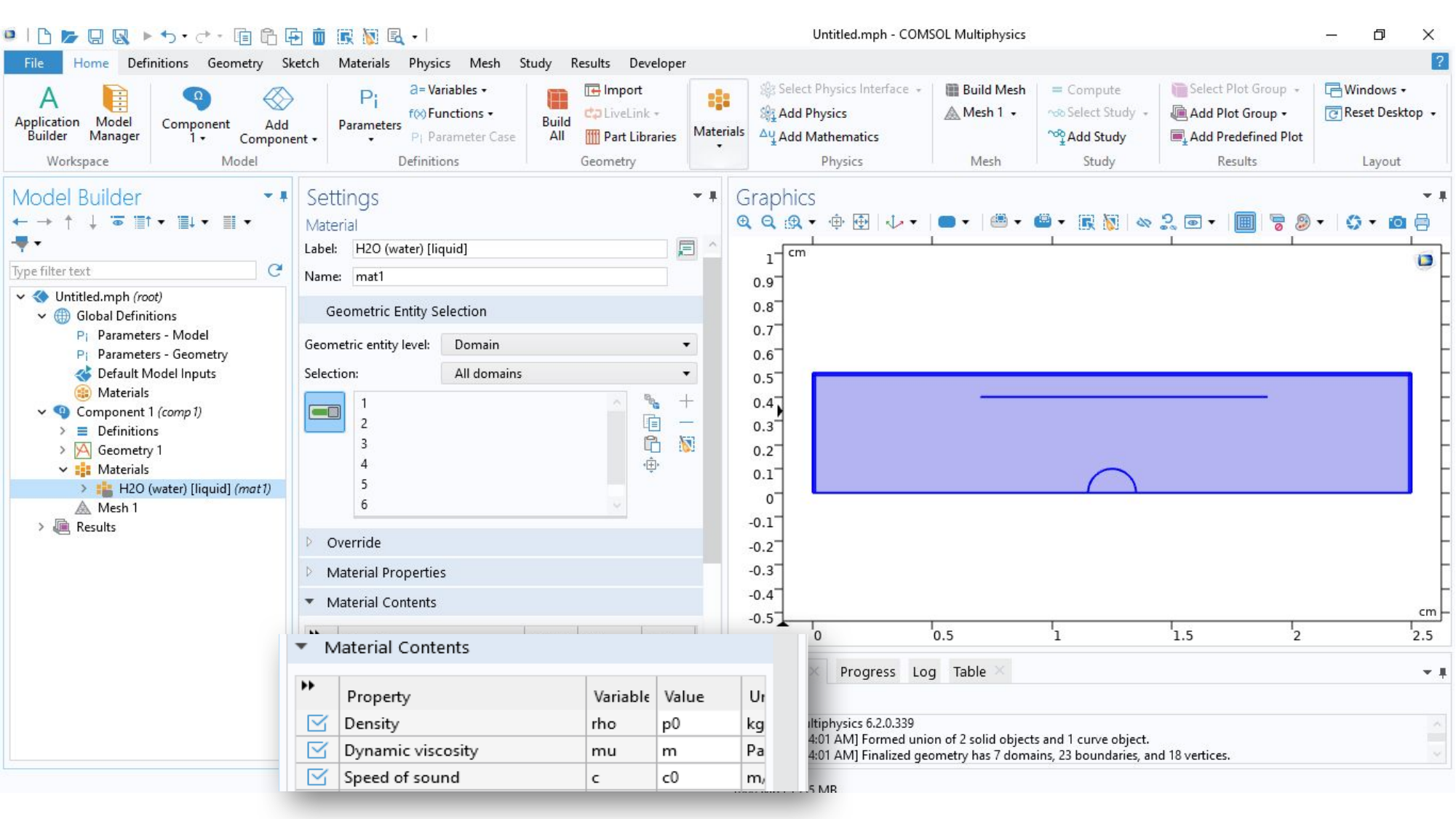

## Slide 50
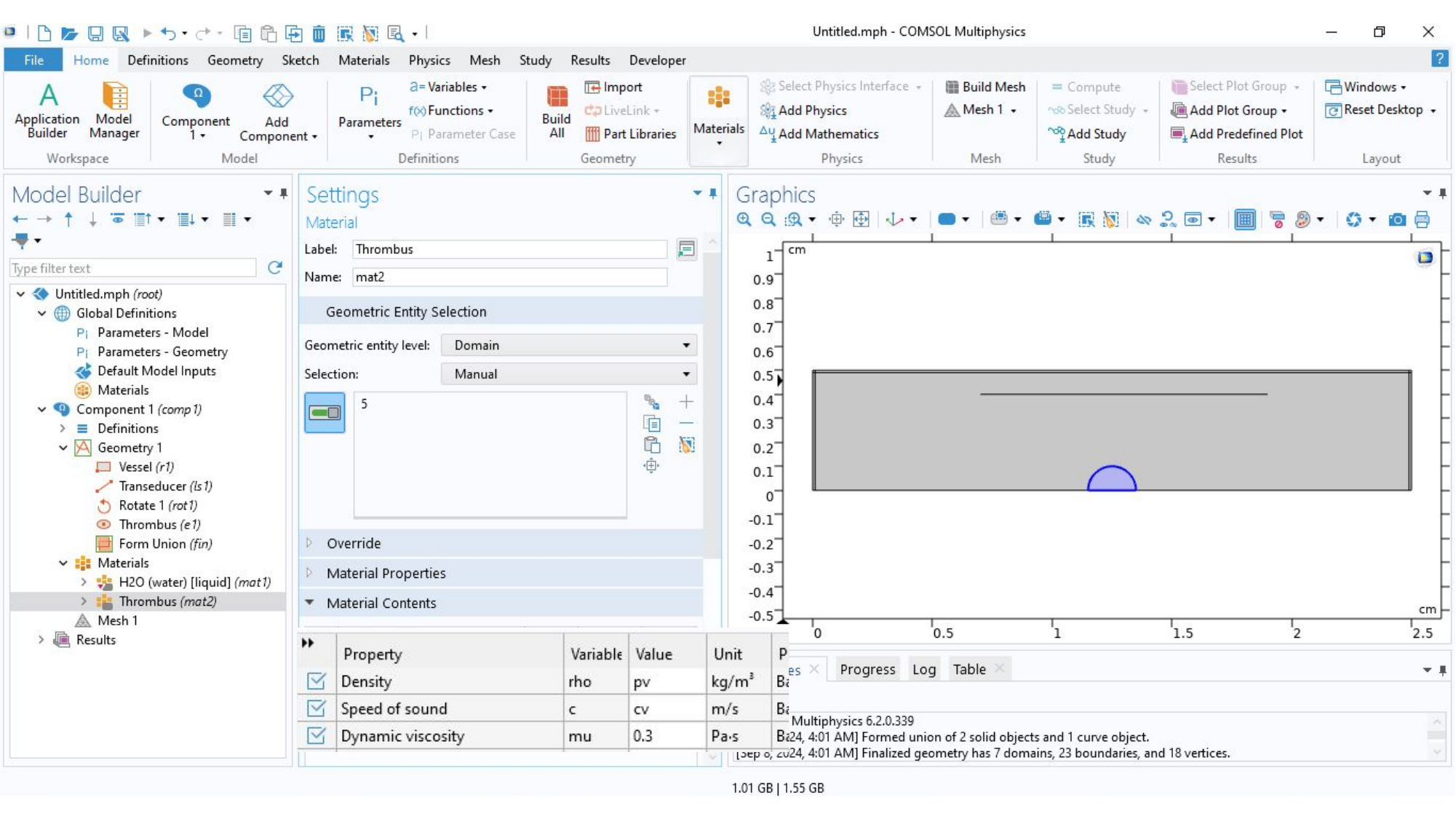

## Slide 51
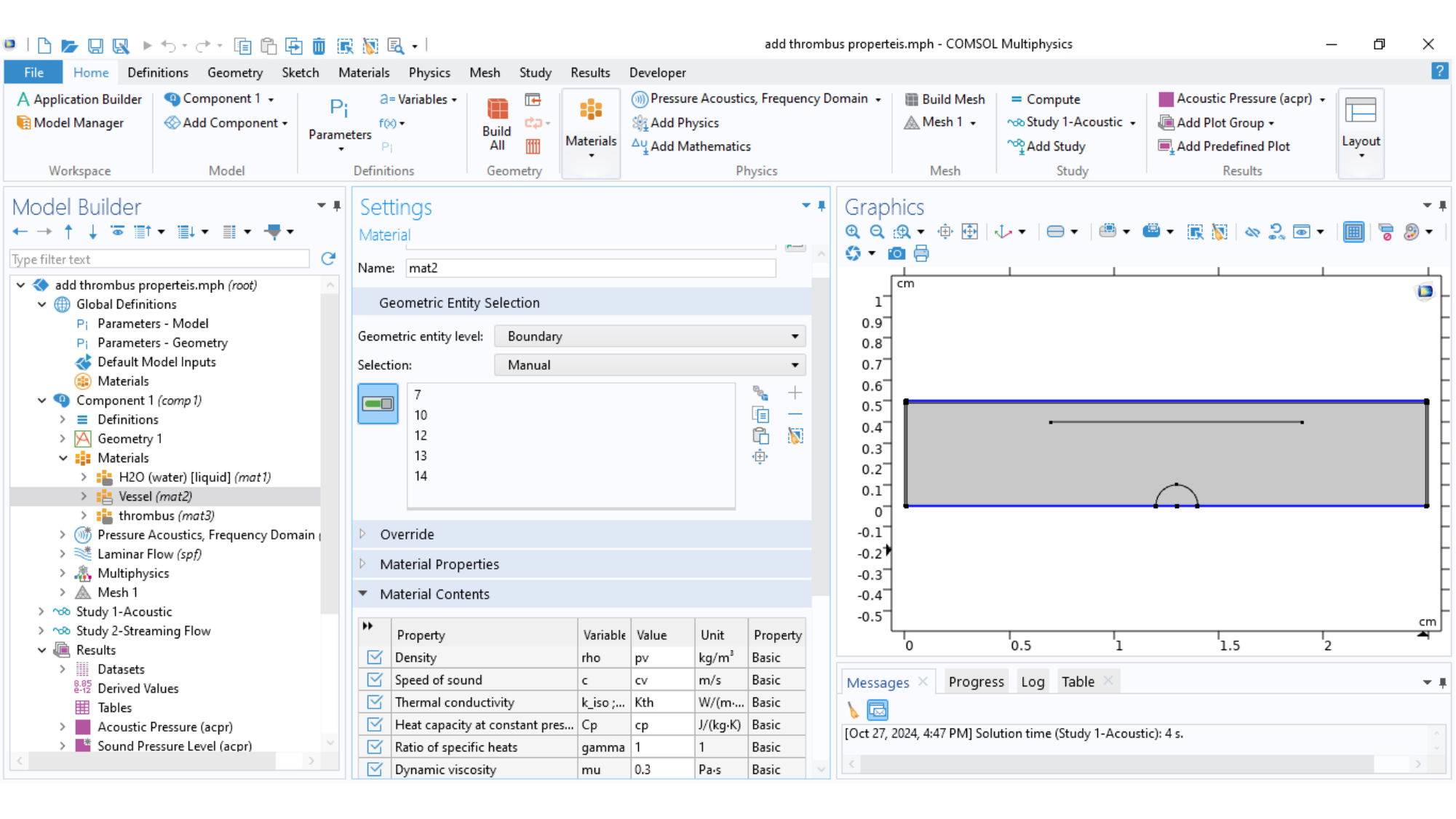

## Slide 52
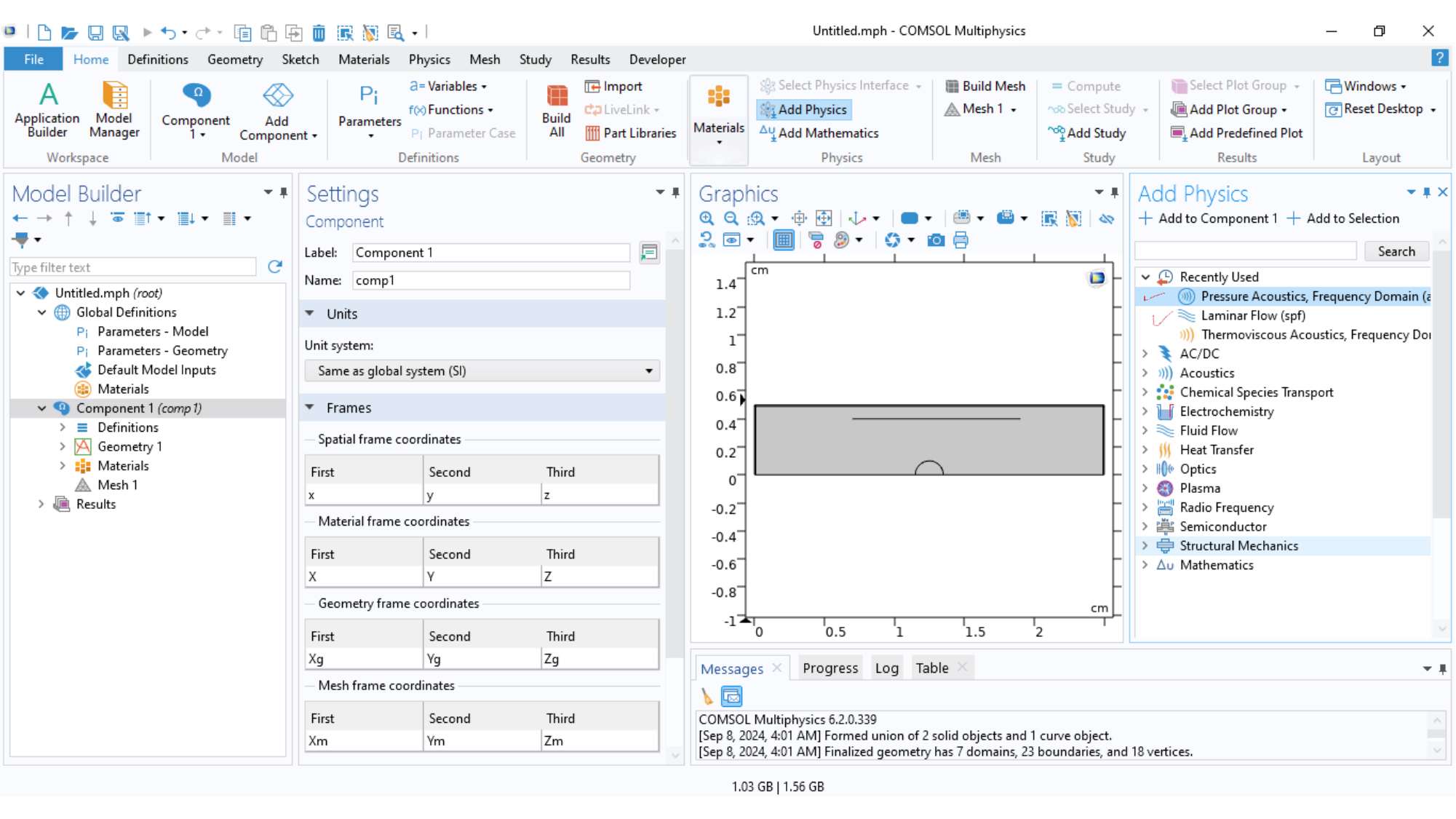

## Slide 53
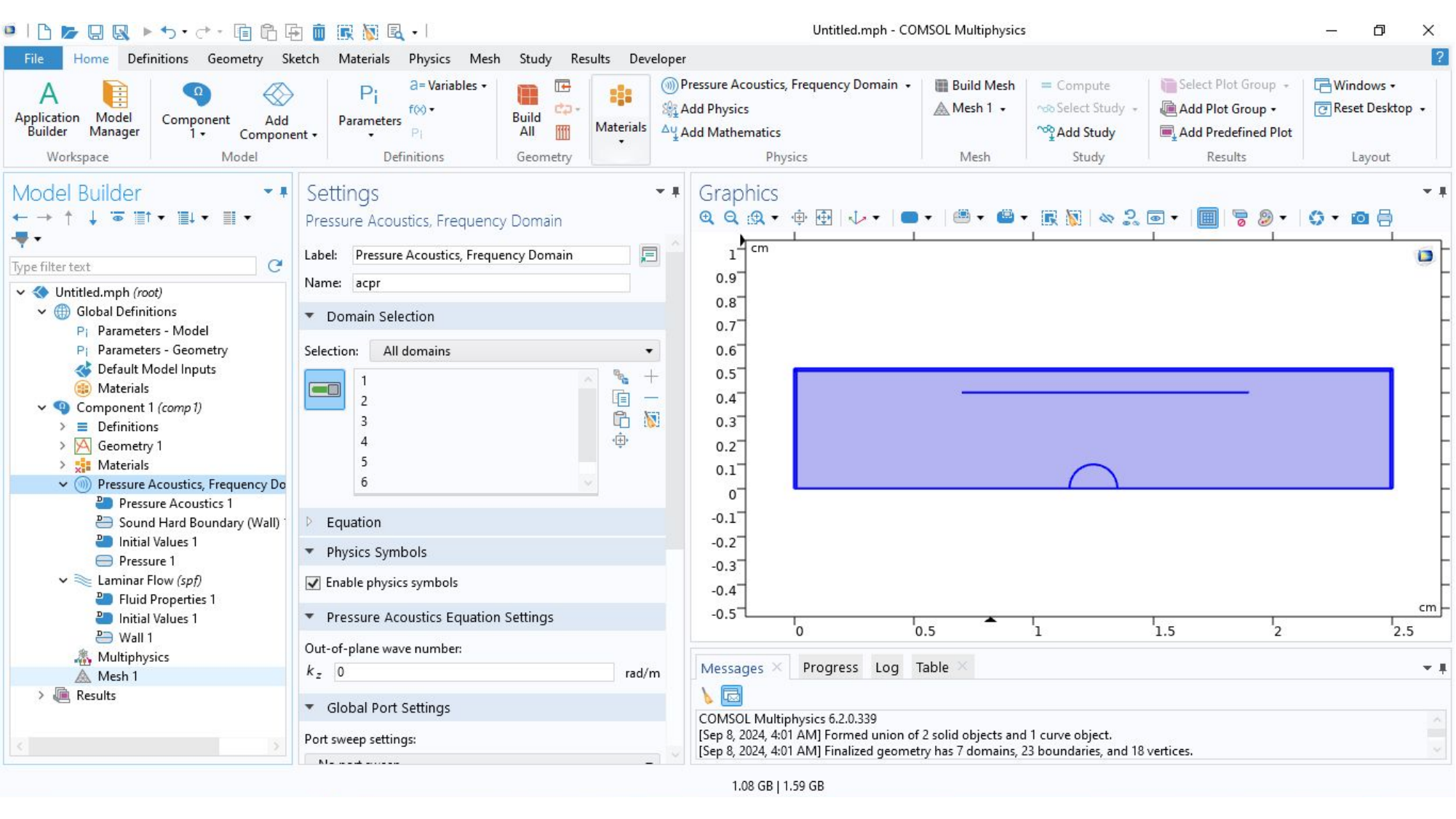

## Slide 54
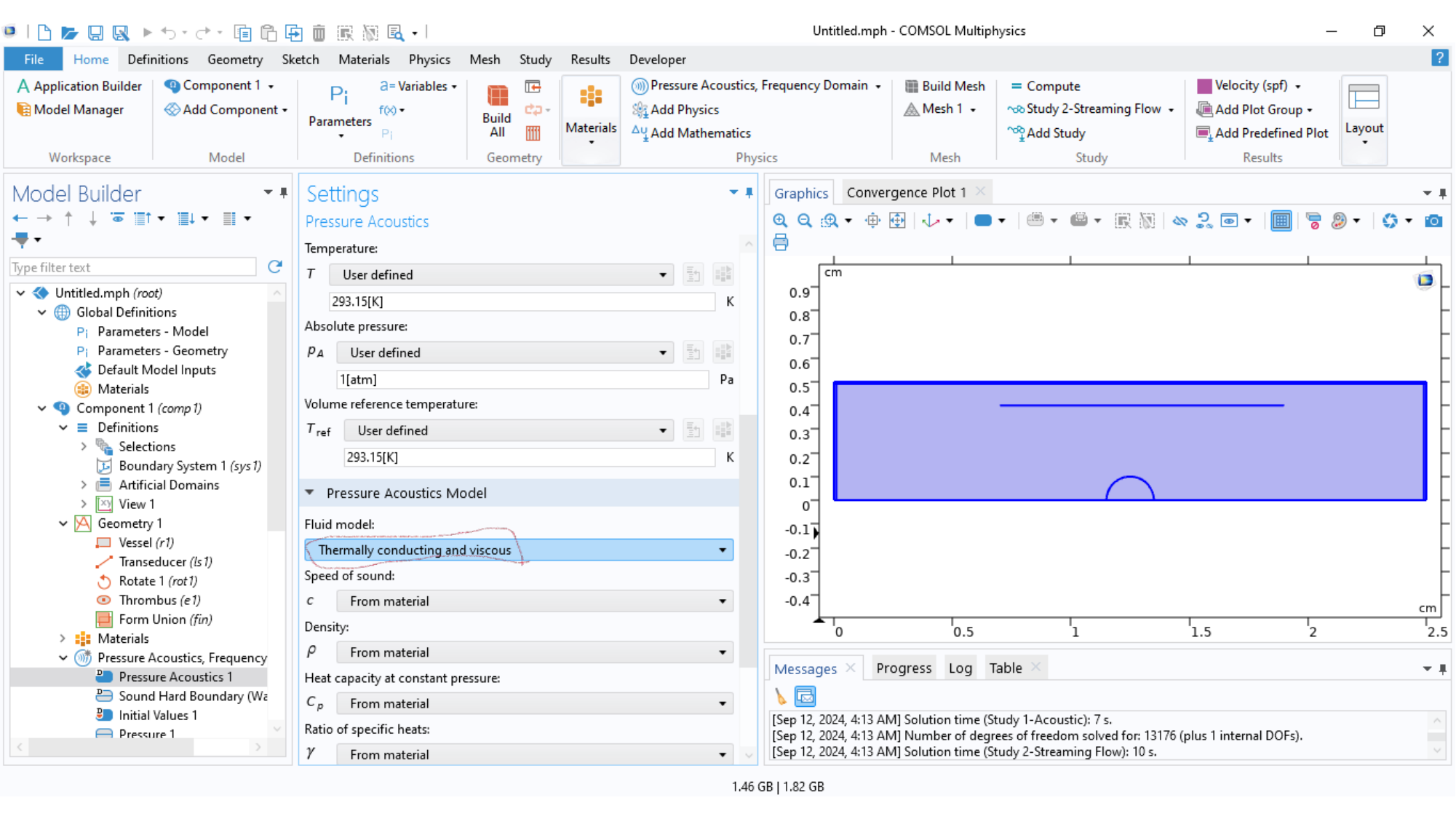

## Slide 55
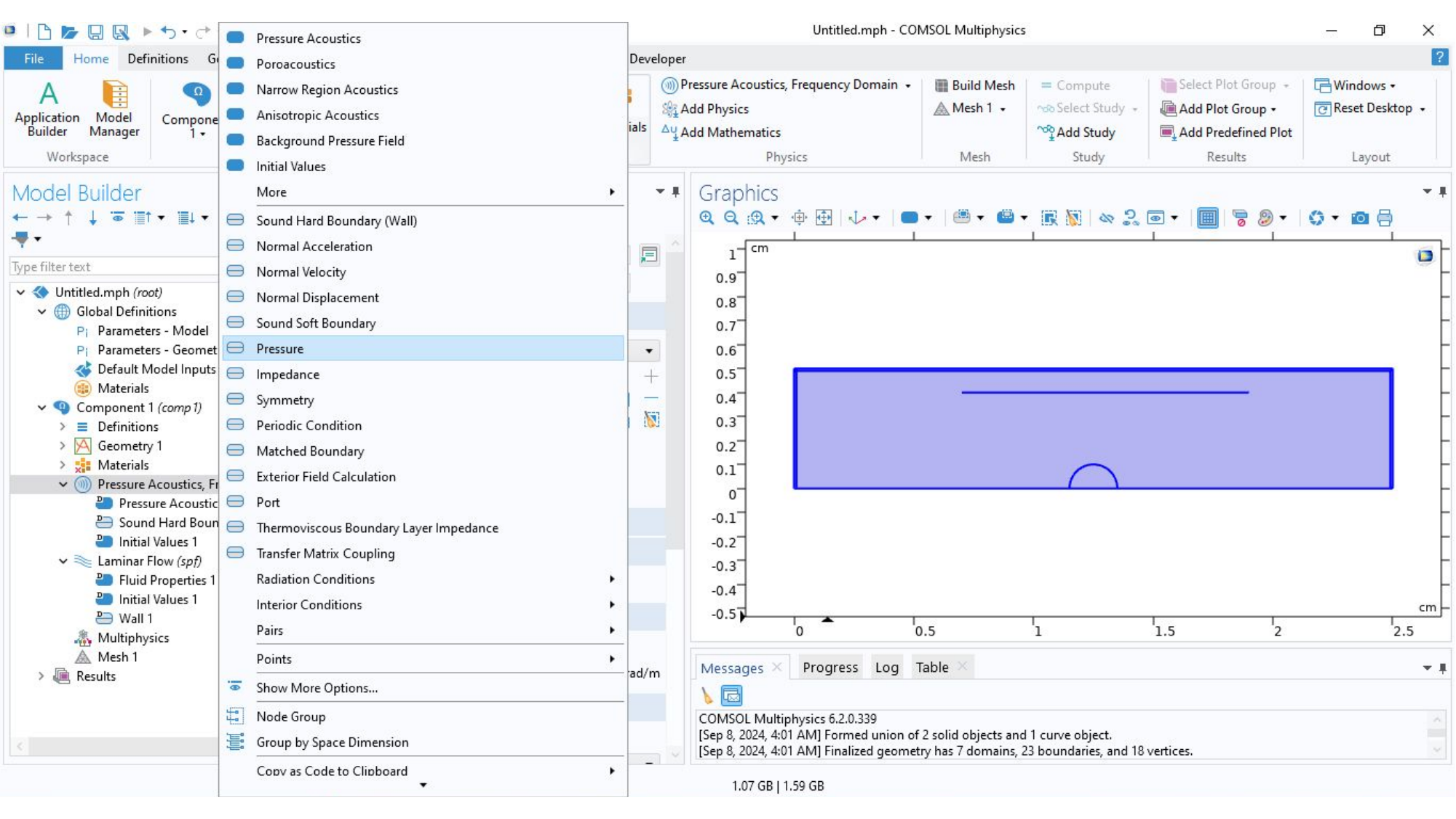

## Slide 56
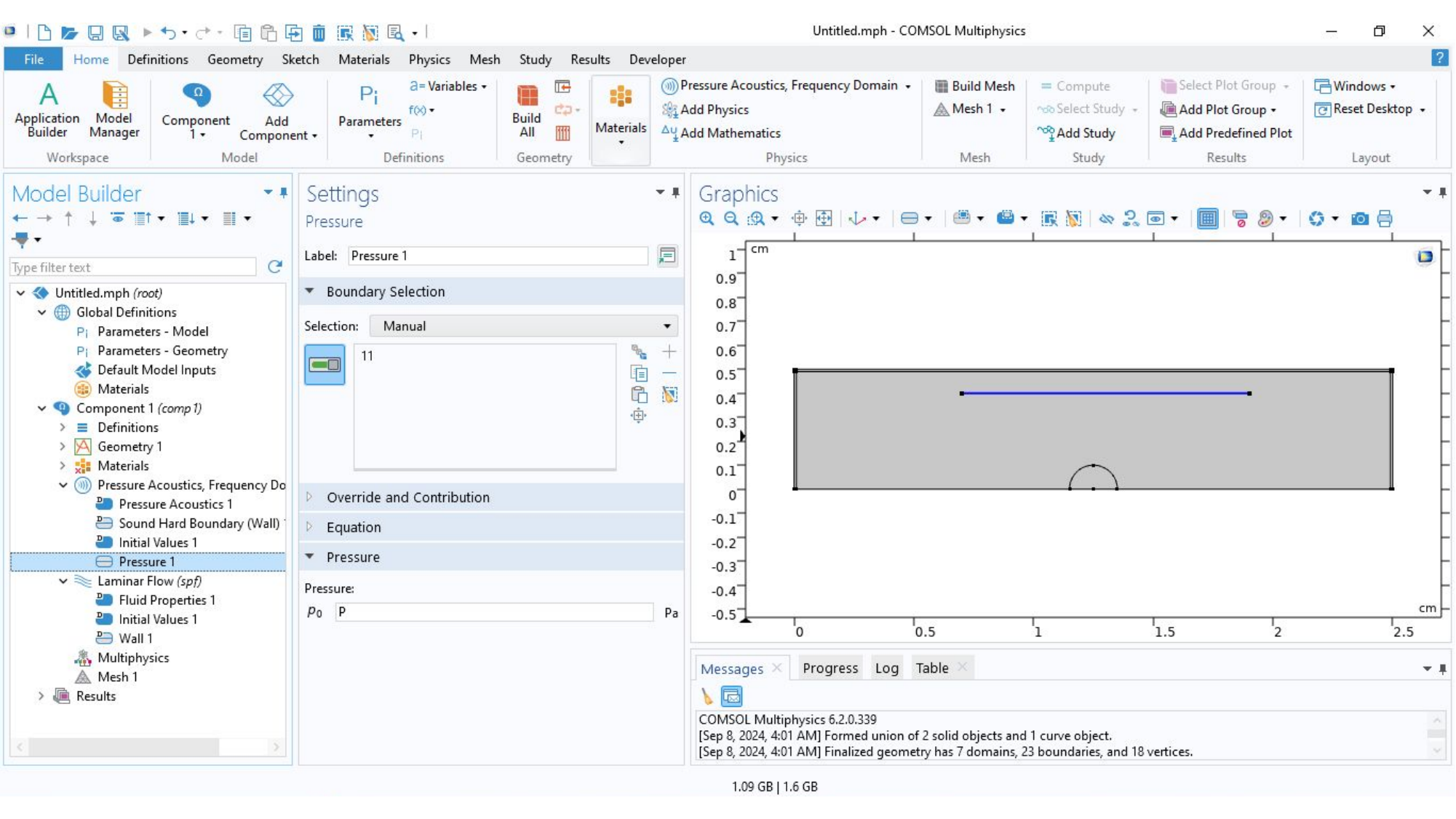

## Slide 57
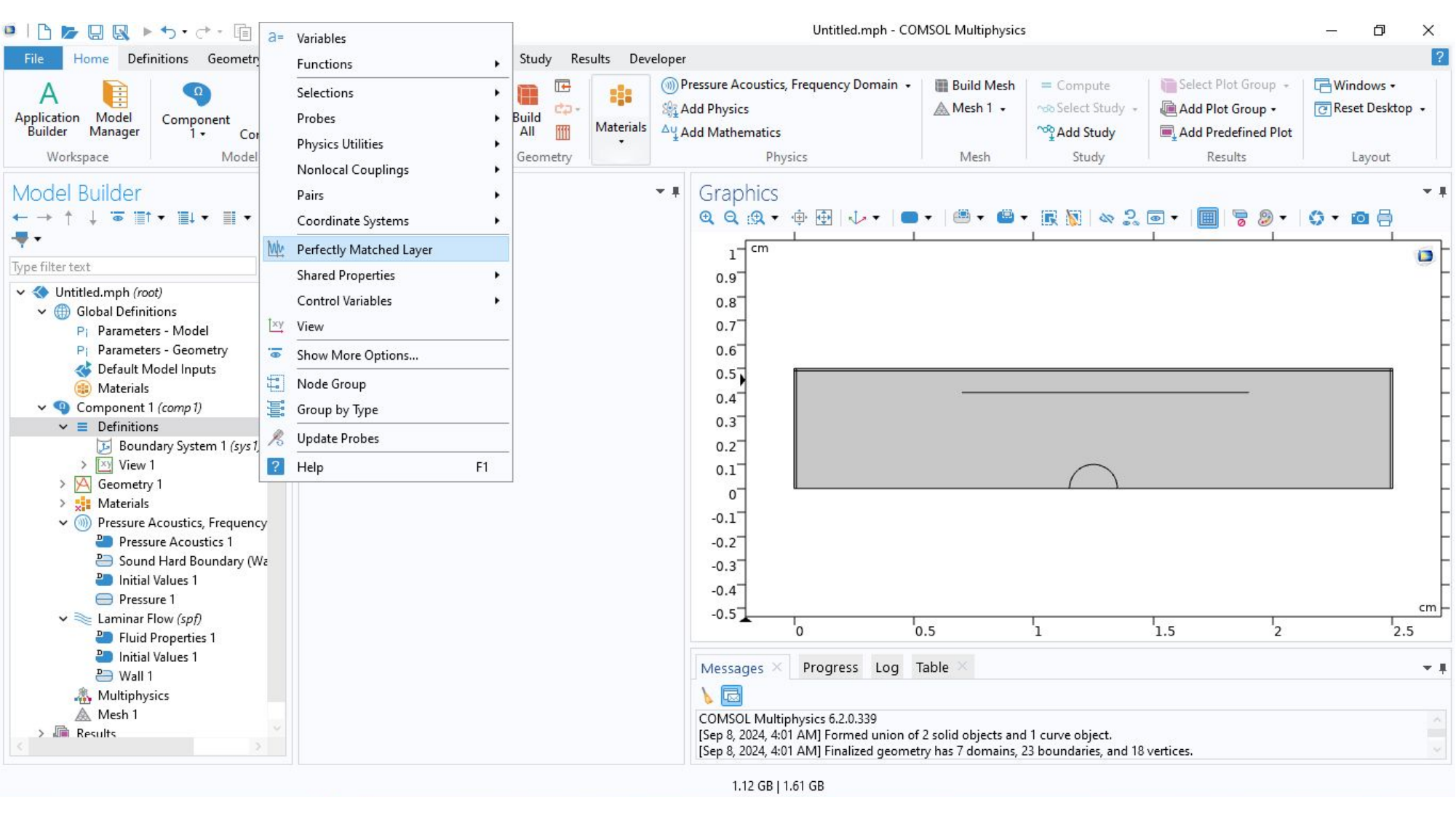

## Slide 58
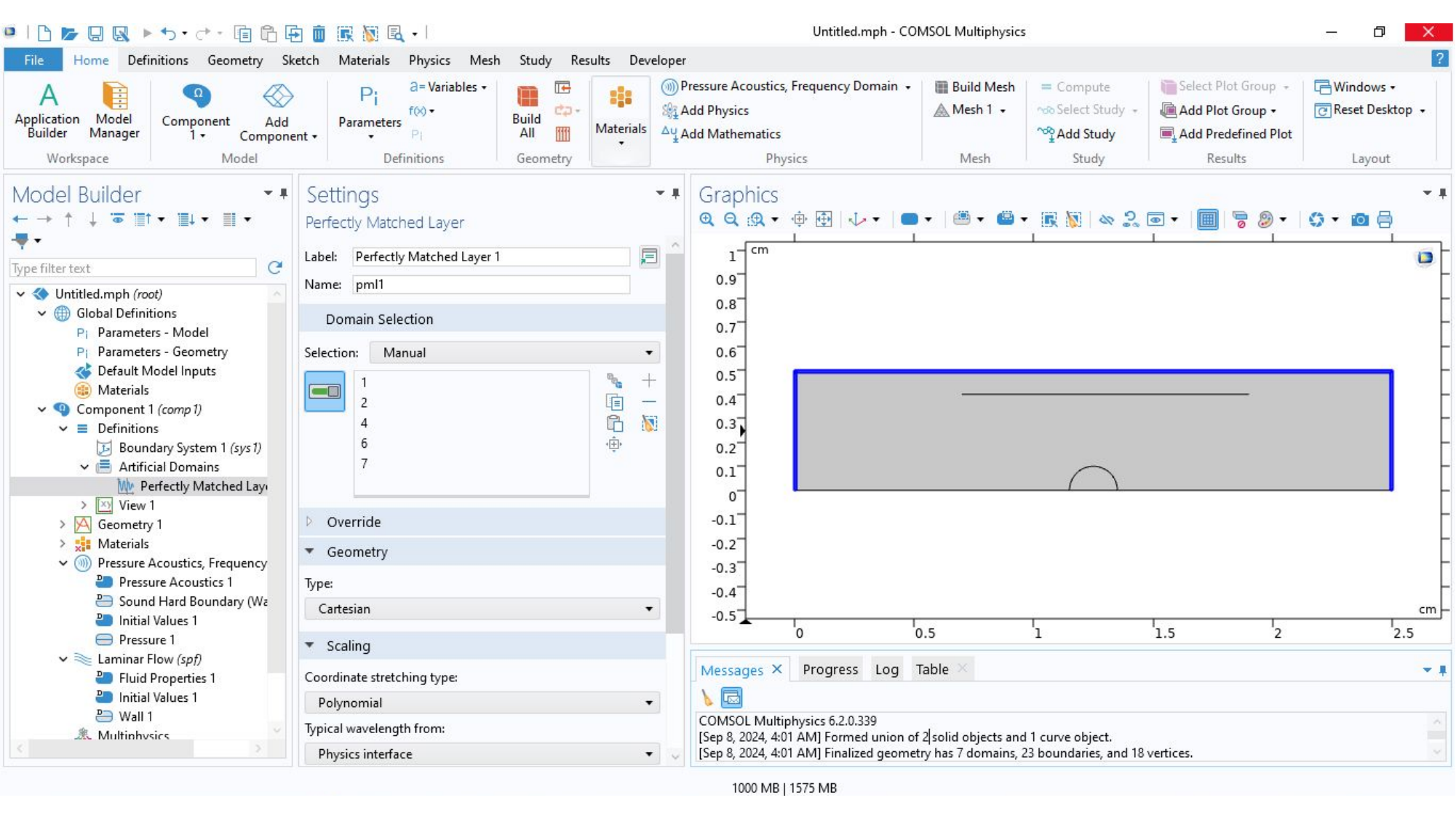

## Slide 59
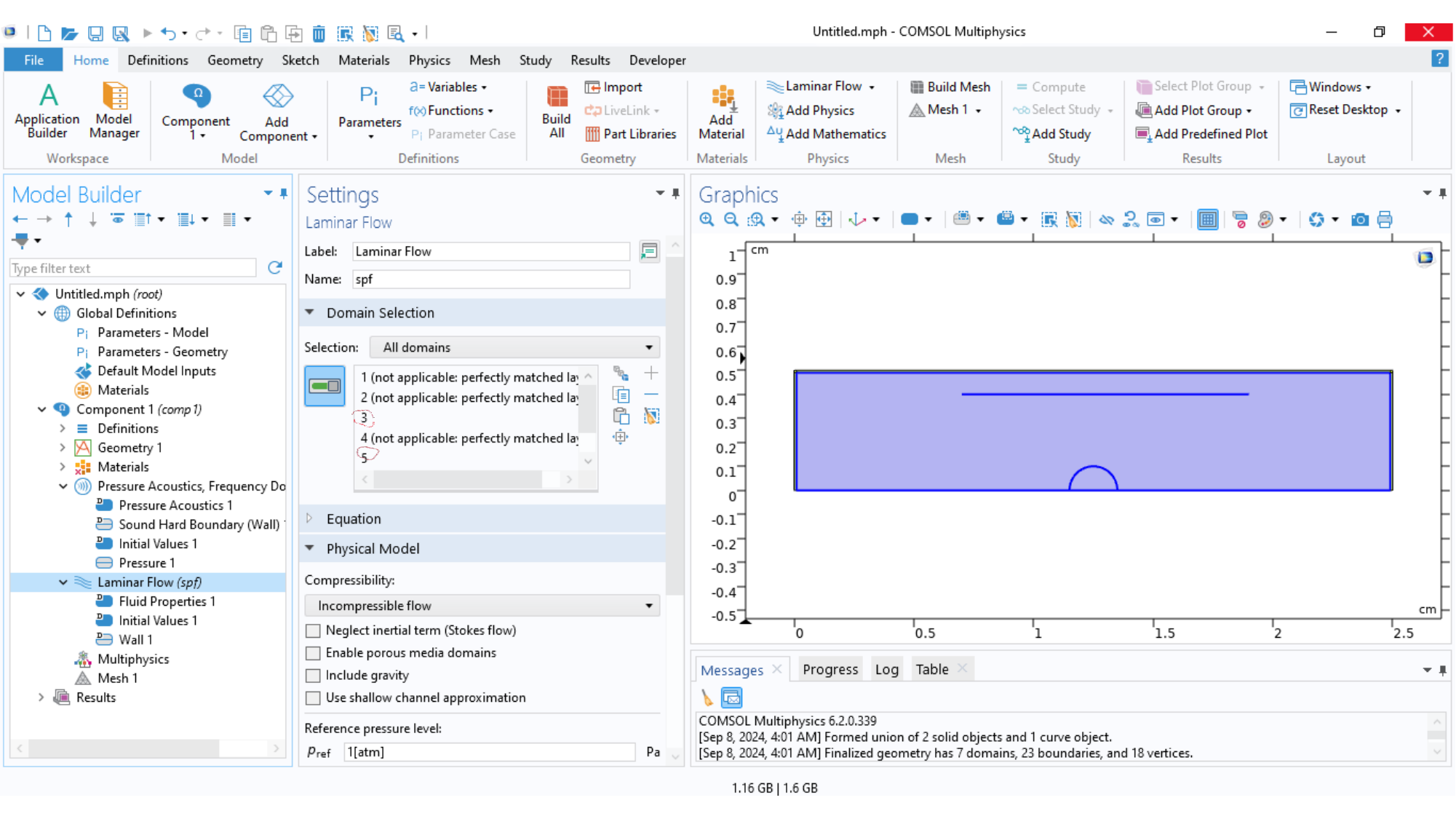

## Slide 60
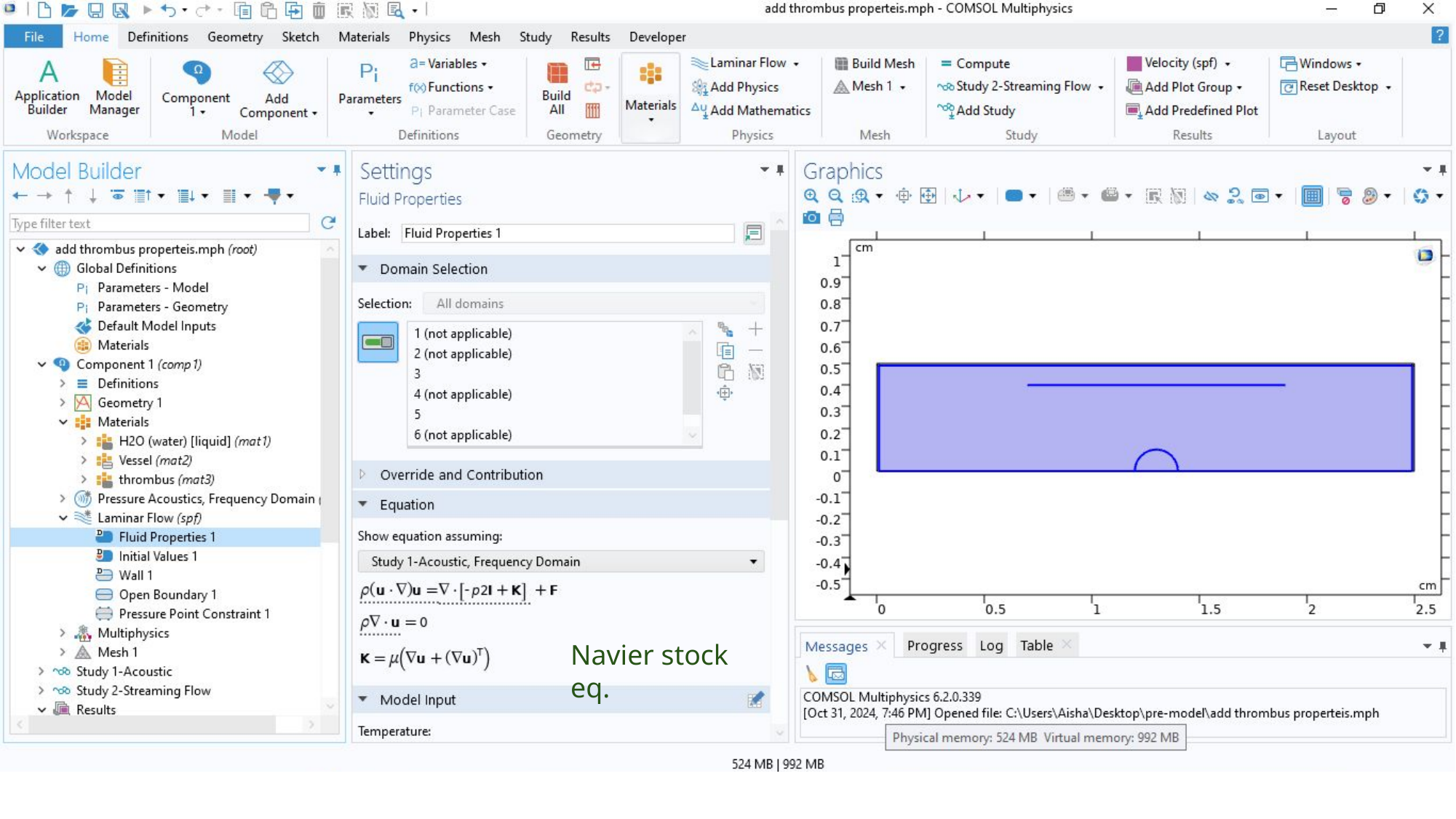

Navier stock eq.

## Slide 61
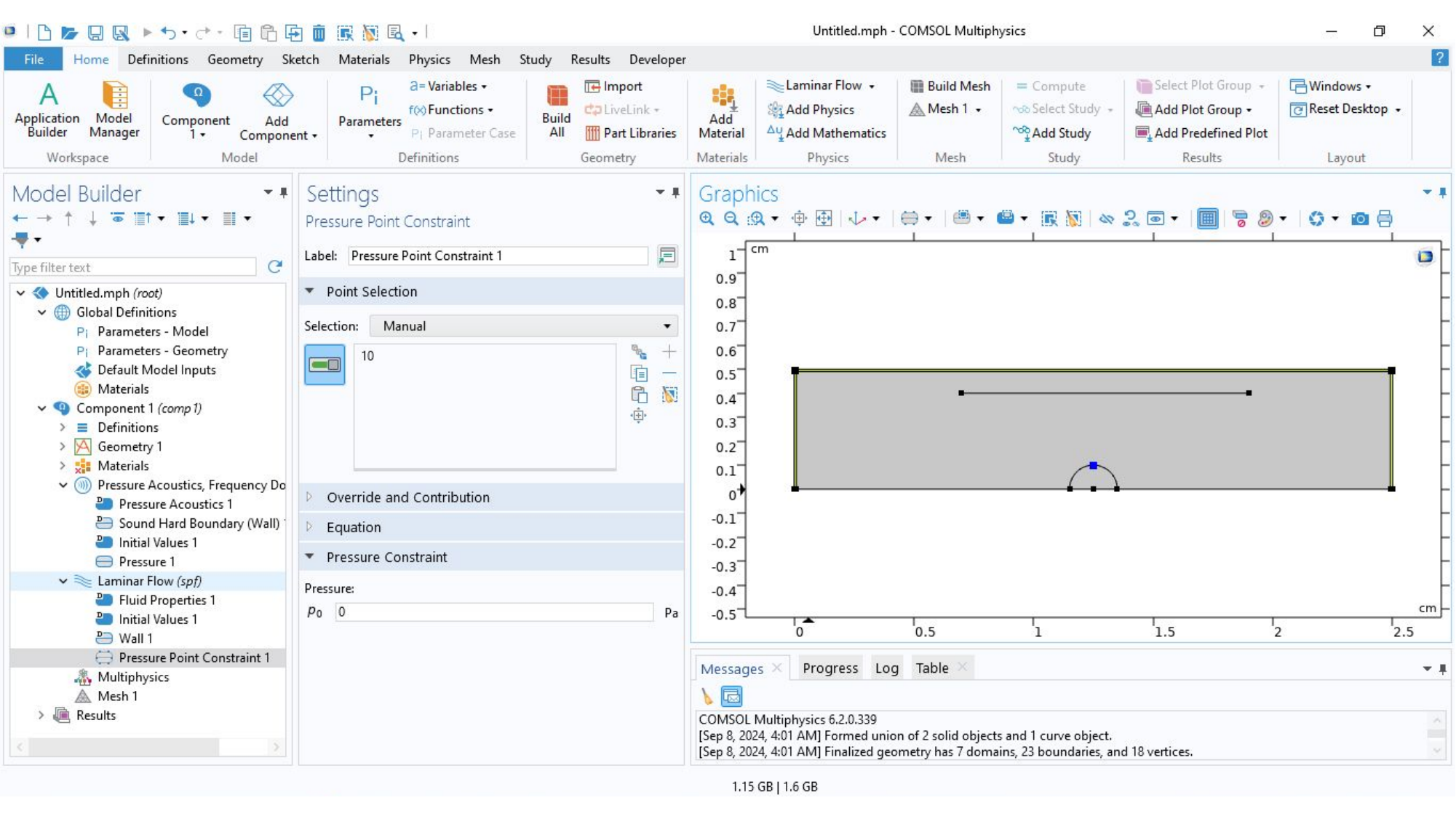

## Slide 62
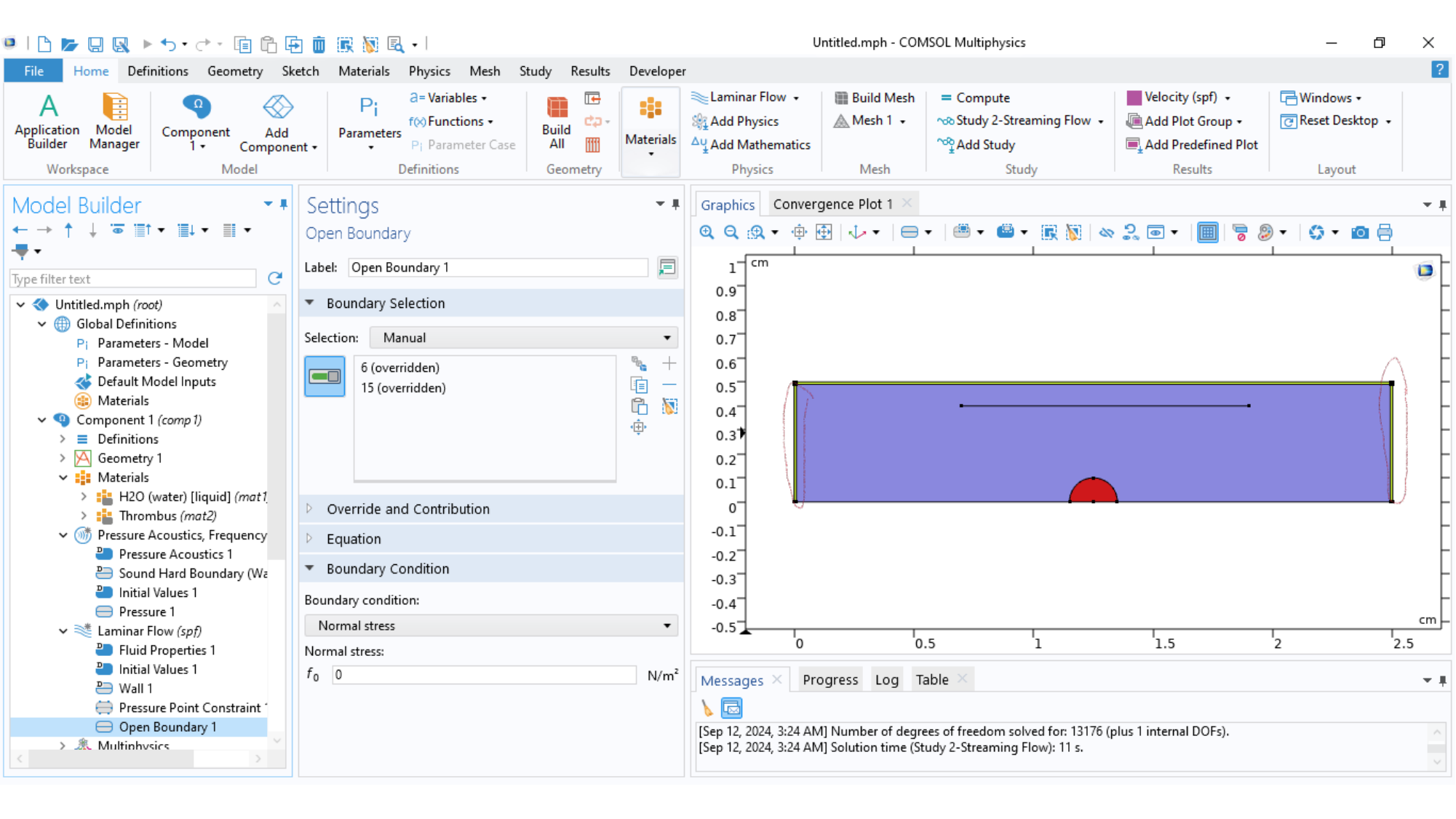

## Slide 63
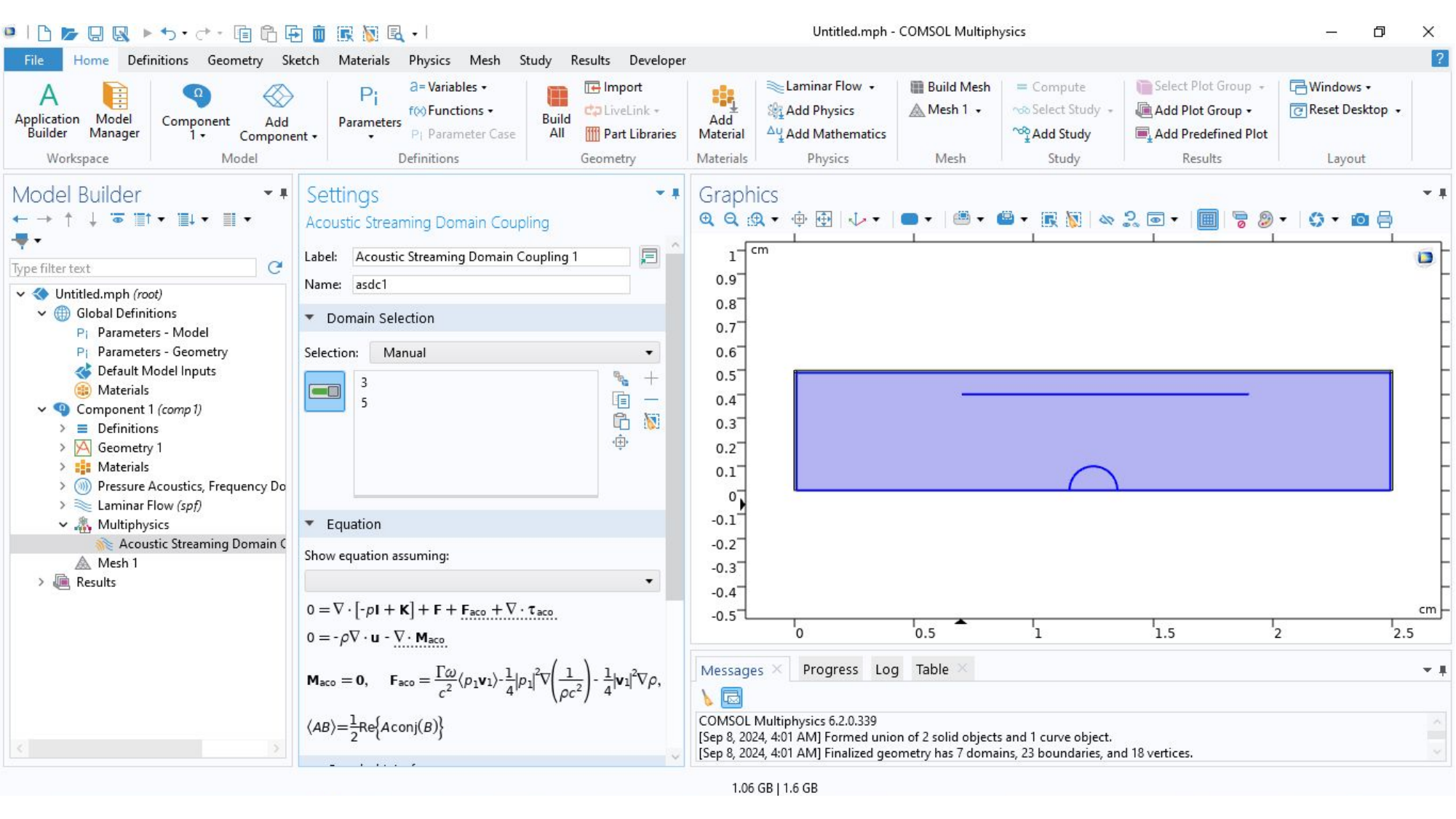

## Slide 64
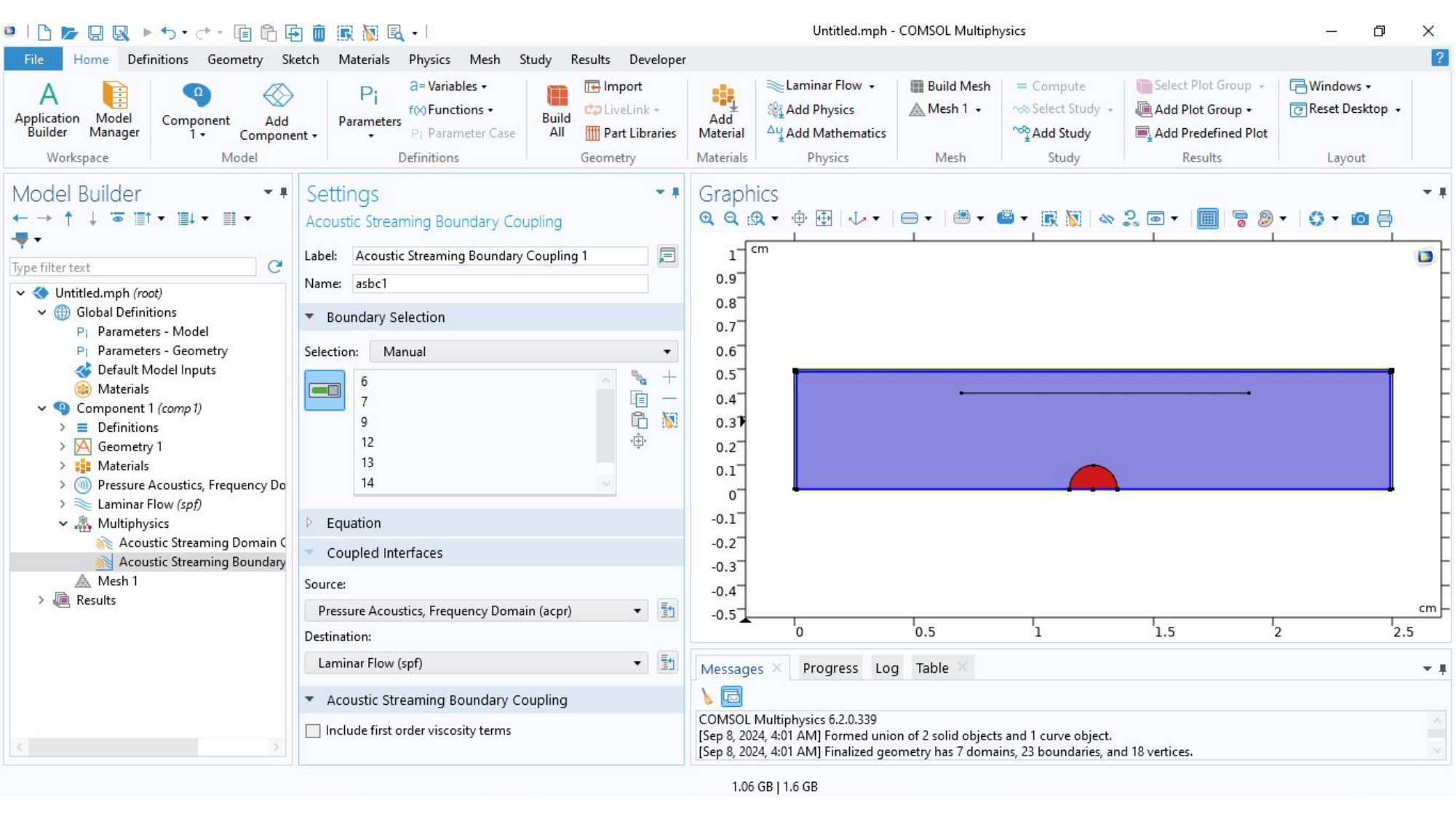

## Slide 65
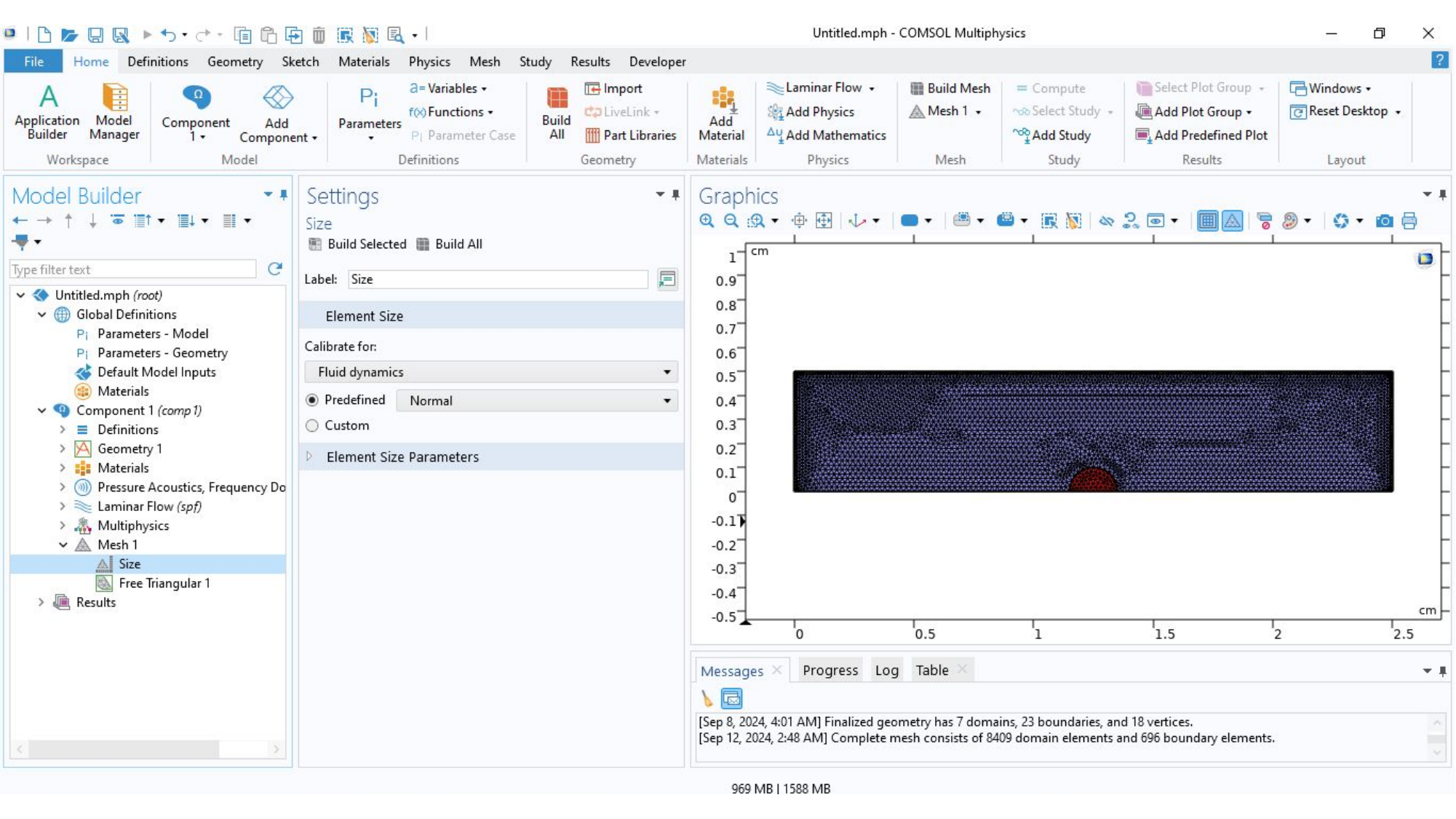

## Slide 66
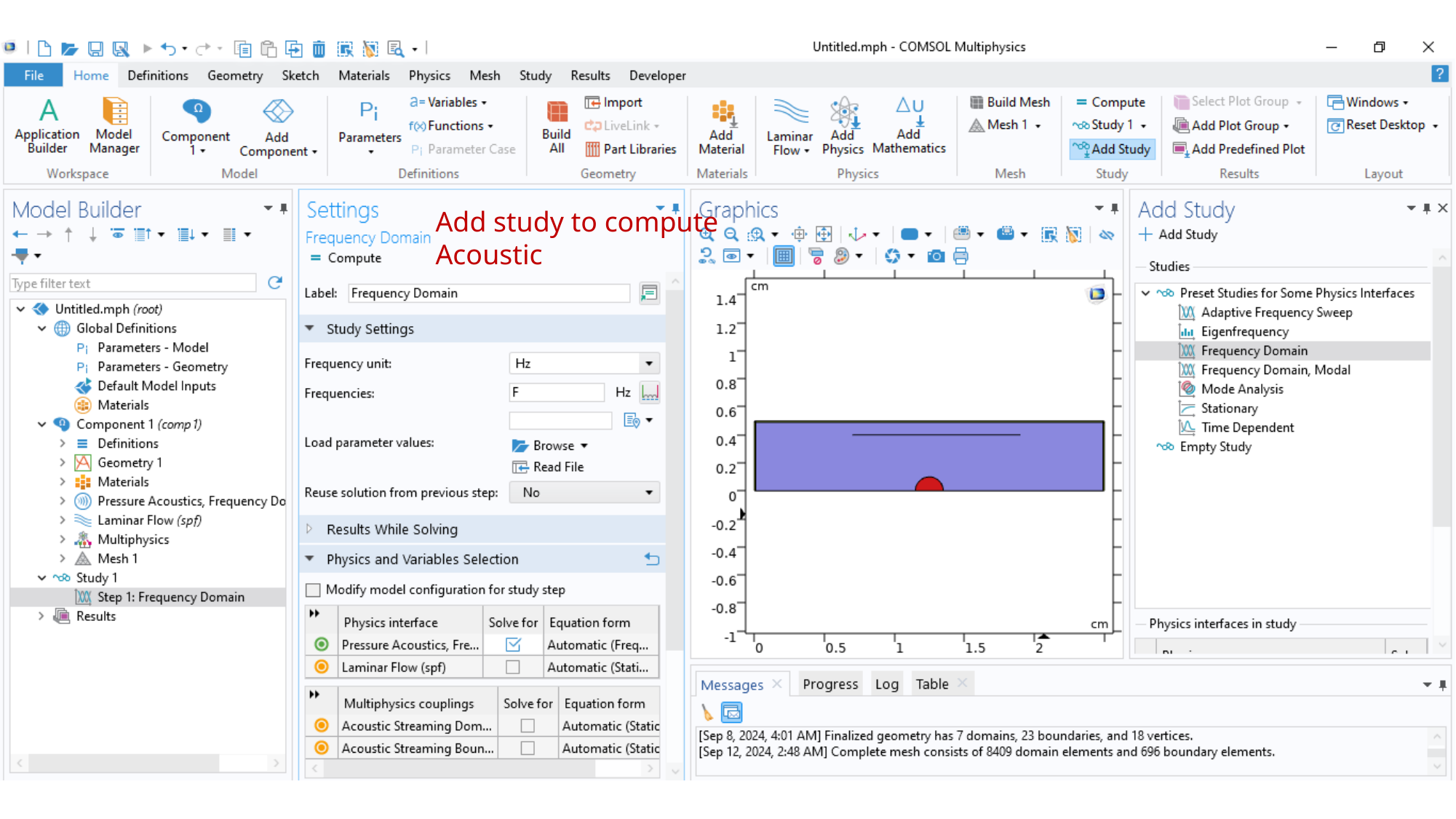

Add study to compute Acoustic

## Slide 67
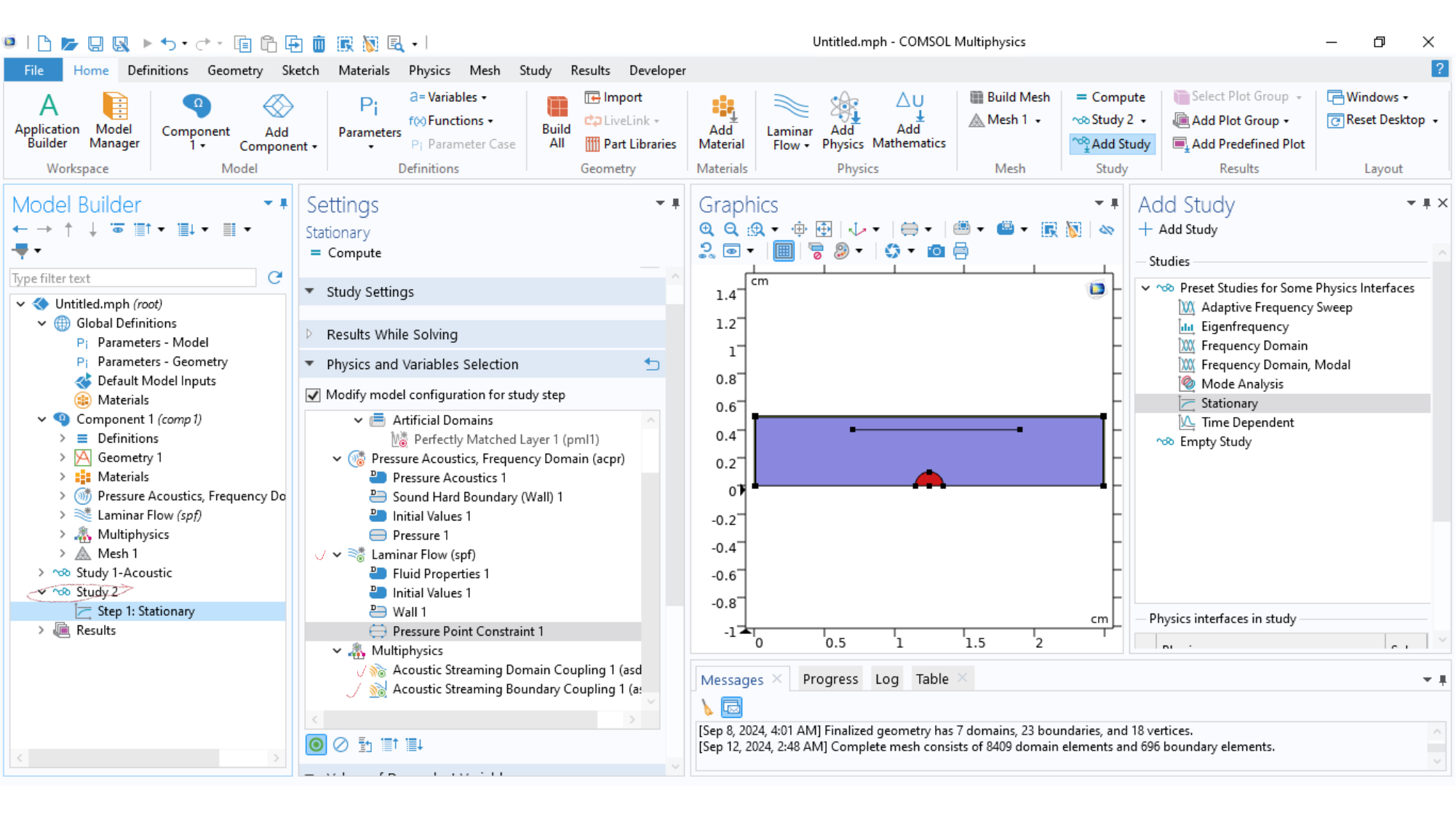

## Slide 68
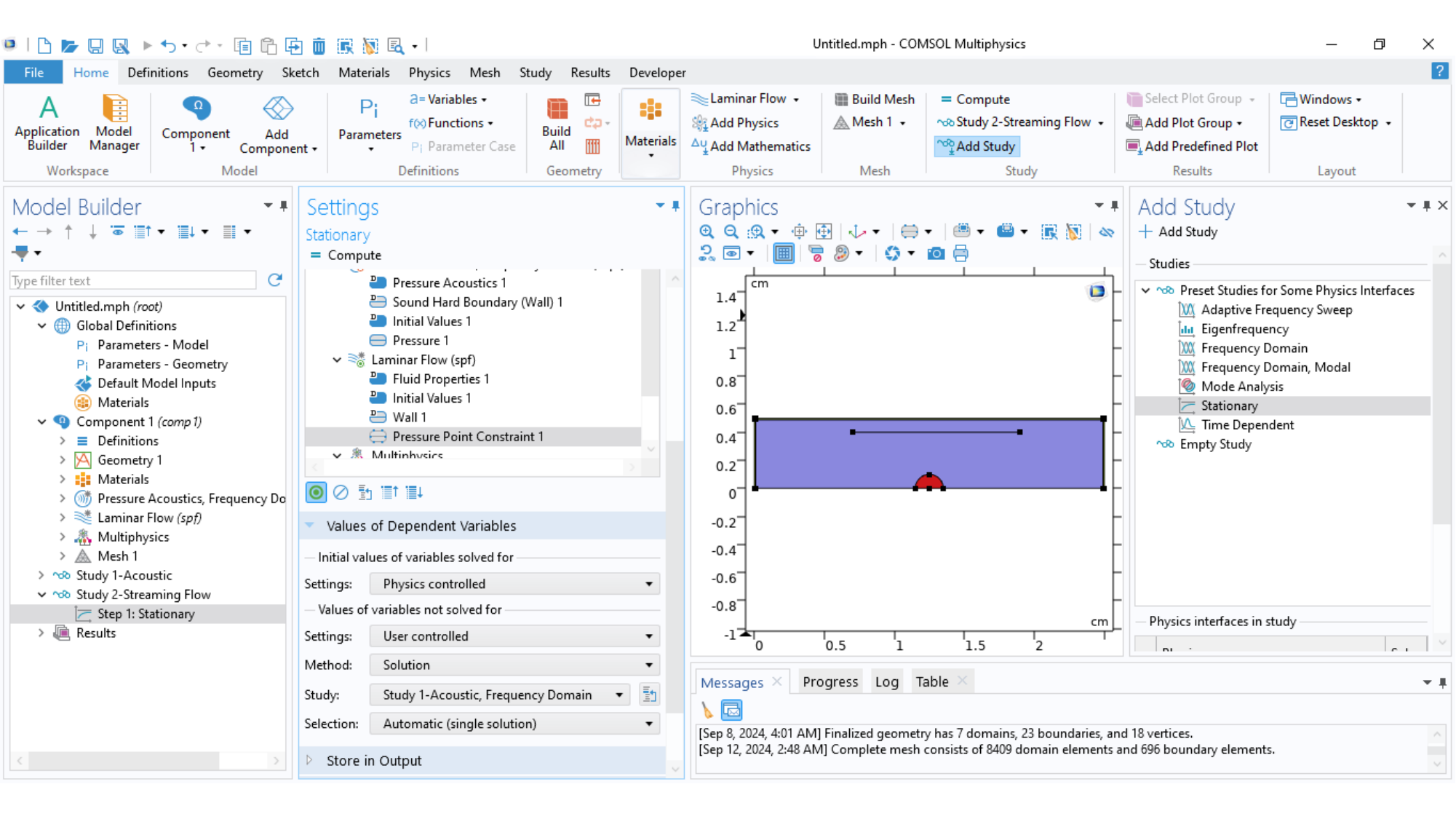

## Slide 69
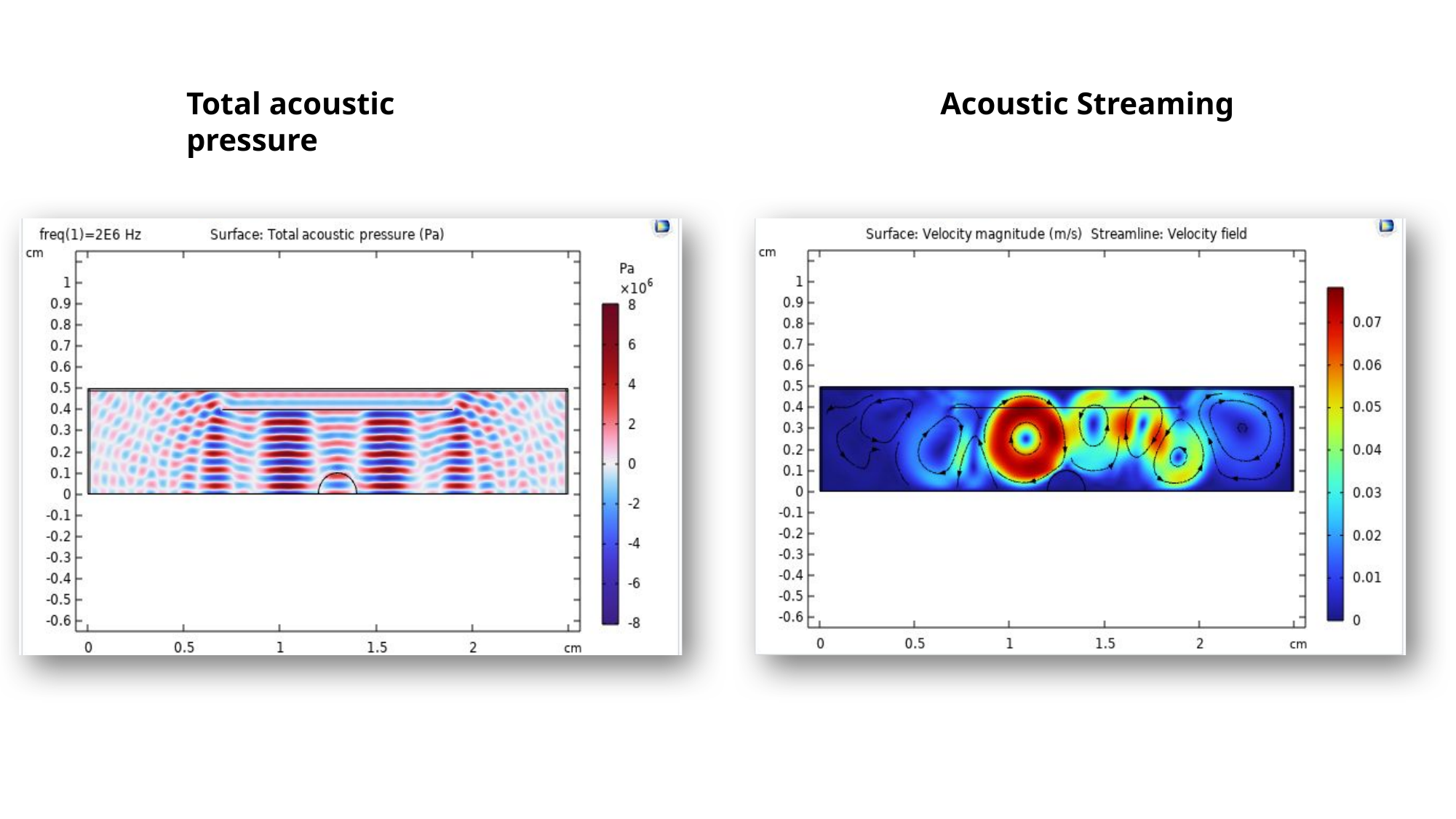

Total acoustic pressure
Acoustic Streaming

## Slide 70
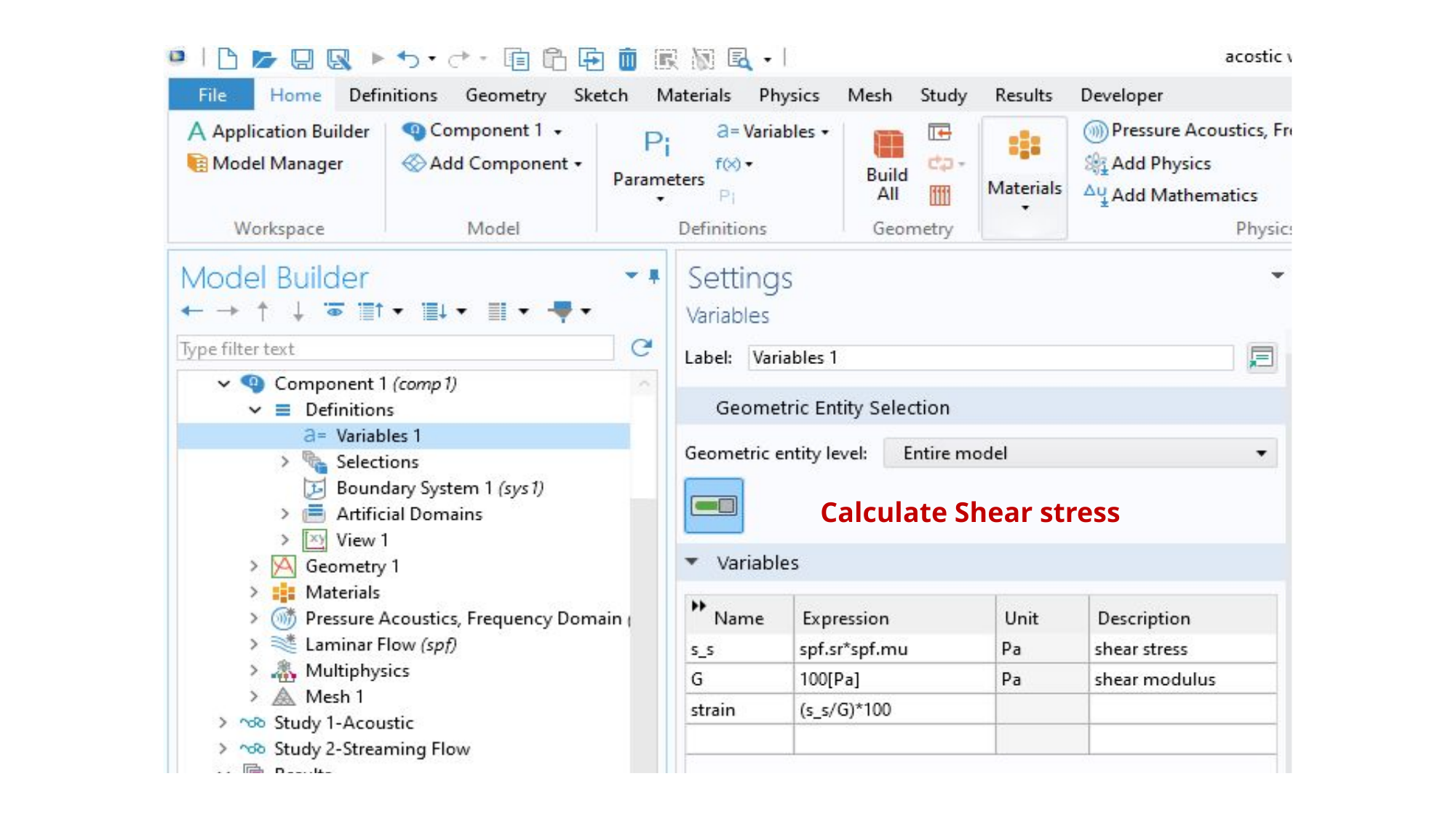

Calculate Shear stress

## Slide 71
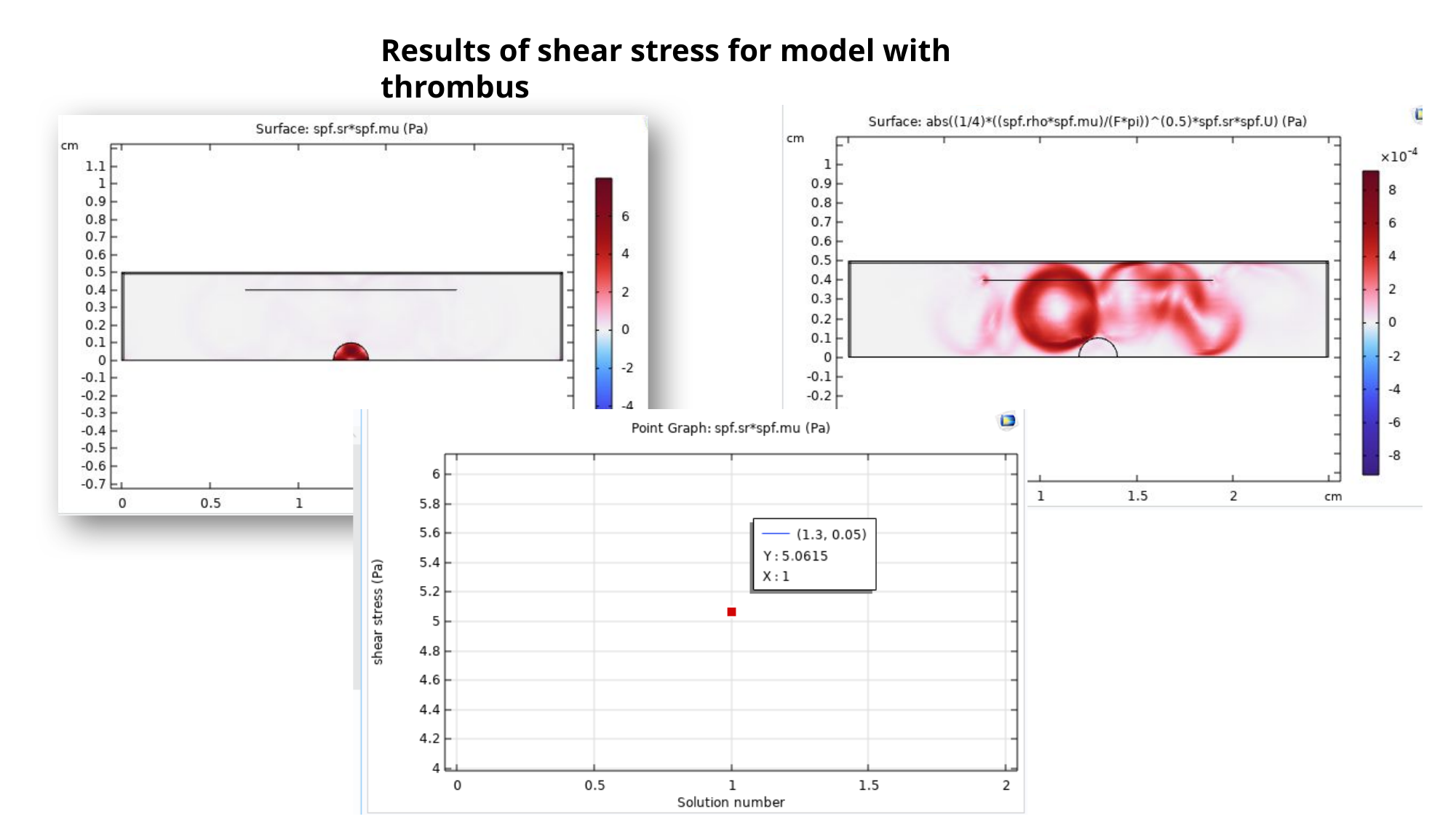

Results of shear stress for model with thrombus

## Slide 72
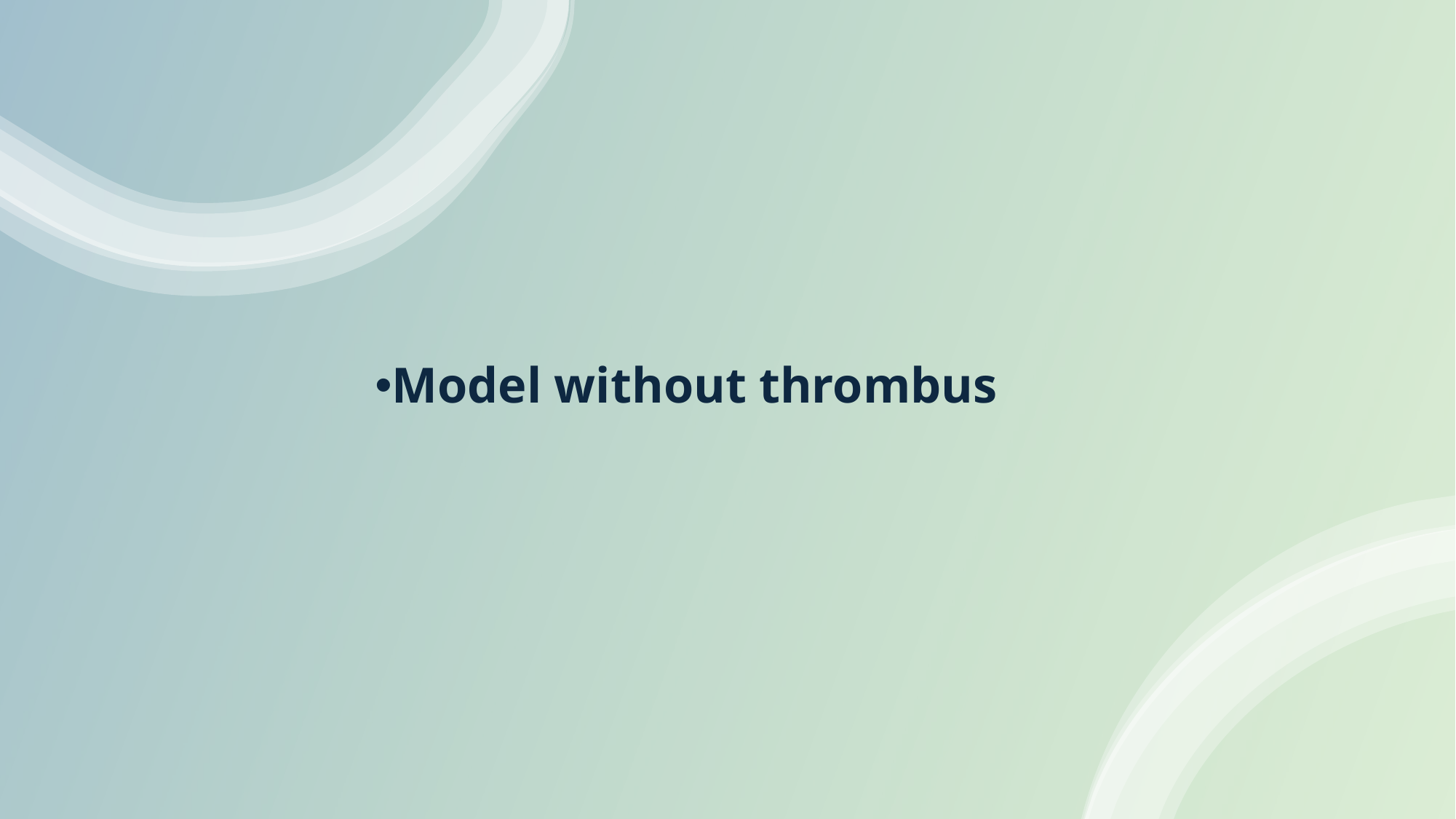

Model without thrombus

## Slide 73
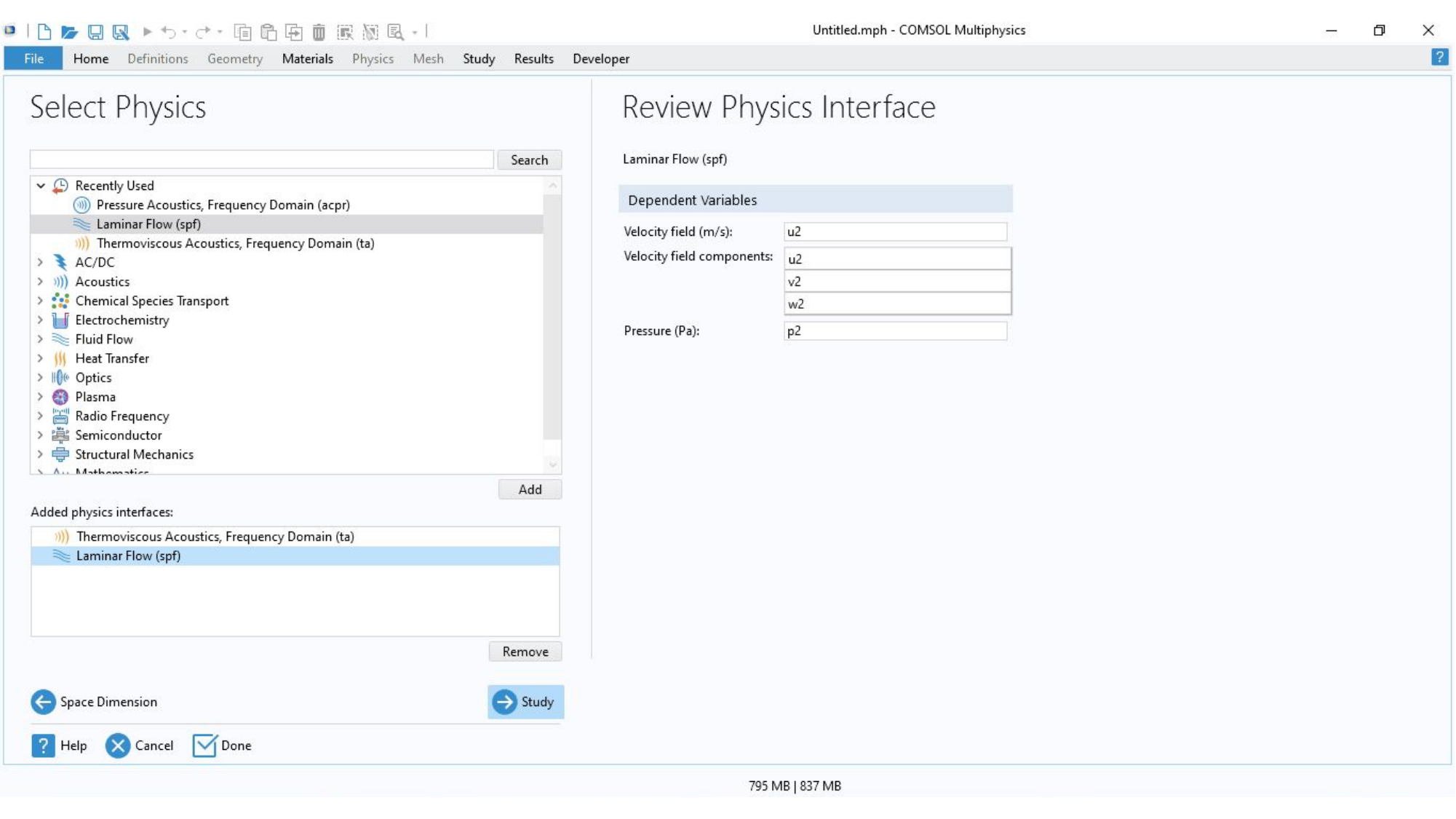

## Slide 74
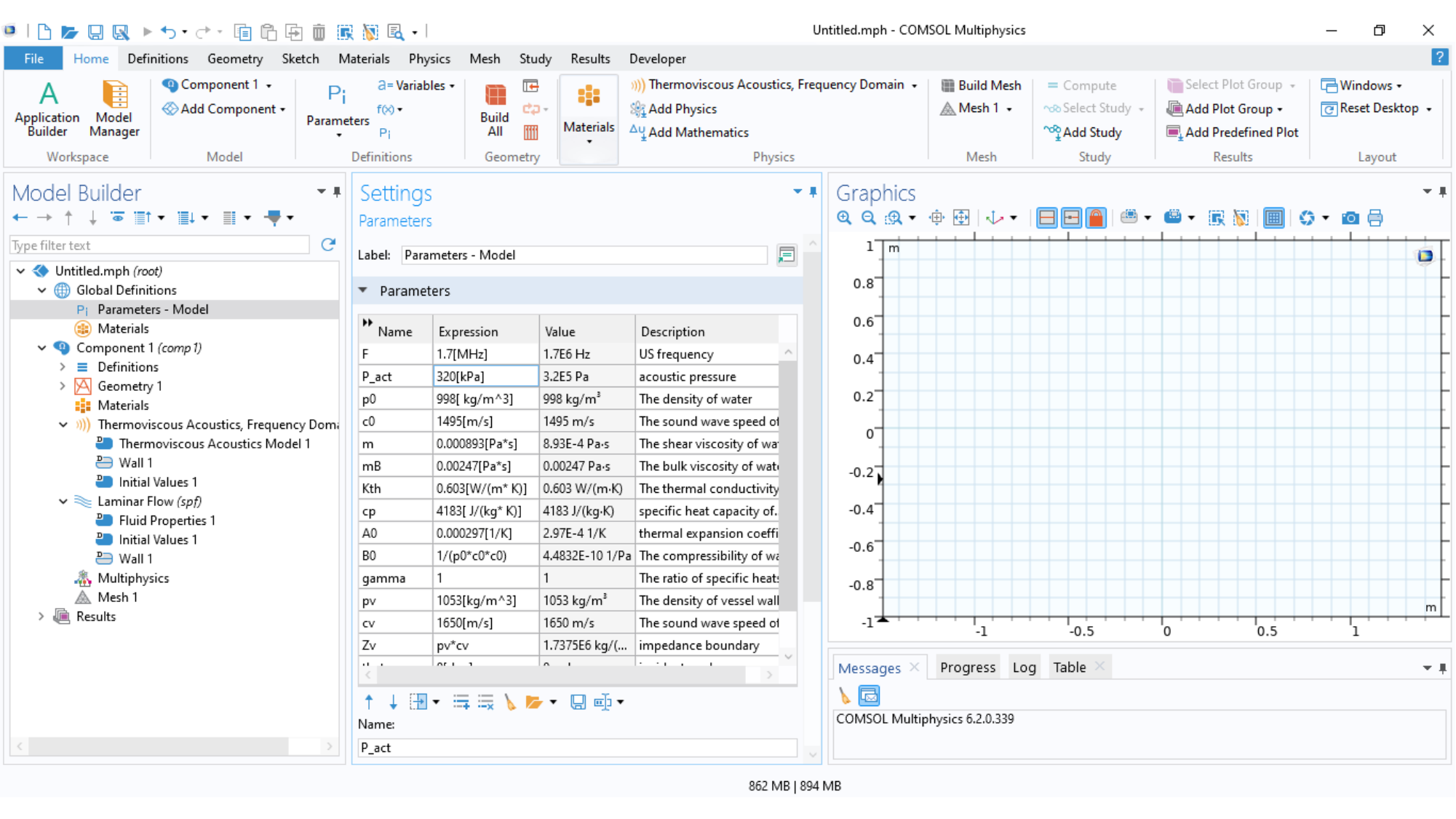

## Slide 75
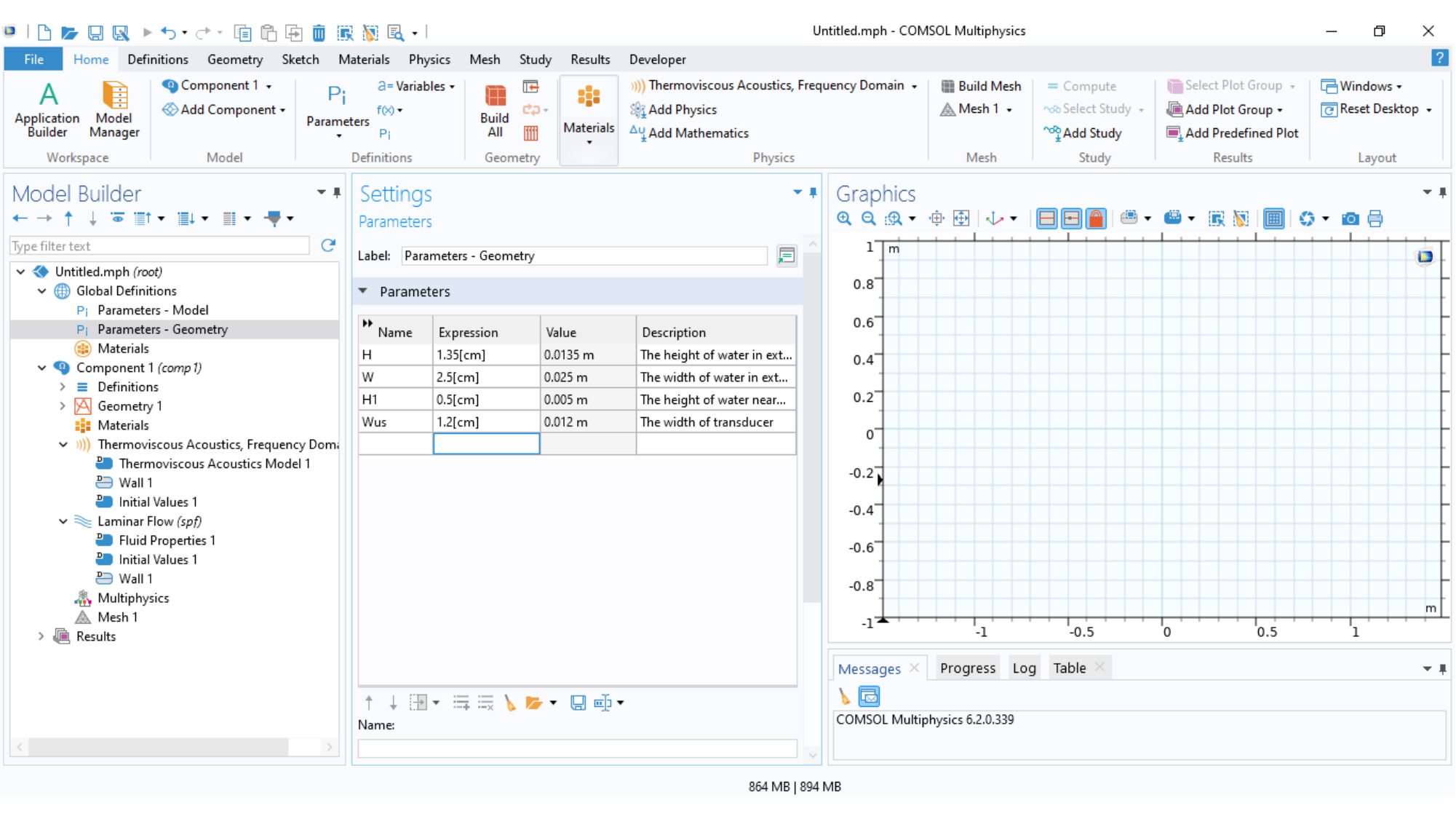

## Slide 76
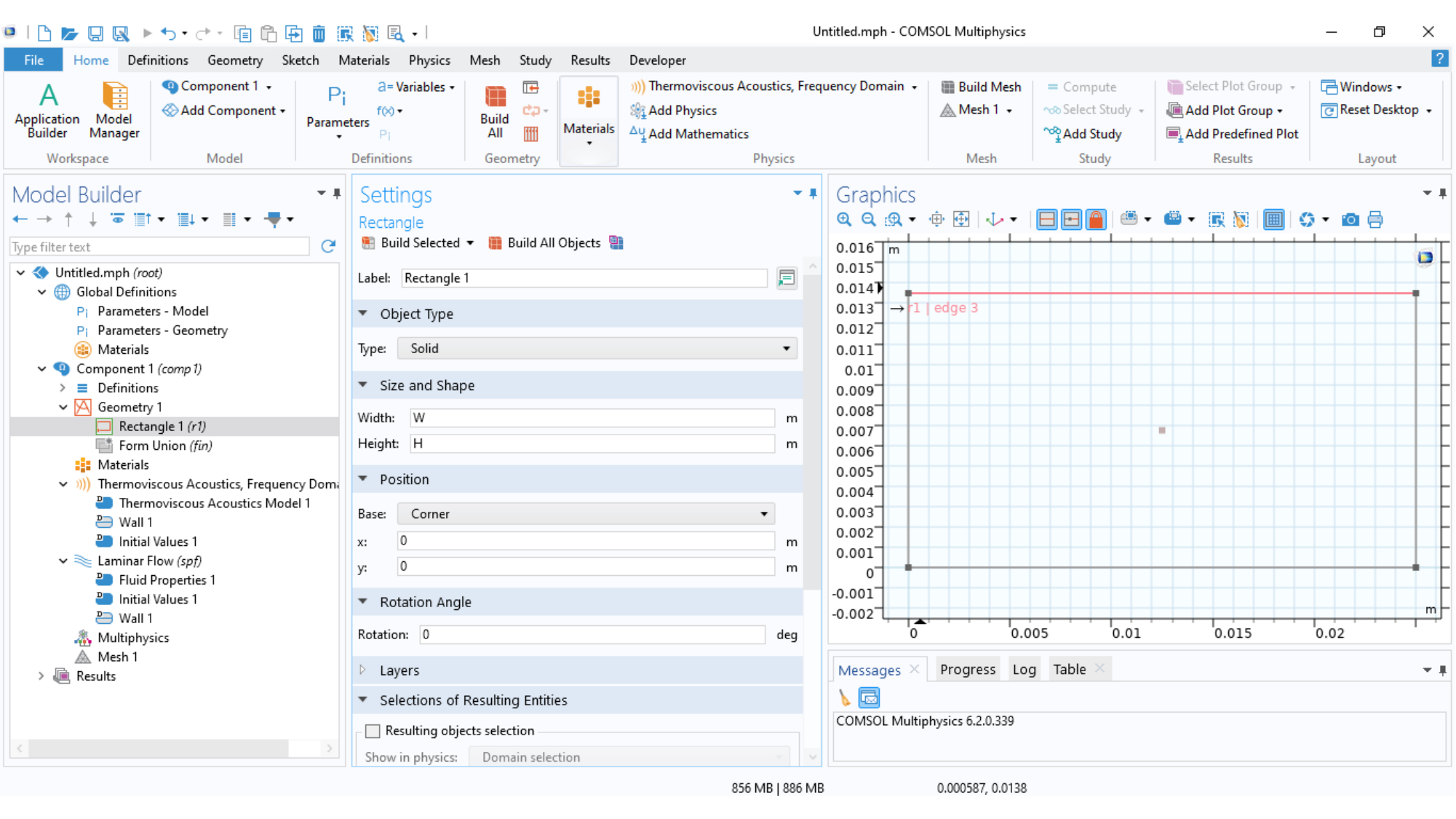

## Slide 77
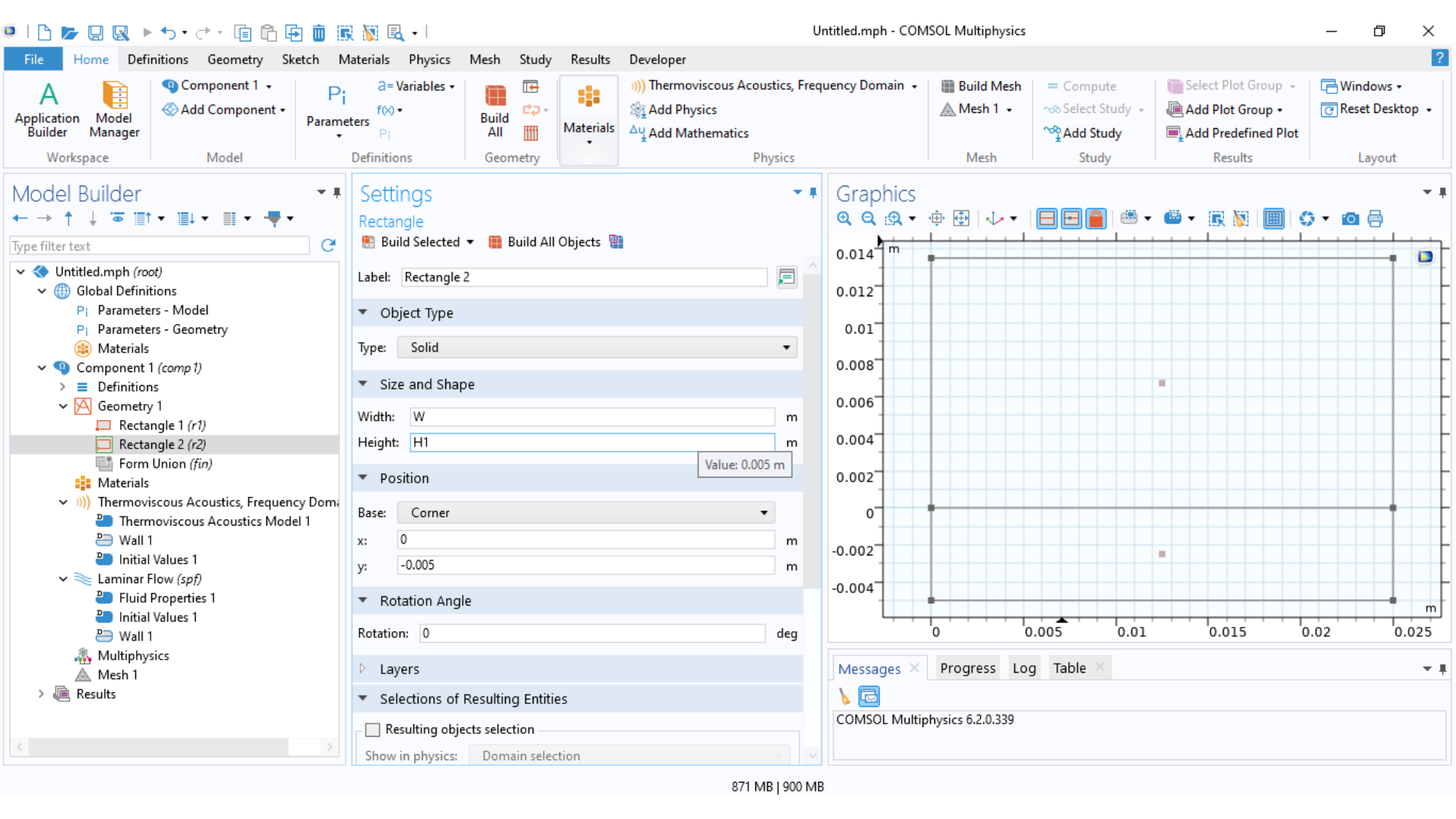

## Slide 78
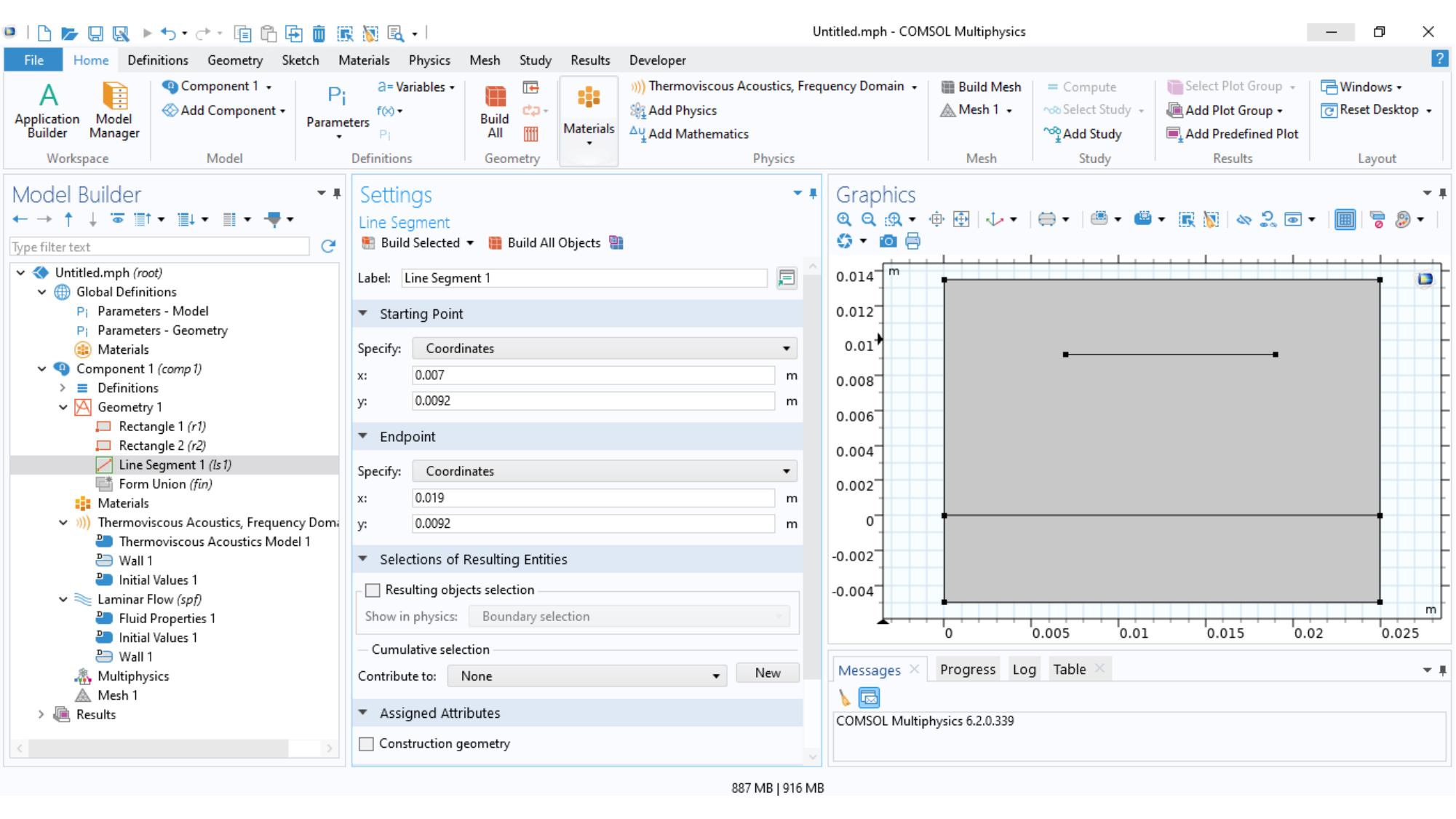

## Slide 79
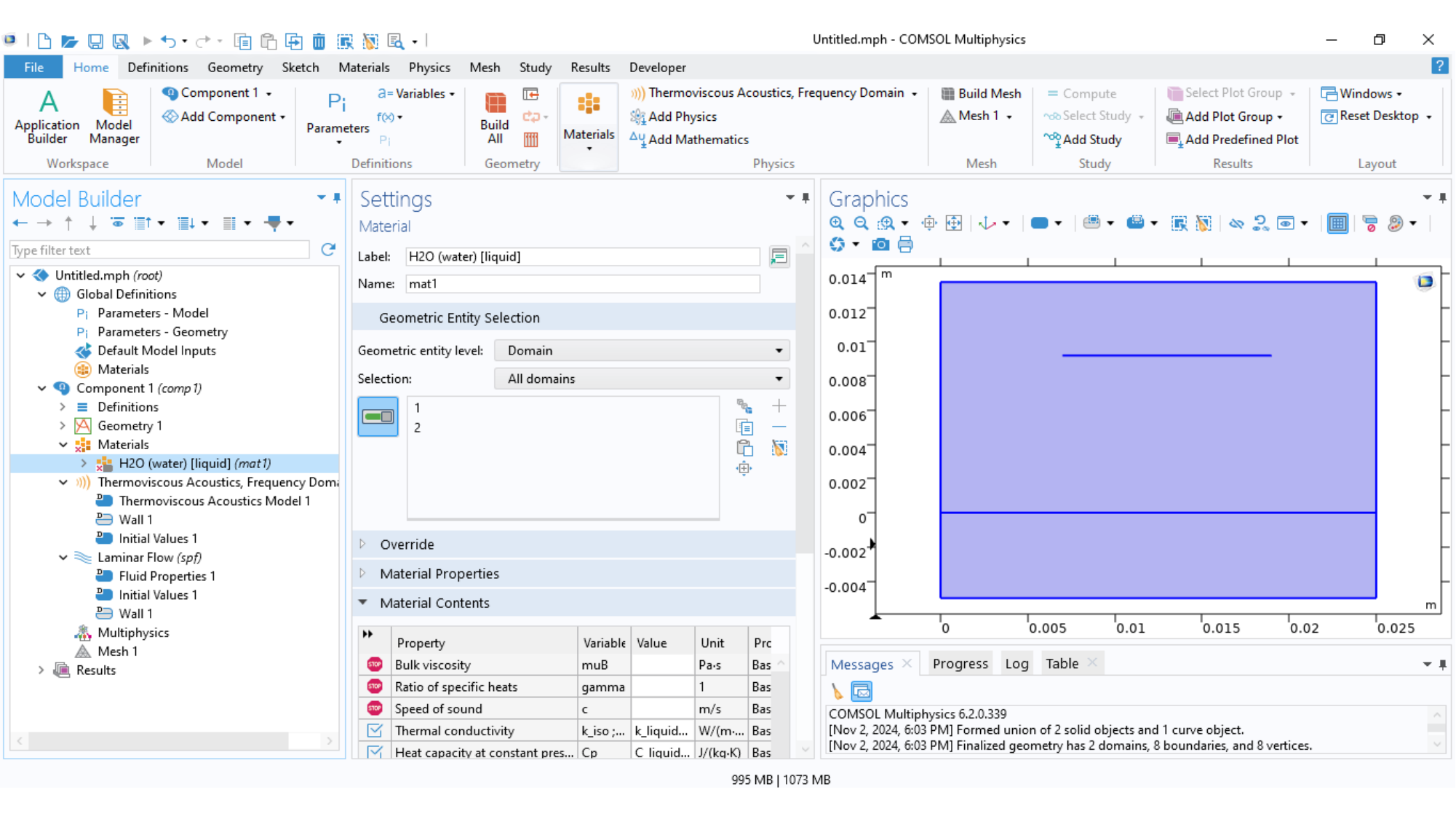

## Slide 80
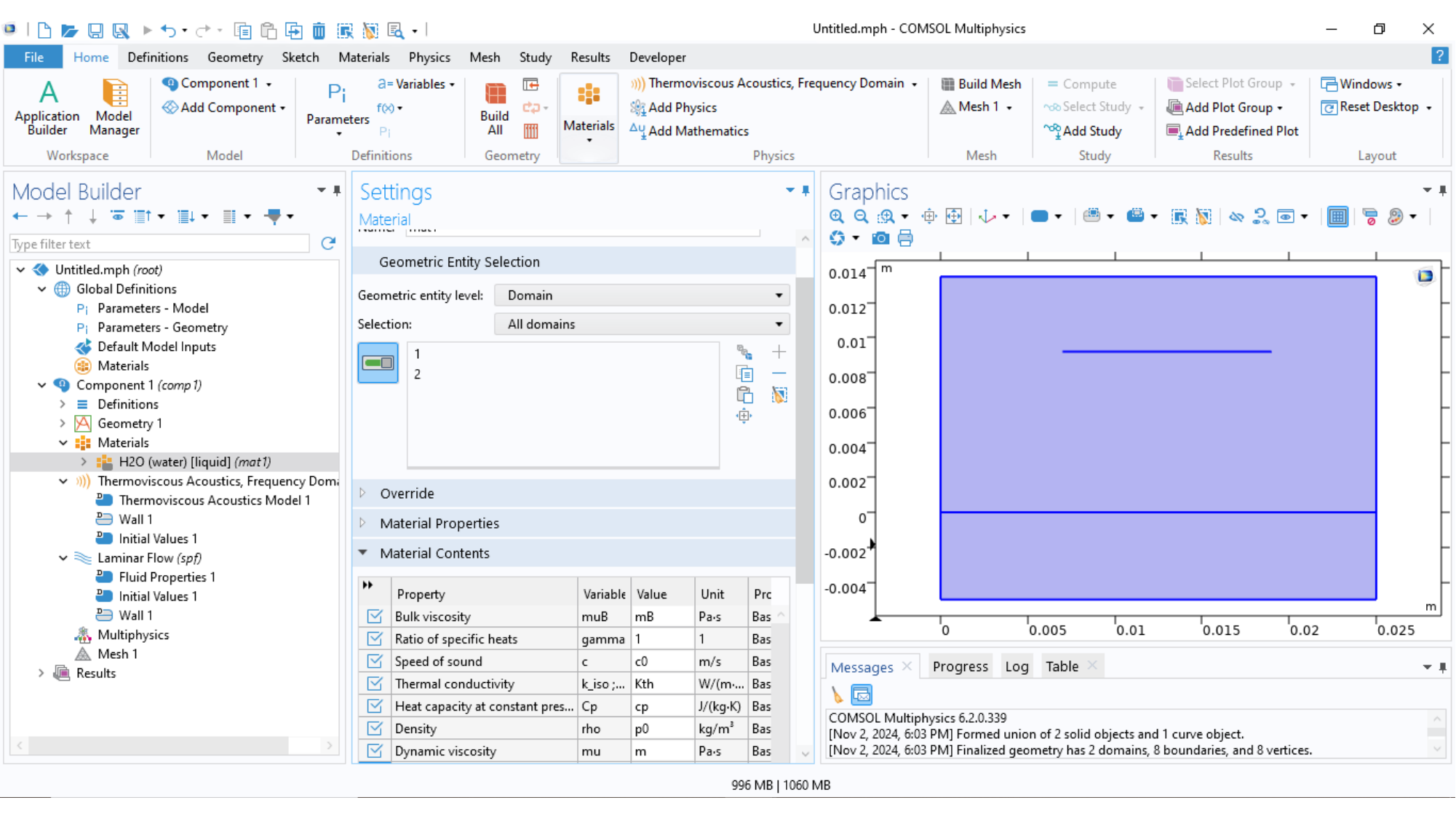

## Slide 81
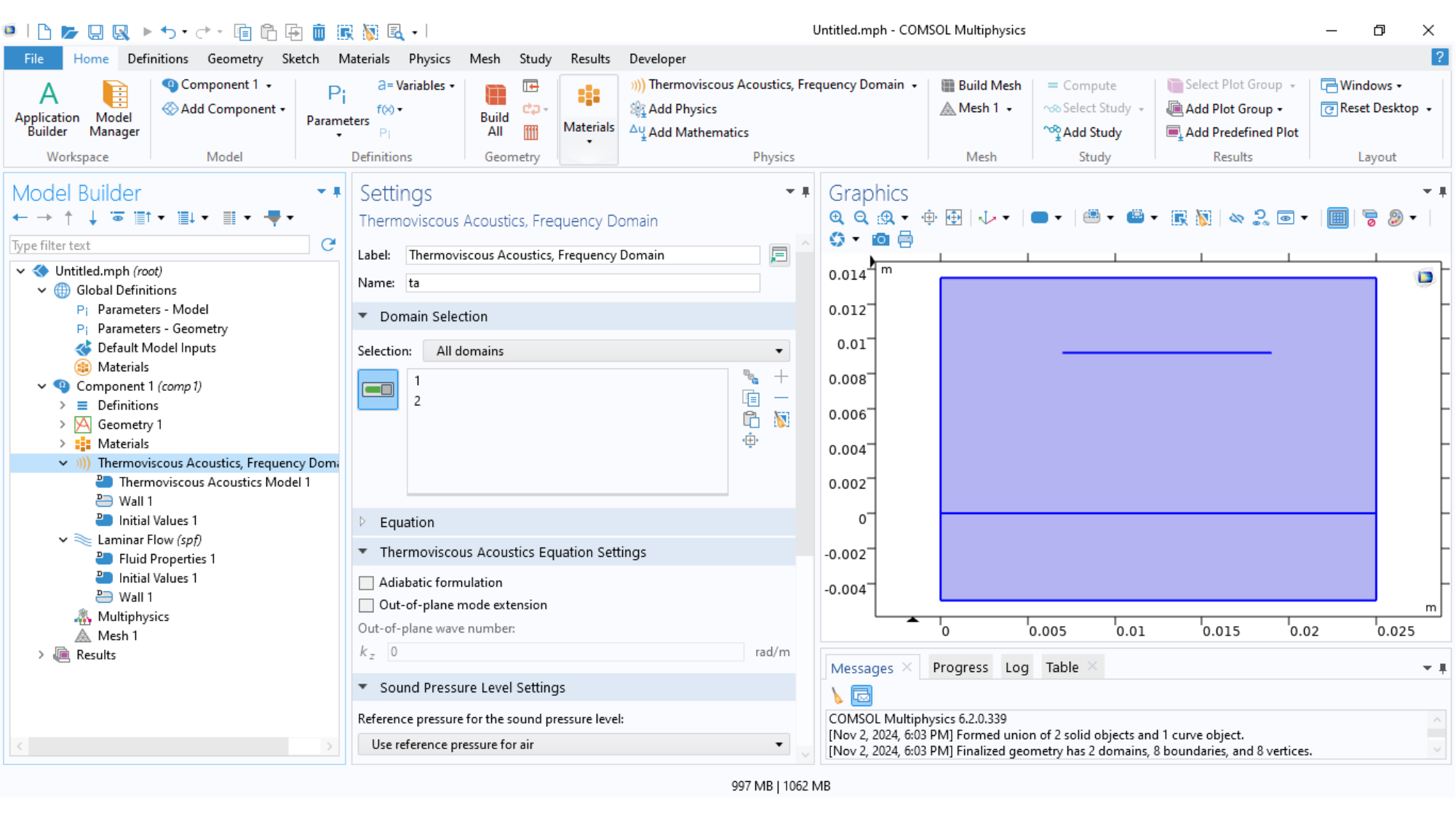

## Slide 82
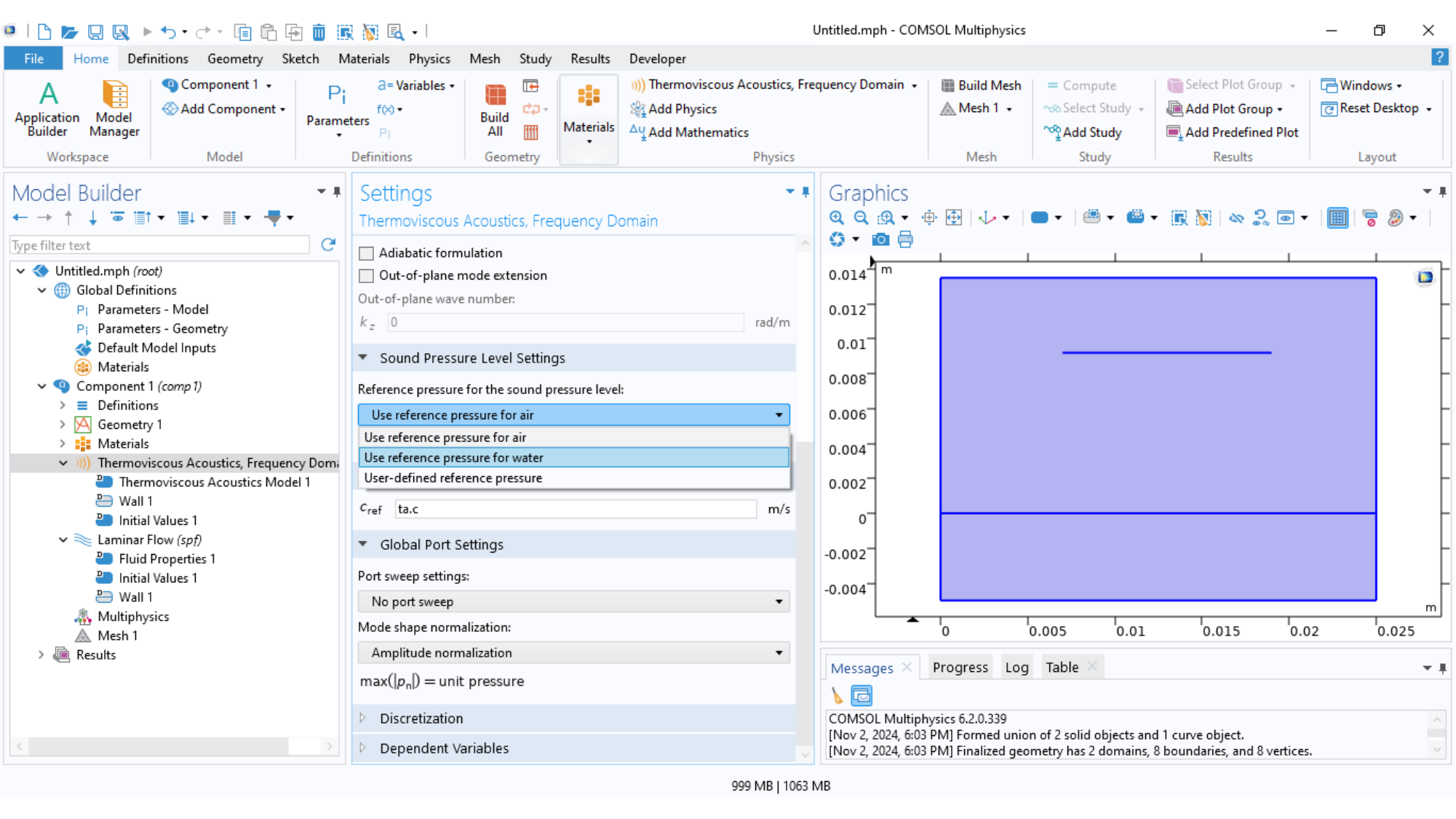

## Slide 83
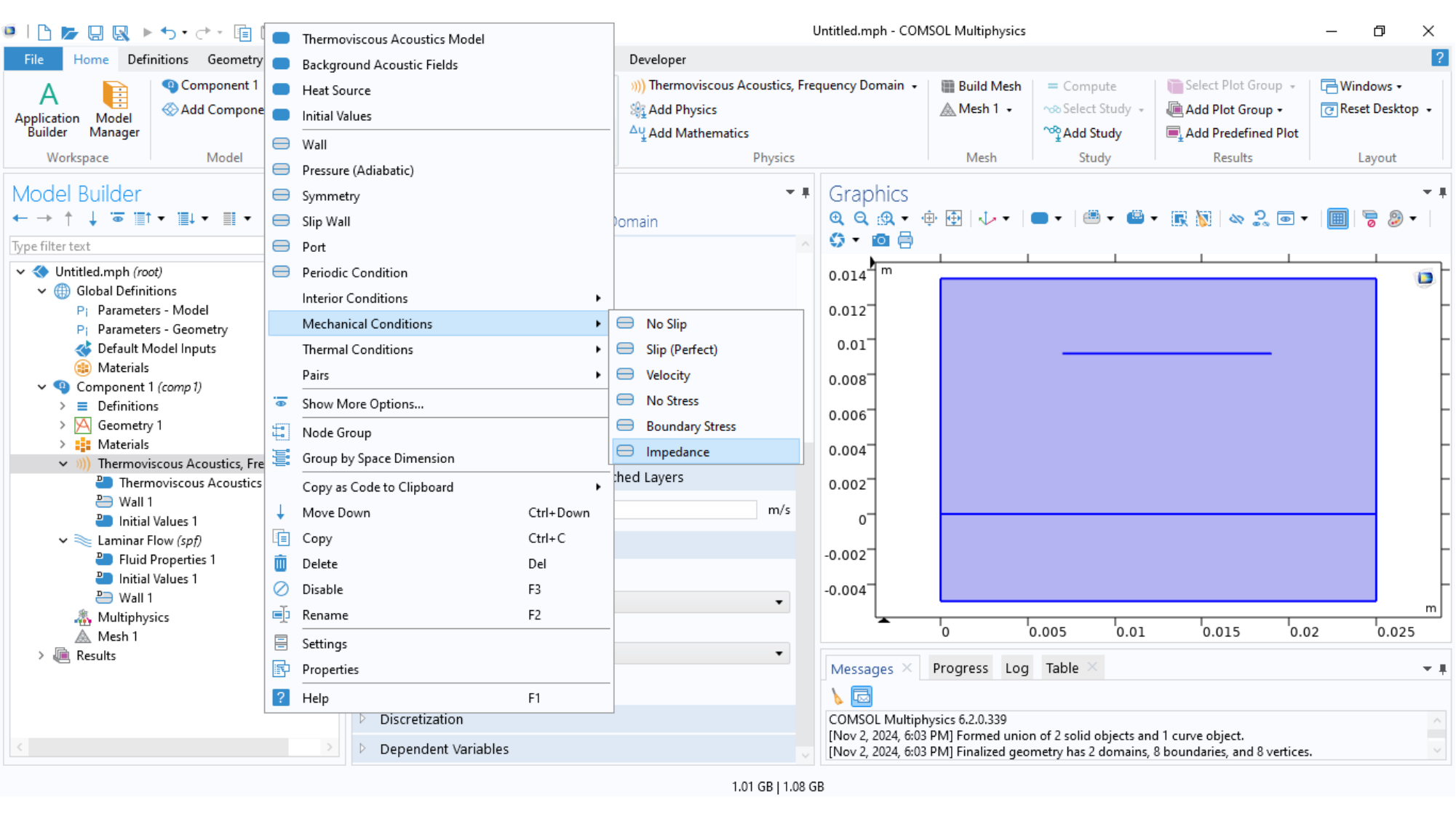

## Slide 84
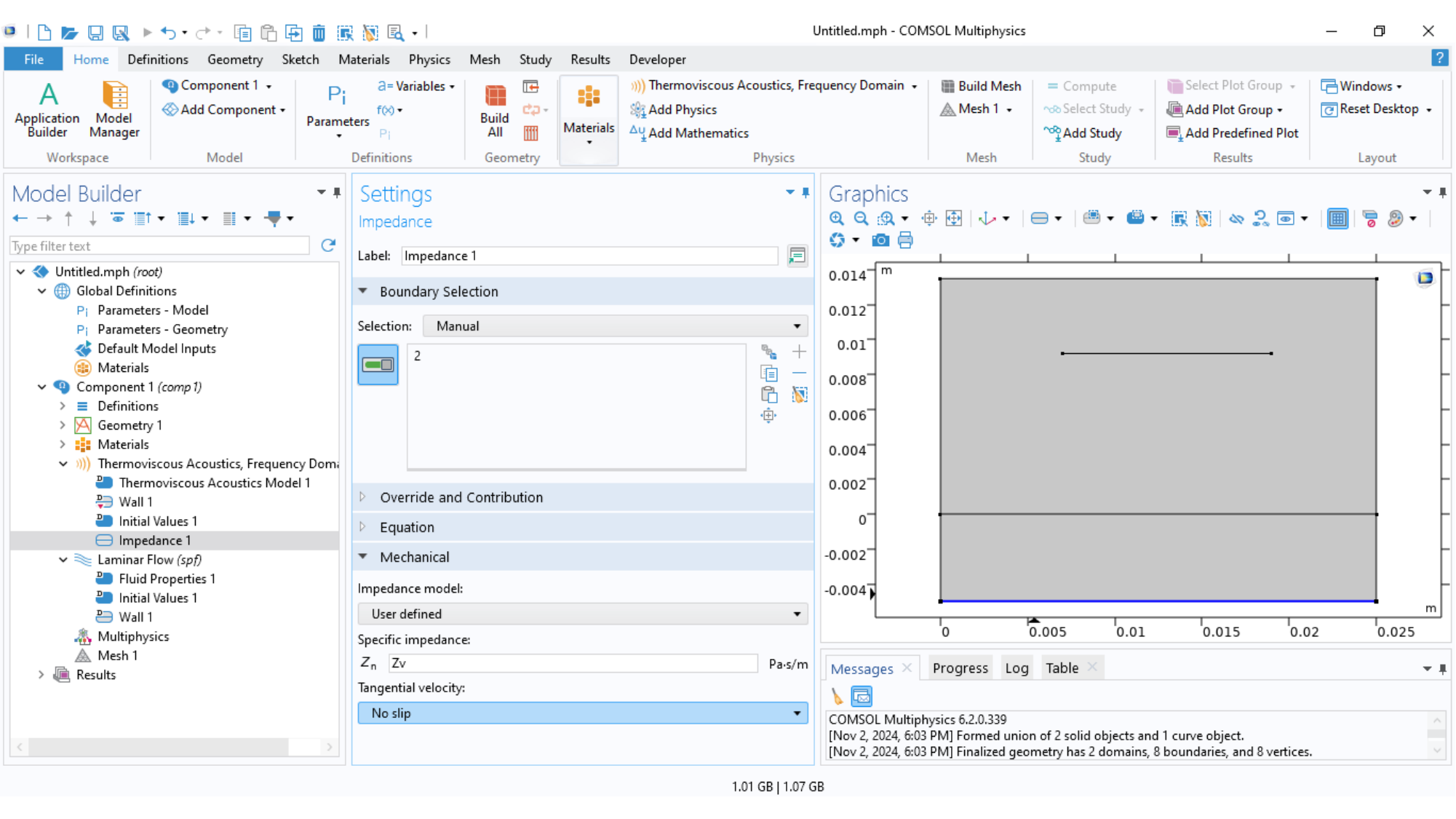

## Slide 85
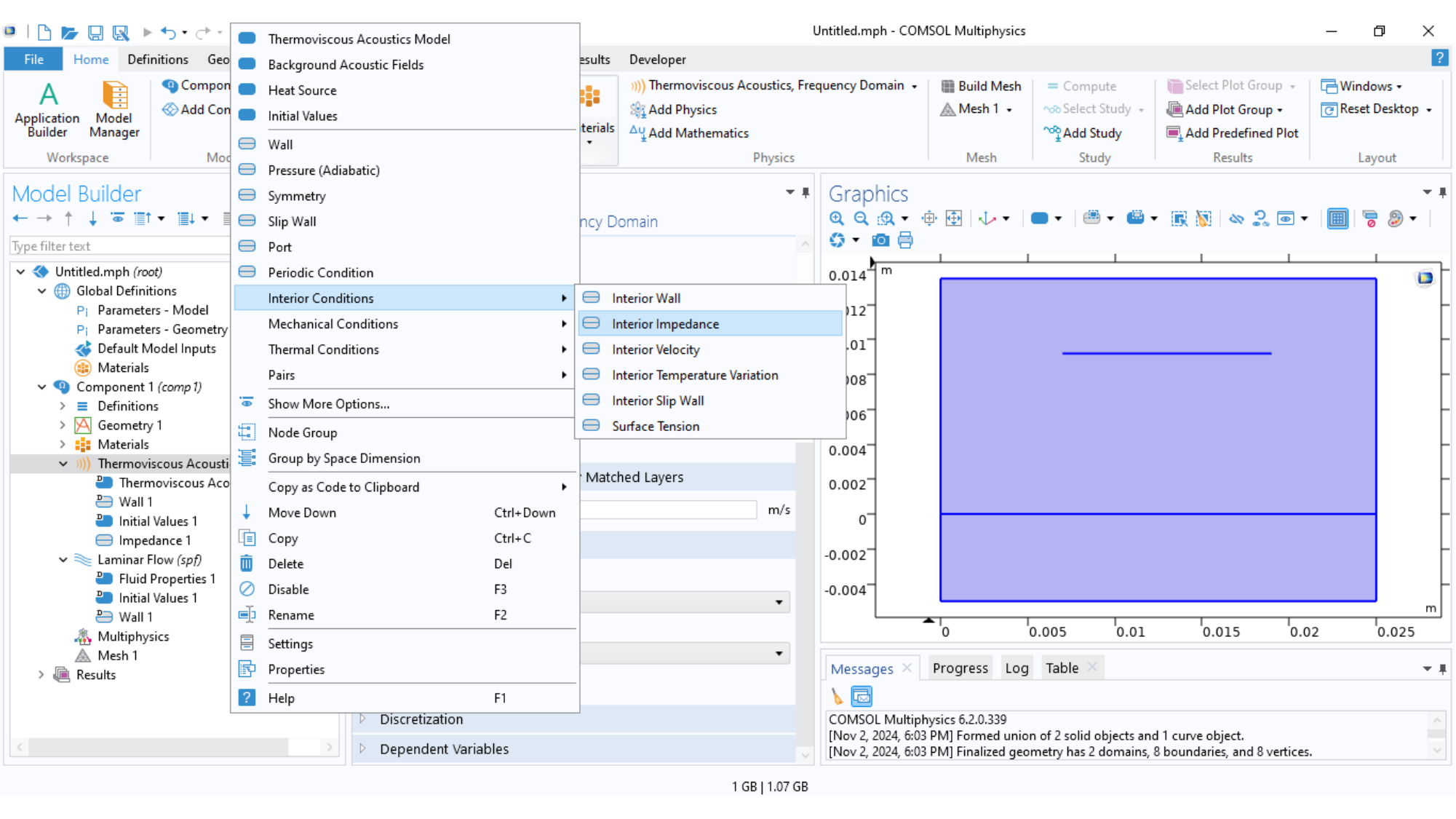

## Slide 86
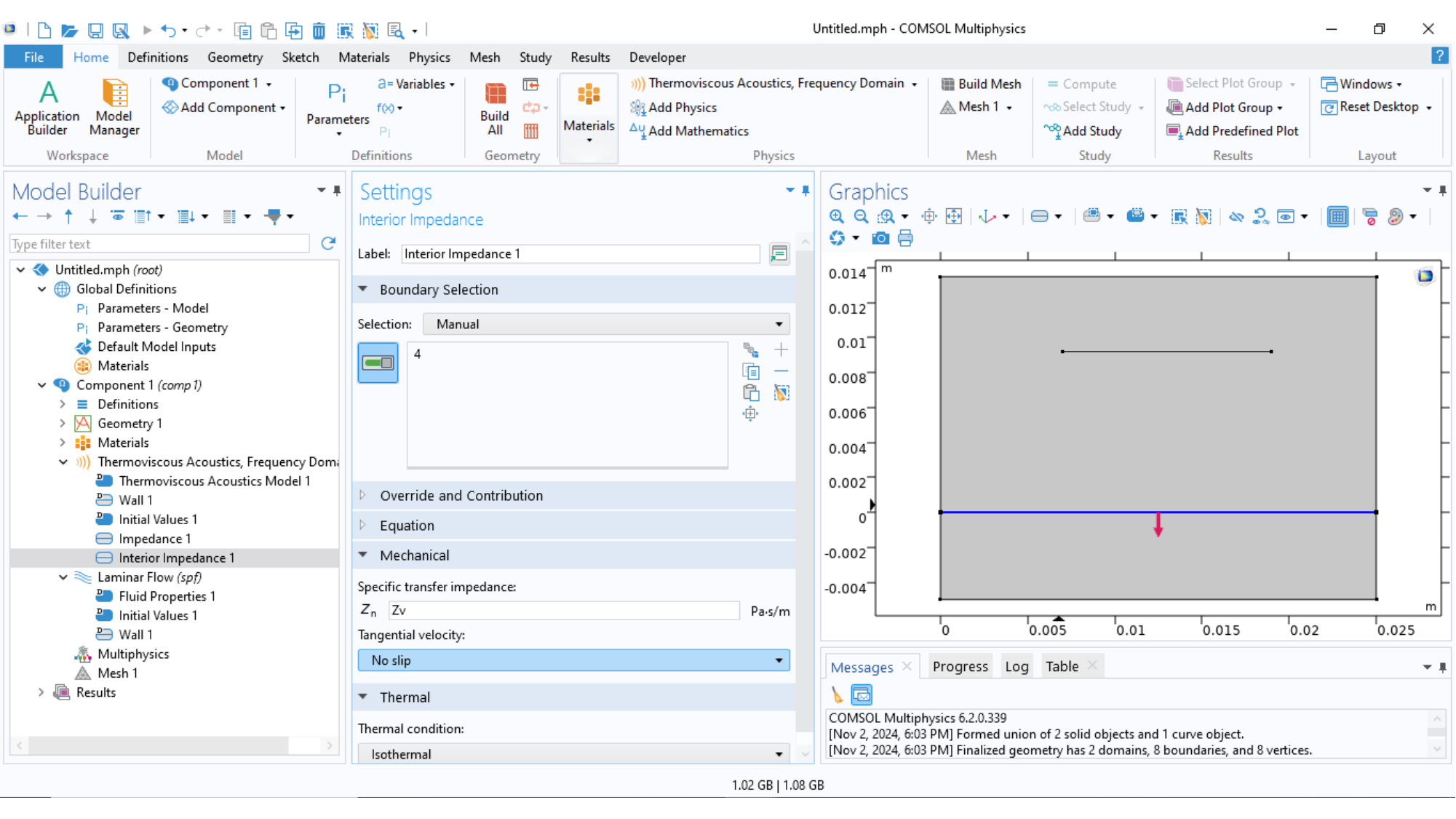

## Slide 87
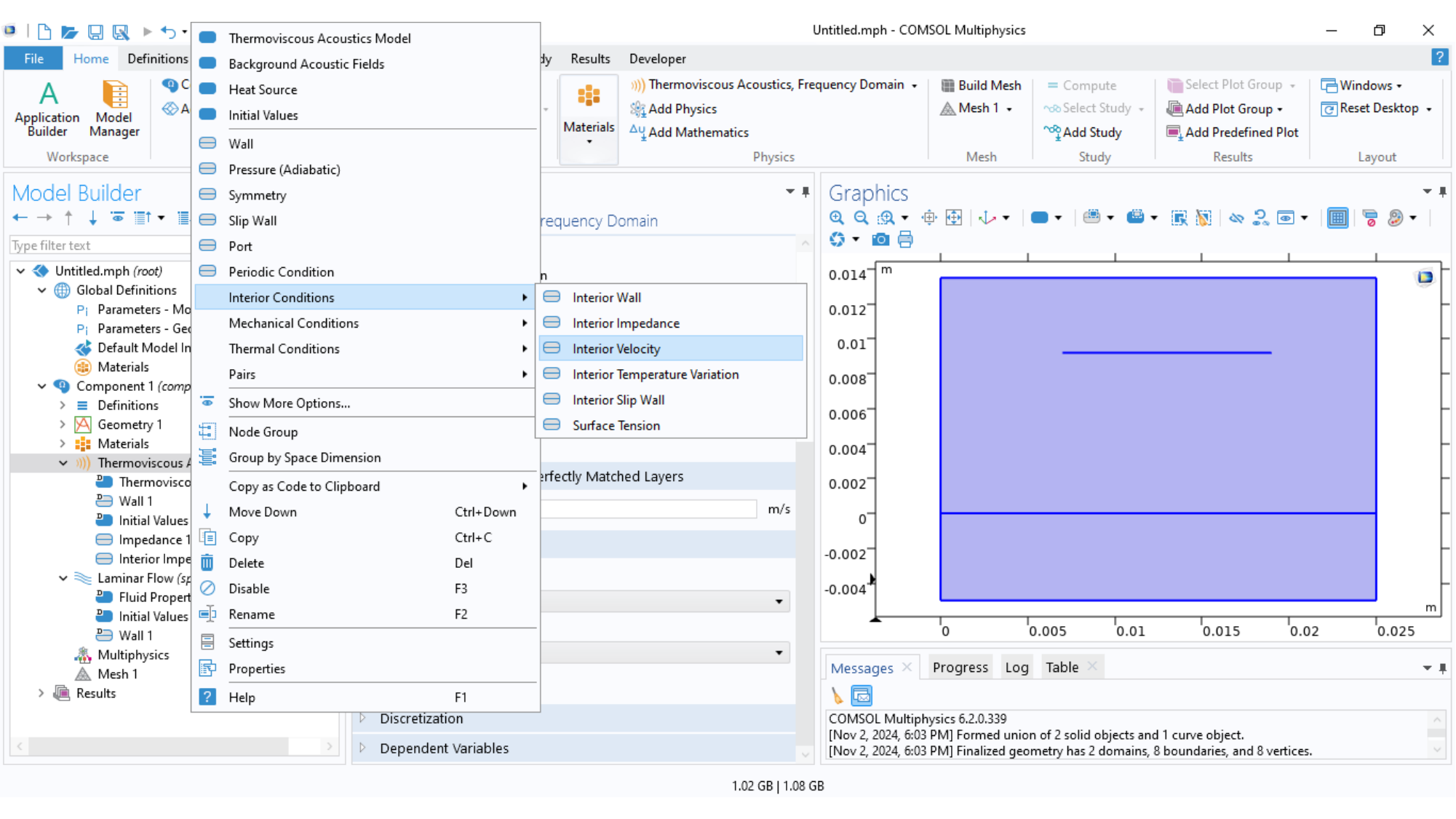

## Slide 88
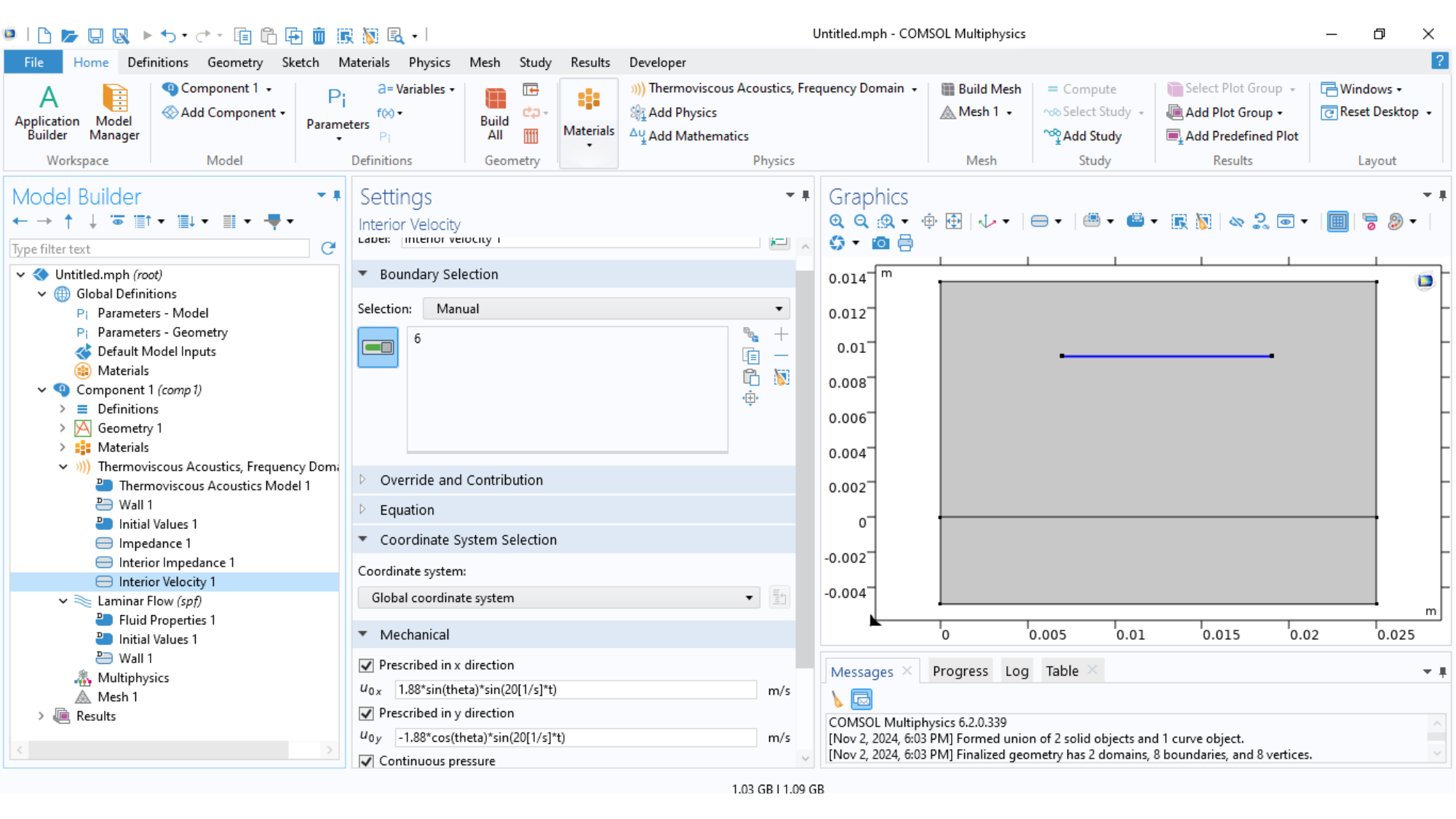

## Slide 89
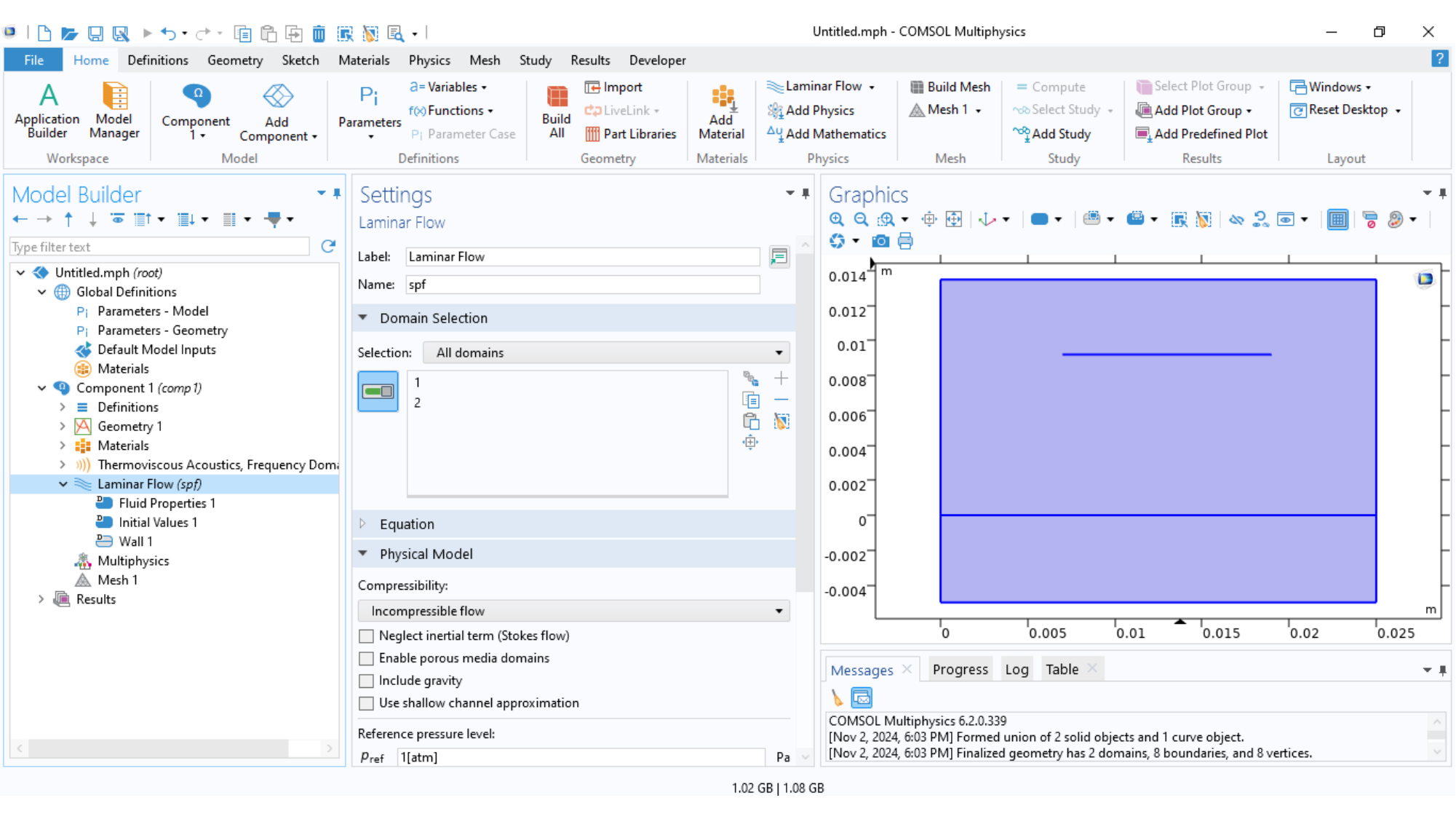

## Slide 90
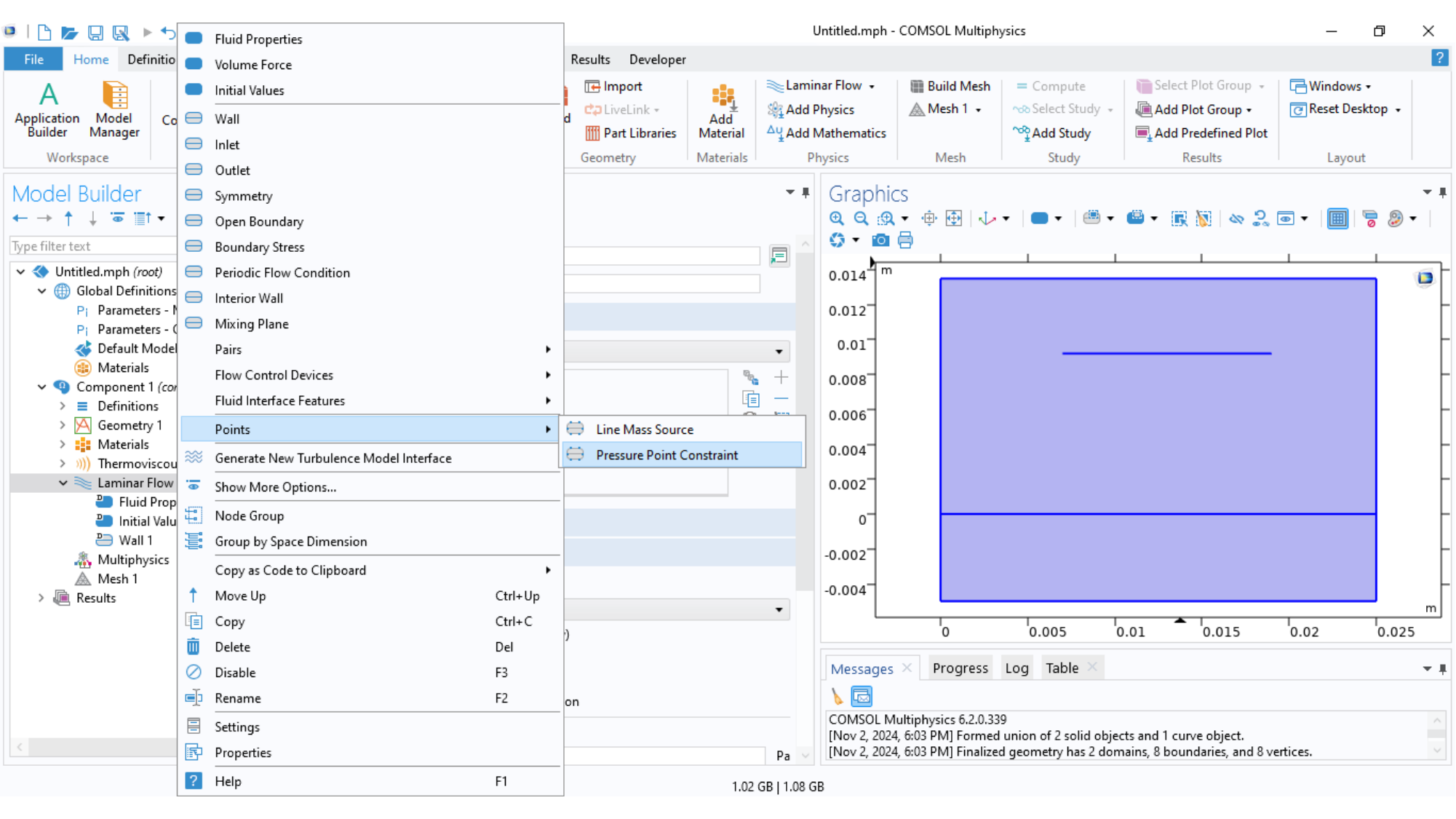

## Slide 91
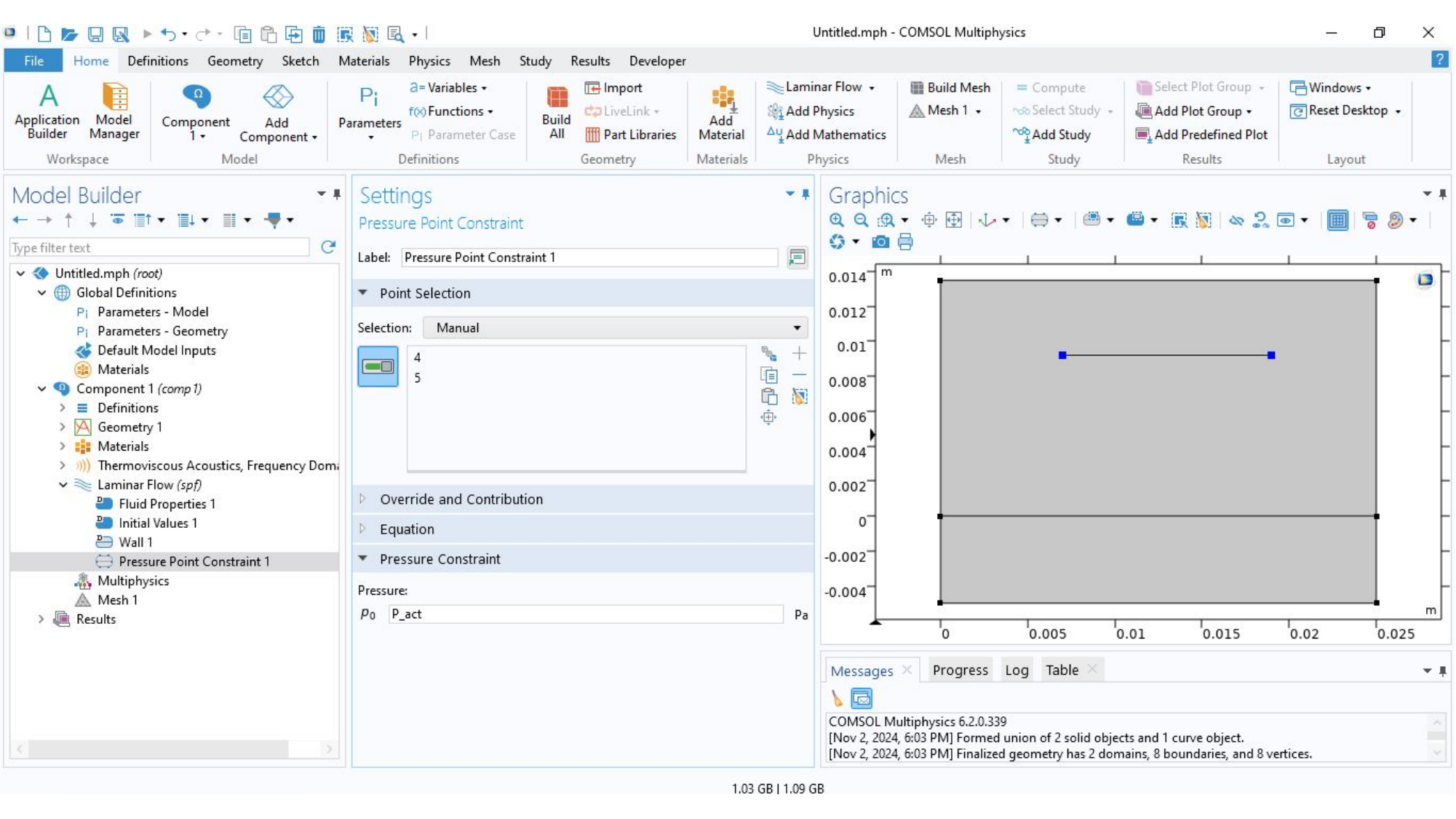

## Slide 92
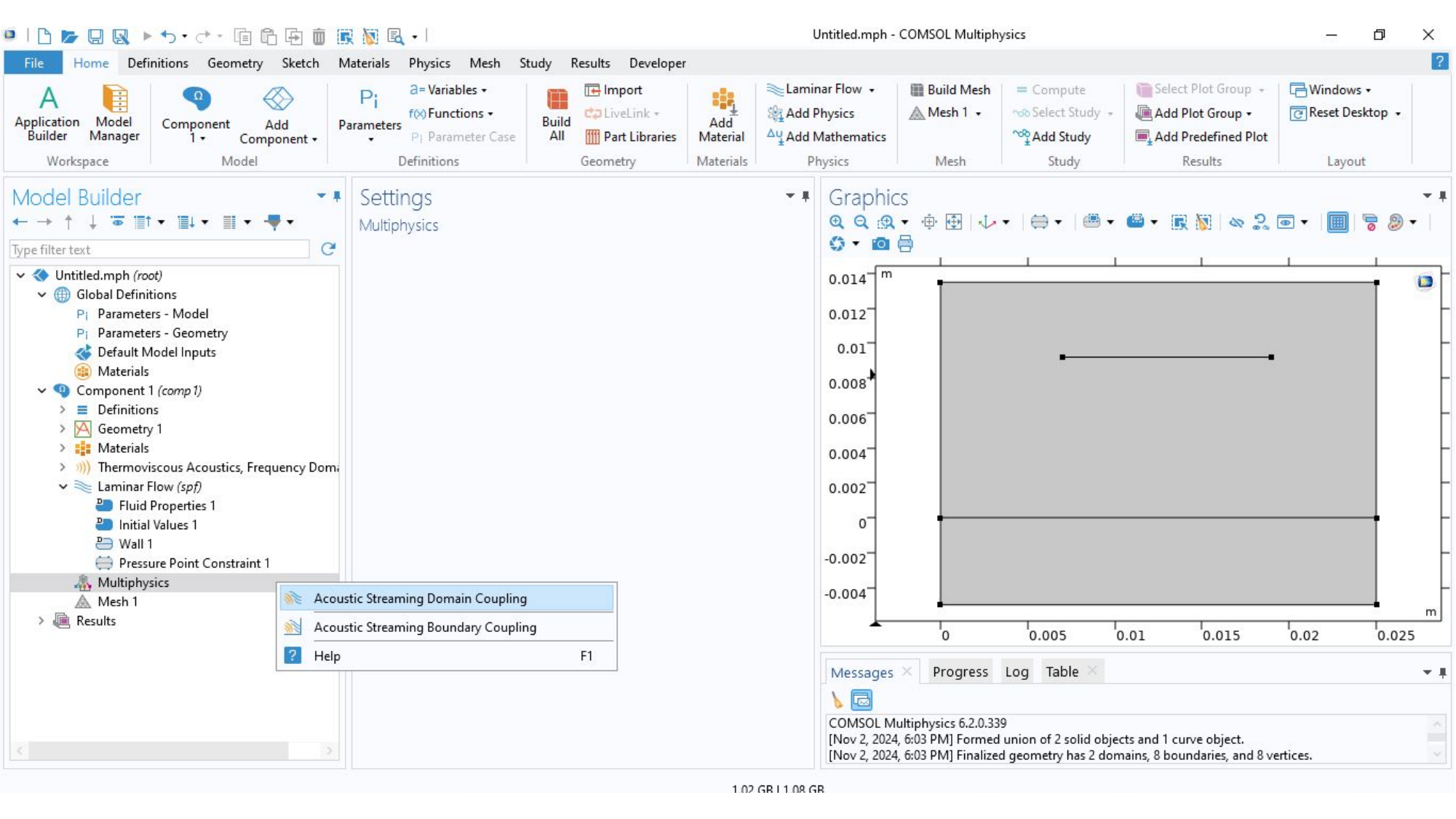

## Slide 93
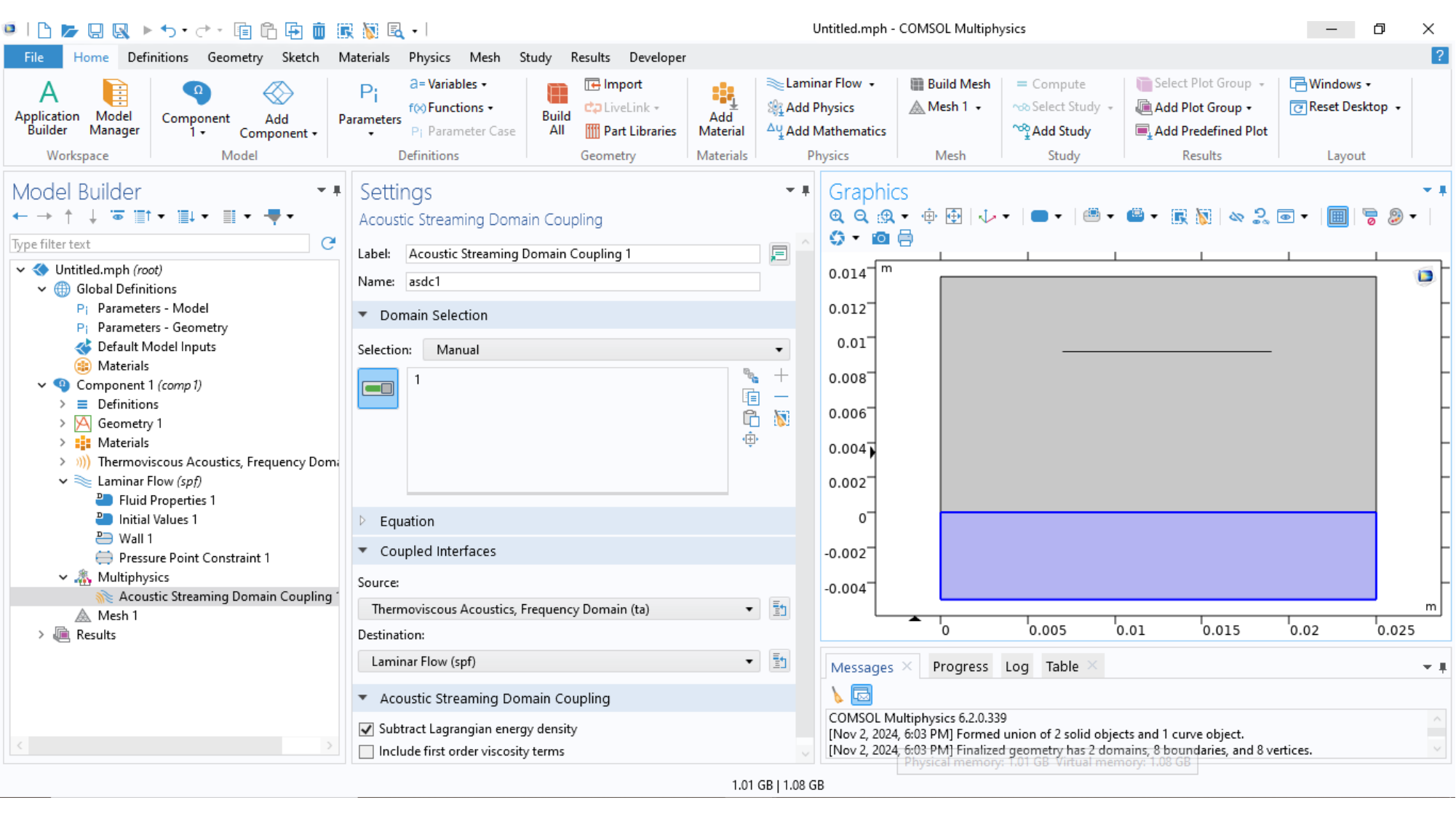

## Slide 94
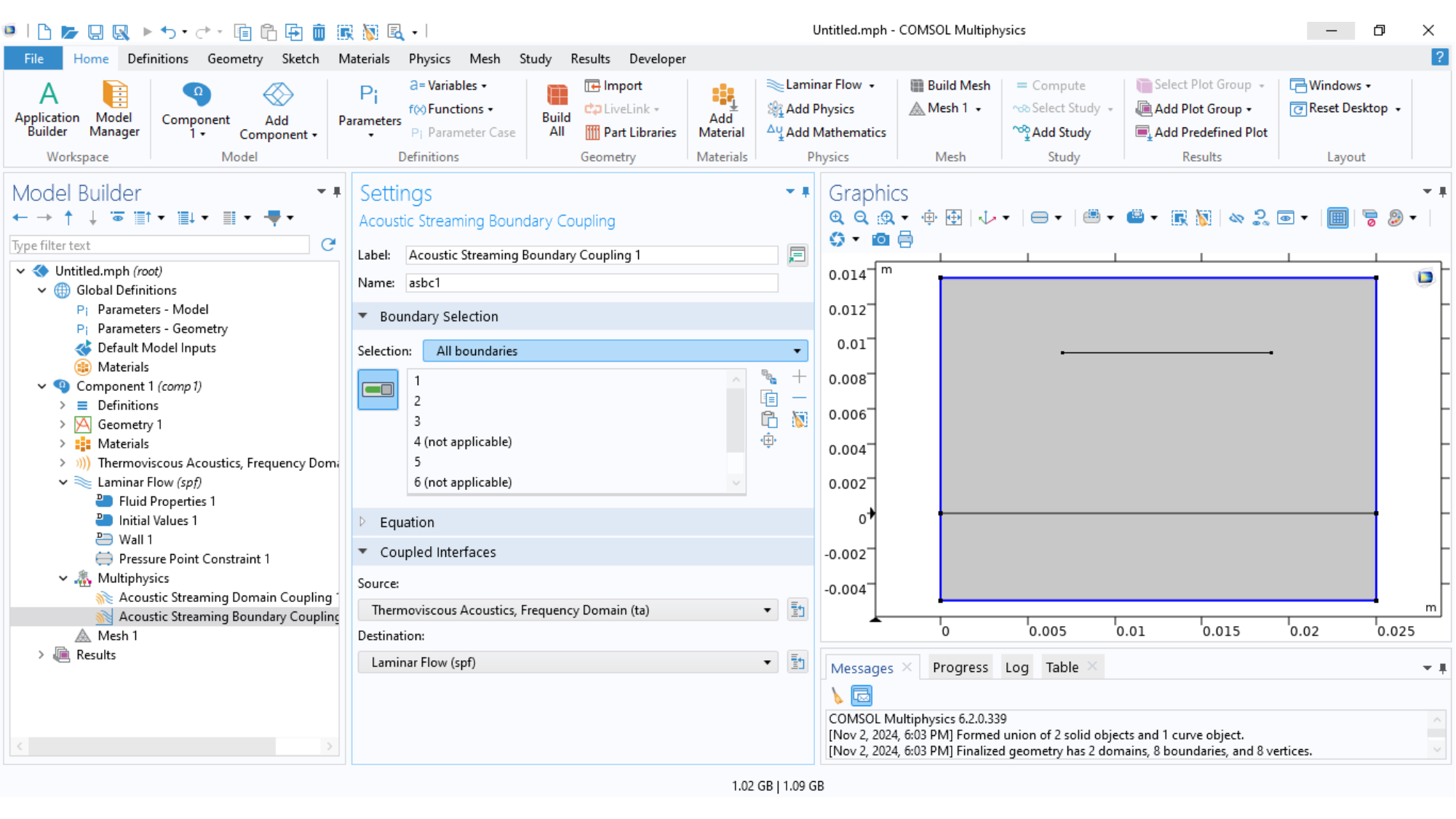

## Slide 95
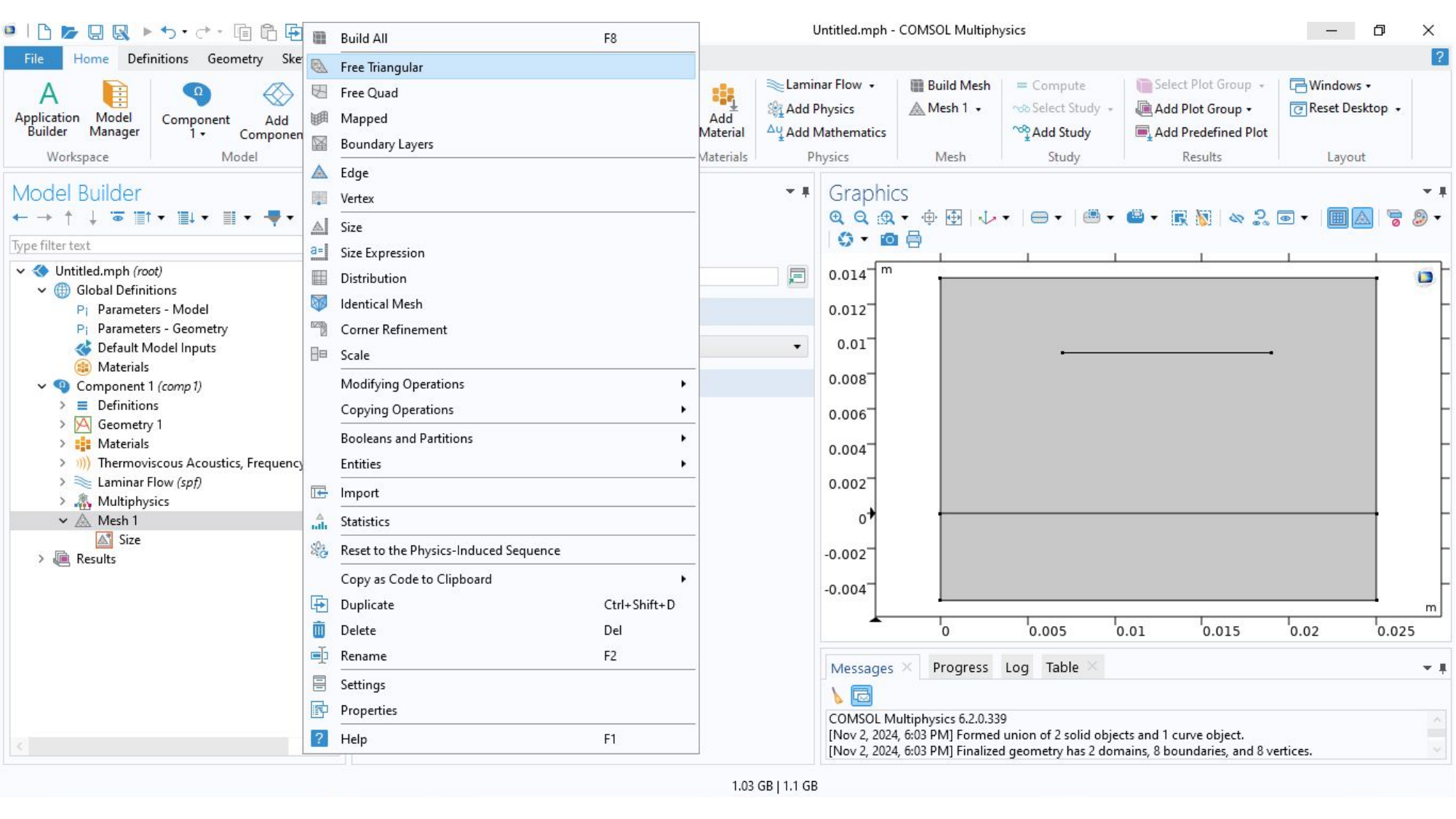

## Slide 96
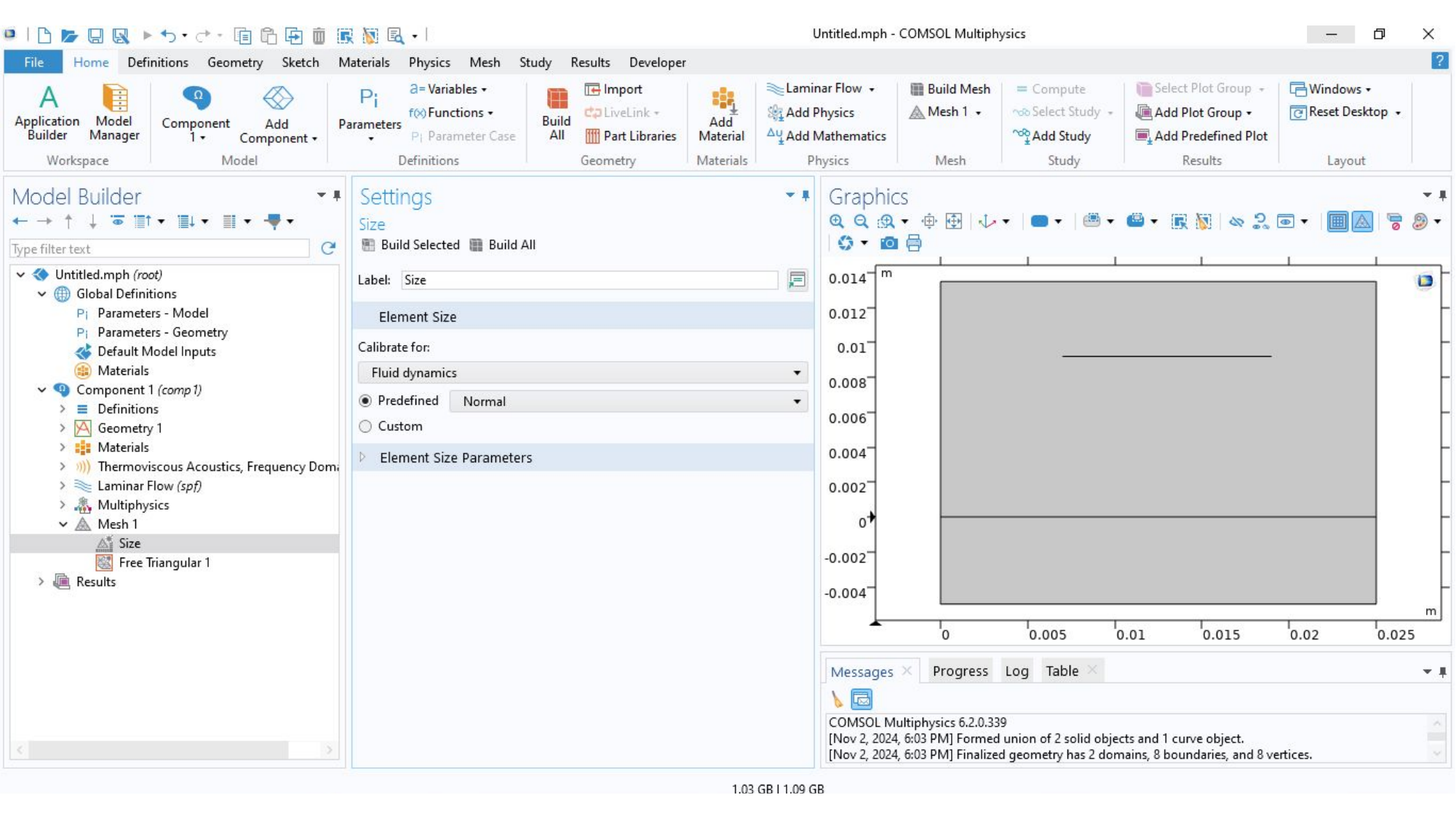

## Slide 97
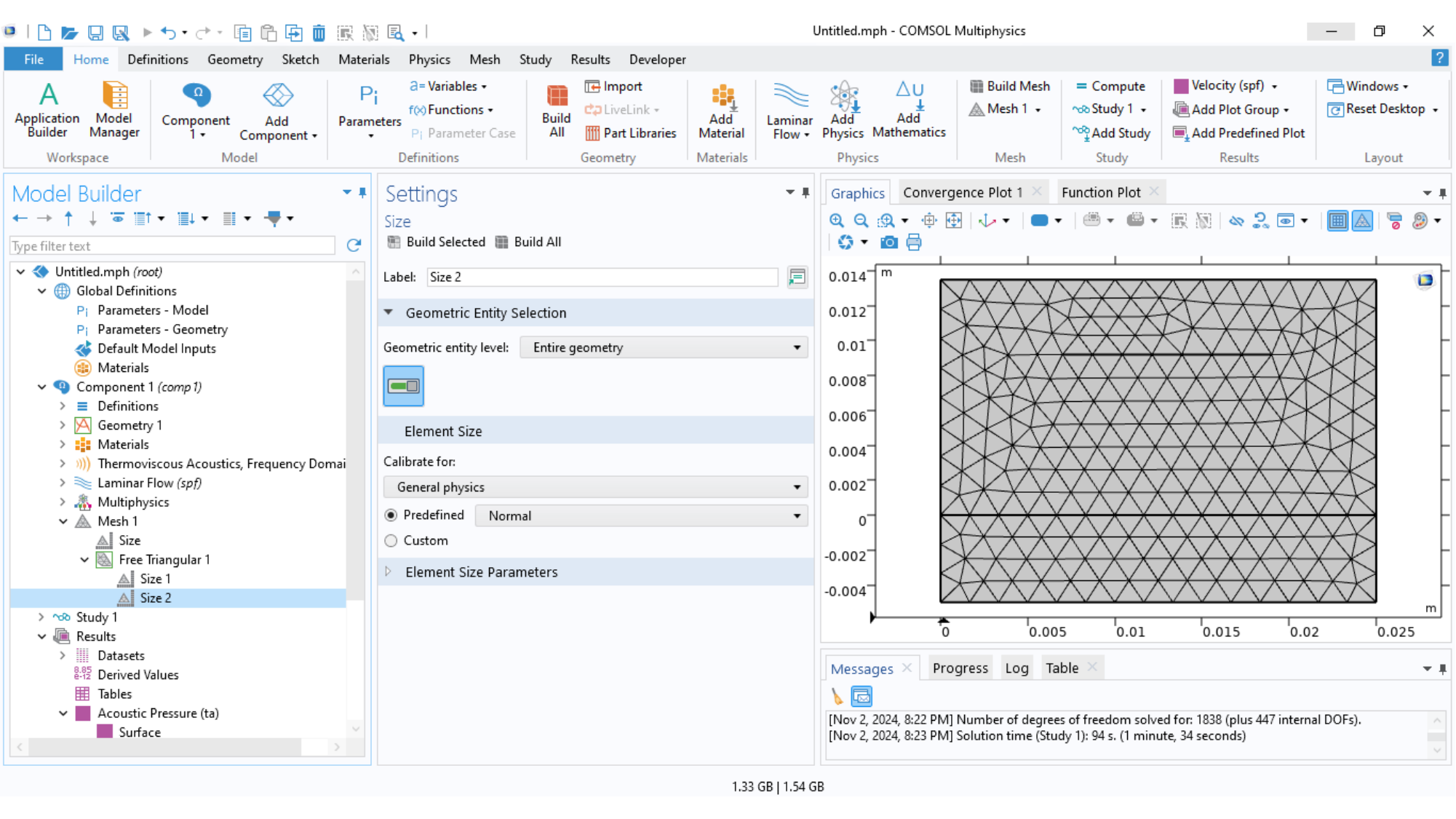

## Slide 98
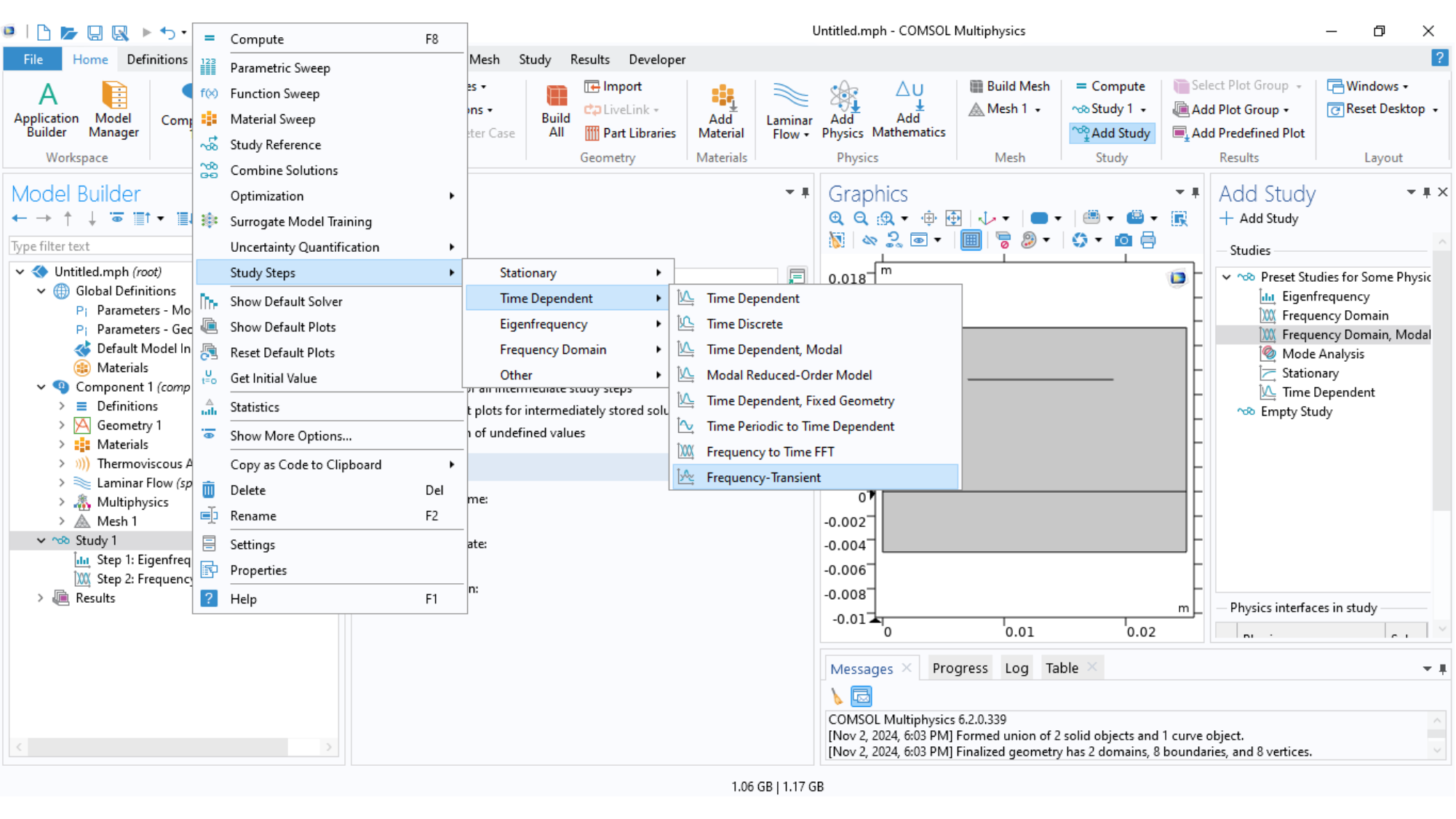

## Slide 99
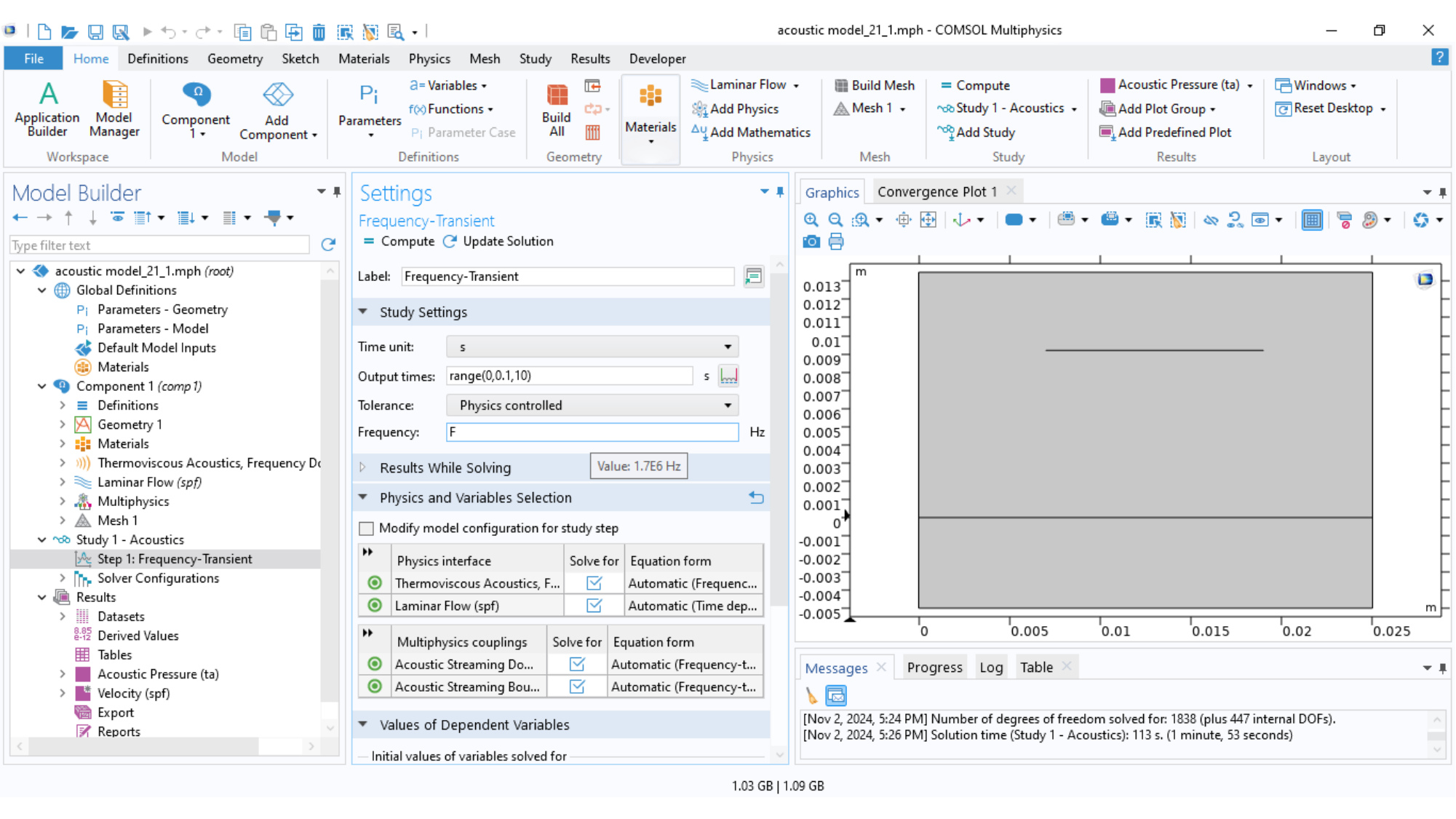

## Slide 100
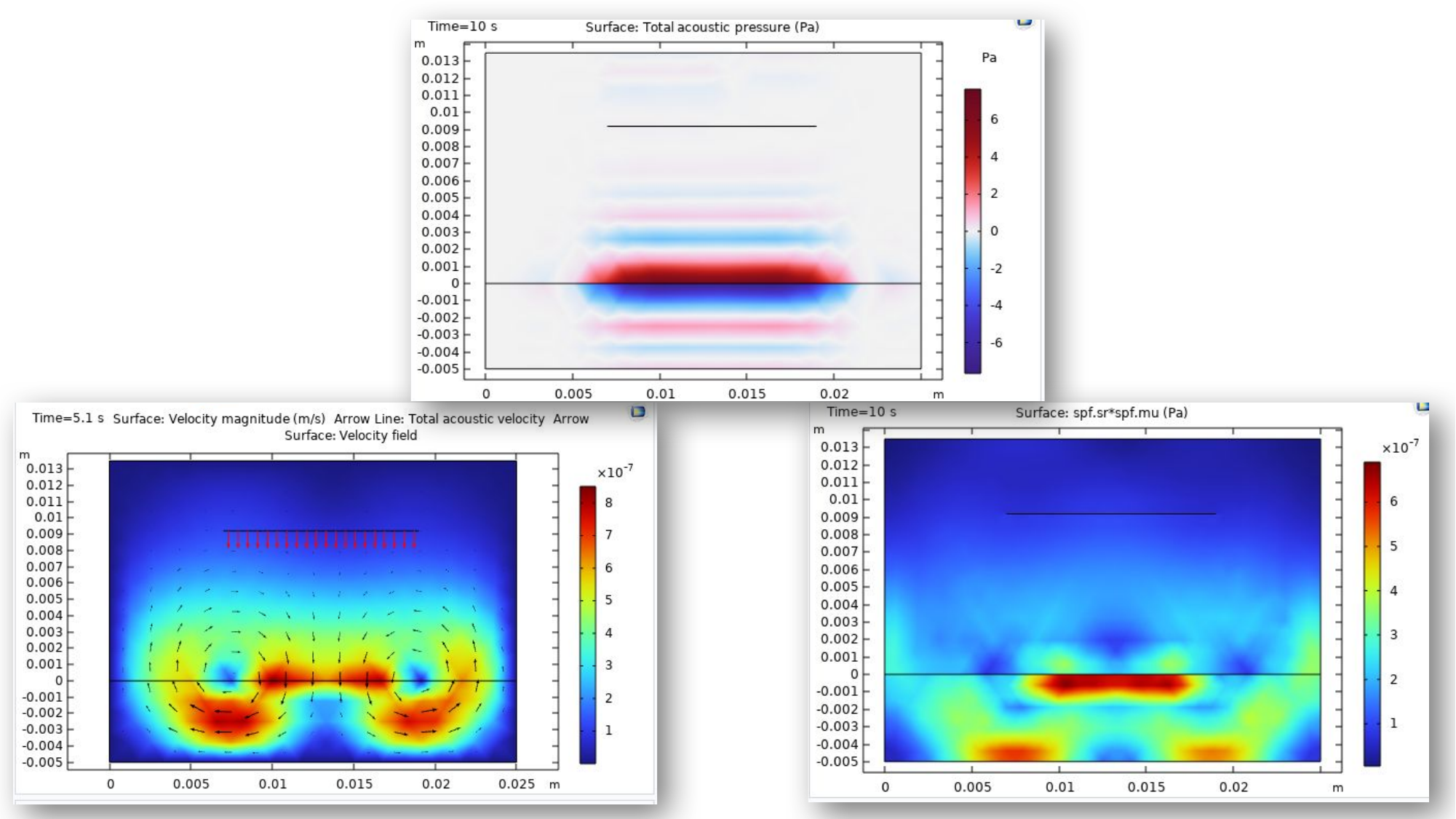

## Slide 101
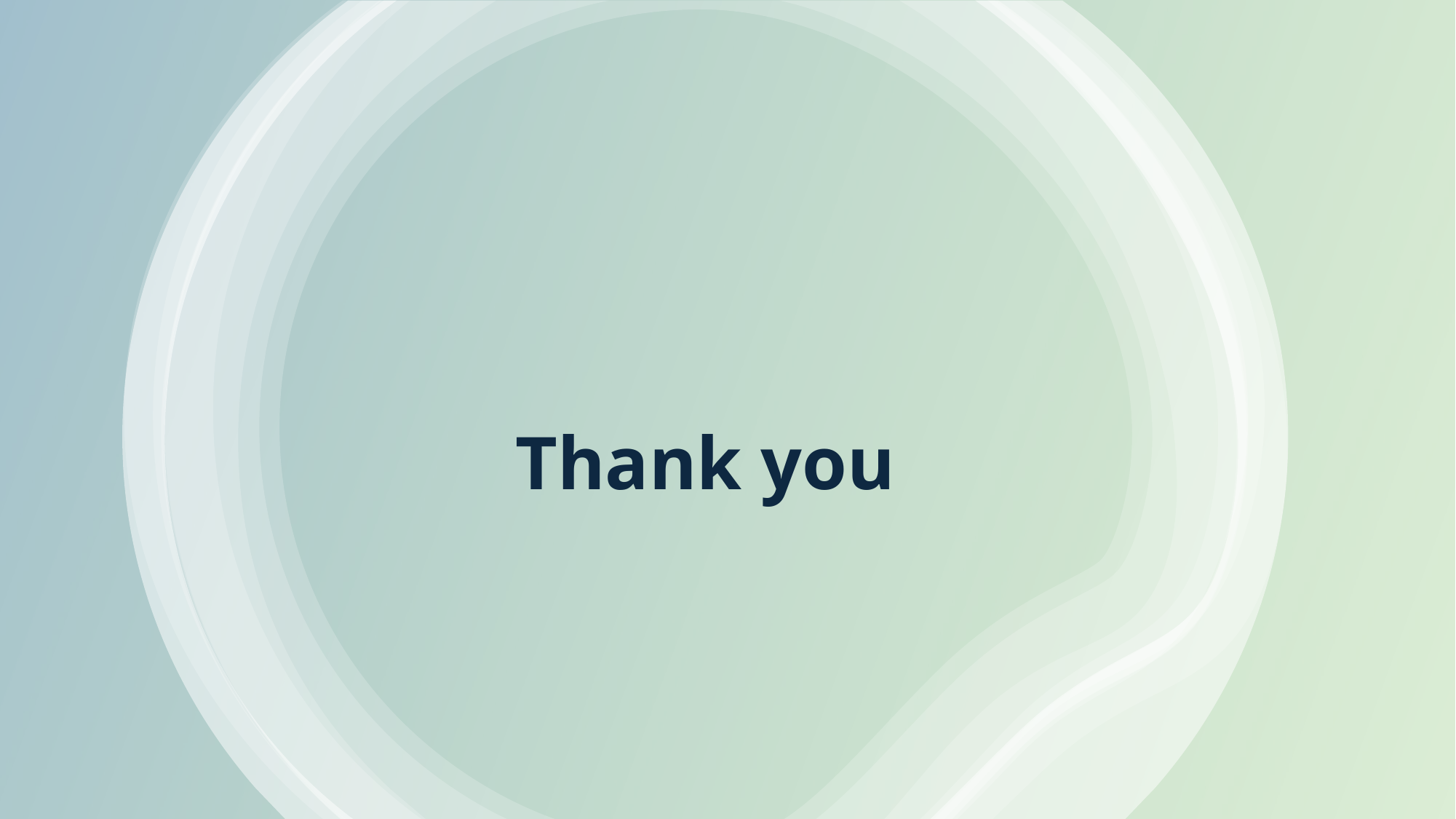

Thank you
