# Supplementary material for: Numerical investigation of ultrasound-induced acoustic streaming and shear stress for blood clot manipulation
Source: Sci Rep. 2026 Apr 20;16:12891. doi: 10.1038/s41598-026-44521-5 (PMC13096658; doi:10.1038/s41598-026-44521-5)
Supplement: Supplementary file 4 — Supplementary Material 4 [file 41598_2026_44521_MOESM4_ESM.docx]

**Data supplementary**

**Simulation Setup:**

1. **Geometry Creation:** A rectangular domain with dimensions of 0.5 cm in height and 2.5 cm in width was created to represent the blood vessel, and PML was used as an added layer with a thickness of 0.01 cm on the left, right, and top.
2. **Transducer Placement:** An acoustic transducer was modeled as a line segment, 1.2 cm in width, positioned in the center of the blood vessel domain to generate acoustic waves.
3. **Thrombus Modeling:** An ellipse geometry with semi-major and semi-minor axes of 0.1 cm was introduced within the center of a rectangular domain to simulate the thrombus. The model was created with the thrombus as a full ellipse.
4. **Material Definition:** The blood vessel was modeled as water with standard fluid properties. The thrombus was defined as a viscous fluid with specific dynamic viscosity.

| **Material** | **Density (kg/m³)** | **Sound Speed (m/s)** | **Viscosity (Pa⋅s)** |
| --- | --- | --- | --- |
| **Vessel** | 1053 | 1650 | 0.3 |
| **Thrombus** | 1080 | 1650 | 0.4 |
| **Water** | 998 | 1495 | 0.000893 |

**Physics Definition:**

1. **Acoustic Pressure Field:** The acoustic pressure field generated by the transducer was simulated using the Pressure Acoustics module on all domains. Apply ultrasound waves at a frequency of 2 MHz and an acoustic pressure of 2 MPa (Thermally conducting and viscous, different frequencies and acoustic pressures were applied).
2. **Fluid Flow:** The fluid flow induced by the acoustic waves (acoustic streaming) was simulated using the Laminar Flow module, coupled with the acoustic pressure field.
3. **Shear Stress Calculation:** Shear stress on the thrombus surface was calculated based on the simulated fluid flow $: \left( \boldsymbol{\tau}=\boldsymbol{\mu}\frac{\boldsymbol{dv}}{\boldsymbol{dz}} \right)$
   - - Prior research has demonstrated that the clot lysis rate is increased under higher shear stress (41 dyne/cm^2) and converted the value to Pascal = 4.1 Pa. If the shear stress exceeds a certain threshold (4.1 Pascals), the thrombus begins to disintegrate.

**Parameter Variation:**

1. **Frequency Sweep:** Simulations were run across various acoustic wave frequencies from 300 kHz to 15 MHz
2. **Pressure Sweep:** Simulations were conducted using various acoustic pressure levels ranging from 20 kPa to 2 MPa.
3. **Transducer Position Variation:** The position of the acoustic transducer relative to the thrombus varied in different simulations.

**Analysis and Results:**

1. **Acoustic Streaming Velocity:** The fluid flow velocity above the thrombus surface was analyzed, and the impact of acoustic streaming induced by ultrasound waves on the clot was investigated by varying several parameters.
2. **Acoustic Pressure Distribution:** The spatial distribution of the acoustic pressure field generated by the transducer within the blood vessel and around the thrombus was visualized and analyzed for different frequencies and transducer positions.
3. **Shear Stress Evaluation:** The magnitude and distribution of shear stress on the thrombus surface were evaluated. The suggested numerical model successfully generated several shear stress values, the highest of which was 10.894 Pascals (Pa) above the clot surface, due to acoustic streaming, and were compared to the 4.1 Pa critical threshold for thrombus fragmentation.
4. **Applying ultrasound to the** **thicker vessels:** After the addition of a thicker vessel above the clot in the vessel wall, the resulting decrease in value is to 2.9039 Pa, and the shear stress value increases with the increase in applied acoustic pressure.

-----------------------------------------------------------------------------------------------------------------------------

- Preliminary work involved constructing a model similar to that described in [^1^], focusing on acoustic streaming flows and vortices above the thrombus to facilitate its breakdown. This initial model successfully mimicked the acoustic streaming effects and vortices observed in that study.


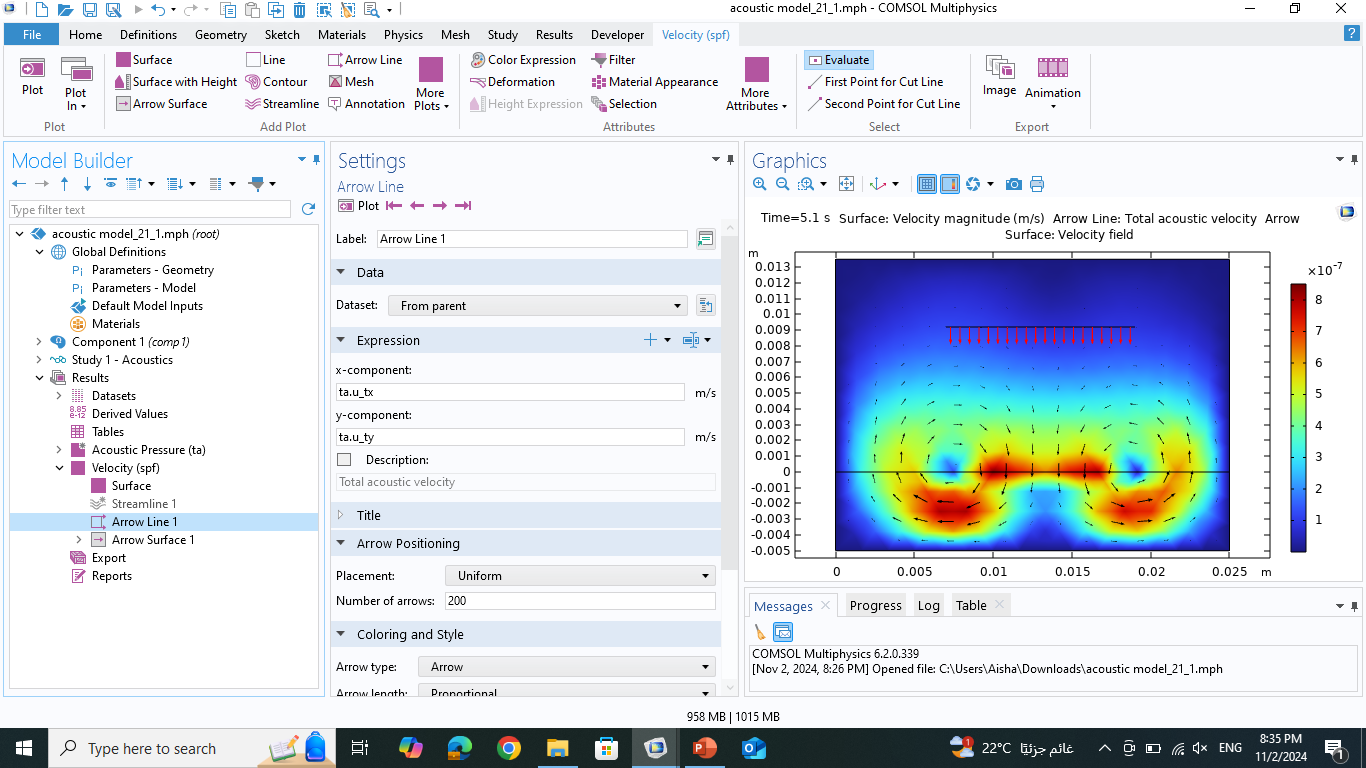

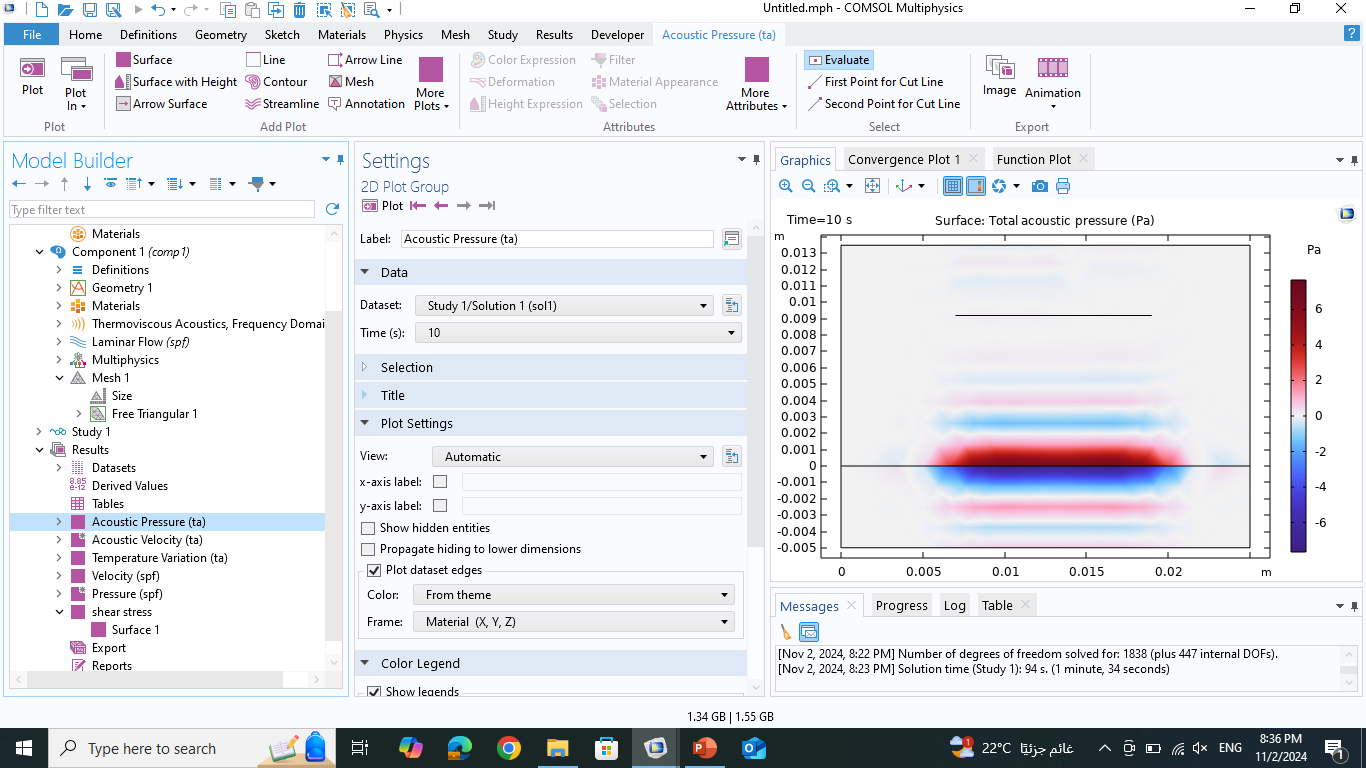


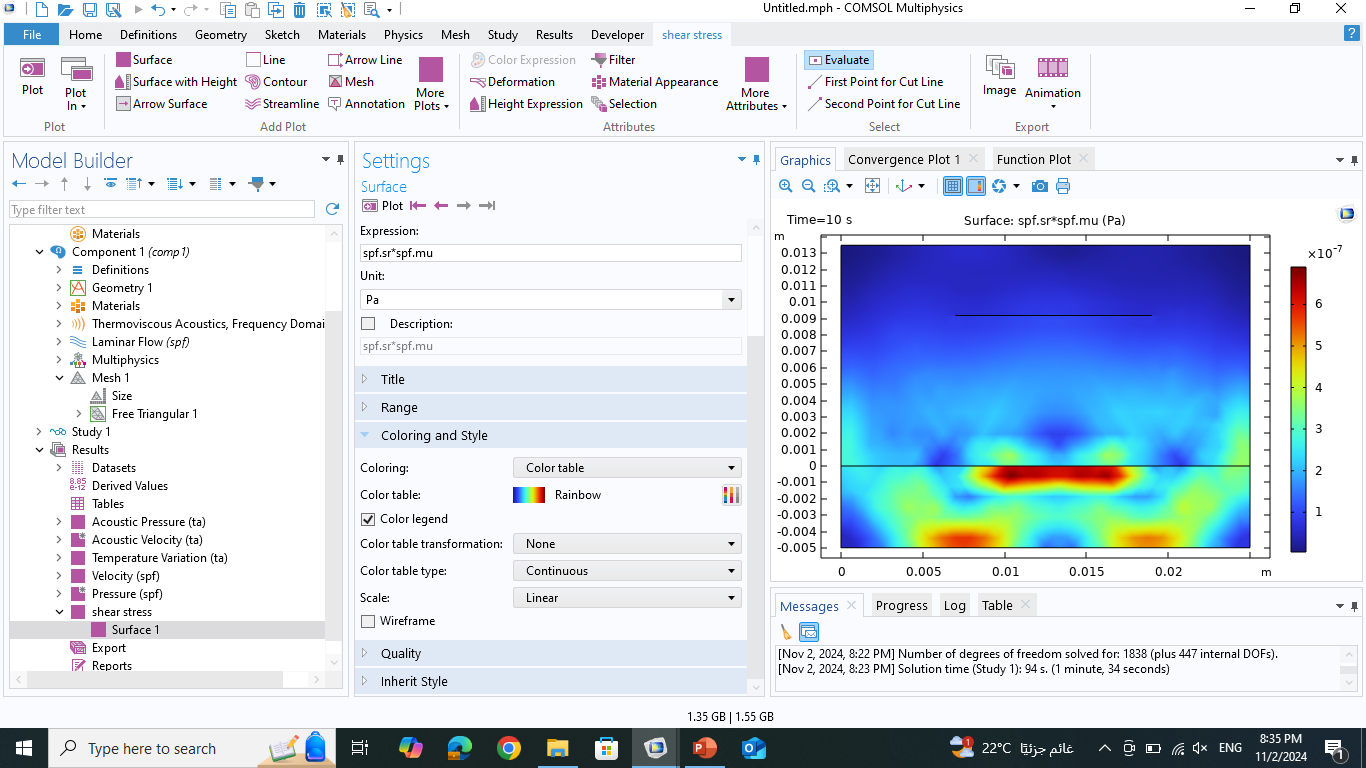


However, the original research paper employed the Thermoviscous Acoustics, Frequency Domain interface, which is quite complex, demands high-performance hardware, and is primarily designed for small-scale simulations due to its detailed modeling of viscous and thermal losses, making it computationally expensive.

Consequently, for this study, we modified the model and utilized the Pressure Acoustics, Frequency Domain interface. "This interface offers greater ease of use and computational efficiency, allowing us to introduce modifications such as simulating the shape of a clot more easily.

1. Zhang, Q. *et al.* Optimized acoustic streaming generated at oblique incident angles to improve ultrasound thrombolysis effect. *Med Phys* 49, 5728–5741 (2022).
